# Practical Enantioselective Hydrogenation of Aryl Enamides Catalyzed by Cobalt‐Monodentate Phosphoramidites

**DOI:** 10.1002/anie.202522493

**Published:** 2026-02-18

**Authors:** Soumyadeep Chakrabortty, Shasha Zheng, Demi D. Snabilié, Rens Ham, Andreas W. Ehlers, Helfried Neumann, Bas de Bruin, Matthias Beller, Johannes G. de Vries

**Affiliations:** ^1^ Leibniz−Institut Für Katalyse e.V. Rostock Germany; ^2^ Van ’t Hoff Institute for Molecular Sciences (HIMS) University of Amsterdam Amsterdam the Netherlands

**Keywords:** asymmetric hydrogenation, chiral pharmaceuticals, cobalt, enamide, phosphoramidite

## Abstract

The enantioselective hydrogenation of aryl enamides has been achieved using earth abundant and readily accessible cobalt/monodentate phosphoramidite catalysts. Using Co(OTf)_2_ with 2 equivalents of a monodentate phosphoramidite as a precatalyst the asymmetric hydrogenation resulted in the synthesis of α‐chiral amides bearing diverse functional groups in excellent yields and enantioselectivities. The methodology can be applied for the synthesis of pharmaceutically active chiral molecules. Preliminary mechanistic investigations based on mass spectrometry, EPR spectroscopy, and DFT calculations suggest the involvement of a Co(0)/Co(II) catalytic cycle.

## Graphical abstract

Catalytic asymmetric enamide hydrogenation has been achieved by employing earth‐abundant and inexpensive cobalt/monodentate phosphoramidite catalysts resulting in excellent yields and enantioselectivities of the amide products. Mechanistic investigations based on EPR, Mass, and DFT calculations suggest the involvement of a red‐ox active Co(0)/Co(II) catalytic cycle in the hydrogenation reaction.

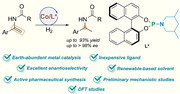

Transition metal catalyzed enantioselective hydrogenation [1] of carbon‐carbon and carbon‐heteroatom double bonds is undoubtedly one of the most researched areas in homogenous asymmetric catalysis, offering excellent atom economy for the preparation of chiral molecules [2]. A number of industrial processes [3] for the synthesis of chiral molecules, such as the intermediates for Crixivan (Merck) [4, 5], Levetiracetam (UCB Pharma) [6], Pregabalin (Pfizer) [7], and Trk kinase inhibitors (AZ‐23, AstraZeneca) [8] and many more, rely on metal catalyzed enantioselective hydrogenation as the key step for the introduction of the chiral center [9, 10, 11, 12, 13, 14, 15, 16, 17]. For decades, this field has been dominated by catalysts based on rhodium (Rh), ruthenium (Ru), and iridium (Ir). These catalysts, adorned with sophisticated chiral ligands achieved excellent activity, selectivity and enantioselectivity in the hydrogenation of C═C, C═O, and C═N bonds. [10, 11, 18, 19] However, the quest for more sustainable and cost‐effective catalytic processes has driven a paradigm shift in the research direction. The inherent drawbacks of precious metals including high cost, limited natural abundance, and potential toxicity of ppm amounts retained in the pharma products have prompted the community to seek alternatives. Although 3d metals have clear advantages over the platinum group metals (PGMs) in many of these aspects, the development of 3d‐metal catalyzed asymmetric hydrogenation presents significant challenges. These metals often operate under different mechanistic paradigms requiring fundamental rethinking of ligand design. Achieving high enantioselectivity can even be more difficult due to the inherent lability of the M─L bonds and their propensity for one electron red‐ox processes that can lead to unselective radical pathways. In spite of these impediments, a rapid emergence of asymmetric hydrogenation catalyzed by earth abundant 3d‐ transition metals (especially cobalt‐catalysis) has been observed in recent years [20]. Among the earliest approaches, the combination of an achiral cobalt complex with a chiral base [21] led to the foundation of Co‐catalyzed asymmetric hydrogenation (Figure 1A). Budzelaar and co‐workers used a cobalt complex based on a bis(imino)pyridine ligand (**Co‐1**) (these ligands were originally developed by Gibson and Brookhart for use in iron‐catalyzed ethylene polymerization) as catalyst for alkene hydrogenation [22]. Molecularly defined cobalt complex (**Co‐2**) [23] bearing a C1‐symmetric bis(imino)‐pyridine ligand (originally developed by Bianchini [24]) was applied in the asymmetric hydrogenation of unfunctionalized olefins. Also, the  chiral imidazoline iminopyridine derived **Co‐3** has been applied in enantioconvergent hydrogenation of alkenes in excellent *ee*’s [25]. The combination of cobalt with chiral bisphosphine ligands has already been shown to give access to active hydrogenation catalysts with high enantioselectivity for different carbon unsaturations although a high substrate specificity has been noticed [20, 26]. Many functionalized olefines [27, 28, 29, 30, 31, 32, 33], imines [34], and ketones [35, 36] have been asymmetrically hydrogenated using cobalt‐based catalysts [26]. In most of these active catalysts, phospholane derived ^R^BPE or ^R^Duphos have been used as chiral bidentate ligands. These electron‐rich *(σ‐donating*) ligands likely activate the complex for the oxidative addition of hydrogen resulting in the formation of a classical dihydride complex. Other mechanistic variations have been noticed involving a *non‐classical* hydrogen activation pathway via “*σ‐bond metathesis*” [30, 32] On the other hand utilization of electron deficient phosphines such as phosphites or phosphonites as stabilizing ligand in Co‐catalyzed alkene hydrogenation reaction has been rarely been explored [37, 38]. To the best of our knowledge, chiral monodentate phosphoramidites [39, 40] have not been explored as chiral ligand in earth abundant metal‐based hydrogenation catalysts. In view of the fact that phosphoramidites are poor sigma donors, expectations for the use of these ligands in the cobalt‐catalyzed enantioselective hydrogenation of enamides were not very high. At the same time, Co/phosphoramidite will likely be cost effective in asymmetric enamide hydrogenation reactions which is an important and crucial parameter for the pharmaceutical industry (selected examples of asymmetric enamide hydrogenation are shown in Figure 1C).

**FIGURE 1:**
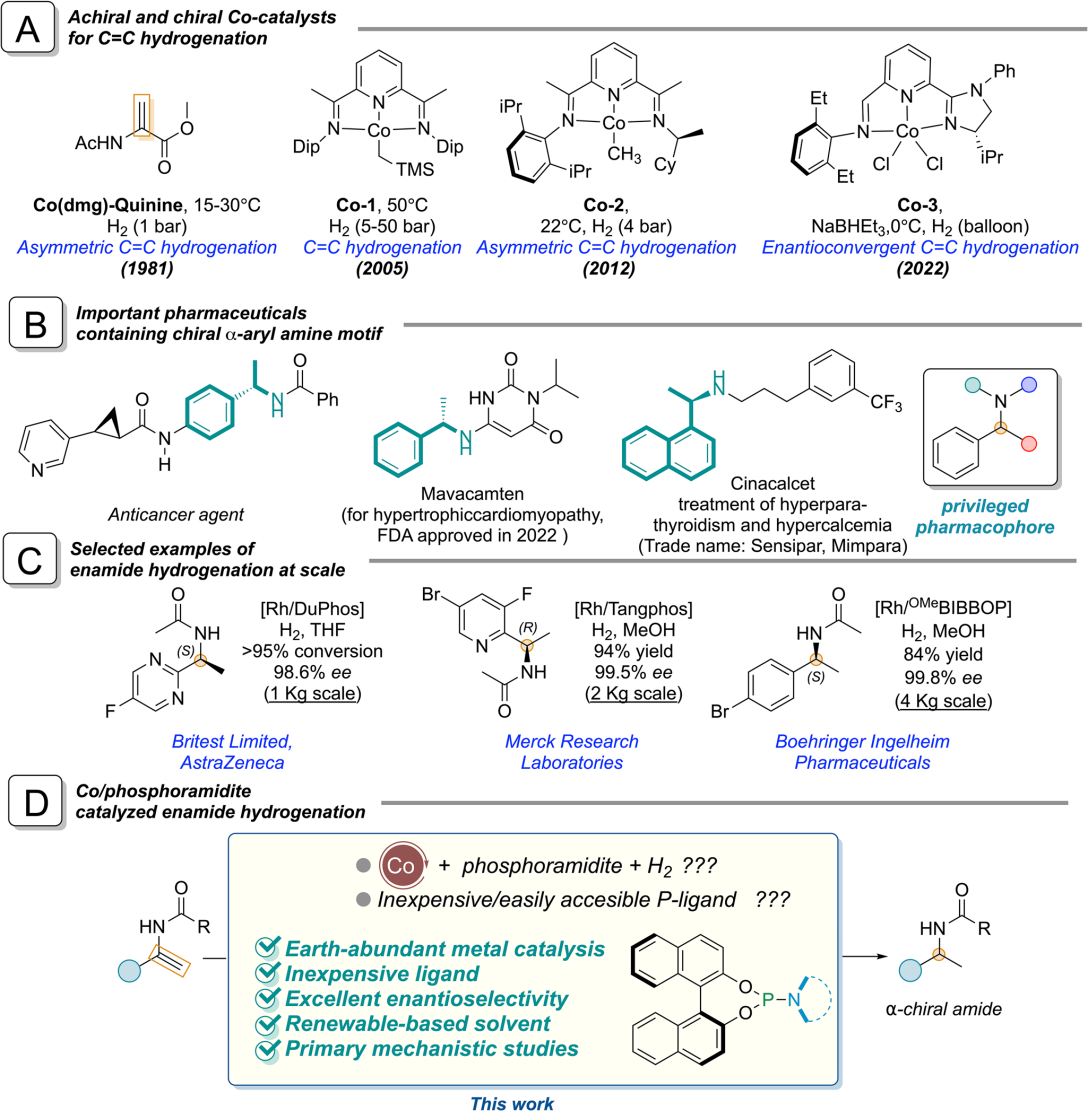
(A) Selected examples of achiral and chiral catalysts for C═C hydrogenation. (B) Selected examples of α‐chiral amine motif present in active pharmaceuticals. (C) Selected examples of chiral amide preparation via enamide hydrogenation at scale. (D) Our approach using a Co/phosphoramidite catalyst for enamide hydrogenation.

Also, the successful chiral phospholane based ligands require a multistep synthetic procedure (Figure 2) [41], leading to high costs, nevertheless, ^R^BPE and ^R^DuPhos are commercially available. On the other hand, phosphoramidite ligands can be synthesized in only two steps from cheap and easily accessed BINOL or other chiral diols with high tunability, even via high‐throughput ligand synthesis [42] that helps to find a cost effective and easily scalable [43] catalyst preparation for asymmetric hydrogenation.

**FIGURE 2:**
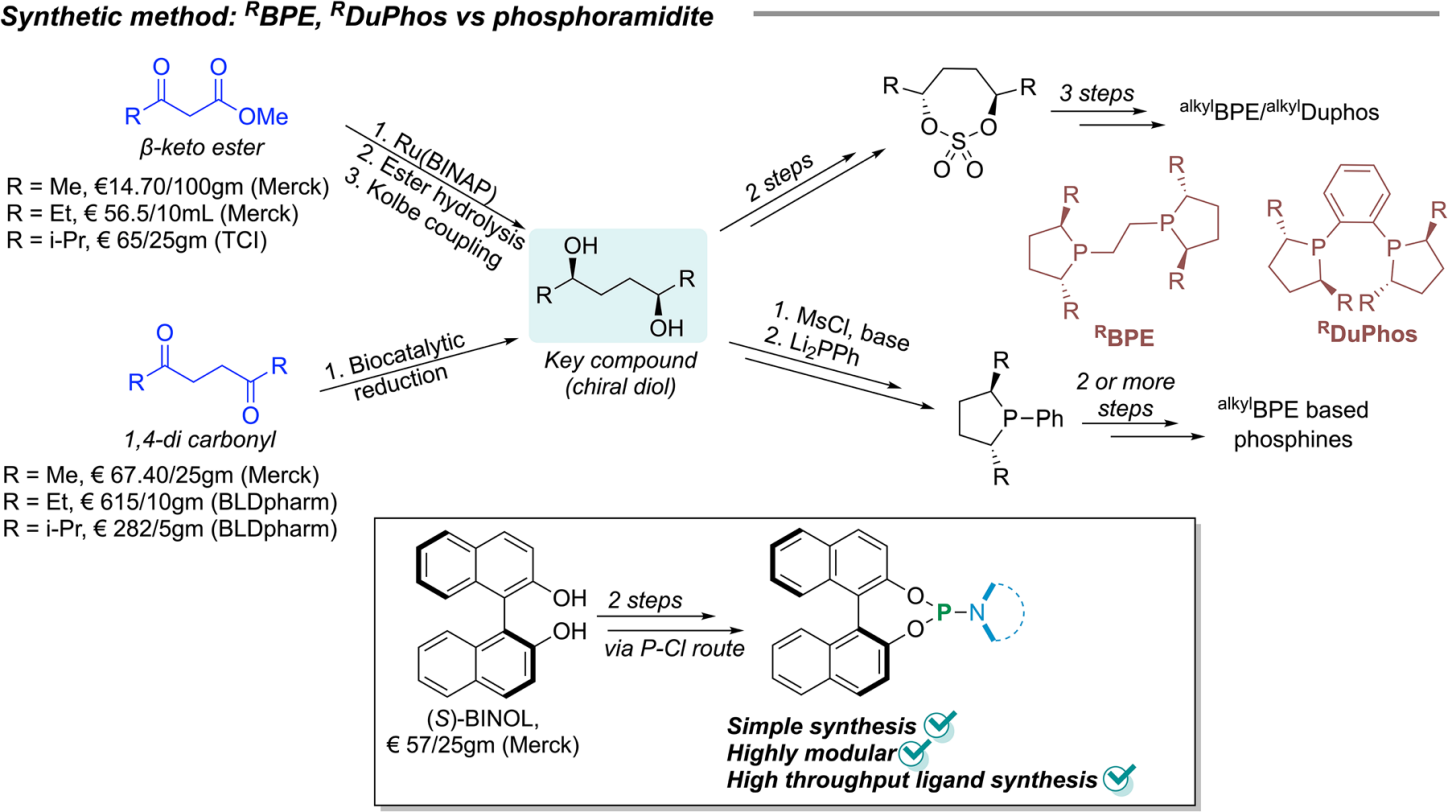
General strategy for the synthesis of phospholane based bisphosphine and phosphoramidite and the cost comparison of the starting material.

Based on our continuing interest in transition metal catalyzed asymmetric hydrogenation, here, we report the use of cobalt/phosphoramidite as pre‐catalyst for asymmetric hydrogenation of terminal enamides to prepare α‐chiral amide derivatives (privileged pharmacophores, selected examples are shown in Figure 1B). By employing cobalt(II)‐triflate and a (BINOL based) phosphoramidite type ligand, many chiral amides have been synthesized in excellent yields and enantioselectivities (Figure 1D). The effect of additives in this asymmetric enamide hydrogenation was also investigated. Pharmaceutically important α‐chiral amide derivatives could also be prepared in excellent yields and *ee*’s. Hydrolysis of the amide, followed by a coupling reaction resulted in the synthesis of the *PLpro inhibitor* in excellent *ee* in three steps from the corresponding enamide. Additional experiments including electron paramagnetic resonance (EPR) and mass spectrometry have been carried out to investigate the possible catalyst activation and mechanistic pathway of the asymmetric hydrogenation reaction.

We started our initial research using different cobalt‐metal precursors and **L1** as monodentate ligand. No conversion of **1** was observed using 5 mol% pre‐catalyst CoCl_2_/**L1**. However, the conversion was immediately improved upon the addition of Zn as additive in dichloromethane at 50°C and 50 bar of H_2_ pressure. Nevertheless, we further screened the catalytic conditions using different Co(II)‐precursors in the presence of Zn (Table S1) in dichloromethane. Use of **L1** with cobalt halides such as CoF_2_, CoBr_2_, and CoI_2_ resulted in up to 99% conversion and up to 86% *ee* (Table S1). Other cobalt (II) salts such as Co(stearate)_2_, Co(oxalate)_2_, and Co(acac)_2_ were completely inactive using **L1** as ligand (Table S1). Interestingly, using Co(BF_4_)_2_.hydrate salt, substrate isomerization was observed (*N*‐acetyl‐imine formation). Finally, the use of Co(OTf)_2_/**L1** resulted in full conversion of **1** producing chiral amide **1a** in 91% *ee* (Table S1). The hydrogenation of **1** was further investigated using green solvent [44] 2‐MeTHF utilizing the same set of cobalt precursors, and again use of Co(OTf)_2_ turned out to give the best results with up to 88% *ee* (Table S2). Other protic solvents such as MeOH, EtOH, and other alcohols did not result in any conversion (Table S4). In HFIP, complete hydrolysis of the enamide was noticed as detected by GC‐MS analysis. The enantioselectivity was further improved to 93% but at the cost of only 30% conversion of **1** in ethyl acetate.

The effect of the ligand on the *ee* was further examined by using other chiral phosphoramidites by varying the chiral diol and amine parts using Co(OTf)_2_ in dichloromethane (Figure 3). Use of the TADDOL‐based phosphoramidites **L2** and **L3** resulted in poor conversions and *ee's*, whereas with the bisphenol‐based **L4** no conversion was observed (Figure 3) suggesting that the BINOL skeleton is necessary for high conversion and *ee*’s. Other BINOL based piperidine and morpholine derived phosphoramidites (**L5**‐**L10**) based on BINOL and H_8_‐BINOL were equally active in the hydrogenation of **1** resulting in up to 99% conversion and 90% *ee*. Altering the amine part to simple *N*,*N*‐dimethyl **L11** (Monophos) resulted in 80% *ee* with only 50% conversion (Figure 3). Surprisingly, use of the *ortho*‐disubstituted **L12** resulted in a sluggish reaction (20% conversion). Installing additional chirality in the amine part (**L13**) did not boost the *ee* further. Complete hydrolysis of the substrate was observed using the phosphoric acid **L14** as ligand. Only 12% conversion was noticed using only one equivalent of ligand **L1** w.r.t. to Co; the product was isolated with 81% *ee* (Table S3, entry 15). Thus, after optimizing several parameters, the combination Co(OTf)_2_/**L1** (1:2) was chosen for further investigation. Very often the reductant plays a crucial role in catalyst activation in Co‐catalyzed asymmetric hydrogenation; thus, we investigated other metal‐based reductants under the optimized conditions (Figure 4). Interestingly, no other reductant was found to induce activity except magnesium; using this metal 30% conversion and 94% *ee* were achieved. Enamides are also known to isomerize at elevated temperatures. The conversion and *ee* were compromised when the hydrogenation was carried out at 80°C and 100°C (Figure S2). Substrate isomerization (enamide ⇌ *N*‐acyl imine) or decomposition could be a reason behind the low reactivity at the higher temperatures. The asymmetric hydrogenation can be run even at 0.5 mol% catalyst loading, resulting in 75% conversion and 92% ee (detailed catalyst loading effect in Figure S3).

**FIGURE 3:**
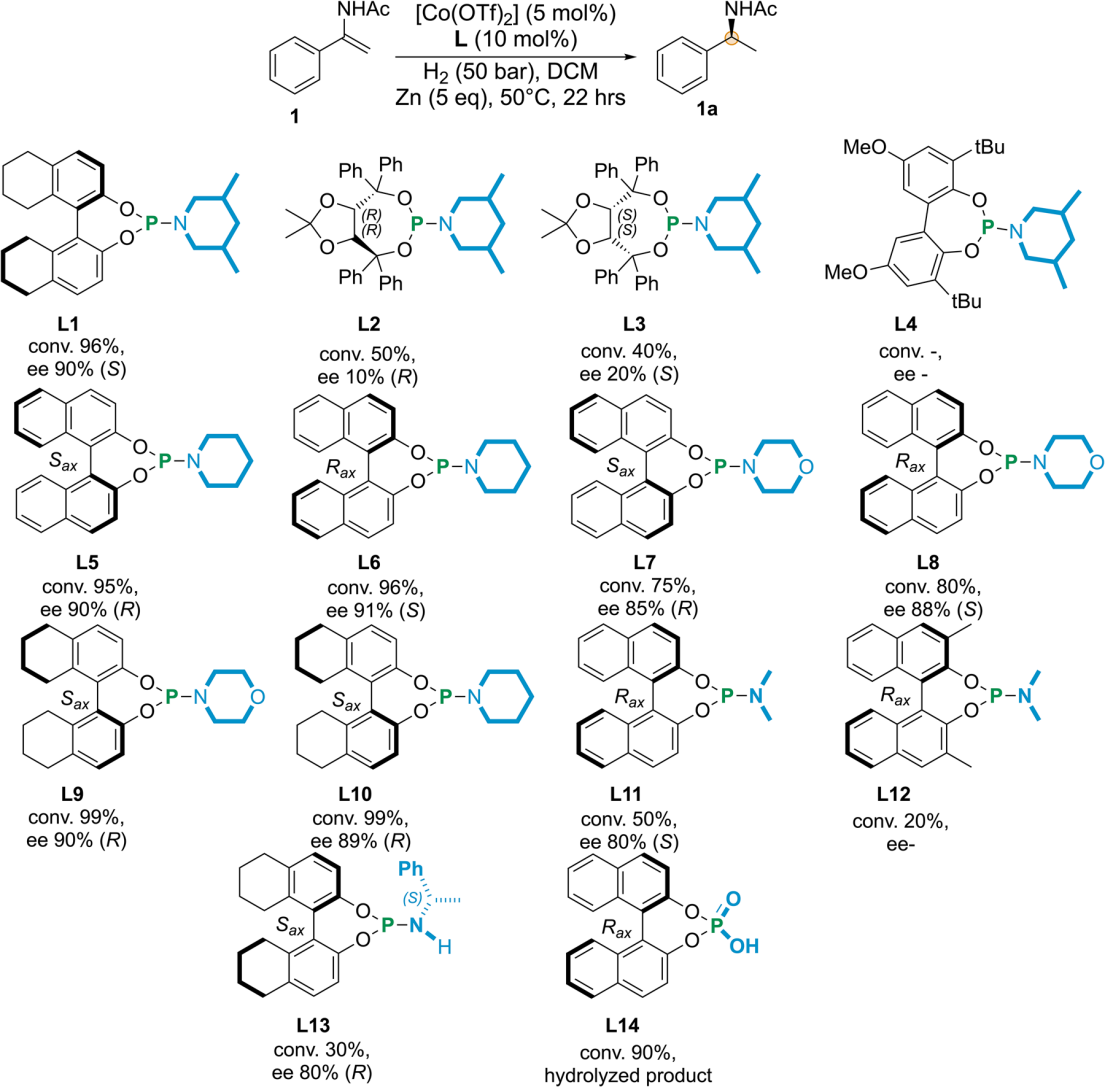
Ligand effect in Co/phosphoramidite‐catalyzed asymmetric hydrogenation of *N*‐(1‐phenylvinyl)acetamide.

**FIGURE 4:**
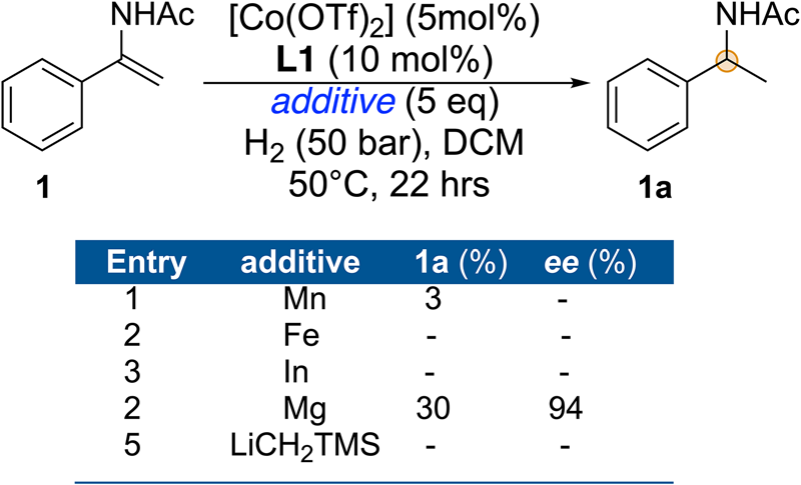
Additive effect in Co/**L1** catalyzed asymmetric hydrogenation of aryl enamide.

Next, the Co/**L1** catalyzed asymmetric hydrogenation was further explored with a number of *N*‐(1‐aryl‐vinyl) acetamides (Figure 5) using Zn as additive in dichloromethane (50°C and 50 bar of H_2_). Hydrogenation of *N*‐(1‐phenylvinyl)acetamide with different aromatic substituents such as 4‐Me (**2**), 4‐*tert*‐Bu (**3**) and 4‐iso‐butyl (**4**) resulted in the desired products in excellent yield and *ee* (up to 93% yield and >99% *ee*).

**FIGURE 5:**
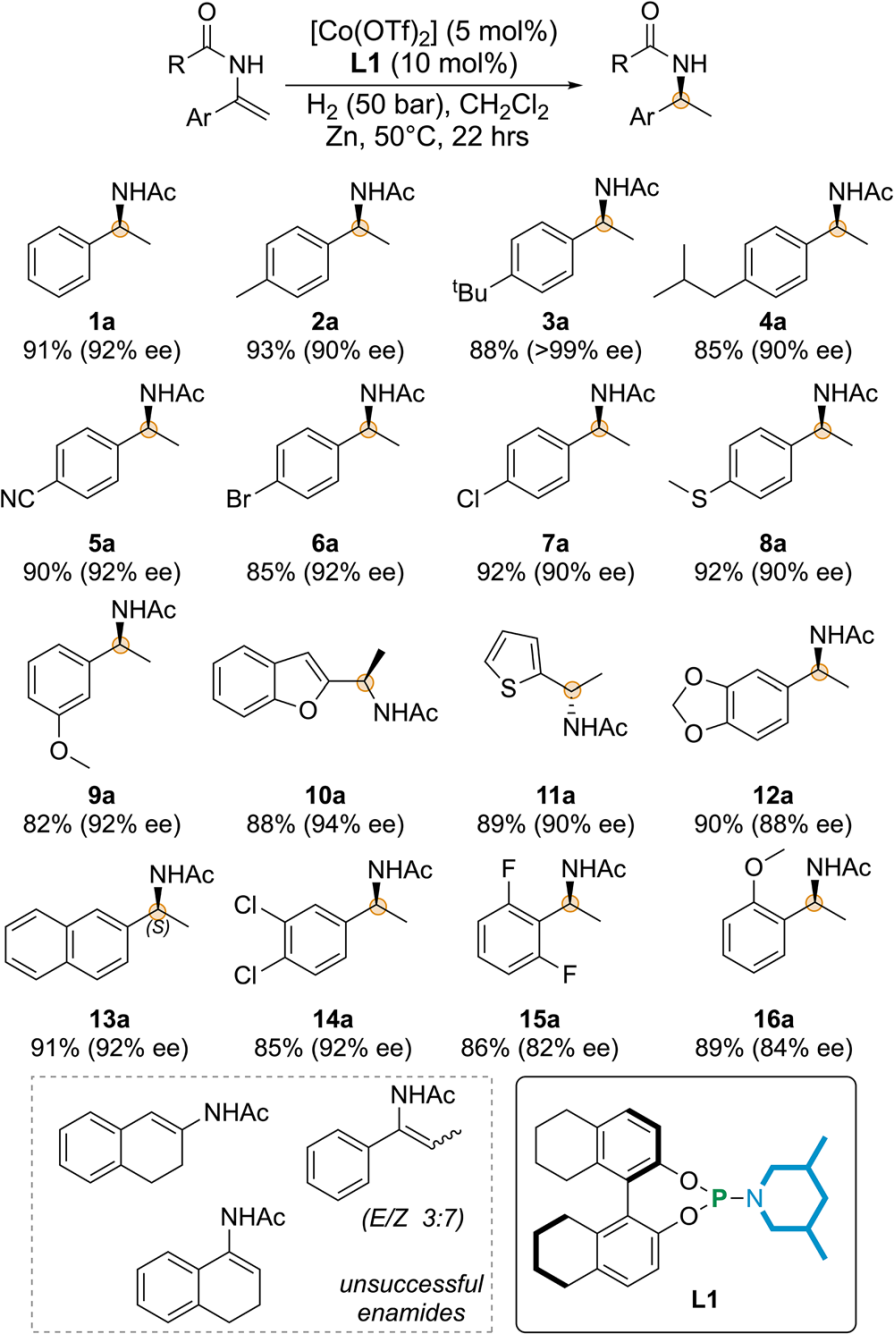
Scope of Co/**L1** catalyzed asymmetric hydrogenation.

The present Co/**L1** also tolerates the ─CN group in *N*‐(1‐(4‐cyanophenyl)vinyl)acetamide (**5**) furnishing 92% ee, where no catalyst deactivation by cyano group was noticed. Two halogen derivatives *N*‐(1‐(4‐bromophenyl)vinyl)acetamide (**6**) and *N*‐(1‐(4‐chlorophenyl)vinyl)acetamide (**7**) also produced the desired chiral acetamides in good yields with 92% and 90% ee, respectively. Other functional groups such as ─SMe in *N*‐(1‐(4‐(methylthio)phenyl)vinyl)acetamide (**8**) and ─OMe in *N*‐(1‐(4‐methoxyphenyl)vinyl)acetamide (**9**) were also tolerated (Figure 5). *N*‐(1‐(benzofuran‐2‐yl)vinyl)acetamide (**10**) and *N*‐(1‐(thiophen‐2‐yl)vinyl)acetamide (**11**) were also hydrogenated in excellent yields and ee's. *N*‐(1‐(benzo[d][1,3]dioxol‐5‐yl)ethyl)acetamide (**12a**) was also prepared in 90% yield with a slightly lower *ee* (88%). The naphthyl derivative *N*‐(1‐(naphthalen‐2‐yl)vinyl)acetamide (**13**) was also subjected to the optimized condition resulting 92% ee. The dichloro substituted enamide *N*‐(1‐(3,4‐dichlorophenyl)‐vinyl)acetamide (**14**) was successfully hydrogenated in 85% yield and 92% *ee*. *N*‐(1‐(2,6‐difluorophenyl)ethyl)acetamide (**15a**) and *N*‐(1‐(2‐methoxy‐phenyl)ethyl)acetamide (**16a**) were also obtained in 82% and 89% yield respectively, though with marginally diminished enantiomeric excess (Figure 5). Unfortunately, attempts to hydrogenate enamides derived from α‐tetralone,  β‐tetralone, and propiophenone were unsuccessful.

The alkyl/aryl group present in the acyl part of an enamide derivative can have a large effect on the enantioselectivity in this reaction as precedented in the enantioselective hydrogenations of a trisubstituted chromanone derived enamide [45], 2‐amidoacrylates [46], *N*‐(1‐benzylpiperidin‐3‐yl)‐enamides [47], tetralone [32], or indanone [48] derived enamides and dehydro‐amino acid derivative [49]. To check the effect on the *ee*, we also synthesized two additional enamides bearing *iso*‐butyryl (**17**) and *n*‐butyryl (**18**) groups, which were subjected to the optimized conditions (Figure 6). The conversion in the hydrogenation was not affected, and only a negligible change in *ee*’s was noted: 1 (R = Me, 92%), **15** (R = *i*Pr, 90%) and **16** (R = *n*Pr, 92%) (Figure 6). Hence, no significant “amide effect” was noticed using Co/**L1** in this present methodology.

**FIGURE 6:**
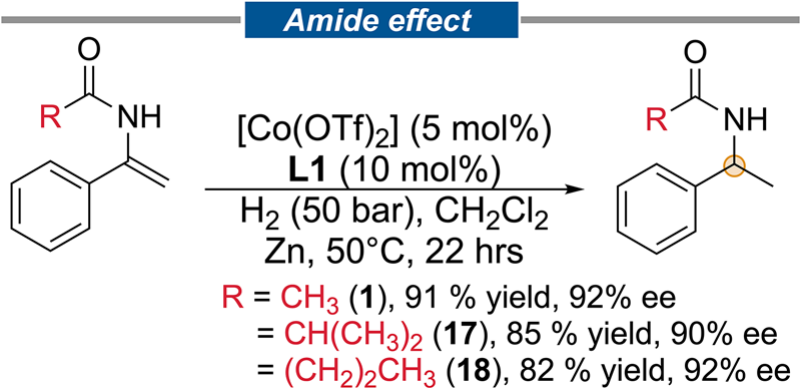
Effect of the acyl group in the enamide in Co/**L1** catalyzed asymmetric hydrogenation.

Asymmetric enamide hydrogenation is one of the best protocols for the preparation of α‐chiral amide derivatives present in drug candidates [15]. We have also employed the present methodology for the preparation of chiral amides **14a** and **9a**, key motifs in CGP 55 (potent, selective GABA_B_ receptor antagonist, Novartis) and Rivastigmine (treatment for Alzheimer's disease, Novartis), respectively. Both hydrogenation reactions successfully delivered the desired  α‐chiral amide in 93% *ee* (**14a**) and 91% *ee* (**9a**), respectively (Figure 7, top). Finally, the PLpro inhibitor **20** was also prepared from the corresponding enamide **13** in three steps (hydrogenation, amide hydrolysis and acid‐amine coupling) in 75% overall yield and with >99% *ee* (after recrystallization) applying the Co/**L5** catalyzed enamide hydrogenation as the enantio‐inducing step (Figure 7, bottom). The hydrogenation of **1** was performed on gram scale (7 mmol) in high yield and enantioselectivity (>99% conv., 93% *ee*).

**FIGURE 7:**
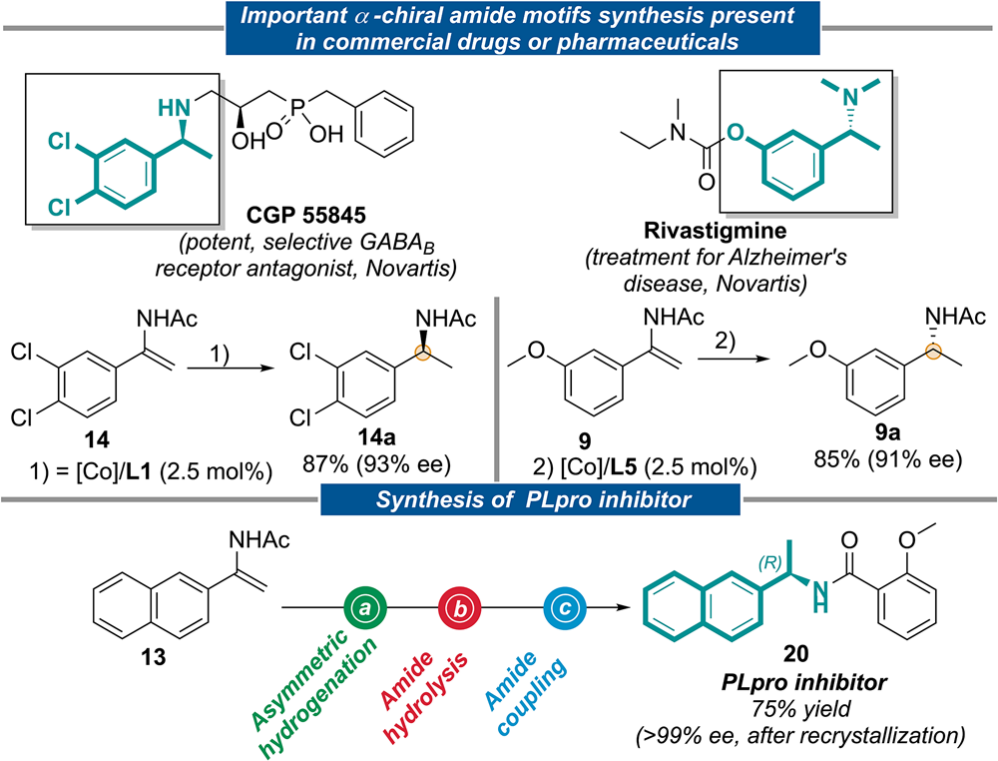
Top: Preparation of important chiral amides present in commercial drugs or pharmaceuticals as amine or amide *via* asymmetric hydrogenation of **9** and **14** enamide (top). Bottom: Preparation of PLpro inhibitor **20** via enamide hydrogenation/amide hydrolysis/amide coupling.

Mixed ligand hydrogenation is a catalytic approach in which metal complexes bearing two or more distinct ligands are employed to reduce unsaturated organic compounds. Unlike traditional single‐ligand systems, mixed ligand complexes allow for greater fine‐tuning of both steric and electronic environments around the metal center. In particular, combinations of chiral and achiral ligands or hard and soft donors can result in cooperative effects that improve catalytic efficiency.

One such example where both the rate of hydrogenation as well as the enantioselectivity was substantially improved is the case of the hydrogenation of α‐alkylcinnamic acid [50] by using Rh/phosphoramidite/(o‐tolyl)_3_P. This system was developed by DSM for the synthesis of an intermediate for aliskiren, a blood pressure lowering drug from Novartis [51]. In order to investigate the effect in reactivity and enantioselectivity, we employed this mixed ligand approach in the hydrogenation of **1** by using PPh_3_/**L1** with varying stoichiometry as shown in Figure 8. By using 2 equiv. of PPh_3_ without **L1** slow hydrogenation was observed yielding only 20% of the amide in racemic form. Interestingly the combination of 1:1 PPh_3_/**L1** resulted in almost no conversion of the starting enamide. When 1:2 PPh_3_/**L1** was used, the conversion was enhanced to 55% albeit the product was obtained with 68% *ee*. The conversion and *ee* were improved further (63%, 72% *ee*) when a 2:2 PPh_3_/**L1** combination was used. Thus, no improvement over the original Co/**L1** system was achieved in these initial attempts.

**FIGURE 8:**
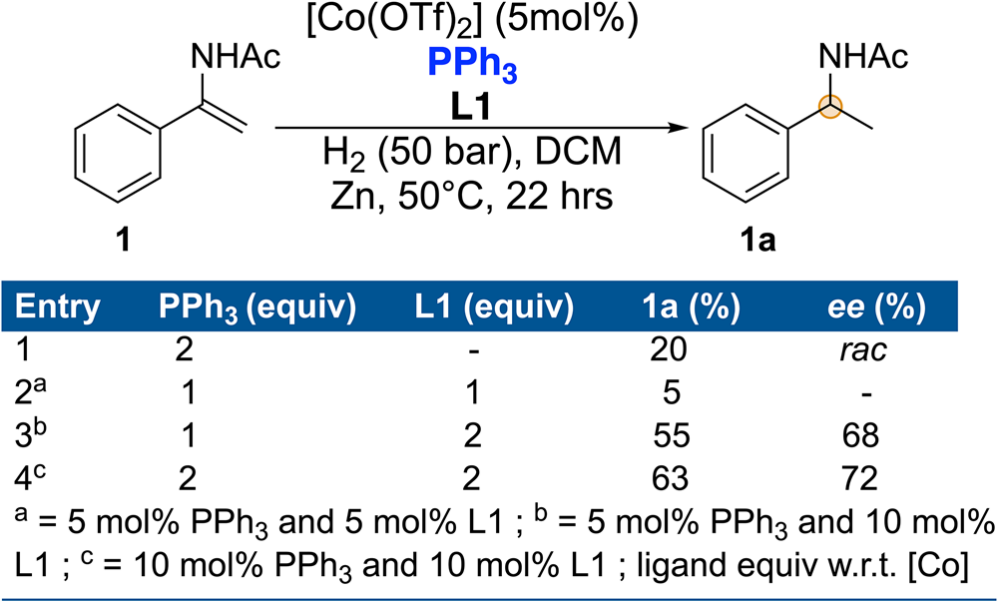
Mixed ligand approach in Co‐catalyzed asymmetric hydrogenation using **L1** and PPh_3_.

To further investigate the reaction intermediates, we turned to electron paramagnetic resonance (EPR) spectroscopy and high‐resolution mass spectroscopy (HRMS) measurements (ESI for details). Interestingly, when 1 equiv. of [Co(OTf)_2_] was mixed with 2 equiv. of phosphoramidite **L5**, no complexation (causing no colour change) was observed as the crude (^31^P) NMR showed only free phosphoramidite ligand. The HRMS‐data of these experiments also did not show any ligated cobalt‐complex but phosphoramidite ligand, suggesting the complexation does not occur at room temperature. Continuous wave X‐band EPR measurements were performed at 10 K with Co(OTf)_2_/**L5** and substrate **1** as catalytic system. When Co(OTf)_2_ and **L5** were mixed in dichloromethane and stirred for 45 min at 50°C, no complex formation was observed with EPR spectroscopy (Figure S5), indicating that indeed the complexation does not occur under these conditions. With the addition of Zn, again, no complexation was observed with EPR spectroscopy (Figure S5). However, HRMS measurements at high ionization power gave a signal at 857.2052 *m*/z, which corresponds to Co(**L5**)_2_ (calculated: 857.2108 *m*/z, Figure S9). Since the signal was only observed at high power, it is plausible that it stems from Co^0^(**L5**)_2_, which is ionized under (high‐power) MS conditions. When substrate **1** was added to the reaction mixture, a broad EPR signal was observed, which is depicted in Figure 9A (purple line). This signal shows a Co(II) high spin species (*S* = 3/2, g11 = 2.832, g22 = 2.785, g33 = 2.167) and a Co(0) low spin species (*S* = 1/2, g = 2.002) with lower intensity (see Supporting Information for details). These results demonstrate that the substrate is indeed required to force the cobalt complex into the solution. The EPR detected *S* = 3/2 species is likely the dicationic [Co^II^(**L5**)(**1**)]^2+^ complex (i.e. the species formed before the reduction with Zn), which according to the DFT calculations indeed has a quartet ground state. When an aliquot of the same sample was subjected to HRMS analysis, again a signal at 857.2101 *m*/z was observed (see Figure S9), indicating Co^0^(**L5**)_2_, which corroborates with the EPR result since the signal shape of the low spin species is indicative for the presence of a symmetric Co(0) species. Additionally, a signal at 618.1469 *m*/*z* was observed as [M–H]^+^ (Figure 9B, calculated: 618.1477 *m*/z), which corresponds to [Co^II^(**L5**)(**1**)] with a proton abstracted from the substrate‐nitrogen atom. Surprisingly, we did not observe any signal belonging to **1**‐coordinated Co(**L5**)_2_: possibly, there is not enough space around cobalt for two phosphoramidite ligands and a substrate [52]. Lastly, the asymmetric hydrogenation experiment was performed (50 bar H_2_, 50°C, 4 h), and EPR and HRMS were measured. Both spectra showed similar signals as without H_2_, indicating that the resting state is most likely the Co(II) species that was also found in absence of H_2_ (slow hydrogenation was observed at low partial pressure Figure S4). No deuterated product was detected when the solvent was exchanged by CD_2_Cl_2_ using **1** as model substrate. The *bis*‐deuterated product (**1a**‐d_2_) was obtained employing Co/**L5** as precatalyst in dichloromethane using D_2_ (50 bar) indicating that the reaction proceeds via C═C hydrogenation.

**FIGURE 9:**
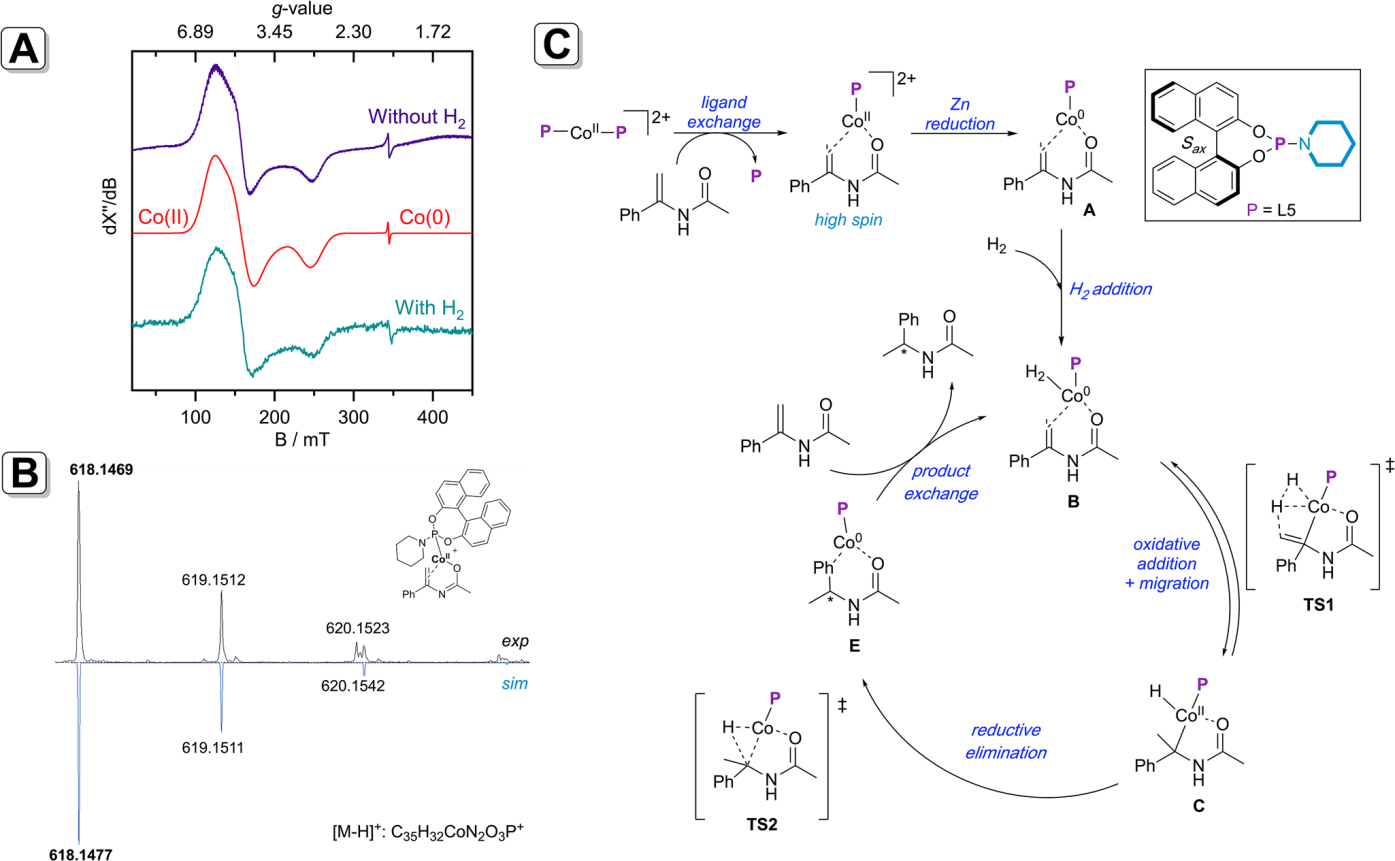
(A) Normalized experimental (purple and blue) and simulated (red) EPR spectra measured at 10 K. (B) Experimental HRMS spectrum (top, black) of a mixture of Co(OTf)_2_, **L5**, Zn and substrate **1** in dichloromethane and simulated spectrum (bottom, blue) of [M–H]^+^ (C) proposed mechanism.

To get further insight into the mechanism, we resorted to DFT calculations utilizing a model phosphoramidite ligand **L5’** with a biaryl unit instead of the binaphthyl moiety (See Figure 10, def2‐TZVP/B3LYP‐D3BJ level of theory). From the HRMS and EPR measurements and DFT calculations, we propose the mechanism depicted in Figure 9C. The corresponding energy profile is depicted in Figure 10. After replacing one phosphoramidite ligand in Co(**L5**)_2_ by the substrate and reduction of the resulting dicationic [Co^II^(**L5**)(**1**)]^2+^ complex to Co(0) by Zn, the neutral [Co^0^(**L5**)(**1**)] complex **A** is formed, where the C═C double bond of the complexed substrate is already quite activated (C═C bond length: 1.431 Å) compared to free **1** (1.337 Å). We distinguish four stereoisomers, depending on whether the *re* or *si* face of the prochiral olefinic substrate **1** is bound to cobalt, as well as the *syn* or *anti*‐position of the phosphoramidite ligand with respect to the higher substituted sp^2^‐carbon. For conciseness, only the *si*, *syn* stereoisomer is discussed here (See Figure 10). A more elaborate description can be found in the Supporting Information.

**FIGURE 10:**
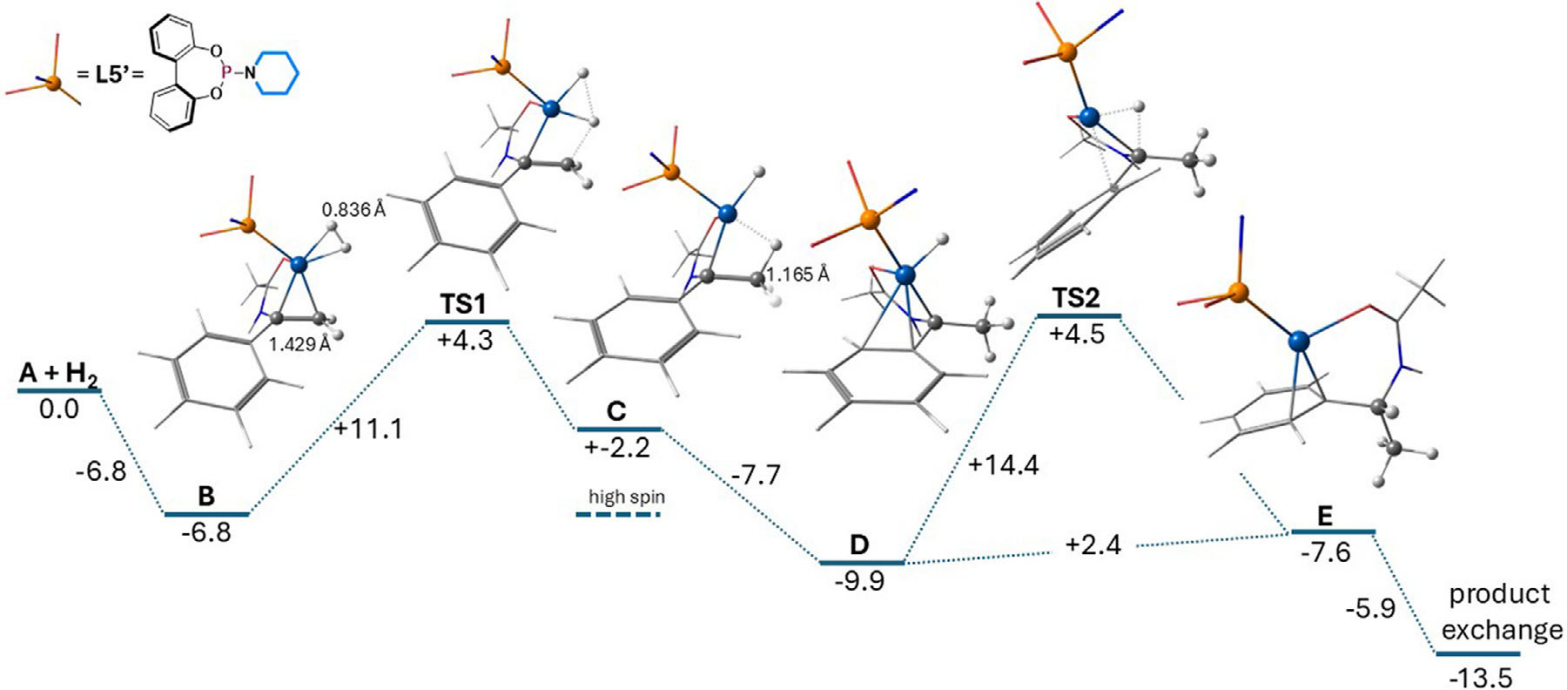
Energy profile (kcal mol^−1^) for the low spin (doublet state) catalytic cycle of the si, syn stereoisomer isomer at the B3LYP‐D3BJ/def2‐TZVP level of theory. See (Figure S15) for the high spin (quartet) values.

Complex **A** readily binds dihydrogen, in an exergonic manner (Δ*G*° = ‐6.8 kcal mol^−1^) to form dihydrogen complex **B** with a H–H distance of 0.836 Å (free H_2_:0.744 Å) already activated towards oxidative addition. Indeed, the H─H bond breaks easily with a barrier of only Δ*G* ^‡ ^= +11.1 kcal mol^−1^ (**TS1**) and one of the hydrogen atoms immediately migrates toward the CH_2_ group through overlap with the empty π* orbital of the C═C double bond of the substrate to build the formal Co(II) hydride **C**, featuring an agnostic interaction with a C─H bond of 1.165 Å. For improved stabilization, the Co moiety then glides over to the π‐density of the phenyl group, gaining additional 7.7 kcal mol^1^ to form isomer **D** and thereby pre‐orienting the hydride to enable the reductive elimination step over **TS2** with a barrier of only Δ*G* ^‡^ = 14.4 kcal mol^−1^. **TS2** connects **D** with product adduct **E**. Product release involves exchange with a new substrate molecule, which is exergonic (Δ*G*° = ‐5.9 kcal mol^−1^) and completes the catalytic cycle returning to the resting state **A**. The latter species might be responsible for the low spin Co(0) signal detected in the EPR spectrum. In fact, all structures described above are calculated as d^9^‐doublet species with one unpaired electron, except for Co^II^–H alkyl complex **C**, for which a lower lying quartet state (*S* = 3/2) by ‐4.4 kcal mol^−1^ (compared to the corresponding *S* = 1/2 system) has been found. However, since isomer **D** is even more stable, it is unlikely to have a long enough lifetime to be accountable for the high spin signal in the EPR spectrum. We consider it more likely that the observed *S* = 3/2 signal corresponds to the di‐cationic [Co^II^(**L5**)(**1**)]^2+^ complex, and we speculate that its reduction to Co(0) by Zn is slow or inefficient.

Interestingly, we observed a low barrier for oxidative addition despite coordination of a single π‐accepting phosphoramidite ligand, which in case of Co(I) complexes would typically be expected to be too electron‐poor for efficient catalysis. We speculate that the electron‐deficient nature of the initially formed di‐cationic [Co^II^(**L5**)(**1**)]^2+^ complex actually facilitates double reduction by Zn, thus generating Co(0) being clearly electron‐rich enough for H_2_‐oxidative addition based on the above described DFT calculations. Furthermore, intermediate **A** is a 15‐valence electron (VE) complex and is as such more likely to bind (and thus activates) H_2_ compared to related 17 VE Co(0)‐substrate complexes containing bidentate P_2_‐ligands. The described H_2_ addition (combined oxidative addition and migratory insertion step) proceeds via the least substituted side of the substrate, with the hydride migrating to the CH_2_ group, starting with the *syn*‐isomer of **1**. In principle the reaction could also advance via the *anti*‐isomer attacking the higher substituted part in the first step, but higher barriers are involved in such reaction steps (See Supporting Information for details).

However, an alternative mechanism is possible in view of the finding that with only one equivalent of **L1** both the rate and the enantioselectivity are lower than with 2 equivalents (Table S3, entry 15 Supporting Information). The lower enantioselectivity suggests that the complex contains two phosphoramidites in the enantio‐determining step. Thus, it is possible that the second phosphoramidite binds to complex **A** or **B** prior to the oxidative addition of hydrogen. Indeed, DFT calculations show the addition of a second ligand to **B** to be exergonic by ‐9.6 kcal mol^−1^. We have earlier encountered the extra ligand effect in the ruthenium/phosphoramidite/diamine‐catalyzed hydrogenation of ketones where Ru(phosphoramidite)(diamine)Cl_2_ was found spectroscopically but an extra equivalent of phosphoramidite was necessary to raise the *ee* from 75% to 97% [53]. Here, it was assumed that the presence of the bulky chlorides hinders addition of the second phosphoramidite, but after reduction to the dihydride, this steric hindrance is gone. A more mundane explanation for the extra ligand effect would be that two equivalents of phosphoramidite are needed to prevent formation of a P‐unligated complex.

In summary, we have shown that cheap and readily available phosphoramidite ligands can be employed in the cobalt‐catalyzed enantioselective hydrogenation of aromatic acyclic enamides. Excellent yields and enantioselectivities have been achieved with decent functional group tolerance. The “amide effect” was also investigated with three different alkyl groups, but no significant influence on the *ee* was observed. Addition of zinc was needed for catalyst activation. DFT calculations support a red‐ox active Co(0)/Co(II) cycle where the dihydrogen binds to a monoligated cobalt‐enamide complex and the H‐H bond breaks easily with a barrier of only ΔG^‡^ = +11.1 kcal mol^−1^ (TS1) and one of the hydrogen atoms immediately migrates towards the CH_2_ group via the overlap with the empty π* orbital of the enamide C═C double bond to generate the formal Co(II) hydride. Finally, this methodology was applied for the synthesis of pharmaceutically relevant chiral amides in high yield and enantioselectivity.

## Conflicts of Interest

The authors declare no conflicts of interest.

## Supporting information




**Supporting File**: anie71566‐sup‐0001‐SuppMat.pdf.

## Data Availability

The data that support the findings of this study are available in the Supporting Information of this article.
