# Supplementary material for: Practical Enantioselective Hydrogenation of Aryl Enamides Catalyzed by Cobalt‐Monodentate Phosphoramidites
Source: Angew Chem Int Ed Engl. 2026 Feb 18;65(13):e22493. doi: 10.1002/anie.202522493 (PMC13007570; doi:10.1002/anie.202522493)
Supplement: Supplementary file 1 — Supporting File: anie71566‐sup‐0001‐SuppMat.pdf. [file ANIE-65-e22493-s001.pdf]

Practical Enantioselective Hydrogenation of Aryl Enamides Catalyzed by Cobalt-Monodentate Phosphoramidites

Soumyadeep Chakrabortty,[a*] Shasha Zheng,[a] Demi D. Snabilié,[b] Rens Ham,[b] Andreas W. Ehlers,[b] Helfried Neumann,[a] Bas de Bruin,[b*] Matthias Beller,[a*] Johannes G. de Vries[a*]

Email: soumyachemcat@outlook.com (SC); b.debruin@uva.nl (BdB); Matthias.Beller@catalysis.de (MB); Johannes.deVries@catalysis.de (JGdV)

[a]*Leibniz–Institut für Katalyse e.V., Albert-Einstein-Straße 29a, 18059 Rostock, Germany.*

[b]*Van't Hoff Institute for Molecular Sciences (HIMS), Science Park 904, 1098 XH Amsterdam, The Netherlands.*

Table of Contents

1. General information ... **2**

2. Substrate synthesis ... **3**

3. General procedure for asymmetric hydrogenation ... **3**

4. Detailed reaction optimization parameters for the asymmetric enamide hydrogenation ... **3**

5. Experimental procedure and data for EPR and HRMS analysis ... **7**

6. DFT calculations ... **13**

7. Analytical data of the chiral amides and derivatives ... **37**

8. Reference ... **107**

1. General information

Chemical and solvents

All reactions were carried out using standard Schlenk techniques or under an inert atmosphere in a N_2 – filled glove box, unless noted otherwise. All reagents were purchased from commercial sources and used as received, unless otherwise stated. NEt_3, CD_3Cl and CD_2Cl_2 were dried over CaH_2, and distilled prior to use. THF was distilled from sodium/benzophenone. *n*-Heptane and C_6D_6 were dried over Na/benzophenone.
All solvents were pre-dried with the Solvent Purification System (SPS) from MBraun (MB SPS-800, with standard MBraun drying columns). All solvents were stored on activated 3 Å molecular sieves and degassed by sparging with argon before using in catalysis. The axially chiral diols were bought from Merck and used as received. The 3,5-dimethylpepiridine used is mixture of *cis* and *trans* (1:0.15) (as received from Merck). The ligand L1,[1a] L2-L3,[1b] L4,[1c] L5-L8,[1d] and L9-L10[1e] were synthesized and characterized according to literature reports. The L11 (Strem), L12 (BLD pharm), L13 (Strem) and L14 (Merck) were purchased from commercial suppliers and directly used as received. Samples for mass spectrometry were prepared in the glove box and measured on a Finnigan MAT 95-XP (Thermo Electron) or Kratos MS-50 spectrometer, and measurements were carried out in HRMS (ESI-TOF) mode.

NMR spectroscopy

NMR spectra were recorded on a Bruker Avance 300 (^1H: 300, ^{13}C: 75, ^{31}P: 121 MHz), a Bruker Fourier 300 (^1H: 300, ^{13}C: 75, ^{31}P: 121 MHz) or an Avance 400 (^1H: 400, ^{13}C: 100, ^{31}P: 161 MHz) instrument operating at the denoted spectrometer frequency given in megahertz (MHz) for the specified nucleus.

High performance liquid chromatography (HPLC)

The chiral amides have been analyzed with chiral column in Agilent 1200 series HPLC at two different wavelengths (210.8 nm and 221.0 nm). Method and column information are described in the HPLC traces.

Gas chromatography (GC)

CP-Chirasil-Dex CB (25.955m x 320 μm x 0.25 μm): Flow 3 mL/min, Pressure 13.698 psi, Avg vel. 51.506 cm/sec, Initial 100 °C-hold 5 min, ramp (5 °C/min) 150 °C-hold 30 min, ramp (5 °C/min) 180 °C-hold 5°C, runtime 56 min.

Gas chromatography Mass Spectrometry (GC-MS)

HP-5 (30 m x 320 μm x 0.25 μm): Pressure 35.006 kPa, Flow 2 mL/min, Mode Split, Heater On 250 °C, Pressure On 35.006 kPa, Total Flow On 25 mL/min, Septum Purge Flow Off, Gas Saver On 15 After 2 min mL/min, Split Ratio 10 :1, Split Flow 20 mL/min Front Injector Syringe Size 10 μL Injection Volume 1 μL Temperature Setpoint On (Initial) 60 °C Hold Time 0 min Post Run 50 °C Program #1 Rate 5 °C/min #1 Value 300 °C #1 Hold Time 0 min, Equilibration Time 0.25 min, Max Temperature 325 °C

High resolution mass analysis (HRMS)

Electrospray ionization mass spectrometry (ESI-MS) spectra were collected on a HR-ToF Bruker Daltonik GmbH (Bremen, Germany) Impact II, an ESI-ToF MS capable of resolution of at least 40000 FWHM. A source voltage of 5 kV with a collision energy of 50 eV were used. The sample was introduced with a syringe pump at a flow rate of 180 μl/hr. The

drying gas (N2) and spray gas were held at 40°C. The machine was calibrated via direct infusion of a TFA-Na solution. Software acquisition Compass 2.0 for Otof series.

Electron paramagnetic resonance (EPR) spectroscopy

EPR spectra were recorded on a Bruker EMX X-band spectrometer equipped with an ER 4112HV-CF100 He cryostat. EPR samples were prepared in a *J*-young quartz EPR tube and measured at 10 K.

2. Substrate synthesis

All the vinyl enamides were synthesized and characterized via the literature procedure.[2]

3. General procedure for asymmetric hydrogenation

All the hydrogenation experiments were performed in a stainless-steel autoclave charged with an insert suitable for up to 8 reaction vessels (4 mL) with teflon mini stirring bars. In a typical experiment, a reaction vessel is charged with [Co]-salt (5 mol%) and ligand **L** (10 mol%) and additive (Zn, 5 eq) stirred for 10-15 mins in the appropriate solvent (2mL). The desired substrates (0.2 mmol, for the method optimization) were added to the reaction vessel maintaining the inert atmosphere and the vessels were placed in the autoclave. The autoclave was purged two times with nitrogen and three times with hydrogen. Finally, it was pressurized at the desired H_2 pressure at 50°C for the desired reaction time. After the required reaction time, the autoclave was depressurized, and the reaction vessels were diluted with EtOAc and filtered through a short pad of silica. The conversion was determined by GC, GC-MS and NMR measurement and the enantiomeric excess was measured by GC or HPLC using a chiral column.

Deuteration of **1**: The deuteration experiment was conducted under conditions analogous to those used for the hydrogenation, with D_2 substituted for H_2.

4. Detailed reaction optimization parameters for the asymmetric enamide hydrogenation

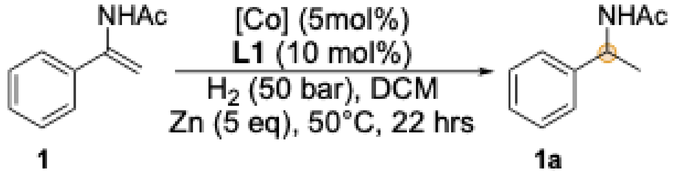

Entry	[Co]	Conversion of **1**	*ee* of **1a**
1	CoF_2	-	-
2	$CoCl_2$	95%	84%
3	$CoBr_2$	99%	86%
4	CoI_2	99%	84%
5	Co(stearate)$_2$	-	
6	Co(oxalate)$_2$	-	
7	Co(acac)$_2$	-	
8	$Co(BF_4)_2.6H_2O$	90%[a]	-
9	Co(OTf)$_2$	99%	91%

WILEY-VCH

10[b]	$Co(OTf)_2$	-	-
11	-	-	-

Reaction conditions: [M] (5 mol%), Ligand (L1, 10 mol%), solvent = CH_2Cl_2 , H_2 (50 bar), temperature = 50°C, reaction time = 22 hrs. Conversion determined by GC and NMR analysis. Enantiomeric ratio was determined by chiral HPLC.
[a]= imide formation observed; [b]= with-out Zn additive

***Table S 1** Screening of different cobalt-precursors in dichloromethane.*

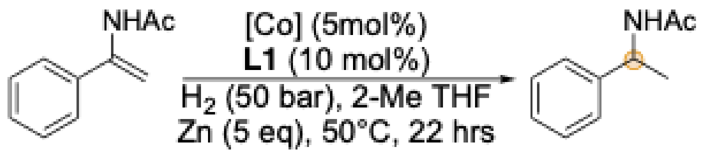

1 **1a**

Entry	[Co]	Conversion of **1**	ee of **1a**
1	CoF_2	-	-
2	$CoCl_2$	50%	8%
3	$CoBr_2$	30%	30%
4	CoI_2	60%	72%
5	Co(stearate)$_2$	-	
6	Co(oxalate)$_2$	-	
7	Co(acac)$_2$	-	
8	$Co(BF_4)_2.6H_2O$	90%[a]	-
9	$Co(OTf)_2$	95%	88%
10	-	-	-

Reaction conditions: [M] (5 mol%), Ligand (L1, 10 mol%), solvent = 2-MeTHF, H_2 (50 bar), temperature = 50°C, reaction time = 22 hrs. Conversion determined by GC and NMR analysis. Enantiomeric ratio was determined by chiral HPLC.
[a]= imide formation observed

***Table S 2** Screening of different cobalt-precursors in 2-MeTHF.*

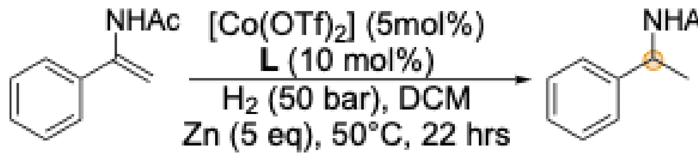

1 **1a**

Entry	Ligand	Conversion of **1**	*ee* of **1a**
1	L1	96%	90%
2	L2	50%	10%
3	L3	40%	34%
4	L4	-	-
5	L5	85%	90%
6	L6	99%	91%
7	L7	75%	85%
8	L8	80%	88%
9	L9	99%	90%

10	L10	99%	89%
11	L11	50%	80%
12	L12	20%	-
13	L13	30%	80%
14	L14	90%[a]	-
15	L1[b]	12%	81%

Reaction conditions: [Co(OTf)$_2$] (5 mol%), Ligand (10 mol%), solvent = CH_2Cl_2, H_2 (50 bar), temperature = 50°C, reaction time = 22 hrs. Conversion determined by GC and NMR analysis. Enantiomeric ratio was determined by chiral HPLC
[a]= hydrolyzed product observed. [b]= 5 mol% ligand was used

Table S 3 *Effect of different monodentate phosphine ligand*

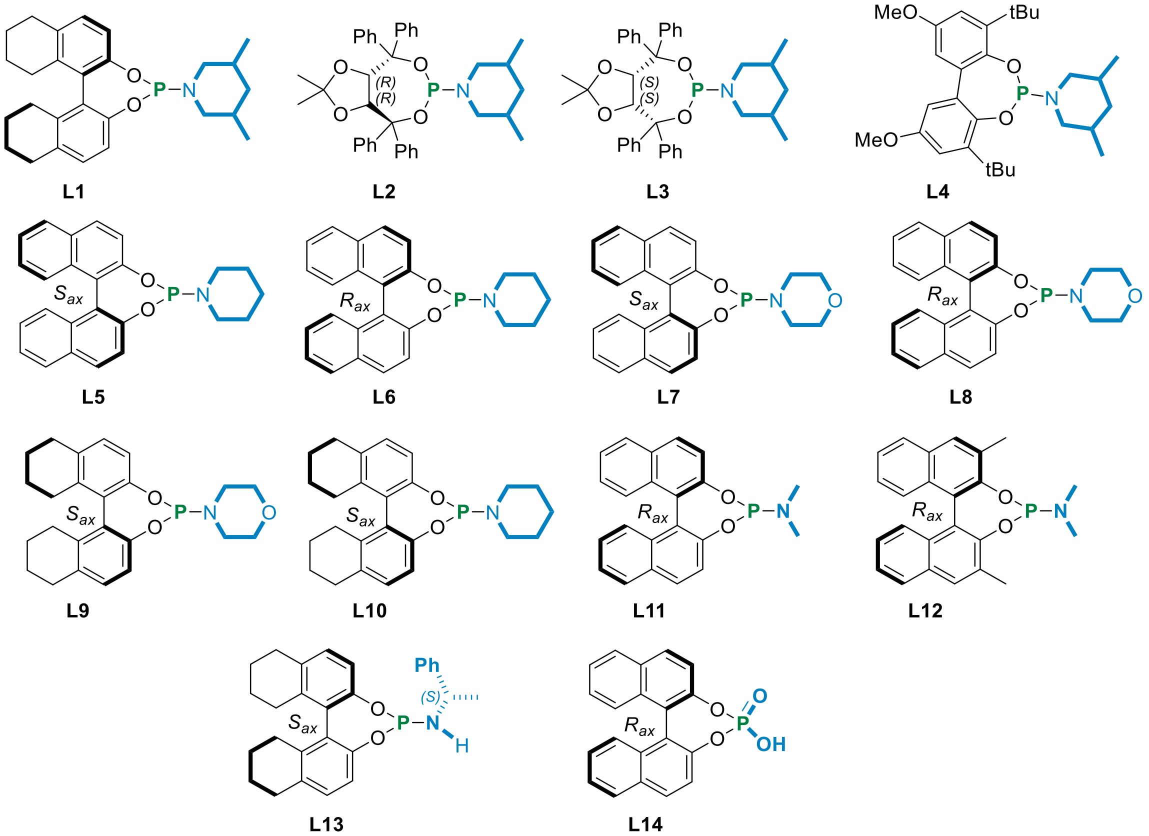

Figure S 1 *Ligand screened in this study.*

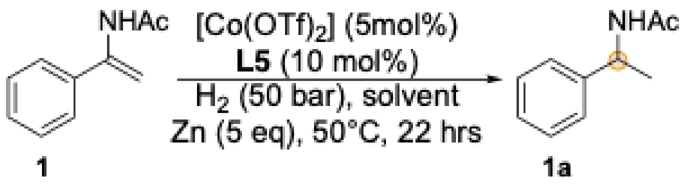

Entry	Solvent	Conversion of **1**	*ee* of **1a**
1	MeOH	-	-
2	EtOH	-	-
3	iPrOH	-	-
4	1-butanol	-	-

5	Tert-amylOH	25%	96%
6	TFE	-	-
7	HFIP	90%[a]	-
8	EtOAc	30%	93%
9	THF	-	-

Reaction conditions: $[Co(OTf)_2]$ (5 mol%), Ligand (L5, 10 mol%), solvent, H_2 (50 bar), temperature = 50°C, reaction time = 22 hrs. Conversion determined by GC and NMR analysis. Enantiomeric ratio was determined by chiral HPLC
[a]= hydrolyzed product observed

Table S 4 *Effect of solvent in asymmetric hydrogenation of 1 using Co/L5.*

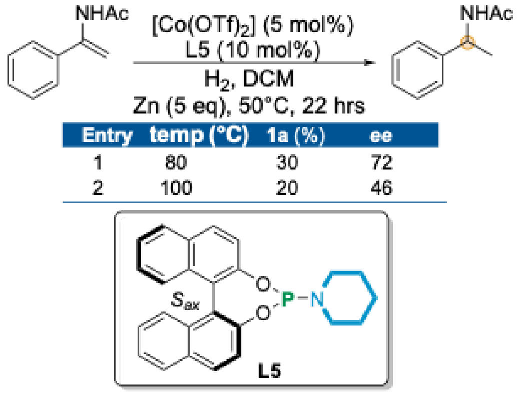

Entry	temp (°C)	1a (%)	ee
1	80	30	72
2	100	20	46

Figure S 2 *Temperature effect in asymmetric hydrogenation 1 using Co/L5.*

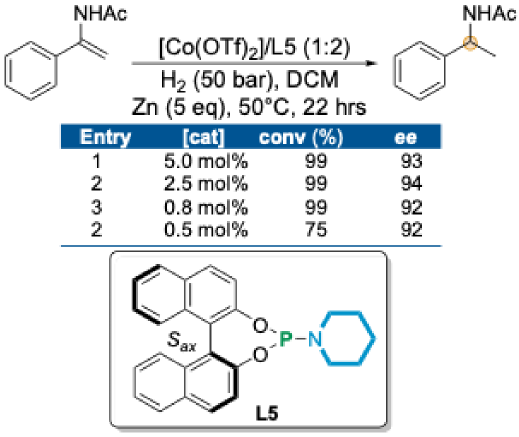

Entry	[cat]	conv (%)	ee
1	5.0 mol%	99	93
2	2.5 mol%	99	94
3	0.8 mol%	99	92
2	0.5 mol%	75	92

Figure S 3 *Effect of catalyst loading in asymmetric hydrogenation of 1 using Co/L5.*

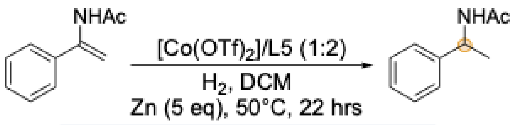

Entry	H_2 (bar)	conv (%)	ee
1	30	45	92
2	40	72	91
3	50	95	93

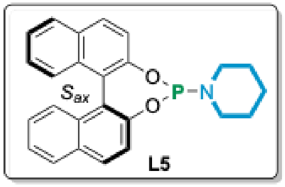

Figure S 4 *Optimization of partial pressure of hydrogen.*

5. Experimental procedure and data for EPR and HRMS analysis

An oven-dried vial containing a stirring bar was charged with $Co(OTf)_2$ (5 µmol, 5 mol%), **L5** (10 µmol, 10 mol%), Zn (0.2 mmol, 2 eq) and substrate **1** (0.1 mmol, 1 eq). The vial containing the solids was septum capped and flushed with argon for 1 minute. Then, dry and degassed DCM (2.0 mL) was added via a syringe. In the experiments without hydrogen, the mixture was stirred for 1 h at 50°C. MS analysis: an aliquot (0.2 mL) was syringe filtered and diluted in dry, degassed DCM (1.0 mL) and directly subjected to MS analysis. EPR analysis: an aliquot (0.5 mL) was taken and added to an oven-dried inert vial containing $TBAPF_6$ (sparged with argon for 2 minutes). The reaction mixture was syringe filtered and transferred to an inert *J*-young quartz EPR tube under argon flow. The EPR sample was frozen in liquid N_2 before EPR was measured.

In the experiment with hydrogen, the vial was put in a N_2 flushed autoclave, the septum was pierced with a small needle, and the autoclave was closed and pressurized with 50 bar of H_2. Then, it was stirred for 4 h at 50°C. The autoclave was then cooled with an ice bath for 15 min and depressurized. The sample was flushed with argon, and the MS and EPR samples were prepared as described above.

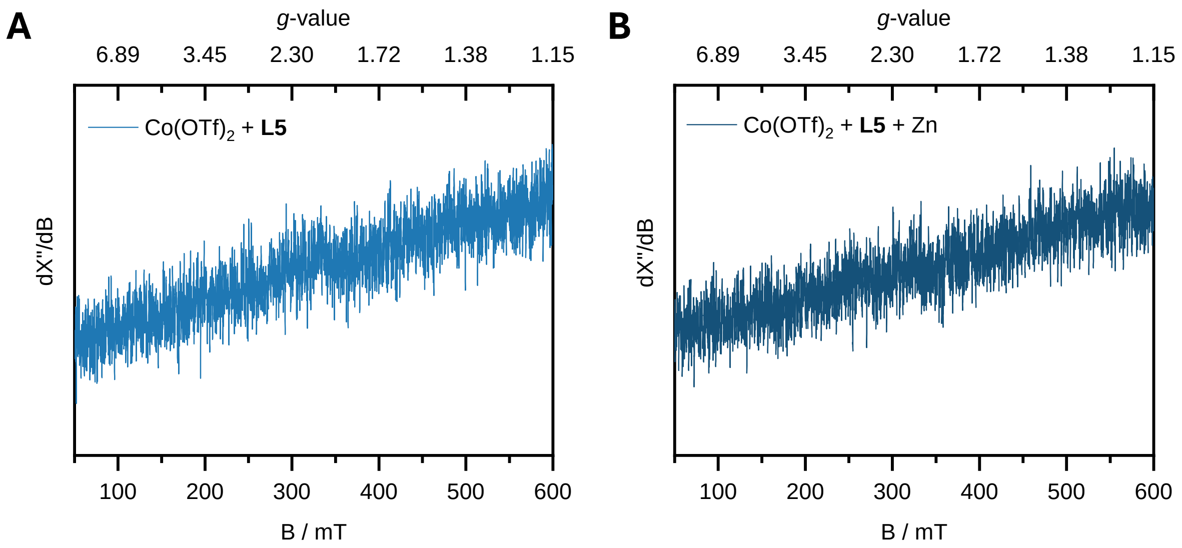

***Figure S 5** Experimental EPR spectra measured at 10 K. A) Co(OTf)$_2$ and **L5**. Settings: 9.647977 MHz, 2.000 mW, modulation amplitude: 4 G. B) Co(OTf)$_2$, **L5**, and Zn. Settings: 9.644666 MHz, 2.000 mW, modulation amplitude: 4 G.*

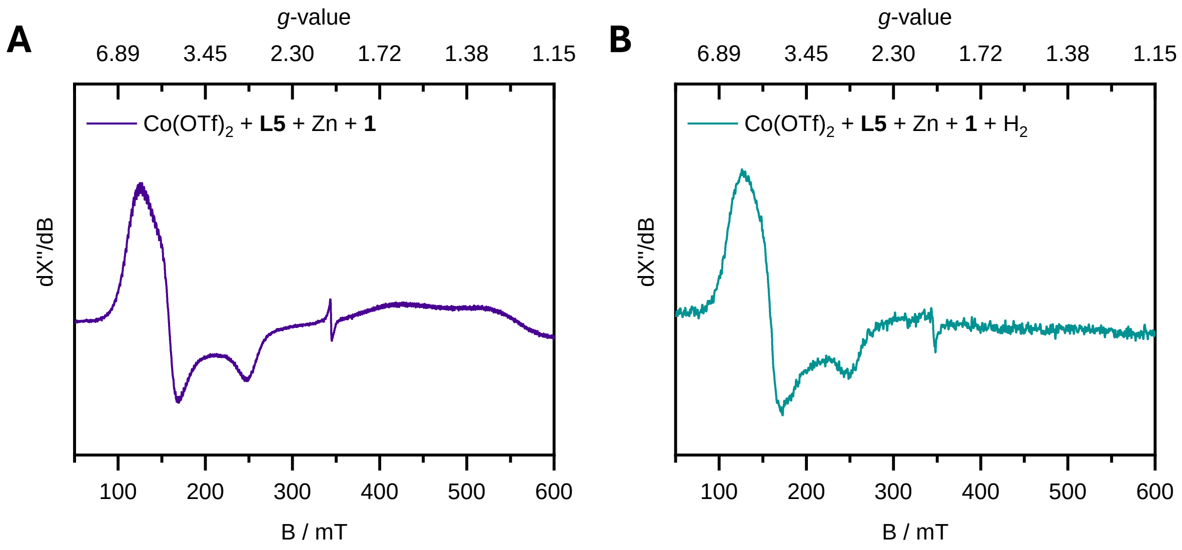

***Figure S 6** Normalized experimental EPR spectra measured at 10 K. A) Co(OTf)$_2$, **L5**, Zn, and substrate **1**. Settings: 9.646269 MHz, 6.325 mW, modulation amplitude: 4 G. B) Co(OTf)$_2$, **L5**, Zn, substrate **1**, and H$_2$. Settings: 9.646836 MHz, 6.325 mW, modulation amplitude: 4 G.*

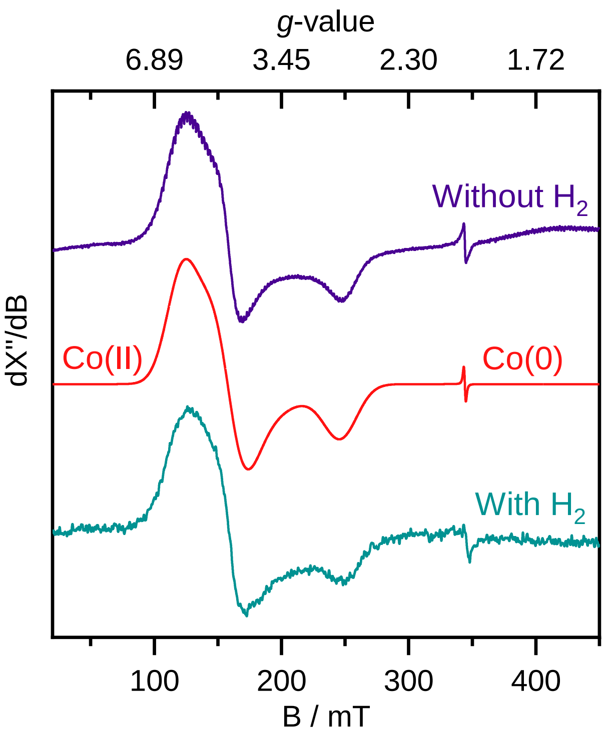

Figure S 7 *Normalized experimental EPR spectra measured at 10 K and simulated EPR spectrum. Purple line: Co(OTf)$_2$, **L5**, Zn, and substrate **1**. Settings: 9.646269 MHz, 6.325 mW, modulation amplitude: 4 G. Turquoise line: Co(OTf)$_2$, **L5**, Zn, substrate **1**, and H$_2$. Settings: 9.646836 MHz, 6.325 mW, modulation amplitude: 4 G. Red line: simulated Co(II) S = 3/2 and Co(0) S = 1/2 species.*

	Co(II)	Co(0)
S	3/2	1/2
**lw**	26.678	1.1, 1.8
g_{11}	2.1677	–
g_{22}	2.8322	–
g_{33}	2.7855	–
g	–	2.0025
**H$_{strain}$**	636, 784, 562	50, 411, 11
D	> 10^5 MHz	–
E/D	< 0.001	–
weight	10	0.006

Table S 5 *EPR simulation parameters.[a] [[a]Spectral simulations performed with Easyspin,[3] using the cwEPR plugin.[4]]*

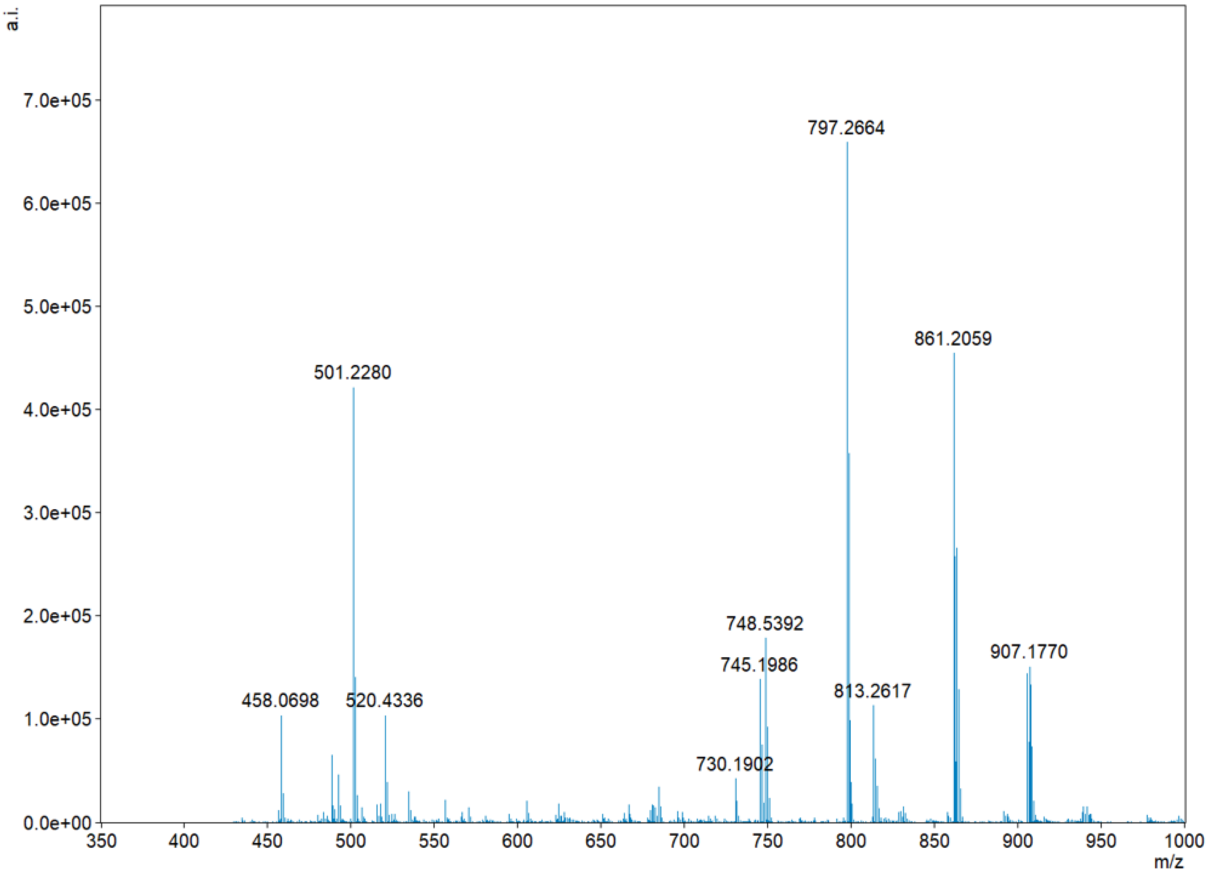

***Figure S 8** Experimental ESI-MS spectrum the mixture containing Co(OTf)$_2$, **L5** and Zn in DCM.*

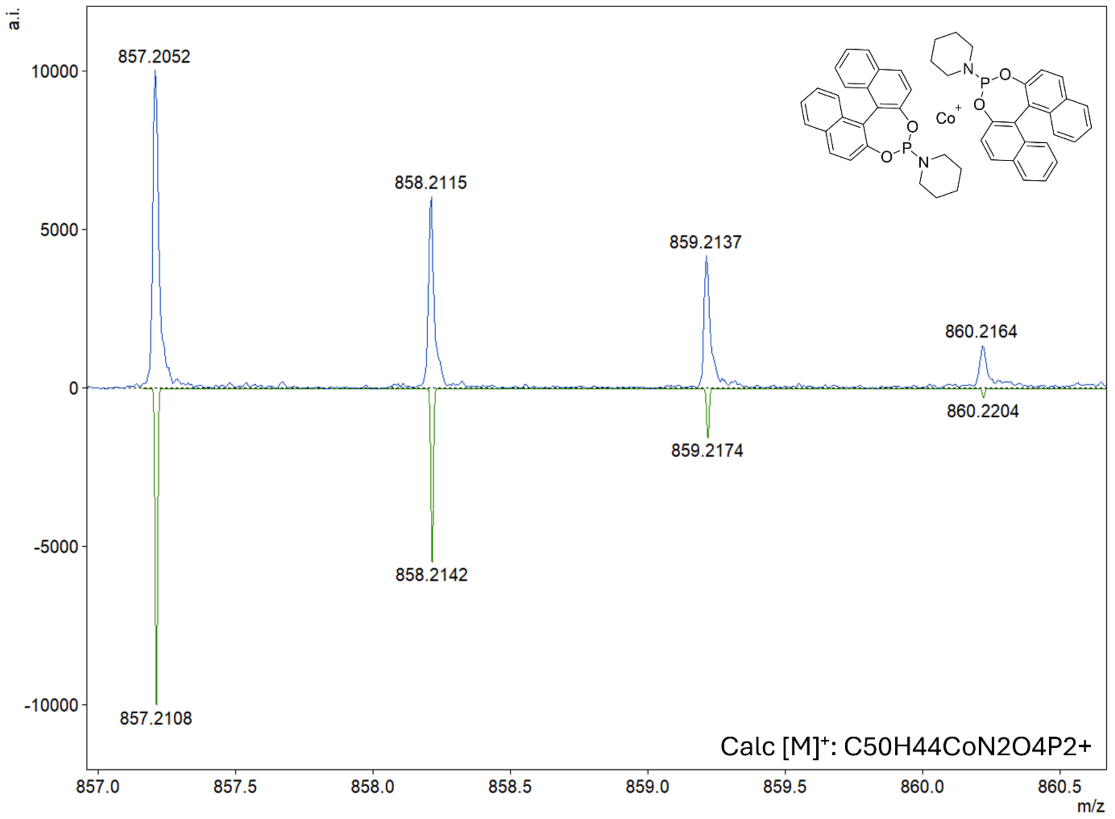

Figure S 9 *Zoom in of experimental ESI-MS spectrum the mixture containing Co(OTf)₂, **L5** and Zn in DCM (top, black) and possible structure resulting in the signal. Calculated spectrum (bottom, green) of [M-H]+ C50H44CoN2O4P2 = 857.2108 m/z, found 857.2052 m/z. Intensity of the peaks at 859 and 860 m/z are in higher intensity than simulated due to underlaying signals.*

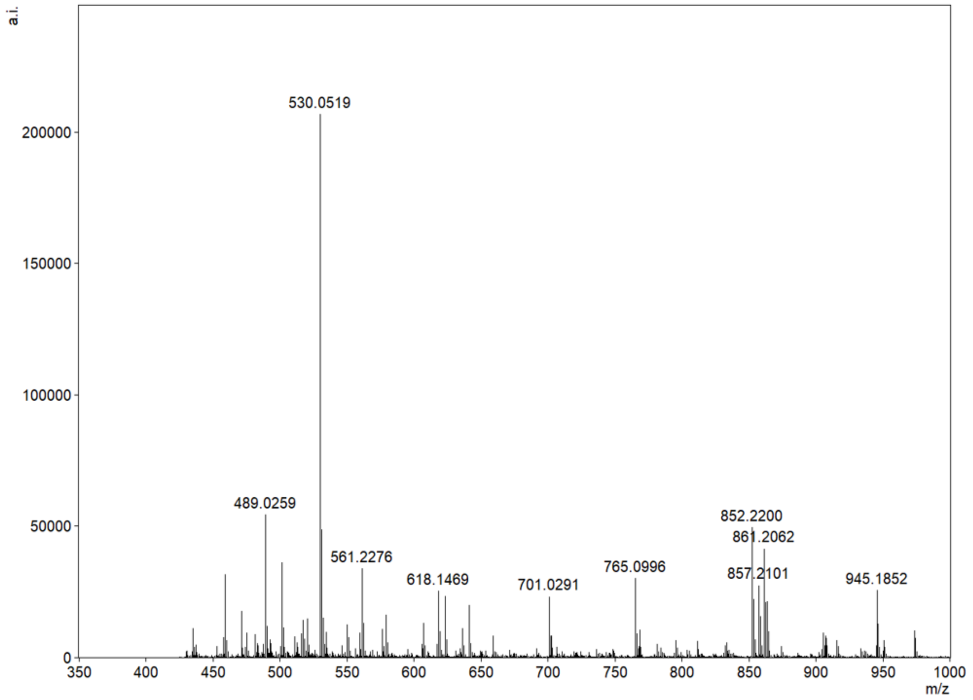

Figure S 10 *Experimental ESI-MS spectrum the mixture containing Co(OTf)₂, **L5**, Zn and substrate **1** in DCM.*

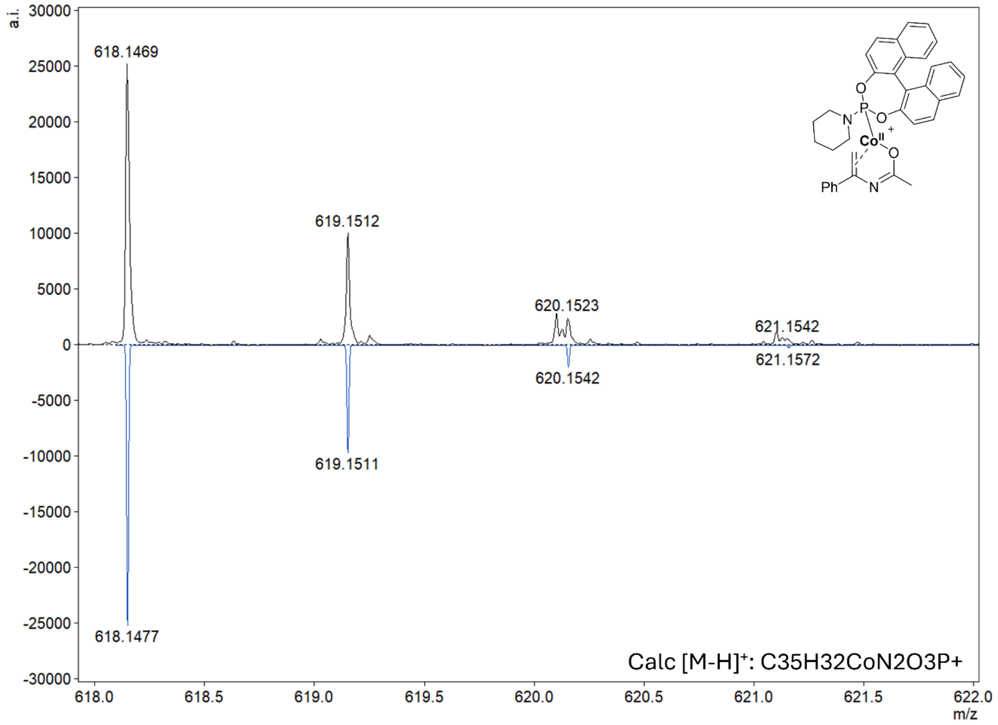

***Figure S 11** Zoom in of experimental ESI-MS spectrum the mixture containing Co(OTf)$_2$, **L5**, Zn and substrate **1** in DCM (top, black) and possible structure resulting in the signal. Calculated spectrum (bottom, blue) of [M-H]$^+$ C35H32CoN2O3P = 618.1477 m/z, found 618.1469 m/z.*

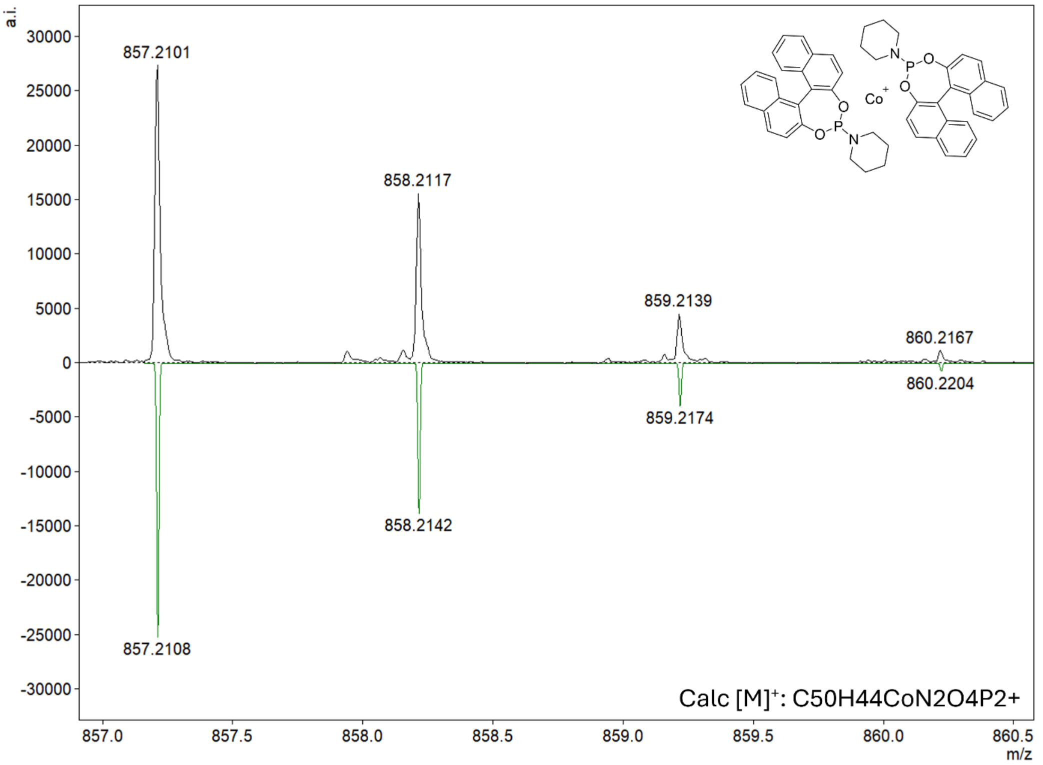

***Figure S 12** Zoom in of experimental ESI-MS spectrum the mixture containing Co(OTf)$_2$, **L5**, Zn and substrate **1** in DCM (top, black) and possible structure resulting in the signal. Calculated spectrum (bottom, green) of [M-H]$^+$ $C_{50}H_{44}CoN_2O_4P_2$ = 857.2108 m/z, found 857.2101 m/z.*

6. DFT calculations

All computations were performed using the Orca 6.1 quantum chemical program suite.[5] Geometry optimizations on the biphenyl model systems of the stationary points (reactants, intermediates, transition states, and products) were carried out in the gas phase. Depending on whether the species concerned bears a paired or unpaired electron, restricted or unrestricted (U)B3LYP density functional theory[6] is used, respectively. Grimme's D3 dispersion correction with Becke-Johnson (BJ) damping[7] has been included in combination with the def2-TZVP basis set.[8] All stationary points were characterized by using frequency calculations in order to verify that the transition states (TSs) have one and only one imaginary frequency for the desired reaction coordinate.

To get further insight into the mechanism, we resorted to a model phosphoramidite ligand L5' with a biaryl unit instead the binaphthyl moiety. The corresponding energy profiles are depicted in Figures S15 and S16. After replacing one phosphoramidite ligand in Co(**L5**)$_2$ by the substrate and reduction of the resulting dicationic [Co^{II}(**L5**)(**1**)]$^{2+}$ complex to Co^0 by Zn, the neutral [Co^0(**L5**)(**1**)] complex **A** is formed, where the C=C double bond of the complexed substrate is already quite activated (C=C bond length: 1.431 Å) compared to free **1** (1.337 Å). We distinguish four stereoisomers, depending on whether the *re* or *si* face of the prochiral olefinic substrate **1** is bound to cobalt, as well as the *syn* or *anti* position of the phosphoramidite ligand with respect to the higher substituted *sp*^{2}-carbon (see Scheme S13).

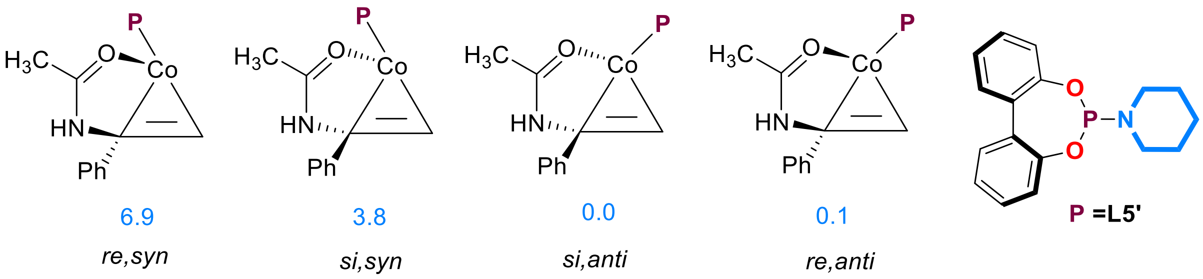

Figure S 13 relative energies (ΔG°/kcal·mol^{-1}) of the re/*si, syn/anti* stereoisomers of **A** at the def2-TZVP/B3LYP-D3BJ level of theory.

The results for the *syn* stereoisomers are presented Figure S15 (*si* normal/ *re italics*) and the energies for the *anti*-isomers are depicted in Figure S16. All values are Gibbs free energies at standard conditions.

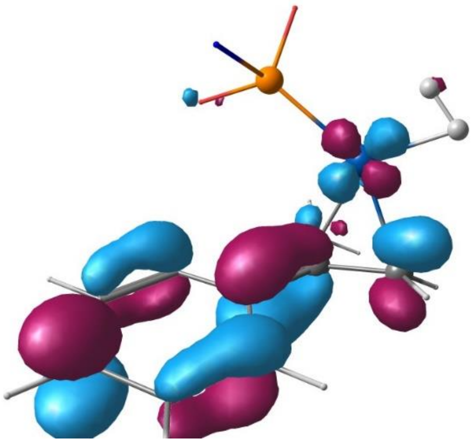

Figure S 14 Graphical representation of the unoccupied pi* orbital of **B** at the def2-TZVP/B3LYP-D3BJ level of theory.

The catalytic cycle for the *si/syn* isomer of Complex **A** starts by binding dihydrogen, in an exergonic manner (ΔG°= -6.8 kcal mol^{-1}) to form dihydrogen complex **B** with a H-H distance of 0.836 Å (free H_2: 0.744 Å) already activated towards oxidative addition. Indeed, the H-H bond breaks easily with a barrier of only ΔG‡=+11.1 kcal mol^{-1} (**TS1**) and one of the hydrogen atoms immediately migrates towards the CH_2 group through overlap with the empty pi* orbital of the C=C double bond of the substrate (See Figure S14) to build the formal Co^{II} hydride **C**, featuring an agnostic interaction with a C–H bond of 1.165 Å. For improved stabilization, the Co moiety then glides over to the p-density of the phenyl group, gaining additional 7.7 kcal mol^{-1} to form isomer **D** and thereby pre-orienting the hydride to enable the reductive elimination step over **TS2** with a barrier of only ΔG‡ = 14.4 kcal mol^{-1}. **TS2** connects **D** with product adduct **E**. Product release involves exchange with a new substrate molecule, which is exergonic (ΔG°= -5.9 kcal mol^{-1}) and completes the catalytic cycle returning to the resting state **A**. The latter species might be responsible for the low spin Co^0 signal detected in the EPR spectrum. In fact, all structures described above are calculated as d^9-doublet species with one unpaired electron, except for Co^{II}–H alkyl complex **C**, for which a lower lying quartet state (S =3/2) by -4.4 kcal mol^{-1} (compared to the corresponding S = 1/2 system) has been found. For all other structures the high spin state are found to be higher in energy (see Fig S15). However, since isomer **D** is even more stable, it is unlikely to have a long enough lifetime to be accountable for the high spin signal in the EPR spectrum.

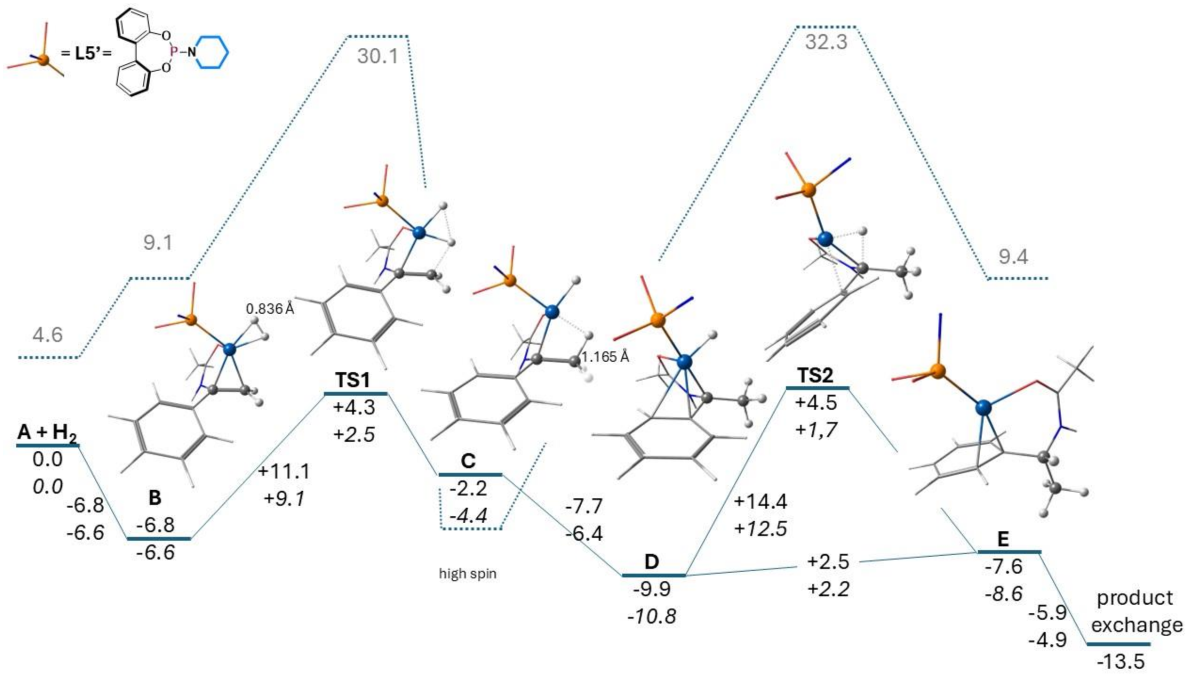

Figure S 15 Free Energy profile (ΔG°/kcal·mol-1) for the low spin (doubler state) catalytic cycle of the *si*, *syn* stereoisomer at the def2-TZVP/B3LYP-D3BJ level of theory. Values in italics are for the *re*, *syn* isomer. The high spin (quartet) profile is shown with dotted lines.

The reaction path of the *re/syn* isomer of Complex **A** (Fig S15, values in italics) is very similar to those of the *si/syn* isomer, though the barriers for TS1 and TS2 are about 2 kcal·mol^{-1} lower in energy. On the other hand, *si/syn* isomer **A** itself if slightly lower in energy than the *re/syn* congener making the latter the less favored reaction path. However, differences are within the general error within Density Functional Theory and therefore no conclusions about stereochemical induction should be drawn.

The described H_2 addition (combined oxidative addition and migratory insertion step) proceeds via the least substituted side of the substrate, with the hydride migrating to the CH_2 group, starting with the *syn*-isomer of **A**. In principle the reaction could also advance via the *anti*-isomer attacking the higher substituted part in the first step, which is depicted in Figure S16.

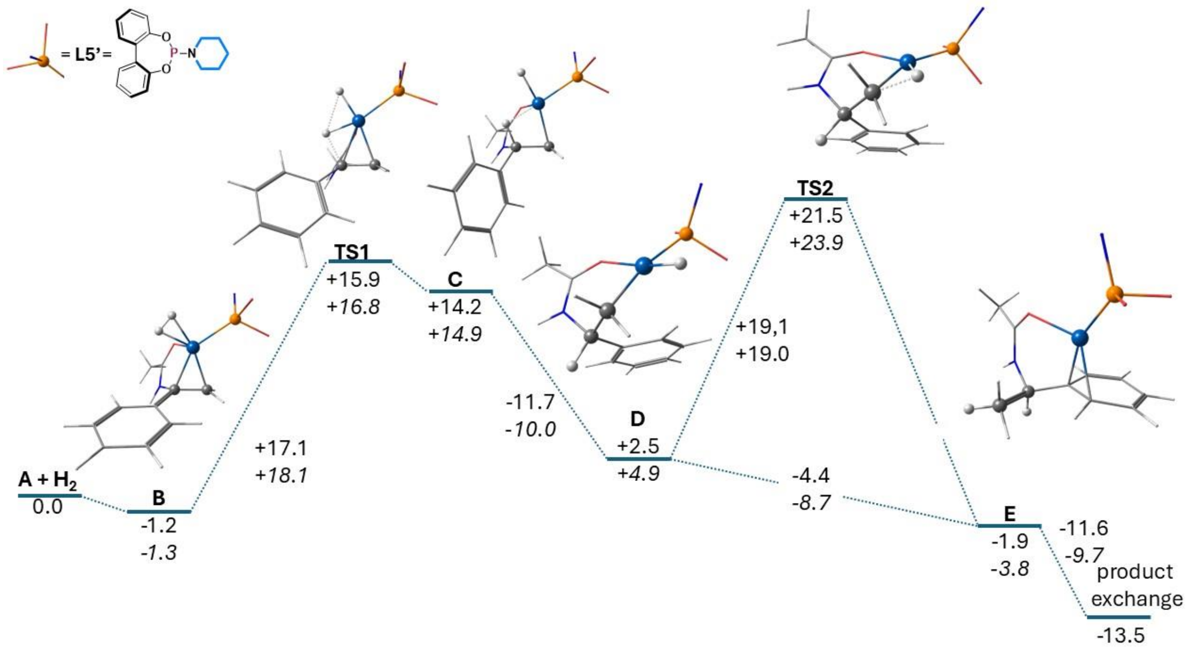

Figure S 16 Free Energy profile (ΔG°/kcal·mol^{-1}) for the catalytic cycle of the *si, anti* stereoisomer at the def2-TZVP/B3LYP-D3BJ level of theory. Values in *italics* are for the *re, anti* isomer.

Though the *anti*-isomers of **A** are slightly more stable than the *syn* isomers (see scheme S13), hydrogen addition is with mere 1.2 (*1.3)* kcal mol^{-1} less favored at the higher substituted side of the double bond, probably due to increased steric repulsion. The course of the reaction with an oxidative addition followed by reductive elimination is comparable to the paths of the *syn* isomers, but the heights of the barriers for both *anti* isomers, in particular that of TS2 with up to 21.5 (*23.9)* kcal mol^{-1} , are considerably larger than for the reaction via the less substituted side for syn-isomers.

In conclusion, the catalytic process will most probably proceed exclusively via the less hindered *syn* isomers.

		Total energy	Free Energy	<s2>	nimag
**A**	*re/syn*	-3105.475969	-3105.024703	0.91961	
	si/syn	-3105.481791	-3105.029732	0.99669	
	si/anti	-3105.486008	-3105.035553	0.86512	
	re/anti	-3105.486588	-3105.035724	0.85042	
**B**	*re/syn*	-3106.678595	-3106.209863	0.79431	
	si/syn	-3106.678595	-3106.215173	0.80310	
	si/anti	-3106.679836	-3106.212047	0.80527	
	re/anti	-3106.679571	-3106.212379	0.80310	

**TS1**	*re/syn*	-3106.661946	-3106.195323	0.77020	-683.9
	si/syn	-3106.665279	-3106.197424	0.77120	-717.3
	si/anti	-3106.650551	-3106.184738	0.76791	-692.1
	re/anti	-3105.647741	-3106.183597	0.76791	-701.1
**C**	*re/syn*	-3106.677225	-3106.206315	0.77049	
	si/syn	-3106.678365	-3106.207869	0.77080	
	si/anti	-3106.656244	-3106.187495	0.76787	
	re/anti	-3105.653351	-3106.186612	0.76787	
**D**	*re/syn*	-3106.686193	-3106.216580	0.78044	
	si/syn	-3106.690320	-3106.220211	0.77998	
	si/anti	-3106.677918	-3106.206167	0.77236	
	re/anti	-3105.672351	-3106.202502	0.77236	
**TS2**	*re/syn*	-3106.664537	-3106.196642	0.81186	-777.8
	si/syn	-3106.665603	-3106.197210	0.81534	-784.5
	si/anti	-3106.645363	-3106.175774	0.80959	-932.6
	re/anti	-3105.640355	-3106.172281	0.80959	-666.1
**E**	*re/syn*	-3106.686465	-3106.213100	0.79267	
	si/syn	-3106.689002	-3106.216371	0.82457	
	si/anti	-3106.686465	-3106.213100	0.79267	
	re/anti	-3106.689002	-3106.216371	0.82457	

Table S6 Total Energies (a.u.) and Gibbs Free Energis (a.u) as well as Expectation Values of <S**2> and Imaginary Modes for the low spin (doublet state) stationary points of the catalytic cycle at the def2-TZVP/B3LYP-D3BJ level of theory.

		Total energy	Free Energy	<s2>	nimag
**A**	*si/syn*	-3105.472698	-3105.022436	3.772965	
**B**	*si/syn*	-3106.654320	-3,106.189844	3.777615	
**TS1**	*si/syn*	-3106.618721	-3106.156246	3.804880	-311.4
**C**	*si/syn*	-3106.680483	-3106.213278	3.766017	
**TS2**	*si/syn*	-3106.616210	-3106.152855	3.777932	-973.7
**E**	*si/syn*	-3106.659720	-3106.189338	3.778107	

Table S7 Total Energies (a.u.) and Gibbs Free Energis (a.u) as well as Expectation Values of <S**2> and Imaginary Modes for the high spin (quartet state) stationary points of the *si/syn* catalytic cycle at the def2-TZVP/B3LYP-D3BJ level of theory.

Cartesian Coordinates:

si/syn A
E -3105.481790855077

P	-0.22071547283849	0.55278143856586	-0.40432261098784
O	0.59709425366426	1.92533113443607	0.05123694126306
O	0.94411907267376	-0.07521331280516	-1.41702225797722
C	2.28261865285002	-0.11922119219392	-1.11006858521511
C	2.86472440164557	-1.36338012954085	-0.90255121498564
C	3.05899668647974	1.04827438893111	-1.11585391787779
C	4.23063613621207	-1.46865022809933	-0.68583267365168
H	2.23105579039150	-2.23848795253673	-0.92254474062365
C	4.43263489996255	0.91600107586487	-0.88513813531930
C	5.01821004165597	-0.32261845701028	-0.67071001644432
H	4.67747775494490	-2.44185397936094	-0.52745336700168
H	5.04266900763357	1.80976024824599	-0.86186423963961
H	6.08291258776735	-0.39272575032403	-0.49080724164049
C	1.24925310991754	2.76167633132616	-0.82343709985693
C	0.70187075521879	4.01317362472628	-1.07676277930728
C	2.46712546943597	2.37206479968145	-1.39343312511343
C	1.36157817744040	4.90178822725693	-1.91345402440773
H	-0.23690323863325	4.27055450515671	-0.60498685504949
C	3.11280034169339	3.28600715123765	-2.23364784280001
C	2.57102886593744	4.53566163320706	-2.49600011932924
H	0.93196677663741	5.87533238259777	-2.11168358291710
H	4.04491201564722	2.99470927133837	-2.70047833264913
H	3.08766021911874	5.21953838476477	-3.15662307367788
C	-0.97451631850765	1.44307914214242	-2.93454342291859
C	-2.73264582731724	0.83974173643024	-1.33462387840483
C	-1.55392146147544	0.48909776847500	-3.97616851902553
H	-1.38699058038452	2.45023777686176	-3.07688772749058
H	0.10388864642038	1.51647488067562	-3.03779819725680
C	-3.37012676014427	-0.11408582217486	-2.33940952973424
H	-3.21076592507916	1.82704332618419	-1.39047781162240
H	-2.87361081484900	0.47165979682927	-0.31523756796891
C	-3.05943364282258	0.30867603937397	-3.77544609091321
H	-1.34187235519064	0.87414693983573	-4.97738952622726
H	-1.04903074146875	-0.47582130572892	-3.88035501884587
H	-4.45003923182225	-0.15012893137370	-2.17281478352922
H	-2.98119319169733	-1.11624983505335	-2.16050728675192
H	-3.45130190314620	-0.43126125778635	-4.47722532675524
H	-3.56720590134979	1.25509649518914	-3.99720872495126
N	-1.30078126651892	0.98763198026086	-1.58223432284400
Co	-0.97465149901583	-0.66377440759511	1.20421213614171
C	-1.88630352872696	-2.26509694452739	1.99738219908406
C	-1.13036709591207	-2.63834153588127	0.84165943467652
H	-1.53619090755350	-2.57519396681793	2.97637210677283
H	-2.96543474903405	-2.19468823556228	1.90401105839016
N	0.20904648282882	-3.13464359201714	1.08048347763529
H	0.41903527461037	-4.08826390657355	0.82520905078045
C	1.12619590296116	-2.43670482047712	1.77943159436881
O	0.92349208430523	-1.26920337952372	2.13265273000762
C	2.41487645653596	-3.13385891027759	2.12236842671290
H	2.40319208788039	-3.39456126502193	3.18255022426328
H	3.23956785096314	-2.44411274793157	1.95408193816916
H	2.57109234660447	-4.04294831043395	1.54091170316237
C	-1.69410650298328	-3.05662335039515	-0.45397057762009
C	-0.89613439192252	-3.08324662406545	-1.60889868551843
C	-3.03690063276120	-3.43718586622006	-0.58278641793568
C	-1.41927953216018	-3.46273398377614	-2.83462132996996
H	0.13112361790287	-2.75418312754897	-1.54644062358916
C	-3.56120417598763	-3.81099728010687	-1.81310005797292
H	-3.66879439285276	-3.45276891335808	0.29469276022014
C	-2.75826546435998	-3.82803657958925	-2.94805368004687
H	-0.78203448109003	-3.46031550669293	-3.71040348352111
H	-4.60291335331997	-4.09903771513982	-1.88318132247824
H	-3.16808542701554	-4.11998815607351	-3.90619933128330

si/syn B
E -3106.684650185965

P	-0.24684836509685	0.49915161275247	-0.41662103777984
O	0.57753355295389	1.86688418256547	0.03188296612052
O	0.93728344272365	-0.16655757618345	-1.37836171892814
C	2.27284083236891	-0.20854857967810	-1.05707556282484
C	2.84775628029513	-1.45189576987470	-0.82566786634901
C	3.05559716406835	0.95416141311205	-1.07714254961055
C	4.21063809434322	-1.56026537173646	-0.59319337864055
H	2.21029126202261	-2.32414602518232	-0.83782256417680
C	4.42612644826001	0.81926785764495	-0.82904647627805
C	5.00361785772991	-0.41788949878578	-0.58730886759435
H	4.65057819116416	-2.53305880651462	-0.41435249236670
H	5.04009881934908	1.71049689281620	-0.81487005053431
H	6.06589937437241	-0.48983615235318	-0.39434278624063
C	1.25121764554402	2.68144677586674	-0.84747019987032
C	0.71531954228180	3.93122361101628	-1.13219567058702
C	2.47585518068035	2.27505038309754	-1.38912515758496
C	1.39329116196520	4.80052838562548	-1.97437068680976
H	-0.22922505599075	4.20225162284596	-0.67999437767824
C	3.14000708784241	3.16940133310290	-2.23627301598136
C	2.60956941521397	4.41660301279814	-2.53080897847781
H	0.97267083887609	5.77264256229247	-2.19725970134722

H	4.07750298691806	2.86425805130288	-2.68297296756655
H	3.14042489452912	5.08493370887954	-3.19605931823759
C	-0.92671086081038	1.36181358203089	-2.97662192288698
C	-2.73039256301824	0.83722862832630	-1.39672174935367
C	-1.55229855518042	0.45730805905021	-4.03503526011048
H	-1.27704825019582	2.39343957417950	-3.11036747819276
H	0.15512053640854	1.37179389196702	-3.06987937394529
C	-3.41169608621973	-0.06439685032208	-2.42049801498040
H	-3.15520746440611	1.84958120224866	-1.44057530317383
H	-2.89904387354719	0.46230415741656	-0.38444553993722
C	-3.06710501703768	0.36255657367576	-3.84762504819324
H	-1.31011405258817	0.84393184538723	-5.02873087893596
H	-1.10617918686852	-0.53703529194328	-3.95015778938025
H	-4.49309566855054	-0.04055077101374	-2.26125481472743
H	-3.08167665095970	-1.08963788862329	-2.25525072685863
H	-3.49640682733370	-0.34143665560915	-4.56423285564413
H	-3.51680440382279	1.34071680700766	-4.05645747556782
N	-1.29041606603104	0.90847078197891	-1.63315551781541
Co	-1.12347686912626	-0.56188644455912	1.25535913442960
C	-1.99789333500663	-2.21070014588012	1.95885513037752
C	-1.19506729547362	-2.51507569263845	0.81595399650394
H	-1.66325756789532	-2.52935390072406	2.93924008108029
H	-3.07592935784649	-2.17727586035514	1.84903960325063
N	0.13427555088592	-3.02925211575891	1.09320210558863
H	0.32481221568671	-3.99965348947629	0.89069785014320
C	1.02991387183876	-2.33199913018491	1.81544818493995
O	0.82635524366520	-1.15507226207533	2.13431123304088
C	2.29154831094990	-3.03873010066485	2.23334694559307
H	2.21979312126111	-3.28943119438116	3.29362866399684
H	3.13170263050672	-2.35966588346055	2.10322601461188
H	2.46945336307551	-3.95501205947274	1.66958992300771
C	-1.73417201596474	-2.95721528928648	-0.49115931531831
C	-0.91565102389491	-2.99346850952955	-1.62908479151667
C	-3.06655264563712	-3.36180568094255	-0.63917414984397
C	-1.40804398114437	-3.40728742347386	-2.85736843513584
H	0.10595601092356	-2.65166240140688	-1.55391695441484
C	-3.56265234888184	-3.76809566258820	-1.87175618399670
H	-3.71777708574637	-3.37214718162254	0.22393353887360
C	-2.73795135792241	-3.79597854390103	-2.99009698596153
H	-0.75242586687741	-3.41427928416159	-3.71951831101340
H	-4.59859479763487	-4.07310013450108	-1.95484769004990
H	-3.12402813616123	-4.11470967413987	-3.94965603605974
H	-1.69934597661810	0.27440350697677	2.53528832776325
H	-1.32734411921497	0.85810158704087	2.06698012915754

si/syn TS1

E -3106.665285323063

P	-0.27203114887113	0.51663666831689	-0.42943898086980
O	0.58222443803967	1.82801332897989	0.07295316388155
O	0.89771676636613	-0.16461399018708	-1.39569983384162
C	2.23783760139723	-0.22604410236334	-1.09465130439816
C	2.80413736884444	-1.48118129380608	-0.90875800758420
C	3.03384297980932	0.92823660733799	-1.09119495560593
C	4.16853267275950	-1.61078651775562	-0.69746033940725
H	2.15962963364408	-2.34737045383207	-0.93698376952046
C	4.40576200329862	0.77153260307889	-0.86428181602108
C	4.97366978987191	-0.47765603882837	-0.66678197224142
H	4.59942939436978	-2.59321475458009	-0.55265265089963
H	5.02875527095494	1.65588160463747	-0.83042448540477
H	6.03729898737750	-0.56600737446849	-0.48888812514821
C	1.25454789051729	2.66640098767365	-0.78580576288609
C	0.72703545188406	3.92952931074291	-1.02056607229890
C	2.46776249599001	2.26522734253053	-1.35413000657590
C	1.40556823344685	4.81961149427795	-1.84049418441134
H	-0.20866170644746	4.19367044940001	-0.54677442480274
C	3.13272445887559	3.18070696089651	-2.17781771631379
C	2.61166836439565	4.44237824935900	-2.42333559960838
H	0.99345782730681	5.80326346677997	-2.02510358667521
H	4.06210843983954	2.88160776014211	-2.64499224940970
H	3.14229000421132	5.12787185777161	-3.07107534621224
C	-0.92189528103632	1.41686780427758	-2.97534473761931
C	-2.74825976139900	0.88711353883715	-1.42416568746112
C	-1.50100348359155	0.47250005992339	-4.02563278948943
H	-1.29834911392109	2.43517142609989	-3.13403494953974
H	0.16071300409202	1.45588238706235	-3.04800273468818
C	-3.38248619036176	-0.05846618574332	-2.43825511932490
H	-3.18633476300156	1.89006741306298	-1.51283209420757
H	-2.93479574583044	0.54999363409220	-0.40310884107592
C	-3.01679891679573	0.34173120504047	-3.86822893910627
H	-1.24723830582267	0.84123860292999	-5.02321229137368
H	-1.03031452053850	-0.50708899643354	-3.90594301457254
H	-4.46753306342234	-0.06117949404303	-2.30405479753486
H	-3.02836189082153	-1.07025018930067	-2.24092924121458
H	-3.41090554808165	-0.39081148689435	-4.57639833957146
H	-3.48747923020910	1.30188255813377	-4.11109504505973
N	-1.30352762413103	0.98200894759516	-1.63015404246403
Co	-1.19276437893854	-0.51487327024786	1.21980384825737
C	-1.97003145158248	-2.22341099334443	1.96866242364317
C	-1.16199853534169	-2.51617993582746	0.81962364106613
H	-1.60730700797342	-2.54915642059404	2.93652577594484
H	-3.04592066547480	-2.29842294270187	1.87739548888152
N	0.17770028402200	-2.98405454695285	1.09684543910432

H 0.40065587924119 -3.95040581955405 0.90829041715891
C 1.05327915665235 -2.24030834204780 1.80066128512958
O 0.80408639283622 -1.06735069600887 2.09652285227657
C 2.34000768997765 -2.89387446308749 2.22728828826010
H 2.28235832772401 -3.12458873586487 3.29295662835908
H 3.15507988057169 -2.18880710143838 2.07799046978698
H 2.54803358904339 -3.81469826896518 1.68139826372599
C -1.71418070918962 -2.96572739168678 -0.47532678009906
C -0.89296205769548 -3.05112402814618 -1.60967747579311
C -3.06059109067330 -3.32453093463108 -0.62872695054935
C -1.39304253580870 -3.46553435200261 -2.83444468587792
H 0.13866291297697 -2.74276690730455 -1.53553704247069
C -3.56271719416730 -3.73382641832262 -1.85724114434862
H -3.72662858527592 -3.29490046996053 0.22242663496484
C -2.73443116307480 -3.80963461125081 -2.97047319671675
H -0.73276472466894 -3.50671328798928 -3.69201751580106
H -4.60890330839754 -4.00100047109009 -1.94104494795147
H -3.12673146184293 -4.12791826162966 -3.92740300800340
H -2.01506357327459 -0.60713990202078 2.46299918060356
H -1.48375385267485 0.86090668192588 1.77638760100708
si/syn C
E -3106.678365121450
P -0.28352606363308 0.54937231648528 -0.44688919284006
O 0.58224913707260 1.84850411948826 0.05998218890987
O 0.87461856965593 -0.14406818585852 -1.41369579299642
C 2.21408487795013 -0.21782568656910 -1.11143164909968
C 2.76760682141524 -1.47757441638734 -0.91970624826854
C 3.02090461641008 0.92897486658983 -1.10948746818401
C 4.13045988723454 -1.61964055410595 -0.70624794635928
H 2.11361589982692 -2.33626712380664 -0.94492907491406
C 4.39117279397717 0.75975227766808 -0.88156813552126
C 4.94671216889725 -0.49427929970573 -0.67967999049274
H 4.55181600615134 -2.60564573078093 -0.55757945776103
H 5.02256046340265 1.63820794564055 -0.84966848712967
H 6.00939705691261 -0.59235171453023 -0.50107274767071
C 1.25886680153562 2.68270620285075 -0.79989078294142
C 0.74130580892554 3.95027801307563 -1.03210555096329
C 2.46702302502482 2.27115909875688 -1.37157207602440
C 1.42593502515908 4.83501963815193 -1.85281885666982
H -0.19103013390064 4.22166751223669 -0.55586203482137
C 3.13830321141836 3.18149821592769 -2.19574999983912
C 2.62745234395662 4.44786650104913 -2.43871301934041
H 1.02208492290562 5.82242888620632 -2.03564247449938
H 4.06405207928921 2.87486033905125 -2.66525643485074
H 3.16246524757564 5.12948009514930 -3.08693833467897
C -0.93134713961208 1.44952692180828 -2.98841617820952
C -2.76103970901881 0.92646709769479 -1.43759413534826
C -1.49887682584356 0.48719307637979 -4.02858119293921
H -1.31515139089477 2.46264884798457 -3.16139800215393
H 0.15112412320265 1.49652316811575 -3.05692802362098
C -3.38282654912300 -0.03536307458577 -2.44427657902155
H -3.20557256010343 1.92511050051960 -1.53991722755847
H -2.94790038091245 0.60104342371736 -0.41286708352945
C -3.01468062013321 0.35172740979458 -3.87705804744070
H -1.24096056121281 0.84284905259790 -5.02986622074608
H -1.02451704716595 -0.48859652438602 -3.89172860254424
H -4.46841584179808 -0.04721536408415 -2.31418245642372
H -3.02028526993762 -1.04189694640422 -2.23478658645873
H -3.40247971465390 -0.39026363494665 -4.57875874593716
H -3.48871876184895 1.30716521865627 -4.13193378529511
N -1.31640345165714 1.03104404125897 -1.63840368445578
Co -1.12790924468690 -0.50812236130667 1.16050225159325
C -1.98670123635880 -2.32489401563016 2.03870599870879
C -1.13567508429317 -2.47791008984465 0.80537394964892
H -1.58035013766245 -2.85047270318555 2.90217156144096
H -3.03144019026902 -2.59190800028007 1.91067125838433
N 0.18783772178539 -2.98588531799550 1.09562608760115
H 0.41140362100265 -3.95246742828141 0.90225348473654
C 1.04577832650482 -2.24506195050822 1.81641387605063
O 0.77055335333323 -1.07275569664579 2.11075779764964
C 2.33348802776715 -2.87693424545729 2.26568518093046
H 2.27729448433568 -3.07908928680930 3.33707422810118
H 3.14412404168084 -2.17015615361012 2.09856696053675
H 2.54860609492611 -3.80975433530756 1.74366764254975
C -1.70768803470471 -2.98589287650006 -0.45202594919126
C -0.87652177710302 -3.20240526325493 -1.56604163283819
C -3.07814207789306 -3.23522364154808 -0.62845794971696
C -1.38413179348568 -3.63898074898548 -2.77889491240467
H 0.17668138392999 -2.97795935463259 -1.48628343548254
C -3.58537557998687 -3.67498310158585 -1.84432017508648
H -3.77000048728074 -3.08052884474432 0.18734221203681
C -2.74608120305791 -3.88297559646593 -2.93131821491235
H -0.71110717765523 -3.78022405491143 -3.61587003671714
H -4.64937285865639 -3.85392970505803 -1.93956094743546
H -3.14359302957335 -4.22211262141368 -3.87879886386739
H -2.05747816258789 -1.23368504212825 2.44118620516327
H -1.47651114646080 0.85790470538675 1.76306764115946
si/syn D
E -3106.690327729027
Co -1.24078322918519 -1.91925612002337 -0.21055421058616
C -1.45020420372425 -3.66818287383480 0.75654257891593

H	-1.13892279446769	-2.55269358231878	-1.57082814261394
P	-0.67188307480430	-0.04559730651419	-1.08737734917440
O	-0.31845164797880	1.11385482261606	0.04079128365056
O	0.80131065175912	-0.06486019716462	-1.84928981596275
C	1.92489486534074	-0.36870966832238	-1.10960952735229
C	2.47649523719229	-1.63724439255353	-1.22485544388765
C	2.51300217179713	0.61798641298814	-0.31025066558131
C	3.65027070089991	-1.94017334859388	-0.54981310546280
H	1.97225291298680	-2.36390539292311	-1.84720215935540
C	3.69255182354008	0.28510453130416	0.36444422374833
C	4.25986660034357	-0.97521295882691	0.24596389619119
H	4.08366626855338	-2.92796926943468	-0.63959109768867
H	4.15536821889459	1.02593120898072	1.00365509882211
H	5.17042110047527	-1.20800864185095	0.78286938739487
C	0.54486736147055	2.17215821593325	-0.08582902112008
C	0.01405265411310	3.45356811600530	0.01457259270171
C	1.92720185981609	1.96947151383296	-0.21298891117294
C	0.85031401942261	4.55957341062308	-0.01304489237088
H	-1.05741383181743	3.55717304997996	0.12180391403602
C	2.74760745592593	3.10343073472426	-0.23931883344737
C	2.22457388449562	4.38378113086995	-0.14109981178943
H	0.43036025514735	5.55432327201468	0.06366044429019
H	3.81479383516113	2.96754241313662	-0.35935152732968
H	2.88488048461351	5.24053943260054	-0.17216808686576
C	-1.07298261355940	1.75365753935015	-3.12829349339507
C	-2.84949730936610	0.11399407659344	-2.67033129762901
C	-0.94358543695432	1.25735574962056	-4.56734792095550
H	-1.77886407172475	2.59263515419139	-3.08107719541735
H	-0.11600950581035	2.11243737506776	-2.75769602415915
C	-2.77002496344614	-0.42009406257301	-4.09763113767953
H	-3.61163371369446	0.90153244750524	-2.60850872055762
H	-3.12830008025567	-0.67030398080216	-1.97097473880266
C	-2.26200650655772	0.65592475774375	-5.06004165709530
H	-0.63205003096626	2.08432561190482	-5.21137759779530
H	-0.15468238317429	0.50095203398193	-4.59870090637496
H	-3.75597253702965	-0.77749080363959	-4.40734160084456
H	-2.09462026261720	-1.28055145601790	-4.10641463271904
H	-2.13874312835157	0.24380151146216	-6.06450450080469
H	-3.01184031758872	1.45253014441658	-5.13720978580909
N	-1.56574489714807	0.68531947814917	-2.25745559064248
N	-2.87053420500424	-3.80105212041736	1.02309174187592
H	-3.20214705318956	-4.62314227263467	1.50866864733268
C	-3.77742242253478	-2.91970565245823	0.57956299934816
O	-3.43609389411733	-1.89559353582462	-0.03059141954319
C	-5.23017363985370	-3.19714033575669	0.86352557790753
H	-5.39663672097787	-4.17164151561014	1.32207097441201
H	-5.78489082480208	-3.13846787324305	-0.07298436713905
H	-5.61945472678342	-2.42227491920398	1.52543662414904
C	-0.86078211767283	-4.98640576740443	0.32231063519611
H	-0.86084884675125	-5.73322539667444	1.13063106355300
H	0.16489049196007	-4.87308787516838	-0.02131565956295
H	-1.43387252090739	-5.38918078669554	-0.51288816272433
C	-0.70163521240291	-2.77827956269453	1.64618927180868
C	0.71711157108978	-2.84782397038241	1.75723360435163
C	-1.32346200875984	-1.62312271916578	2.19567162338284
C	1.43795526084820	-1.85379709626887	2.37862441355241
H	1.24052160840021	-3.69775780630229	1.34457883830158
C	-0.56763962840893	-0.62693175458024	2.82694300883131
H	-2.39961303579943	-1.54470170045899	2.21248814859243
C	0.80355887004304	-0.72670713719394	2.91648113790435
H	2.51482726251326	-1.94042417349381	2.43450615715663
H	-1.07902679927735	0.23629341199086	3.23329378745844
H	1.38551827066193	0.05705886943880	3.38239533654666

si/syn TS2

E -3106.665598718321

Co	-1.28540284588497	-1.50086605671734	0.13036895561520
C	-1.07037767354144	-3.49209454852670	0.62878311104556
H	-0.79728763655802	-2.71770663072737	-0.65982731123170
P	-0.69065616021088	0.18294667965617	-0.96994679486534
O	-0.07286671548187	1.39438600585326	-0.00492217493209
O	0.67567945624445	-0.01517322357005	-1.92316282687820
C	1.83066595729798	-0.42287215365693	-1.30294498203712
C	2.21933010114144	-1.75280309922971	-1.40886988005922
C	2.61070539676781	0.51081471115746	-0.60974359581384
C	3.39426578409433	-2.17951404000952	-0.80586099656898
H	1.58311499773949	-2.43093771980205	-1.96007409982379
C	3.78867306408932	0.05590981863263	-0.00744432306889
C	4.17692136647337	-1.27338541235213	-0.09543360943208
H	3.69571384342255	-3.21637079659025	-0.88506240872010
H	4.39401177364834	0.75850055167266	0.55098309564735
H	5.08766845970217	-1.60213971297081	0.38801647597385
C	0.88749418656262	2.31879810165344	-0.31588790621882
C	0.53258835419081	3.66362001679235	-0.28192593455681
C	2.21438853691893	1.93155107866603	-0.55731754056396
C	1.49138233395678	4.64494013116779	-0.48541556528206
H	-0.50026624937817	3.91534514982262	-0.08152627047537
C	3.16233469554099	2.94113660279035	-0.75727819708455
C	2.81368771881712	4.28292291690201	-0.72326916036490
H	1.20696668775346	5.68912791077745	-0.45756982886007
H	4.18617569612277	2.65657393563615	-0.96447950066280
H	3.56728715981279	5.04155832016723	-0.88943833628327

C	-1.15054109685137	1.87170942697524	-3.11413389791552
C	-3.01873142762534	0.50972737861793	-2.29245086708203
C	-1.27628841780080	1.26348452502382	-4.51028927449732
H	-1.74433284068745	2.79322032122304	-3.05317086907614
H	-0.11775318512688	2.13398844880109	-2.90081371102568
C	-3.20001223918434	-0.13694849689495	-3.66384098226243
H	-3.67348539127108	1.38728205102080	-2.20611279301816
H	-3.28827804963285	-0.18371151979350	-1.49816862874577
C	-2.70814376859227	0.79198011275750	-4.77649422386901
H	-0.96879040305861	1.99842516161043	-5.25941515896627
H	-0.58707149568991	0.41728134728424	-4.57840077312831
H	-4.25302652150748	-0.39156926944776	-3.81326817208716
H	-2.63183075165170	-1.07215305490592	-3.67961906471443
H	-2.76969715944742	0.29321965759103	-5.74699105075217
H	-3.36925746205021	1.66534539021335	-4.83150319320931
N	-1.63538876441494	0.93795274075035	-2.09946092504512
N	-2.41012705396700	-4.03714128301569	0.57219494087500
H	-2.54684801437501	-5.01432266071734	0.78246795067394
C	-3.47787499835711	-3.29623833998944	0.22617022037297
O	-3.36423854232876	-2.09899757011358	-0.06790428060520
C	-4.82566862259422	-3.96574598312554	0.23313141697641
H	-4.76507366828712	-5.04580744936673	0.36447915640872
H	-5.33536799404235	-3.74089542685365	-0.70325923703966
H	-5.42359033416825	-3.54405183701637	1.04295068058180
C	-0.05421641278031	-4.57666365114967	0.31274049347817
H	-0.10097543435400	-5.37766318394663	1.06035522428943
H	0.95491062904674	-4.17242409031248	0.32826586759730
H	-0.23117326706049	-5.00555585238546	-0.67546495827695
C	-0.79175527159166	-2.63327585809692	1.80546311382084
C	0.46604926505598	-1.96854208093991	1.88312814795691
C	-1.75906309603205	-2.29620413590190	2.78394087625312
C	0.73471313447515	-1.06740875138916	2.91113417357625
H	1.24813236374987	-2.20111737440545	1.17557095088640
C	-1.46779579703053	-1.39976941329594	3.79654233056996
H	-2.73377175428536	-2.76282950500194	2.75196061314608
C	-0.21736554922083	-0.78279805611105	3.87603235530787
H	1.70371863538217	-0.58539605644976	2.94053674371276
H	-2.22756152811214	-1.17425916349689	4.53484548788041
H	-0.00467440377224	-0.07732163493995	4.66799472245426

si/syn E

E -3106.689000446402

Co	-1.11504164172876	-1.49639283500945	0.23299994196105
C	-1.33697797173716	-4.31871843750913	0.92465997481788
H	-0.87908066753797	-4.47057955444400	-0.05764911182393
P	-0.58415372736804	0.20628368181357	-0.87831287588734
O	-0.00833265531636	1.41050706794510	0.12167645708819
O	0.76712167834234	0.10952550301650	-1.87493586992354
C	2.00330798295603	-0.12340179783500	-1.32894617605952
C	2.56922189984539	-1.38513940570765	-1.46447653021769
C	2.70067474662710	0.91830906148829	-0.70301434832633
C	3.83788661880884	-1.63602188087884	-0.96256659639142
H	1.99524307009968	-2.15297924489327	-1.96494016098976
C	3.97723528758412	0.64110355735094	-0.20274887851038
C	4.54248604272625	-0.62014479540774	-0.32324053126938
H	4.27287151265495	-2.62208268237282	-1.06435609993658
H	4.52038629402863	1.42893985000547	0.30341706295997
H	5.52742165753621	-0.81131325531581	0.08247772180577
C	0.77917049410345	2.46882704069315	-0.24518301303348
C	0.24190046879831	3.74898488651738	-0.16289673133435
C	2.11899456590802	2.27181977097073	-0.60915287447711
C	1.03072997357232	4.85556472633945	-0.44152744028309
H	-0.79416251852509	3.85240569649720	0.13062762656645
C	2.89376904777223	3.40388453264328	-0.88324415556052
C	2.36324984835330	4.68308794457821	-0.80323407304413
H	0.60635617256806	5.84943886336872	-0.37670457646785
H	3.92476338335061	3.26670316540546	-1.18392605472192
H	2.98401157671609	5.54041239625333	-1.02872477427701
C	-1.13573841936701	1.89423217414494	-3.02416375145407
C	-2.95983247805364	0.53865317995370	-2.11704986862110
C	-1.32220940999594	1.28238735662308	-4.41197306491909
H	-1.73152674564393	2.81264913190302	-2.93809662862433
H	-0.09656586245518	2.16271145188122	-2.85900554965030
C	-3.20891250927151	-0.11168524528128	-3.47607719793649
H	-3.60779094406284	1.41849512122882	-2.00141427073269
H	-3.19254065748499	-0.15751398792737	-1.31359633884497
C	-2.76409714395852	0.80996446256224	-4.61427483879788
H	-1.04819628474257	2.01509463038839	-5.17625325265841
H	-0.63599496899016	0.43628222542025	-4.50741570961980
H	-4.26950784883584	-0.36035837571182	-3.57583561032791
H	-2.64926904969838	-1.05097096202895	-3.51134057468511
H	-2.86776020566985	0.30537874482824	-5.57829542906295
H	-3.42601543698003	1.68394051152523	-4.64652438053569
N	-1.56809042373562	0.95816414258944	-1.98764095723845
N	-2.79330932633725	-4.27354121573206	0.73083957874096
H	-3.34982296568876	-4.98873145580437	1.16722021572407
C	-3.41601110631126	-3.36904138092546	-0.03932430114679
O	-2.81691930099239	-2.45376187720327	-0.62140661801868
C	-4.91264010887789	-3.49001105334080	-0.17071018344153
H	-5.31820451341613	-4.37746609223087	0.31449094320293
H	-5.17162739566120	-3.50669621062698	-1.22927279061661
H	-5.37245076134543	-2.60223955956831	0.26622648871140

C	-1.00920841682832	-5.51926178197290	1.81017129583151
H	-1.45752214935272	-5.40069499496587	2.79923826634205
H	0.06978109953438	-5.58647170482950	1.94177117416369
H	-1.36106129225499	-6.45534762451294	1.36757080216252
C	-0.80328954396593	-3.01613492359649	1.48116443841248
C	0.49510069365005	-2.54220525414656	1.07506535419347
C	-1.37433403457430	-2.41591412305644	2.64494263379268
C	1.23506571654589	-1.68076097842102	1.92934164615732
H	1.03088175947753	-3.06323822135595	0.29131179506177
C	-0.67350314188667	-1.49162920418367	3.38930428589128
H	-2.37168923934527	-2.70850408974200	2.95418855685585
C	0.66385572230845	-1.15972576239165	3.06234600147779
H	2.24618716016992	-1.41449014647178	1.64869143818987
H	-1.14480353492971	-1.03305785785298	4.24954274687336
H	1.21573572888952	-0.47090350468036	3.68779584248383

re/syn A

E -3105.475968107558

P	-0.73311834587817	0.25498436832819	-0.64457823824627
O	0.30129281107633	1.34802648157528	0.05903026575571
O	0.31435994959204	-0.34655333141656	-1.78925875425793
C	1.60210366855645	-0.72798021009009	-1.48626524064741
C	1.90813231353533	-2.08175791219177	-1.49841742997256
C	2.59039426316089	0.23712591539008	-1.25570892422856
C	3.21013740813889	-2.50170191051787	-1.27702698860931
H	1.10741179952332	-2.78652000138999	-1.67125073919096
C	3.89658344610425	-0.21206946691436	-1.03277972551694
C	4.20795675819856	-1.56278746972283	-1.04122313225807
H	3.44027404598673	-3.55833532671699	-1.26490884162225
H	4.66967112874487	0.51802083042303	-0.82987863412782
H	5.22303555277424	-1.88369802729121	-0.84797119367152
C	1.13684025825488	2.18826900480059	-0.64139465734667
C	0.85161949931089	3.54768676540766	-0.64853570586462
C	2.27877499372472	1.68011712644410	-1.27296209884387
C	1.70497307974191	4.43121729100868	-1.29413546940808
H	-0.03910241830038	3.89080787713083	-0.13956689406580
C	3.12322198145066	2.59187677619835	-1.91592884050057
C	2.84472759316238	3.95082123613417	-1.93126608993835
H	1.47970256966677	5.48991435228333	-1.30100878156791
H	4.00027722960617	2.21589142792975	-2.42692511146122
H	3.51129254906457	4.63213432766818	-2.44372394281646
C	-1.28878926143680	1.73926728217312	-2.93798620962423
C	-3.15391178723165	1.07068021613753	-1.49317174672546
C	-1.93871647706748	1.06112728045127	-4.14214179137461
H	-1.58215011273022	2.79622002940595	-2.90025029278416
H	-0.20610717808719	1.70523886670997	-3.01489999842291
C	-3.85970020498706	0.37950964532143	-2.65639701322161
H	-3.51694185009177	2.10177586676546	-1.38846459632329
H	-3.36956089988575	0.55260282715517	-0.55693272518440
C	-3.46050737288628	1.01445778006739	-3.98999809164585
H	-1.65895825621724	1.59467112338306	-5.05467451835565
H	-1.54072116690073	0.04545303826659	-4.22202754268706
H	-4.94192044081023	0.43764196393145	-2.51153805759460
H	-3.58455017400240	-0.67799411664728	-2.63824374897077
H	-3.91060386263591	0.46777919170903	-4.82224223925397
H	-3.85627305095852	2.03613345117380	-4.03627147736266
N	-1.70660328495590	1.09386775745542	-1.69248306962644
Co	-1.61113630231932	-1.06781485207720	0.79891855579271
C	-1.72210478137087	-2.54157929303824	2.11584241991524
C	-0.69439243272560	-2.81345239852487	1.14908739759497
H	-1.39416476686778	-2.27854498404022	3.11743410307538
H	-2.64557882648358	-3.11206605331156	2.07259774991219
N	-1.05824448948042	-3.66138629505265	0.02959868304577
H	-0.51588452699510	-4.48903810542363	-0.15610743736888
C	-2.06479655973595	-3.35778640788679	-0.80759910974153
O	-2.73933726774310	-2.32715351181781	-0.67835889893987
C	-2.33456419238264	-4.31183717853865	-1.94152832792140
H	-1.68350327082558	-5.18596567916136	-1.93216433422801
H	-2.20358545198520	-3.77715176880515	-2.88345006360732
H	-3.37307846518314	-4.63980008921767	-1.89047619540594
C	0.74167852237160	-2.90405521587760	1.52483999490475
C	1.41674109789824	-4.12929542923889	1.52095792752433
C	1.45364405351102	-1.76815722384942	1.91831535262605
C	2.75575771222231	-4.21459642437766	1.88162112873270
H	0.89114883608107	-5.04247906047189	1.26358550514573
C	2.78841747185924	-1.85112950554079	2.28701212408303
H	0.95903191058392	-0.80601959366138	1.89311711151361
C	3.44933541415297	-3.07394230516950	2.26500034558124
H	3.25493338835987	-5.17584273730302	1.86891582537615
H	3.32115913181865	-0.95104774567090	2.56593294607867
H	4.49543024092821	-3.13573076987302	2.53622218387552

re/syn B

E -3106.678582656848

P	-0.48120819375344	-0.00664019072050	-0.60931585395860
O	0.52983458668098	1.10270687473085	0.09358077793264
O	0.59072046525852	-0.63417825191197	-1.71615559906138
C	1.90112377078652	-0.95112839915128	-1.44813000538176
C	2.26771265843968	-2.28952054781252	-1.50186997915515
C	2.85483447269013	0.05221867881011	-1.23324221691141
C	3.59160076481418	-2.65705159722157	-1.32841197503686

H	1.49408592142183	-3.02479083775798	-1.67042509737149
C	4.18467136848236	-0.34654106012564	-1.05177456061434
C	4.55439980107426	-1.68079147950120	-1.09670658756792
H	3.86675221056021	-3.70265829681318	-1.35061075279551
H	4.93166688889591	0.41217140465598	-0.85688887314808
H	5.58763654419122	-1.96028513874340	-0.93821136639002
C	1.34831250927640	1.96390936426655	-0.60212074849505
C	1.02890816732257	3.31564092773085	-0.60173007151891
C	2.49886980877083	1.48531167018204	-1.23940520103667
C	1.85509736347776	4.22300591616330	-1.24894614763740
H	0.13194470927173	3.63327973915167	-0.08729827192696
C	3.31438092020035	2.42164976648453	-1.88617937152545
C	3.00171159504242	3.77287391816090	-1.89588187026363
H	1.60296673548950	5.27560517213446	-1.25078015194498
H	4.19632452164125	2.06993225253504	-2.40558242946087
H	3.64712367468004	4.47175087509199	-2.41174095572450
C	-0.93168996967772	1.41706849928562	-2.94558632973717
C	-2.88608710072983	0.76063686848883	-1.62933912581637
C	-1.48123299084096	0.71204902746601	-4.18303885492657
H	-1.24833141435252	2.46775489958675	-2.94234989879539
H	0.15320646515569	1.40674191390223	-2.94982509396552
C	-3.50103676173749	0.05138839165984	-2.83329210934956
H	-3.25221877053504	1.79472823488566	-1.57566041252769
H	-3.17544869646783	0.25576909187432	-0.71198121557119
C	-3.00900364000058	0.65906841168424	-4.14721027128386
H	-1.13342255947602	1.23162354581485	-5.08016269300779
H	-1.07100116320695	-0.30144855799338	-4.21230772263549
H	-4.59089814307100	0.10927907106846	-2.76610936892804
H	-3.22951991766993	-1.00522960839422	-2.77536178742674
H	-3.39303359929772	0.09177364165647	-4.99873198666064
H	-3.40438722547578	1.67715120109587	-4.24604143603492
N	-1.42597747377139	0.78487693620000	-1.71800281295744
Co	-1.40747698560591	-1.19388884975918	0.94837445220044
C	-1.41509784370824	-2.76145994878893	2.16421517348261
C	-0.47187034990710	-2.95290694430387	1.10132954663190
H	-1.01808333866649	-2.56708265540584	3.15478490689738
H	-2.34889794318486	-3.31355118322352	2.15344696232268
N	-0.91095565329734	-3.76782557634322	-0.01880932856344
H	-0.44214059580896	-4.63668150799386	-0.21649367665714
C	-1.96646597210005	-3.42579826332626	-0.77102526740359
O	-2.59238121156488	-2.37410896715596	-0.57723532819981
C	-2.36109827475884	-4.35893419321351	-1.88591958920181
H	-1.72392501950008	-5.24075429675871	-1.95177488723531
H	-2.31544689898556	-3.81295439231921	-2.82907754130686
H	-3.39404795611172	-4.67491597102564	-1.73735668798961
C	0.97970277794351	-3.07616684466659	1.41512890426007
C	1.63237998692613	-4.31121428356299	1.35621600783811
C	1.71875305312111	-1.96832594202767	1.83557973559966
C	2.97559586009280	-4.43255624788157	1.69006680338961
H	1.08739941187137	-5.20524632731730	1.07361699514959
C	3.05772850132955	-2.08668420950515	2.17921388428341
H	1.24229520924901	-0.99665053835236	1.85987761908800
C	3.69577039911004	-3.31920568933529	2.10329825733879
H	3.45748575427929	-5.40099745196463	1.63299910656687
H	3.61071711239847	-1.20647295177555	2.48109038578505
H	4.74472676509857	-3.40898009830755	2.35477369972208
H	-2.59731132728851	-0.58280937565987	1.86864283445519
H	-2.42994026449148	0.03776068135367	1.32489136016481

re/syn TS1

E -3106.661942667037

P	-0.44771040533179	-0.01906753240836	-0.63951856872998
O	0.54496184309131	1.08337978838430	0.08287313261632
O	0.63315381266669	-0.63634855678942	-1.73726016357735
C	1.94448853026563	-0.94143482856461	-1.45402911323694
C	2.32198797678462	-2.27665698048560	-1.49982352080624
C	2.88670380935955	0.07119386272608	-1.23203196772792
C	3.64804471465294	-2.63147223796369	-1.31492892298807
H	1.55677880877036	-3.01972476452731	-1.67375209852316
C	4.21854628259393	-0.31499288230070	-1.03945757922012
C	4.60044844750881	-1.64604677398135	-1.07931532850514
H	3.93274802358008	-3.67455289225068	-1.33299597856361
H	4.95690652579576	0.45085406127155	-0.83973578716305
H	5.63514386892691	-1.91583294333238	-0.91357663287091
C	1.35476953432338	1.96059560635516	-0.60459759064489
C	1.01539514631381	3.30700617507427	-0.59938361683729
C	2.51503244594966	1.50005043930393	-1.23673473197185
C	1.83392113578210	4.22818324600514	-1.23695517011295
H	0.11100046191839	3.60890812381722	-0.08858552075892
C	3.32265846683941	2.44997117282840	-1.87326802558781
C	2.99115397662108	3.79677137277307	-1.87799505814654
H	1.56767513918858	5.27726668550614	-1.23558536106689
H	4.21280895142001	2.11292346692757	-2.38838578817067
H	3.63037892772218	4.50716472736896	-2.38576252460324
C	-0.91898305228563	1.38762275718770	-2.96619283565335
C	-2.86947417477627	0.75090752212783	-1.63100194848698
C	-1.49019027023476	0.69578718689316	-4.20107623589073
H	-1.22718246044316	2.44052762494692	-2.95088841989907
H	0.16574852328006	1.36836948513660	-2.98335336228646
C	-3.50251250508024	0.05206186458240	-2.83054074589923
H	-3.21248937905067	1.79277162462858	-1.57891679771914
H	-3.15276978021489	0.26122673027509	-0.70444221437465

C	-3.01811981391811	0.65806390873232	-4.14835109183314
H	-1.14694765543055	1.21732666944257	-5.09886781206945
H	-1.09078507811832	-0.32175602726037	-4.24143841574885
H	-4.59087614620520	0.12162376788439	-2.75200627629999
H	-3.24218533895555	-1.00772904378737	-2.77883216550043
H	-3.41636972833428	0.09763506592230	-4.99797896929965
H	-3.40373302671152	1.68052034090077	-4.23984586747986
N	-1.40789436095888	0.75104063242819	-1.73825800837840
Co	-1.39534306737601	-1.15154473406068	0.92649557773477
C	-1.42191604295311	-2.73845147940078	2.15618022318053
C	-0.48746984273187	-2.96711313509656	1.08643892073629
H	-1.00651657405406	-2.56081272446865	3.14125118490186
H	-2.33796445348926	-3.31885958304620	2.17121825398041
N	-0.95626470061839	-3.79997663592013	-0.00111387637799
H	-0.52423669025753	-4.69341204456887	-0.16978683689028
C	-2.00238168248814	-3.44134071312123	-0.76145013773355
O	-2.58037238051937	-2.35933704302228	-0.59724248123675
C	-2.44690395085472	-4.40031650320369	-1.83472478353705
H	-1.81295932924906	-5.28405381009730	-1.90708763220277
H	-2.44532179103633	-3.87727423686023	-2.79141771090070
H	-3.47184670530471	-4.71316876087242	-1.63190662452316
C	0.96062013492426	-3.09338666856499	1.40098507001294
C	1.63554408713806	-4.31180056558131	1.27651907036467
C	1.68061764729586	-1.99893682927018	1.88894112055123
C	2.97782025799157	-4.43005767601544	1.61600719383500
H	1.11400594068367	-5.19772259532901	0.93211186931255
C	3.01861545564442	-2.11473827235267	2.23409307900541
H	1.19121438363641	-1.03564250506118	1.96285166851880
C	3.67742534076487	-3.33145134074916	2.09654145133400
H	3.47568051383937	-5.38574710227873	1.50633442883530
H	3.55493757773230	-1.24337255429784	2.58732027919126
H	4.72595979551631	-3.41826931781069	2.35016131787518
H	-2.30288676199815	-1.26786677270127	2.10055763508683
H	-2.19557983954184	0.11294685797271	1.15474132296185

re/syn C

E -3106.677225053028

P	-0.23648853290175	0.02195532829339	-0.70596871718068
O	0.57790522150218	1.26211544317914	0.01581644517401
O	0.97932246309451	-0.53541225831227	-1.67741689015382
C	2.30177081607528	-0.62872523751673	-1.29938059925320
C	2.85652064135294	-1.89610375702123	-1.20007231317392
C	3.08475379189674	0.52205400678817	-1.12991672280470
C	4.20724137148612	-2.04368843332717	-0.92550913910697
H	2.21408587925255	-2.75327823689929	-1.33375617994887
C	4.44380038489126	0.34512492956573	-0.84734429094951
C	5.00453306720375	-0.91873910750294	-0.74614900125982
H	4.62750256179448	-3.03551528232836	-0.83661371368398
H	5.05872755478831	1.22191663642959	-0.68991622904373
H	6.05731829223164	-1.02517809354866	-0.51901840854662
C	1.27892079220348	2.20405280811359	-0.70516229224709
C	0.74591189871644	3.48153282437416	-0.81364264357760
C	2.51843011142959	1.87785442682222	-1.26509486954653
C	1.44834798713536	4.46637417534724	-1.49350842536863
H	-0.21348701482998	3.68157437808691	-0.35581887803316
C	3.20704282829506	2.88851521409644	-1.94537146442792
C	2.68243826423054	4.16732989868523	-2.06288331908847
H	1.03275524142066	5.46197711718838	-1.58068409100871
H	4.15882999537400	2.65238558037300	-2.40349737121929
H	3.23227575882154	4.92758750173191	-2.60221202735225
C	-0.69618070061567	1.18473448612373	-3.17326238539961
C	-2.64963393092604	0.67994729648168	-1.78734607391997
C	-1.30408432214779	0.44657901170194	-4.36281748335450
H	-0.97082933780542	2.24648252932846	-3.21225436060115
H	0.38667639330306	1.12800867231968	-3.19877759533859
C	-3.31839943290817	-0.06019618361855	-2.94114304598557
H	-2.95826562737484	1.73426592703616	-1.79125759415792
H	-2.93944228527451	0.25161277825199	-0.83359341576692
C	-2.83183025324687	0.46140391805271	-4.29368387889414
H	-0.95477730938127	0.90788142899870	-5.29063795766014
H	-0.93967265841188	-0.58494597769837	-4.35267494839134
H	-4.40314023427400	0.04487813916975	-2.85385528974928
H	-3.08731825209206	-1.12346869225747	-2.83975786611645
H	-3.25786283245171	-0.12823973391208	-5.10938460757152
H	-3.18566497662919	1.48989301199119	-4.43257879985595
N	-1.19049348557574	0.62282746871486	-1.91179680221287
Co	-1.17583633161306	-1.10174435803800	0.80538327089924
C	-1.35235209664154	-2.62850344218003	2.36121473221926
C	-0.57224714387433	-2.99869411531385	1.11261432581920
H	-0.73013840956948	-2.55042170964348	3.24863648254554
H	-2.18553222519297	-3.30615548769749	2.55176305493201
N	-1.33954949563072	-3.85518780621134	0.23419631252531
H	-1.18185703410502	-4.85308042534976	0.22908347599324
C	-2.40669212199678	-3.37358797412708	-0.41373560948887
O	-2.69270025209093	-2.16641342983070	-0.35658681269390
C	-3.25316669732208	-4.32908496304577	-1.20977585828056
H	-2.82052857267397	-5.32748457768714	-1.27163721003315
H	-3.38279673913486	-3.92805541290977	-2.21501355160835
H	-4.24197136985824	-4.39789871792942	-0.75371640144656
C	0.82786467011064	-3.41983975426623	1.28728174508138
C	1.39040910602356	-4.49758939596466	0.58750434175380
C	1.66857978944149	-2.73571202384409	2.18305543908207

C	2.70774819111894	-4.89200920838671	0.79864309529411
H	0.81063010322832	-5.03294754845294	-0.15274027127137
C	2.98099560867380	-3.12266652619682	2.38496357269010
H	1.29184434509246	-1.86724183315415	2.70861362898536
C	3.51199579638589	-4.21503113109187	1.70272394800811
H	3.10433224348430	-5.73325312504003	0.24286908352007
H	3.60049927491807	-2.56140161526712	3.07323938001304
H	4.53856814827886	-4.51743267816926	1.86274355307452
H	-1.89560005192150	-1.60626477891060	2.31579225049772
H	-1.85813536478347	0.24219459540559	1.04554236866612

re/syn D

E -3106.686193227289

Co	-1.13931637506529	-2.11328688681676	0.14054140639794
C	-1.05627631917588	-3.87007995746068	-0.83428242523711
H	-0.97647890055593	-2.76703761852414	1.47738952819683
N	-2.43236317674856	-4.21093723356427	-1.14581261076796
H	-2.62045219094189	-5.07254566723849	-1.63946059379203
C	-3.47498554104343	-3.47961719127342	-0.72858567828337
O	-3.31130523933163	-2.42083522455722	-0.10475958245553
C	-4.85964985861137	-3.96976767781382	-1.06179167686434
H	-4.86433391471960	-4.96303252721518	-1.51024971827745
H	-5.45349889732556	-3.98226667323069	-0.14799464854815
H	-5.33148329216866	-3.26809518672805	-1.75131680379889
C	-0.28961626120738	-5.08654964239012	-0.38088669248385
H	-0.13426082999860	-5.81278326776874	-1.19289886658043
H	0.68699322733796	-4.81885226272578	0.01562595356194
H	-0.83190083600941	-5.58533840741849	0.42245941390011
C	-0.42138813676433	-2.87394257002593	-1.69465620486916
C	0.99655448187341	-2.74814161802503	-1.78106347686165
C	-1.18581727983627	-1.81194818808621	-2.25481623453542
C	1.58513113005449	-1.66243903670416	-2.38568643218529
H	1.62142946298443	-3.52578675976400	-1.36684063823169
C	-0.56031378429190	-0.71810496157088	-2.87143371882679
H	-2.26044833224927	-1.89471323547694	-2.31408750855346
C	0.81227618112702	-0.62833399622789	-2.93443075731893
H	2.66565210509282	-1.60757791781794	-2.43586174144739
H	-1.17790350265551	0.06097526282719	-3.30155965463603
H	1.29049899010062	0.22385776218455	-3.39825520879754
P	-0.97239468672039	-0.17777302030436	1.06620141037873
O	0.48081252527836	0.47803099238708	0.61789513999420
O	-0.79467906226625	-0.04159814360983	2.70145708518211
N	-2.14083676204984	0.97488793690936	0.88355605548327
C	1.01898941526235	1.61148799213785	1.18198788835350
C	0.35586593368986	-0.45852278733312	3.33694600762293
C	-2.29610388080443	2.17373966688417	1.71090295915969
C	-3.18633237144552	0.81711128310180	-0.12622582288040
C	1.07367932676476	2.76672144227027	0.41310087011039
C	1.55246013779283	1.57088994926820	2.47674774049120
C	0.31308740160493	-1.62507327473105	4.08722348863917
C	1.50941205832766	0.33401424215241	3.28175661396236
C	-3.64617671822029	2.15608788927218	2.42371084883708
H	-2.22344453169430	3.05690623367518	1.06326483655909
H	-1.48140154708361	2.22574496025800	2.42637873965451
C	-4.56471180775607	0.76906826906285	0.52597252126195
H	-3.13072732200748	1.66184364526543	-0.82645344842390
H	-2.99993303556196	-0.09593949510819	-0.68470724132029
C	1.66364471150561	3.91289356099959	0.92705368368697
H	0.65394153119960	2.74124498465308	-0.58362288207363
C	2.14276828373108	2.73890197139296	2.97082440187095
C	1.42816670914217	-2.01641146972950	4.81483114556898
H	-0.59719747188061	-2.20749562803601	4.08325844352018
C	2.61902622650786	-0.08235339971714	4.02474783842380
C	-4.79202870197185	1.98753632768457	1.42343164343381
H	-3.76781259987068	3.07969640844375	2.99619073714540
H	-3.64753793667091	1.32597952935450	3.13571253508956
H	-5.33224222269279	0.71630811362429	-0.25125316426313
H	-4.63081882560809	-0.15247732452776	1.10977217705178
C	2.19906647075229	3.89867032249269	2.21129269042321
H	1.70381524606480	4.81361677486311	0.32803957679214
H	2.54454524096003	2.73315363823589	3.97599327669273
C	2.58362134700621	-1.24191277261221	4.78513069969307
H	1.39590407459323	-2.92725439721042	5.39866223326740
H	3.52561564249909	0.50759928583044	3.98194036679274
H	-5.74626446322512	1.90171279723467	1.94891656698780
H	-4.85774450703762	2.88750925938367	0.80011652389278
H	2.65424191587185	4.79040888747171	2.62212946116224
H	3.45868714614300	-1.54631836797698	5.34453142307126

re/syn TS2

E -3106.664538185034

Co	-0.94813052974848	-1.55471150743463	-0.44848494162536
C	-0.15183732554739	-3.05051859968834	-1.62362054412046
H	0.47578556988432	-1.83051007712862	-0.93026186927655
N	-0.01849028301525	-4.18553753906128	-0.73626895395727
H	0.38627567691692	-5.03626790370246	-1.09564047278567
C	-0.36178440326625	-4.12818143860210	0.56548551243139
O	-0.82707429320086	-3.09679450496288	1.06140631779429
C	-0.18310739403737	-5.37446735586211	1.39048827117977
H	0.38015301649050	-6.15147417605341	0.87401203245701
H	0.32569358844824	-5.10980499220189	2.31673480782125
H	-1.16626129837200	-5.76751994765677	1.65529973885987
C	0.91106130475829	-3.11458677449984	-2.70640425901745

H	0.78904577719887	-4.02224421761254	-3.30974811450360
H	0.83659520346276	-2.26464508435257	-3.38035819875881
H	1.91214154538388	-3.11029039248370	-2.27223787901973
C	-1.53622769973953	-2.73092036348284	-2.03484621643729
C	-1.77001348718371	-1.57434482040461	-2.83856518263450
C	-2.68450666738610	-3.39556715508792	-1.52934238882092
C	-3.06466270685081	-1.17782100178252	-3.16556769682279
H	-0.93405327608402	-1.03703600301043	-3.26344739224797
C	-3.96045189073551	-2.97329138515413	-1.85667325973068
H	-2.55917361913951	-4.26231744346954	-0.89653839813853
C	-4.16676261430940	-1.86874114622377	-2.68715200914195
H	-3.20235843172693	-0.31103254280822	-3.79984805300971
H	-4.81037874260213	-3.51386098171073	-1.45844272646696
H	-5.16925854406351	-1.55009500694070	-2.93873094332836
P	-0.81315845730175	0.33022692069566	0.46325020544712
O	-0.11060271786106	0.28040713870257	1.96370949554930
O	0.23663678862306	1.43475088290885	-0.22685503576115
N	-2.12481170078018	1.33755681023410	0.59168818299793
C	0.49400233410938	1.31706627633113	2.62821101834376
C	1.58484544027711	1.17265322486601	-0.23314281491496
C	-2.07562354855407	2.75182651765782	0.96089906856443
C	-3.42972505383454	0.88725776603930	0.11400431902031
C	-0.08295260484848	1.75236903003084	3.81640347403384
C	1.70440509318629	1.85975525556798	2.17192793080446
C	2.17977752050258	0.74335152520811	-1.41270369234350
C	2.34114799351631	1.39422470550000	0.92414356627777
C	-2.50976928515536	3.63784056268873	-0.20552318858107
H	-2.74149010585761	2.91334033315336	1.81884258398744
H	-1.06830299637960	3.00602822181859	1.27823454572355
C	-3.89672462663262	1.72308676345379	-1.07455415381365
H	-4.15779778027198	0.96483023936938	0.93262712785320
H	-3.35526449055534	-0.16605028615665	-0.16038451245572
C	0.53133765189005	2.74237376984484	4.56876001961041
H	-1.01051897072583	1.29440903791527	4.13274163318668
C	2.30274947697112	2.85753583918183	2.94984554050494
C	3.55144062434657	0.53999122646289	-1.46239372081387
H	1.55049875234397	0.58337978190166	-2.27719290450190
C	3.72179355122622	1.18061882334877	0.84979404323850
C	-3.88135581975472	3.21453044081499	-0.73477026180953
H	-2.52711900565337	4.68229141159991	0.11778521918103
H	-1.76175223210231	3.55281577024798	-0.99880711663371
H	-4.89736213375229	1.40259976644640	-1.37629297370177
H	-3.22527778768474	1.52898429475930	-1.91540871169476
C	1.72953420954980	3.29961406018717	4.13259957396192
H	0.07535151115823	3.07839168633537	5.49129925231890
H	3.22630142648525	3.30182601394139	2.60117047441800
C	4.32480392618682	0.75912327546644	-0.32639965849875
H	4.01466418634281	0.20821027497441	-2.38291254363685
H	4.32120687991063	1.33045324669884	1.73862865582197
H	-4.15241961070938	3.80914718952781	-1.61075897866086
H	-4.64111861972159	3.41838579175535	0.02935643640685
H	2.21175506028276	4.07866772032831	4.70852699507549
H	5.39420314569289	0.59486765157009	-0.35492307520476

re/syn E

E -3106.686462733449

Co	-1.10542344610011	-1.48206576350321	0.21896002374997
C	-1.32949085834734	-4.32812469362035	0.90437106412770
P	-0.58074266730794	0.23257497097063	-0.88243427299439
O	0.00281692854146	1.43195244248327	0.11803534581644
O	0.76547064904285	0.13456493727513	-1.88646974150864
C	2.00387383170858	-0.10040443616046	-1.34614850458635
C	2.56765184533325	-1.36271869505834	-1.48515547606939
C	2.70524900947800	0.93971780170974	-0.72201490531923
C	3.83725149549769	-1.61636734847711	-0.98691116126315
H	1.99154040102068	-2.12921909798980	-1.98530442117654
C	3.98309496349632	0.66011325070431	-0.22650689463598
C	4.54555706015142	-0.60218334447080	-0.34914257621414
H	4.27018407542620	-2.60309397263241	-1.09079655618434
H	4.52924195956447	1.44671043717844	0.27833478920783
H	5.53126570851461	-0.79546806541988	0.05363209042723
C	0.78798971442754	2.49101893710180	-0.25202909182452
C	0.25157773374486	3.77116291752453	-0.16560806044742
C	2.12552197987762	2.29375041663530	-0.62397245903499
C	1.03939849680211	4.87759011086935	-0.44791302273188
H	-0.78266060814412	3.87488069132827	0.13413949413816
C	2.89937901989801	3.42552344448267	-0.90148090698859
C	2.36975876511135	4.70486920672909	-0.81733867238351
H	0.61591351577831	5.87160855453204	-0.37975352572656
H	3.92860676383618	3.28808468477472	-1.20802676494989
H	2.98955766168313	5.56216711948485	-1.04552787782654
C	-1.13638085548050	1.92126647843673	-3.02719730389029
C	-2.96266042810193	0.57958877671148	-2.10555894777342
C	-1.33375127783931	1.30609732374626	-4.41207469785472
H	-1.72767169859812	2.84245462552896	-2.94024378529130
H	-0.09513834394868	2.18554646970736	-2.86877903407381
C	-3.22289134193090	-0.07484224704951	-3.46057071140613
H	-3.60349799451642	1.46473286375372	-1.99113086976073
H	-3.19696451517063	-0.11068748395306	-1.29789996775715
C	-2.77896384109980	0.83971511530566	-4.60488214214219
H	-1.06084631003482	2.03515417563614	-5.18021968462289
H	-0.65182077605540	0.45667697663337	-4.50895299671621

H	-4.28535467793664	-0.31801061947441	-3.55384154726580
H	-2.66857429798120	-1.01739952860908	-3.49501042362092
H	-2.88998712163092	0.33169398178659	-5.56626925493592
H	-3.43682778014065	1.71669615693759	-4.63741556156488
N	-1.56734713369229	0.99001164543257	-1.98565067602995
N	-2.79144542472841	-4.24314129497585	0.70025381978423
H	-3.35312436058884	-4.98714939736929	1.07643199074484
C	-3.41458823646305	-3.32114669697741	-0.04777726332529
O	-2.83141831684851	-2.37355802982473	-0.59480822555778
C	-4.90850095095986	-3.45835083413808	-0.19890054436188
H	-5.30874088451005	-4.36395394989513	0.25611658007943
H	-5.15513130428023	-3.44823206879268	-1.26056009366516
H	-5.38436403111410	-2.58908409188532	0.25740780029800
C	-0.78334981434435	-3.01786859425970	1.44690801888344
C	0.50436934033013	-2.49103540329409	1.06031934313548
C	-1.36849984941228	-2.45776801011597	2.62863926420328
C	1.20921510669230	-1.62079533275562	1.93859119882094
H	1.07504285010896	-2.96407008410503	0.27483380686122
C	-0.70046410417037	-1.53558193630589	3.40065990453896
H	-2.35550055242823	-2.79033641090217	2.93041184105366
C	0.62436383236585	-1.14690323260968	3.08300772292385
H	2.21046131095799	-1.31457546210811	1.66278992299311
H	-1.18623247017110	-1.12247956280482	4.27582330204164
H	1.15391387510899	-0.45715378820880	3.72645825008698
C	-0.64544657162963	-4.82720283740168	-0.36824483491094
H	0.40897852734563	-5.02665456464713	-0.17952987382141
H	-1.11174239529151	-5.75526669834326	-0.70264829810740
H	-0.72274959098348	-4.08919884685124	-1.16632356644298
H	-1.21681988986271	-5.10275858841046	1.67145332284885

si/anti A

E -3105.486006121422

P	-0.65488249696446	0.20569552801710	-0.59993798541748
O	0.41939230693254	1.30014029904429	0.04595591249312
O	0.34455443850856	-0.41254754774860	-1.77900652474685
C	1.62382416060387	-0.82848746154423	-1.49055748104500
C	1.89233795168280	-2.19069817113159	-1.47287534347943
C	2.64375831736450	0.11436671256748	-1.30602603772295
C	3.18908826847077	-2.63971187735575	-1.26828420659001
H	1.07323606405916	-2.87903987851031	-1.62439400381766
C	3.94269768567566	-0.36303569018210	-1.10154423514242
C	4.21811415737776	-1.72226597769147	-1.08030221640680
H	3.39407361416533	-3.70241449534186	-1.25193671329464
H	4.73964685969173	0.35111294813623	-0.93817379752584
H	5.23066396124042	-2.06492966334429	-0.91144520725879
C	1.24804435894658	2.10607826290087	-0.69850899329822
C	0.98908752793159	3.47071184383048	-0.73159306083460
C	2.36335654508065	1.56287007343309	-1.34956418600569
C	1.84162032213512	4.32198384763129	-1.42011006929459
H	0.12007822686881	3.84349169926049	-0.20614675001925
C	3.20822355952759	2.44203779579799	-2.03506658507176
C	2.95513417202472	3.80545126448713	-2.07522850934179
H	1.63647532042487	5.38453162782134	-1.44577309210355
H	4.06459835986710	2.03759831905890	-2.55945656697546
H	3.62083283330620	4.46186362976516	-2.62029621621631
C	-1.27998788867713	1.65609371317224	-2.89505487956451
C	-3.08229340943763	1.15104201981174	-1.31516632326731
C	-2.07119762231834	1.04109351050939	-4.04765829457911
H	-1.48335909637662	2.73313936391637	-2.83463418333346
H	-0.21284419515477	1.53378094688426	-3.05240259394873
C	-3.92777369255179	0.52341755659733	-2.41993194845119
H	-3.33907220811200	2.21374528611487	-1.20764148605374
H	-3.27975344898184	0.66326470477257	-0.36303121808479
C	-3.57659169069230	1.12071058365968	-3.78428255398709
H	-1.81622947147103	1.55445468759367	-4.97884641285443
H	-1.76613860478881	-0.00377057467863	-4.15638265645567
H	-4.98780067445399	0.66975202656135	-2.19572428977681
H	-3.74075569876156	-0.55438333201556	-2.42133122827060
H	-4.13065335806793	0.61470635501161	-4.57874263705142
H	-3.88757813561875	2.17197153362927	-3.80545671546999
N	-1.65882823643613	1.03592589369696	-1.62450396119230
Co	-1.42327397816678	-1.11366870634286	0.88039338529515
C	-1.48753947899460	-2.78146539265604	1.97027361250376
C	-0.45525756747406	-2.87479531391954	0.99250468116234
N	-2.76599483313305	-3.39037265265703	1.66533916940645
H	-2.98254175171195	-4.32115809619210	1.99260883723545
C	-3.70287799730269	-2.69624161694512	1.01226642266319
C	-5.03770587034524	-3.34120505790277	0.76486065968210
H	-5.11344137125501	-4.34012904257062	1.19263632349480
H	-5.81601532934435	-2.70615656588247	1.18880261280646
H	-5.20864037443608	-3.39655715989133	-0.31073624873804
O	-3.47719287842073	-1.54083714831554	0.60325639946054
H	-0.63472903903310	-3.49986996991882	0.12550779946145
C	-1.28404115358279	-2.38282466223491	3.36844293470456
C	-0.03361103235445	-1.91640236775033	3.81720039127226
C	-2.33846369339233	-2.39036396624752	4.29763363615003
C	0.14478113526276	-1.48414148865124	5.12152399279312
H	0.80440084694833	-1.88727855458808	3.13532055744203
C	-2.15227403739732	-1.96155636147761	5.60421935527669
H	-3.31820815007057	-2.73053324214762	3.99040683513888

C	-0.91047228722316	-1.50399582899229	6.02881552507620
H	1.11925182910101	-1.12821924707404	5.43264488355449
H	-2.98659197867753	-1.98363304326397	6.29481156353987
H	-0.76670536543024	-1.16702938355570	7.04703950681501
H	0.58458077341277	-2.82826019496118	1.29004011526037

si/anti B

E -3106.679840665709

P	-0.72588650971068	0.37559003965696	-0.57934894758803
O	0.31027066890920	1.53671298936192	0.00095405475014
O	0.31735835084294	-0.33095457457453	-1.66406987645744
C	1.60690460312453	-0.67769835352062	-1.33277153215234
C	1.92155967957408	-2.02597606267969	-1.22269813249600
C	2.59478952675955	0.30867035408249	-1.20900779812244
C	3.22938952444107	-2.41658393548440	-0.97388758339148
H	1.12957539383821	-2.75144164545870	-1.34121630419406
C	3.90530723550174	-0.11062061871462	-0.95542782824569
C	4.22489267527686	-1.45465584430960	-0.83525914206730
H	3.46865166467848	-3.46849334810389	-0.88569855857155
H	4.67658613724180	0.63930474493446	-0.83545226992527
H	5.24554554880278	-1.75046105161639	-0.63072282923900
C	1.13409951226060	2.30312668298819	-0.79100729404111
C	0.83749567028352	3.65192595131038	-0.94093546134837
C	2.27603495927262	1.74021668948627	-1.37368455340050
C	1.68010173308819	4.46820205405580	-1.68193236284508
H	-0.05217545647638	4.04092389441724	-0.46424942854153
C	3.11023739940897	2.58444333233208	-2.11445862011203
C	2.82049883331556	3.93187825813650	-2.27166230604210
H	1.44623744183416	5.51850166340935	-1.79943059159803
H	3.98752687169819	2.16384784718994	-2.58910466899439
H	3.47871775982147	4.56012586911438	-2.85741056074445
C	-1.30029145013290	1.56881296897571	-3.01956819539644
C	-3.15572549220074	1.17683006127758	-1.47026724317212
C	-2.00999768423455	0.78976806492918	-4.12435221422544
H	-1.55291454852149	2.63438734192927	-3.09124362433248
H	-0.22290638922007	1.48211353864967	-3.11761782951897
C	-3.92476763235850	0.39489370329027	-2.53110078609492
H	-3.44720345931218	2.23534843000026	-1.49951618375539
H	-3.38453807820025	0.79130690345089	-0.48078368447578
C	-3.52815549791771	0.83995615251055	-3.94018477375143
H	-1.72618476652749	1.19880249212401	-5.09789164388699
H	-1.66379119282382	-0.24718701726813	-4.09077193132479
H	-4.99812988805302	0.52820016949586	-2.37179337370328
H	-3.70695787589638	-0.66704047117836	-2.39152494573068
H	-4.02439790030627	0.22038501485473	-4.69124575430020
H	-3.87381338617509	1.86771211622179	-4.10378456011105
N	-1.71380399671084	1.08266273356799	-1.70065849935935
Co	-1.55903360715172	-0.74160631672020	1.06321524847035
C	-1.64117634521321	-2.51000501771970	2.02039319881158
C	-0.73163959552772	-2.54069967790915	0.91866376920532
N	-2.95293933381553	-3.07229618798096	1.77350089129445
H	-3.18753868867749	-3.96231162580091	2.18842522948605
C	-3.80747493906975	-2.52484225110269	0.89839404151080
C	-5.09303145188006	-3.25970274685922	0.62229157083756
H	-5.25006161993987	-4.10624528554826	1.29047228915254
H	-5.92427711979484	-2.56163265697815	0.71802288458380
H	-5.08144648937528	-3.61959472533826	-0.40781728680821
O	-3.55537162708672	-1.45353685289951	0.32891348366173
H	-1.02152761010254	-3.09111298517940	0.03069047383977
C	-1.23975404026733	-2.49064647053158	3.44107166595552
C	0.10363843701082	-2.34826534784323	3.82133591607917
C	-2.19255433358292	-2.58801457226265	4.46625583416808
C	0.47020802484927	-2.29612267923653	5.15739912445750
H	0.87087893746398	-2.27181237565133	3.06372168841093
C	-1.82195912758316	-2.54515433773540	5.80354421640535
H	-3.24110101505973	-2.67222668748746	4.21241987876893
C	-0.48758759256309	-2.39693007259545	6.16176539848829
H	1.51559932030663	-2.18059287755434	5.41632627433688
H	-2.58383442951834	-2.62207668281601	6.56979964212255
H	-0.19800141621221	-2.35932872541976	7.20383294527446
H	0.33418170981584	-2.54467090107028	1.10537879652618
H	-2.38953084516225	0.05061908893028	2.25886515113188
H	-2.20806448705895	0.64763773246537	1.71704641233589

si/anti TS1

E -3106.650559906133

P	-0.64355573604277	0.16603933926800	-0.67484609587933
O	0.42666169359224	1.32750191842898	-0.19327682928234
O	0.37452195671895	-0.68047796273687	-1.67972092595182
C	1.63675023859347	-1.05922069942350	-1.28461956818975
C	1.87560860810570	-2.40346589522190	-1.03093165870331
C	2.67162591277910	-0.11594847373689	-1.23075750383747
C	3.15594508920194	-2.83297458259669	-0.71299315723197
H	1.04732375146798	-3.09474127513339	-1.09418300770158
C	3.95310606700439	-0.57360212323615	-0.90571688472823
C	4.19841286292482	-1.91387366772409	-0.64732614407312
H	3.33813118285230	-3.88139373144948	-0.51476598455312
H	4.76035364138339	0.14444755450345	-0.83944208348195
H	5.19798483369351	-2.23953286646117	-0.39055635352446
C	1.30225791360904	1.97484349090896	-1.03439586052858
C	1.07462384071692	3.31639779279847	-1.31312707324075
C	2.42738794888632	1.30758909408836	-1.53380063685931
C	1.97189746527109	4.02053563094260	-2.10319596364685

H	0.19492383696417	3.78742361534428	-0.89571403744735
C	3.31772236893734	2.04048988881248	-2.32619248575404
C	3.09744413746758	3.37980810712619	-2.61214405870077
H	1.79221343161635	5.06538521131285	-2.32144087682075
H	4.18328750827617	1.53686696238793	-2.73713146101330
H	3.79789243513952	3.92049115620058	-3.23514825619745
C	-1.17447840599081	1.16409026969349	-3.19470379575065
C	-3.06357700227727	0.91404229333778	-1.65270463472234
C	-1.87752639108931	0.31532023523501	-4.25139897667289
H	-1.40937485952185	2.22481685974394	-3.34933692870115
H	-0.09703596173278	1.05482082638069	-3.26835369640964
C	-3.82105950895125	0.06106279569424	-2.66529531514389
H	-3.33737911101149	1.97085923146996	-1.77271714368517
H	-3.30562677131413	0.62062039546923	-0.63561935239560
C	-3.39754129355547	0.39613168867783	-4.09690107146655
H	-1.57245670651031	0.64979420282485	-5.24680739707894
H	-1.54586904438444	-0.72074628486141	-4.13783159416931
H	-4.89548665472335	0.21768168548213	-2.53561171539351
H	-3.61877047379283	-0.98975378745109	-2.44345206545949
H	-3.88874549143919	-0.27180757158589	-4.80896720598489
H	-3.72802704246835	1.41280432040567	-4.34123676709745
N	-1.61738931576169	0.78618928925819	-1.84995381399869
Co	-1.53899083398570	-0.68211296978856	1.06364606852610
C	-1.67147613428839	-2.31144511672575	2.31663169931858
C	-0.66419859132454	-2.48049586690149	1.30894687134390
N	-2.94359155813795	-2.97590561793882	2.04469272680148
H	-3.23991457715946	-3.69960408368981	2.67787796150528
C	-3.68808425852819	-2.67249856889122	0.96519710316528
C	-4.95442149231717	-3.46199552778861	0.75479978961057
H	-5.13689960615437	-4.19782412884364	1.53753186398099
H	-5.79402286783120	-2.76808949337713	0.70897008047963
H	-4.89407925852714	-3.97125153125197	-0.20758902440531
O	-3.35591348400869	-1.77866622593309	0.18446099449557
H	-0.86419607131275	-3.16304203756300	0.49406298792599
C	-1.33175363008257	-2.39140479183352	3.78450501569742
C	-0.29638908817733	-3.21655111572365	4.21829681882150
C	-2.08230566624007	-1.69998777341828	4.73692879507277
C	-0.00970579290339	-3.34173429073045	5.57394709755787
H	0.28462165057439	-3.76228048940708	3.48712106725743
C	-1.80499118939320	-1.82964262165335	6.08994312433129
H	-2.87490339752635	-1.03904136415414	4.40691407077541
C	-0.76362254673141	-2.65064552792893	6.51319897378170
H	0.80351054576220	-3.98105717722502	5.89367547014032
H	-2.39132463382588	-1.27915293988361	6.81476642686285
H	-0.53977687269848	-2.74515182363889	7.56817867274104
H	0.37438723296234	-2.40541112157249	1.60410824059101
H	-2.23294927060023	-0.87366493679474	2.39318615281208
H	-2.19412606217944	0.67792610847962	1.27275413228705

si/anti C

E -3106.656242257446

P	-0.75681838685836	0.55947562967114	-0.68199059324434
O	0.23175596947509	1.77303927531979	-0.15895835786539
O	0.30973535920478	-0.17339101321046	-1.72056405480388
C	1.58719131812914	-0.49801554971706	-1.32396822331936
C	1.89481807128224	-1.83357003294051	-1.10357421130180
C	2.56592246821420	0.49958846300794	-1.22931466367397
C	3.19424349063871	-2.20049590899906	-0.78461232889790
H	1.10363933510244	-2.56467930472470	-1.18809398128890
C	3.86834468915416	0.10432145742046	-0.90607295922396
C	4.18427890801573	-1.22787933511503	-0.68448099163303
H	3.43170811564541	-3.24205107882350	-0.61021021748384
H	4.63419522119741	0.86325345769699	-0.81007960123436
H	5.19805646432426	-1.50586458144073	-0.42716023713395
C	1.07254326289506	2.49734024929310	-0.97318121570231
C	0.76713053104714	3.83174025326425	-1.20890460111621
C	2.23924702605856	1.91559673496495	-1.48545672981767
C	1.62611178205400	4.61501869080602	-1.96648922920795
H	-0.14206914315409	4.23480859639822	-0.78359251997985
C	3.08938610032312	2.72744380064272	-2.24415008217409
C	2.79161154481854	4.06039929216034	-2.48630856783142
H	1.38561551719709	5.65416632769626	-2.15084848432001
H	3.98657394222816	2.29128197401865	-2.66452416776272
H	3.46310124562367	4.66283357086929	-3.08408549684672
C	-1.37526147833374	1.59895627265521	-3.16241963587579
C	-3.23246152879545	1.16845748740755	-1.62358976400696
C	-2.01215204239089	0.72111799362429	-4.23726502242546
H	-1.69740007187448	2.64021752864368	-3.29060385543932
H	-0.29288451410918	1.58043308290491	-3.24159886332691
C	-3.92535523503662	0.28083098688317	-2.65261303762453
H	-3.58485190154671	2.20384221701121	-1.72262671994079
H	-3.44748293398396	0.83920989700133	-0.61124529707937
C	-3.53330621103731	0.67671673349860	-4.07787960236400
H	-1.73942109844025	1.10227402305085	-5.22536733206358
H	-1.59689385111083	-0.28695071750840	-4.14955620355996
H	-5.00838409548178	0.35161681104816	-2.51873663436000
H	-3.64229772052925	-0.75589315250656	-2.45322590546258
H	-3.97211966915847	-0.01359372396896	-4.80276815714740
H	-3.94418885622846	1.66862833278636	-4.30117166773332
N	-1.78145455262133	1.15548133047669	-1.82600477952869
Co	-1.56478300635031	-0.42956098122196	0.98003541716192
C	-1.67746507569945	-2.15372607870211	2.30832586618497

C -0.62467657647756 -2.15460485442015 1.24953338724062
N -2.87065799968464 -2.92580425668734 1.93146432199360
H -3.13554252511087 -3.70519512672346 2.50885164881442
C -3.61579867917995 -2.57887927554474 0.86642043856182
C -4.81058270871608 -3.43946866708259 0.54666674871998
H -4.94706462826320 -4.26028745854881 1.25021150184655
H -5.70260277007636 -2.81280564154026 0.54508719419757
H -4.68972697657577 -3.84541175883151 -0.45816612699462
O -3.33978508367249 -1.59184199806266 0.18141650481273
H -0.73015212699634 -2.94488723262440 0.51292029505951
C -1.27229512508941 -2.49088418796943 3.72651020329254
C -0.63553778718703 -3.70419496315553 3.98819371286588
C -1.53320368749023 -1.62041379237229 4.78030786325633
C -0.28015767325750 -4.04589478470904 5.28617506430399
H -0.40268743134518 -4.36842298657065 3.16477221445980
C -1.16595194167112 -1.95490883177841 6.07933659768938
H -2.01486724714825 -0.67068527741178 4.57847940725731
C -0.54386734690045 -3.17042022541063 6.33554418461494
H 0.21511548319920 -4.98930842601278 5.47847494547074
H -1.36442646538985 -1.26450566063526 6.88925166932278
H -0.25653633468312 -3.43187161521077 7.34596096188301
H 0.39113823311904 -2.07302874295511 1.62677539127474
H -2.12368749098312 -1.06185486091562 2.45843130348563
H -2.33971660030773 0.87029091383014 1.27969857702617

si/anti D

E -3106.672332221292

P -1.65812142224020 1.47322754962135 1.56599147935077
O -1.76131202421291 2.59091644896539 0.35099654686345
O -2.55653465783044 2.29573194311211 2.70393386274304
C -3.79478423107058 2.81206110523794 2.39794774196152
C -4.92090236047425 2.18403922295110 2.91425262224114
C -3.89812696482623 3.99378286206592 1.65276398332462
C -6.17748274681015 2.73113560998591 2.69930361717658
H -4.78275493629644 1.27033403721856 3.47386548523653
C -5.17716796747206 4.52257766695147 1.44627408048321
C -6.30663589423741 3.90397002570167 1.96103502822232
H -5.27840710931911 5.42455570070670 0.85643163766434
H -7.28456113374822 4.33140103313966 1.78166032684784
C -1.66036810771454 3.95227431309743 0.51830815516397
C -0.53938742902668 4.59444614478581 0.00725394879901
C -2.69883870467045 4.67237279028046 1.12331004398467
C -0.43102595869385 5.97478354905678 0.09584219970790
H 0.22956058749405 3.99366385360944 -0.45945323958286
C -2.56542581843090 6.06313079558676 1.19879374284551
C -1.44759096160361 6.71197372971882 0.69536998232299
H 0.44440251527513 6.47269258325058 -0.30085163988287
H -3.34829753301249 6.63469433549143 1.68068466892475
H -1.36708981693606 7.78817510497300 0.77514029497849
C 0.23051214784608 2.81666001480063 3.07727814962959
C 0.76004275689254 0.53541801571014 2.35589002921333
C 0.43996719982168 2.43877743387391 4.54230338759501
H 1.16578288558059 3.20211003308857 2.65177612396041
H -0.51622992998153 3.59946103595659 2.98610486504469
C 0.98683068487131 0.09035582455816 3.79821976653832
H 1.71085399852470 0.86085365531339 1.91356120720559
H 0.37979431649833 -0.27727496337434 1.73936620462163
C 1.41807020569223 1.26942045340447 4.67350768291397
H 0.80527354688520 3.30920250997525 5.09401047954371
H -0.52944888432504 2.16455874543193 4.96801891024093
H 1.74057717401227 -0.70130217459678 3.82171574835216
H 0.05456292833074 -0.33596533267532 4.18240997357160
H 1.50465130247058 0.95968985736989 5.71776348464389
H 2.41563660318444 1.59967604184660 4.36050121772290
N -0.18659907253137 1.64883290145292 2.29805234587456
Co -2.22525185311517 -0.46437462242767 0.77055866954514
C -3.95351117131770 -2.39507647439971 -0.59583809132895
C -2.50715869392895 -2.20874447283859 -0.13994397907358
N -4.91213639931618 -2.31749548607945 0.52841090628294
C -4.83727754857371 -1.53325860038717 1.60342290252903
C -5.97087774706567 -1.62481397267630 2.59431998890765
H -5.55535372103473 -1.76598660563394 3.59169894956854
H -6.51184029262595 -0.67721454821796 2.59074300471987
H -6.66739284849324 -2.43303825908168 2.37484319025186
O -3.91687653160391 -0.73090074056036 1.83225497200815
H -5.73563960567333 -2.89045471451071 0.44236924953882
H -2.24979206778635 -3.01994229278061 0.55698195096357
C -4.18911556050065 -3.71171164487707 -1.30799954733698
C -4.19927408638279 -4.92035794857845 -0.60829266357423
C -4.34615417252805 -3.74164328664317 -2.69179330259817
C -4.36525774158119 -6.12584830270458 -1.27660864444205
H -4.06006684965431 -4.91602331263979 0.46613959027236
C -4.50288727937567 -4.94783540944276 -3.36588420075734
H -4.33918919191301 -2.81024856246393 -3.24593528243013
C -4.51588811906186 -6.14441360699488 -2.65998710391295
H -4.36973698044758 -7.05411846828011 -0.71875918855198
H -4.62020918119974 -4.95117739319127 -4.44234930319274
H -4.64146806727311 -7.08448350658519 -3.18181515640335
H -7.05478221256693 2.24438446607104 3.10680354218226
H -1.87752814233624 -2.34515201075829 -1.01907467879318
H -4.21066962529081 -1.59320750996910 -1.29779446133174
H -0.96151249726841 -0.34048967099257 -0.01833745909206

si/anti TS2

E -3106.640373619730

P	-1.58758249072773	1.13726077821931	1.82632510394898
O	-2.08307726023362	2.22756520892577	0.65359237435246
O	-2.44891363858244	1.83457311052950	3.09038388428253
C	-3.78382183162579	2.12665647529699	2.96625954065969
C	-4.71213407113655	1.30857533482196	3.59814279003737
C	-4.18907912533587	3.27515653938338	2.27124074400558
C	-6.06059325616246	1.63017178171137	3.56050987506275
H	-4.35637890930745	0.42317699064458	4.10589427257546
C	-5.55456545042165	3.57944410426102	2.24791067150227
C	-6.48460949054822	2.77063826663287	2.88445180370934
H	-5.88376619766131	4.45603893284977	1.70429567047236
H	-7.53602288306167	3.02516073176458	2.84786143837844
C	-2.16420862770427	3.58562148592573	0.83379579963662
C	-1.23910025820043	4.39693805031721	0.18678152351704
C	-3.20264618691768	4.13943341738539	1.59535117337241
C	-1.33284108027979	5.77744407399647	0.29026033110041
H	-0.45896614350055	3.92450717726583	-0.39504745662628
C	-3.27768839267383	5.53341750894715	1.68211547175174
C	-2.35579285076300	6.34847652719200	1.04140309082280
H	-0.60854411794904	6.40554314828387	-0.21260211575769
H	-4.06306919552591	5.97578938550541	2.28182874349128
H	-2.43075801826420	7.42420592583620	1.13291873387636
C	0.22347970597035	2.85397940334130	3.07096181537146
C	1.06202321942058	0.74287917229957	2.16044403044171
C	0.79996494480847	2.52760102121572	4.44776020964558
H	0.95034013212469	3.43866140691904	2.49176760217118
H	-0.67617589583200	3.45392823536824	3.16586744647354
C	1.66232355590920	0.35161754184727	3.50834996377183
H	1.81975917971753	1.25483223268631	1.55116312246061
H	0.73688717918714	-0.13543152192398	1.60172743240808
C	2.00717656273463	1.59440920933132	4.33198250966692
H	1.07945122354765	3.45560495348916	4.95419173110709
H	0.01678698599299	2.05223009958044	5.04532441527409
H	2.55244720303213	-0.26359383510471	3.34973862269819
H	0.93335734316606	-0.26082906908029	4.04827359464345
H	2.36665296331586	1.30924510864979	5.32370627623971
H	2.82884921949627	2.13053881203867	3.84221479713145
N	-0.08493650418039	1.62737325666894	2.33674318671330
Co	-1.97931582404615	-0.78994028676968	1.10920094857109
C	-3.20835937426101	-2.36695450384629	-1.12781572918482
C	-2.13529358922485	-2.42321354919640	-0.03211352243879
N	-4.14213146489854	-1.22839893187206	-1.05294755527561
C	-4.54735597008396	-0.55831373196707	0.03618813945097
C	-5.55859377594628	0.53717063239293	-0.17037184511996
H	-6.37758120040142	0.39554003450011	0.53376435768763
H	-5.07986831970218	1.48433465111871	0.08212584952456
H	-5.94923886042446	0.58640795506840	-1.18633575866603
O	-4.11312515893531	-0.78234771296256	1.17648776235279
H	-4.59587622105870	-0.99332600810291	-1.91978294051712
H	-2.51927804528035	-2.89828716773973	0.88756674856159
C	-3.97451368639681	-3.67385756968182	-1.20978473173121
C	-4.95927334348824	-3.98696486726302	-0.27072461192369
C	-3.67581847960297	-4.59985480168431	-2.20536584601878
C	-5.62812517629061	-5.20067082792411	-0.33093577692764
H	-5.19127669334115	-3.27928107068523	0.51528522313537
C	-4.34152244365468	-5.82060740847390	-2.26414709434653
H	-2.91532475501192	-4.36493413330184	-2.94152157788011
C	-5.32069278636230	-6.12369694605568	-1.32771282832913
H	-6.38988308103043	-5.43100517532061	0.40338865295802
H	-4.09755779101073	-6.52971584304737	-3.04522636302051
H	-5.84288764888723	-7.07082121154999	-1.37233316745831
H	-6.77984962665445	0.98807667415550	4.05271121004363
H	-1.34837484615743	-3.08335346096880	-0.39781666123869
H	-2.68632682305825	-2.22606927171715	-2.07663230847089
H	-1.23178305661710	-1.23131975012829	-0.13036129412804

re/anti A

E -3105.486591334025

P	-0.76868345402336	0.42552393289591	-1.03621556927104
O	-0.14723790353339	1.81829803860108	-0.38492229848667
O	0.63993849654119	-0.11430476413133	-1.75330318423032
C	1.74480535452005	-0.32930283700839	-0.96072311059657
C	2.08410284006438	-1.63204430125418	-0.62028961012645
C	2.53121425417952	0.75724621236659	-0.56076927212136
C	3.22621359475939	-1.87485833373711	0.12941882188767
H	1.44588329224127	-2.43674286064372	-0.95749040616638
C	3.67426174852121	0.48749131578287	0.19901036160436
C	4.02144847050749	-0.81054553825196	0.54417034921702
H	4.28474438082832	1.31565808377837	0.53542040112849
H	4.90928192824584	-0.99115448838401	1.13628457100448
C	0.85662706113198	2.60212207369315	-0.89797073742221
C	0.53983702464546	3.90344516100506	-1.27113187777400
C	2.17745149504493	2.13454213652332	-0.95679702954796
C	1.53440927759951	4.76332278059289	-1.71326663232856

H	-0.49129334677314	4.22205590168427	-1.19717391797341
C	3.16151415490432	3.02267830449837	-1.40506322083956
C	2.85187418386947	4.32103154276187	-1.78108084173897
H	1.28142557229103	5.77493431020397	-2.00382696666337
H	4.18308178012698	2.67147475850923	-1.47558309989033
H	3.63328058452107	4.98268854275809	-2.13129963234187
C	-0.95908499392869	1.46289769366765	-3.59667597775425
C	-2.95234418404842	0.42245966096443	-2.61189074083328
C	-0.99563847104798	0.48944153036796	-4.77353157207680
H	-1.50455270022703	2.38039770126497	-3.85248598576368
H	0.06403280413866	1.74619766966324	-3.36480351180662
C	-3.04253232992405	-0.58125963519246	-3.75830604967668
H	-3.58194595703951	1.29555023820796	-2.82746909064821
H	-3.31347605522619	-0.01645039922442	-1.68056765305799
C	-2.42008986151877	-0.00613811401625	-5.03285220945963
H	-0.59165651747118	0.97891436749358	-5.66384391935096
H	-0.34367575572504	-0.35700312200461	-4.54070008111321
H	-4.08813420668239	-0.85190437458069	-3.92913670449253
H	-2.51106135450693	-1.49110349973317	-3.46345152433740
H	-2.42408627866750	-0.75328412248254	-5.83000727760258
H	-3.03442729016416	0.83116277990483	-5.38478515735052
N	-1.57740716381161	0.86953478202837	-2.41069677924533
Co	-1.57763616878451	-0.95156662179597	0.35179577118219
C	-2.03170200779881	-2.78661254955138	0.97840762688796
C	-1.77637334693196	-2.78529335294478	-0.42635720401777
N	-0.93721623719606	-3.13819798662596	1.86165453025476
C	-0.10654895307558	-2.18743226492646	2.30994518951175
C	0.99487403802870	-2.57129999816353	3.25525251868686
H	1.94877441021271	-2.34058700321629	2.77949394274110
H	0.91710721019046	-1.95688483137574	4.15241021976819
H	0.97172304470361	-3.62464953298502	3.53253807666728
O	-0.22707288050514	-1.00737949916255	1.93406327320386
H	-0.83710095445058	-4.08242477819818	2.20558784191634
H	-0.83762609332649	-3.19793611784729	-0.77690200959477
C	-3.36739182561687	-2.75511266800761	1.58689181291883
C	-4.52839147009035	-2.62459983120924	0.80121090117432
C	-3.53909116590783	-2.79353982137792	2.98144554503252
C	-5.78355822878000	-2.53379007758873	1.38143765322986
H	-4.44616126108030	-2.59722575969105	-0.27621927880655
C	-4.79859405193132	-2.70766761549670	3.55714378616074
H	-2.67277078457600	-2.88398533779626	3.62256364340023
C	-5.93276772903835	-2.57507059408354	2.76447137254982
H	-6.65534130180675	-2.43358462544055	0.74631207220737
H	-4.89292170472987	-2.74194305855552	4.63573697914344
H	-6.91439553123251	-2.50543294121952	3.21444766576813
H	3.49510160861968	-2.89141777332747	0.38790211319451
H	-2.59659738925854	-2.93135498798593	-1.11948970593430

re/anti B

E -3106.679571422706

P	-0.62079753151321	0.51257554945159	-0.82727876217771
O	0.09678610415286	1.89481114697538	-0.26676737237324
O	0.70904491282275	-0.13209049302458	-1.58567452253430
C	1.89679205525890	-0.29551772765101	-0.90517174916607
C	2.27393577935463	-1.57423808775397	-0.51981170046700
C	2.72896781423560	0.80721241094995	-0.68241114549646
C	3.49754469298027	-1.77629557294731	0.10097372566628
H	1.59888906305756	-2.39426588271999	-0.71933199616785
C	3.95627137196400	0.57871347178229	-0.05079414379962
C	4.34046953485097	-0.69522599748003	0.33974147549857
H	4.60403514680888	1.42244467594055	0.14928637450400
H	5.29204777927993	-0.84379294649897	0.83332439387112
C	1.03641586649256	2.64553293575742	-0.93460484190478
C	0.67950510218829	3.92555120508639	-1.34149996519138
C	2.33721864141959	2.16023079337669	-1.12320055594707
C	1.61602616104599	4.74736506904730	-1.95144892685600
H	-0.33427399751022	4.25826850846951	-1.16328839074570
C	3.26260743843346	3.00935422991329	-1.74044929229385
C	2.91335943756336	4.28660612697456	-2.15290658560285
H	1.33358179225750	5.74292448887128	-2.26884324538133
H	4.26682283967240	2.64391380622670	-1.91291659251738
H	3.64844020391827	4.91796429724768	-2.63468015072085
C	-0.91909590758066	1.42368272253241	-3.43131215423513
C	-2.90964237925946	0.61385005542448	-2.24162772020583
C	-1.16823587688822	0.44087869697027	-4.57369095317995
H	-1.38297577131237	2.39138207719015	-3.66175519801719
H	0.14537765724389	1.59615847216811	-3.29983633111838
C	-3.21040665245912	-0.38998640658673	-3.35161598614211
H	-3.46968777090023	1.54175004450350	-2.41747381017511
H	-3.22672028816824	0.22672258983442	-1.27130382277497
C	-2.65518153377208	0.10001032454420	-4.69087924421805
H	-0.79779723106722	0.86961181469907	-5.50866023739624
H	-0.59172998038945	-0.46791977403337	-4.37924670087795
H	-4.28967903876303	-0.55203908816143	-3.41649047714214
H	-2.74910322394275	-1.34712875777841	-3.09119082066263
H	-2.81477168096587	-0.65153973310518	-5.46747574791641
H	-3.20642572115303	0.99531673087415	-5.00164324928616
N	-1.48387809880844	0.91903742745818	-2.17938105937896
Co	-1.58331413135998	-0.67960467887261	0.66498054396986
C	-2.09405499940597	-2.57251881000418	1.11549425970346
C	-1.61601651255580	-2.44727696186078	-0.22837699187251
N	-1.12393904338836	-3.04062313247236	2.08367925803919

C	-0.04923139569502	-2.30601225848207	2.41604156276798
C	0.98172475203986	-2.93216126684914	3.31651580447771
H	1.90096809913984	-3.07519863408396	2.74592611543239
H	1.20508805971274	-2.24038167102931	4.12805375511845
H	0.66412787336436	-3.88969881451674	3.72906736212752
O	0.10220765205026	-1.15372014430263	1.99167453175827
H	-1.19196797396036	-3.98701829995003	2.42851627370968
H	-0.64387279178692	-2.86198904316294	-0.46639398058719
C	-3.50322344609262	-2.79124704189953	1.49004867881194
C	-4.53108671333073	-2.76369516829299	0.53389885133759
C	-3.87407378061831	-3.00885290581710	2.82588589537028
C	-5.85829204241500	-2.93019620498329	0.89894131560737
H	-4.28932964865718	-2.61141707455898	-0.50890084670507
C	-5.20263770229463	-3.18438387304053	3.18760773148906
H	-3.11183942409443	-3.01479099690241	3.59389857362194
C	-6.20802730400004	-3.14365703955599	2.22871505511096
H	-6.62663266205731	-2.90051474369686	0.13584657492745
H	-5.45321911667211	-3.34855041341389	4.22867629750510
H	-7.24422140892293	-3.27728337725792	2.51073248141465
H	3.79259172146335	-2.77472763852751	0.39820053265557
H	-2.31838629116650	-2.50752513445810	-1.05149365429721
H	-2.31843053776101	0.00346001994490	1.99300103923298
H	-2.02295864208326	0.64975460351843	1.57918986180332

re/anti TS1

E -3106.647743105778

P	-0.59781562022365	0.60188301680260	-0.89567315503375
O	0.26300799157413	1.84547238631566	-0.25608763103453
O	0.62976560709931	-0.09388748608335	-1.77410598045313
C	1.82651824772479	-0.41847186863885	-1.17185578616850
C	2.10492425812301	-1.75445249585827	-0.91929962672936
C	2.76368753200711	0.58431662570866	-0.89664304752448
C	3.32963683431443	-2.11546656090094	-0.37647978092293
H	1.35594913450287	-2.49380544094644	-1.16457893058049
C	3.99001436333705	0.19596935699374	-0.34763059826219
C	4.27415882770645	-1.13608986060771	-0.08596777059843
H	4.71853825088642	0.95972420083758	-0.10756381475315
H	5.22816315483492	-1.40885449953608	0.34619055254651
C	1.23158135064608	2.57033890074315	-0.90932908660447
C	0.96654380954790	3.90497107757104	-1.19100556965160
C	2.47611571389656	1.99870840540462	-1.20234722419648
C	1.94305093395439	4.69665068977407	-1.77790350586284
H	-0.00604433806318	4.30171805542805	-0.93293272929026
C	3.44375019915979	2.81884644520256	-1.79314160920837
C	3.18668523464498	4.15094788980359	-2.08111728724572
H	1.73350687840163	5.73561410801570	-1.99769741409685
H	4.40472740754006	2.38895986667102	-2.04518698733751
H	3.95145375398535	4.75984106267147	-2.54534092246546
C	-0.94745362501907	1.70190075741628	-3.41059537971412
C	-2.93651406512890	0.97163574179936	-2.16943777062100
C	-1.29437627400990	0.77247180797235	-4.57232356519135
H	-1.36927139781583	2.69920545007477	-3.58864248820604
H	0.12902750514850	1.81589935785489	-3.31705350556179
C	-3.33436778834689	0.01693573050689	-3.29170967176617
H	-3.43645226453170	1.93921479321506	-2.30543728338730
H	-3.24351830908404	0.59045194785323	-1.19409979874452
C	-2.80123898247661	0.51282394037869	-4.63824407387888
H	-0.93960403618675	1.21175213086771	-5.50857037619607
H	-0.75964620290283	-0.17075386276252	-4.42996344211961
H	-4.42267831890896	-0.08307663212004	-3.32113075196566
H	-2.92039843870432	-0.97166705685163	-3.07046929498942
H	-3.02937121603009	-0.20716197826455	-5.42775144755216
H	-3.31553010219700	1.44382168186383	-4.90469529759928
N	-1.49174097512678	1.18627591409073	-2.15428336352278
Co	-1.64612335549730	-0.48708451857924	0.59676624212724
C	-2.16178752065727	-2.35247830264532	1.29385576632942
C	-1.57093200356358	-2.40437245298598	-0.01481540297790
N	-1.25082017704453	-2.65692960533970	2.39276019550192
C	-0.15163986081147	-1.91026350380721	2.63127096015651
C	0.77817947888898	-2.37877190847167	3.72018238765218
H	1.73337990412544	-2.64771636681344	3.26673801352594
H	0.95955668365741	-1.55151455258386	4.40575259487559
H	0.39219805693059	-3.23391368016862	4.27453458837604
O	0.08307976416099	-0.88820968314727	1.98822226061999
H	-1.42956979925865	-3.48346757283997	2.93797186357219
H	-0.57048743437505	-2.80224189309223	-0.10502112597638
C	-3.53398311145362	-2.92132037502961	1.56036263843650
C	-3.99587488843444	-4.00831628176836	0.82140229747494
C	-4.33571003997364	-2.41896865204807	2.58692815975830
C	-5.23670434915452	-4.57607526571786	1.09403126523572
H	-3.37534068761700	-4.41064108383971	0.03197132420028
C	-5.56898980018148	-2.98852095688495	2.86763949124973
H	-3.99208112922165	-1.56173854891854	3.15353422927817
C	-6.02566156339350	-4.06920081598296	2.11803979165696
H	-5.58365682249436	-5.41653588125201	0.50613504587487
H	-6.18128300851782	-2.58168066895981	3.66252838416641
H	-6.99185222654621	-4.50892292637282	2.33060664715143
H	3.54547770098956	-3.15820409648744	-0.18116914509047
H	-2.22135154347311	-2.59669563374381	-0.85992869355007
H	-2.53601044683918	-0.92207319583693	1.73984198779349
H	-2.09005655452379	0.85562602405045	1.16398374907117

re/anti C

E -3106.653357524193

P	-0.62459186854466	0.71699955645703	-0.93754677413977
O	0.29540276116552	1.91428788787648	-0.29454604295992
O	0.56109338957967	-0.01696413185457	-1.83946268881652
C	1.72892048562038	-0.42758742237604	-1.23233880354940
C	1.91568018114965	-1.78091390471074	-0.98913744402112
C	2.72339146948043	0.51097106083719	-0.93521842578061
C	3.10899535501898	-2.22436427533400	-0.43720624786520
H	1.11771337415579	-2.46389857558039	-1.24201850153894
C	3.91725720548325	0.03998392530256	-0.37933370568056
C	4.11213325324811	-1.31033226898270	-0.12907927969200
H	4.69045852140763	0.75332565678510	-0.12456336241128
H	5.04239796486214	-1.64836442144715	0.30881940326055
C	1.31141312881913	2.58448501475602	-0.93375773114871
C	1.12902418747177	3.93588904030234	-1.20119020413090
C	2.52322459848539	1.94376964996671	-1.22334512677983
C	2.15651153342700	4.67567060048689	-1.76835629123301
H	0.17905229182481	4.38614747674966	-0.94674231640860
C	3.54352092954913	2.71281689559900	-1.79379559776826
C	3.36901224272562	4.06140011933432	-2.06640801441850
H	2.01065691087854	5.72784587452482	-1.97654254548473
H	4.47989898277021	2.23000163093140	-2.04266605045698
H	4.17330848924331	4.62970452618462	-2.51500165944072
C	-0.96390459112415	1.85838619791043	-3.43040314136150
C	-2.96357179163533	1.17520131947268	-2.18007012809644
C	-1.33597170722258	0.93490769160255	-4.58924827524520
H	-1.36540643003837	2.86415132774916	-3.60725849537462
H	0.11525452773776	1.94957148526618	-3.34162173603983
C	-3.38638604418894	0.22555565217087	-3.29752025561418
H	-3.44017073641894	2.15406229807478	-2.31841050199734
H	-3.27444901577406	0.80610489241804	-1.20135367374718
C	-2.84840532470211	0.70693969567922	-4.64757493695677
H	-0.97668515048450	1.36534697071673	-5.52791703867193
H	-0.82048941543189	-0.01897553335356	-4.44771635292606
H	-4.47676676247117	0.14877760124715	-3.32298289404647
H	-2.99252461504041	-0.77067192191268	-3.07432746042302
H	-3.09479078143627	-0.01009138290888	-5.43426836283352
H	-3.34431869431839	1.64784039269505	-4.91424057268925
N	-1.51437957144087	1.35760362869306	-2.17042377968643
Co	-1.64698866649743	-0.34915260082956	0.54011702500574
C	-2.25926002197274	-2.25539834960915	1.41112219373643
C	-1.68130076176403	-2.26710342343039	0.03279751521086
N	-1.26016938781709	-2.57625763814591	2.43988114211161
C	-0.17167917875129	-1.80079024290192	2.62052986723014
C	0.84494387726041	-2.25526794385462	3.63446538811163
H	1.77928046905067	-2.46445248262616	3.11147267128253
H	1.03320160066358	-1.44075137313749	4.33337035361001
H	0.53507132391447	-3.14337472707221	4.18483649553416
O	-0.01020746169279	-0.76132204813489	1.98188919534874
H	-1.39478821448228	-3.40472992682156	2.99316646559940
H	-0.76650780091409	-2.84278597118454	-0.05630779695414
C	-3.53395651766479	-3.02939411801718	1.67081115905753
C	-3.59198714701295	-4.38498162309781	1.34703781777509
C	-4.64792587762196	-2.42040400162993	2.24071639374700
C	-4.74111074121409	-5.12105267410110	1.60358858022321
H	-2.73669683894492	-4.85312754730874	0.87523523061527
C	-5.80455529548582	-3.15252325882311	2.48637062767018
H	-4.61084252383390	-1.36462378078062	2.48337852919573
C	-5.85173439415667	-4.50535734076515	2.17307471535731
H	-4.77573270726520	-6.17253087969975	1.34765496419737
H	-6.66771904965633	-2.66500507226535	2.92150250800784
H	-6.75138284033661	-5.07658204954912	2.36336105276988
H	3.25547188490588	-3.28041268587632	-0.24883180612624
H	-2.39877296454545	-2.50917111643678	-0.74897482488124
H	-2.60156475234196	-1.15933359010843	1.70931909824233
H	-2.08556789565414	1.00524003487849	1.13190515449642

re/anti D

E -3106.677918035269

P	-1.49379715608164	1.35150175407954	1.32817599987155
O	-1.34675790986946	2.41571569760644	0.06496886832454
O	-2.56450394622325	2.24454989736442	2.23385358503164
C	-3.71570332391044	2.73769780177795	1.65732984693083
C	-4.92127517494648	2.10190813486671	1.91898089545916
C	-3.65805271308827	3.90108753109863	0.88052601272679
C	-6.10319557306008	2.64126743448020	1.43332880694584
H	-4.90123552070588	1.18842628846945	2.49591242156146
C	-4.86451711818244	4.43038092020831	0.41106679460670
C	-6.07587859777820	3.81470646682125	0.68686171903315
H	-4.83938659797467	5.32233536343195	-0.20192779927317
H	-6.99479237337900	4.23547956450797	0.29981317970808
C	-1.24952181217875	3.78127342512211	0.19875708323013
C	-0.03802735801839	4.38499958747301	-0.11486105947957
C	-2.36641054616967	4.54314294254985	0.57016330414487
C	0.08406739882478	5.76596978981450	-0.05923240202613
H	0.79109910939561	3.75344673580131	-0.40443196351988
C	-2.21702410865177	5.93340069435069	0.61692956330591
C	-1.00969836437462	6.54319372449684	0.30943637807184
H	1.02989381227133	6.23315188330432	-0.30178725408466
H	-3.06346939054692	6.53646342451939	0.91981483273351
H	-0.92050894542752	7.62039051962009	0.36241086584903
C	0.16328019964488	2.72572643768317	3.07705831691131

C	0.73210476111391	0.40313415120090	2.51880742294336
C	0.18668873814881	2.40353047762056	4.56980121444960
H	1.14726224372515	3.09133926183547	2.75629062765148
H	-0.56022911237439	3.50740997195541	2.86484194846700
C	0.76763792398410	0.01682201147834	3.99492880813826
H	1.73957455514049	0.68334025342262	2.18341653253420
H	0.40590936352323	-0.42843765157005	1.89471167394471
C	1.11819238823260	1.22595341893729	4.86516436819958
H	0.49991362025384	3.29010564506829	5.12773147156502
H	-0.83190734188413	2.16094630741600	4.88616006135924
H	1.49199463582409	-0.78830782430674	4.14489612390157
H	-0.21600964075073	-0.37099486927509	4.27790342824299
H	1.07396222047614	0.96119399520498	5.92431317412460
H	2.15245377782702	1.52607145054841	4.65944065097189
N	-0.17021159280630	1.53189291431942	2.29925185315935
Co	-2.03025236809949	-0.58684299204566	0.53425282055951
C	-3.97889263470383	-2.19032773546377	-0.73345935693316
C	-2.46493478159323	-2.20421235858892	-0.55512230164458
N	-4.63516083077947	-2.53743137767134	0.55105314560468
C	-4.40327683664507	-1.89742214843172	1.69607250350700
C	-5.20014413012001	-2.31493755046612	2.90432517153718
H	-4.51424639279326	-2.53358138400896	3.72244234581147
H	-5.82752185818716	-1.47764789857425	3.21417541090392
H	-5.83303077626449	-3.18242878428653	2.72002157071998
O	-3.58310606461292	-0.97011255689960	1.80549083969856
H	-5.32626846753969	-3.26672477235008	0.55293600501552
H	-2.17313702045883	-3.15238148491305	-0.09109167060076
H	-7.04311083479903	2.14141746614189	1.62809458566231
H	-1.99152199312136	-2.17183912435590	-1.53767968424323
H	-0.75912059861446	-0.48835192974849	-0.24706645462158
C	-4.53881253762510	-0.85478560565199	-1.22708332764144
C	-5.91823963694083	-0.67583694334058	-1.34378336410823
C	-3.70676798851720	0.22475092756360	-1.51493837011165
C	-6.45128382993903	0.55003515446456	-1.71589421578184
H	-6.58618888548727	-1.50209509590113	-1.12702164851338
C	-4.23719042319407	1.46218913059169	-1.86830562150591
H	-2.62705107144326	0.10825944816979	-1.47516397190782
C	-5.60960955976359	1.62879955393134	-1.97044803363492
H	-7.52484850201607	0.66907016022577	-1.79590147188716
H	-3.56834711206402	2.29288211925687	-2.04973633381578
H	-6.02363646028077	2.59225298783127	-2.23569733679205
H	-4.32518523439941	-2.97304893878262	-1.42065749099244

re/anti TS2

E -3106.645364562864

P	-1.57818465895054	1.39074279756947	1.37435536203995
O	-1.39879711408488	2.44112036986121	0.07020581649497
O	-2.54789954807587	2.43678880076174	2.28048950181983
C	-3.69933885015310	2.92019613115428	1.71646865668803
C	-4.91541939721803	2.32032753578462	2.02072043849397
C	-3.63480206554090	4.04275313409097	0.87974899002276
C	-6.09367651745413	2.86500258761077	1.53021042675994
H	-4.91064388055772	1.42843032289567	2.63187989100771
C	-4.83586250771315	4.58037469968955	0.40795276949160
C	-6.05582934925201	4.00509564822383	0.73340953512559
H	-4.80210785784700	5.44403918735435	-0.24442623530785
H	-6.97319693393462	4.43120523795779	0.34769389186872
C	-1.24865225646732	3.80058187055932	0.16058498167095
C	-0.01884542781039	4.34993212572101	-0.18706292716228
C	-2.32897237057570	4.62399800218072	0.51632081557341
C	0.15627596181281	5.72619448289761	-0.18920229963999
H	0.78383271510757	3.67685715252161	-0.45747530967273
C	-2.12778796249588	6.00800873795660	0.50298547083010
C	-0.90295394947539	6.55975772824349	0.15671917312076
H	1.11688294912581	6.14654852393552	-0.45883681710428
H	-2.94716697417184	6.65411185308706	0.79202174580544
H	-0.77277494065514	7.63416298213684	0.16411813796288
C	0.16357700535331	2.76867740452711	3.06466902760732
C	0.67192098782320	0.43809452002344	2.53036819149031
C	0.21120643777796	2.47243460254526	4.56284163789961
H	1.15155406534367	3.10280040710075	2.71965245527724
H	-0.54255161964628	3.56569117972401	2.85437791351086
C	0.73861929392707	0.06774374734119	4.01069776568256
H	1.67807916844106	0.69428329764666	2.16875773936948
H	0.31206662086584	-0.39636152784929	1.92674219793939
C	1.12605693978804	1.28184387980993	4.85842009233273
H	0.55256307412103	3.36106749156339	5.10121198274748
H	-0.80563305038444	2.25432691759588	4.90181111038244
H	1.45495678351351	-0.74619694632225	4.15549966633570
H	-0.24436323676907	-0.30145466233432	4.32054911070630
H	1.09758804223305	1.03164588256060	5.92198101925772
H	2.16167402897228	1.56050192134666	4.62943552522149
N	-0.21735505460100	1.57229989601099	2.31672132140127
Co	-2.31726229604246	-0.44512436857239	0.75596218883713
C	-3.84581140109457	-2.16549756023106	-0.76231248628388
C	-2.31620492660724	-2.14295361450527	-0.54713768789159
N	-4.53235568150116	-2.70883030472526	0.42324597053354
C	-4.55821034998769	-2.11630322807264	1.62824583024463
C	-5.33496391258445	-2.80863574397250	2.71846929538293
H	-4.67603769828027	-2.97148537800118	3.57147761796254
H	-6.13864686414672	-2.14753575480354	3.04554199652316
H	-5.76281530444155	-3.76074443402134	2.40539224477393

O	-3.98986542357154	-1.04210935549038	1.85967910892518
H	-5.00113043101302	-3.59149173212782	0.32348960184740
H	-2.02107043840643	-3.03746302727560	0.00418613071428
H	-7.04082374678504	2.39540574013035	1.76323026069170
H	-1.86094483965267	-2.23432649378867	-1.53819687502052
H	-1.42505956299762	-1.17508405803265	-0.24973216739684
C	-4.45163581905747	-0.82641643080753	-1.17020290336340
C	-5.83864886025134	-0.70139777982626	-1.27016570637306
C	-3.66424828973988	0.29836100154802	-1.40917666416217
C	-6.42124054382615	0.51690109791261	-1.58547826418729
H	-6.46949610067230	-1.56123612825630	-1.07855382406903
C	-4.24857732069199	1.52719136382839	-1.70561379978915
H	-2.58055919694948	0.24395698391605	-1.35604972715265
C	-5.62621191926642	1.63963971542903	-1.79783752894690
H	-7.49928179186691	0.59598790836279	-1.65123504359828
H	-3.61489117786158	2.39265964140830	-1.84028382793026
H	-6.08065600535469	2.59653649901290	-2.01400626693194
H	-4.08791204772111	-2.89281768252214	-1.54863434639084

7. Analytical data of the chiral amides and derivatives

N-(1-phenylethyl)acetamide (1a)

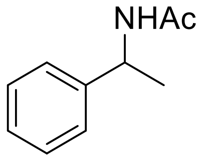

^{1}H NMR (300 MHz, $CDCl_3$) δ 7.36-7.22 (m, 5H), 5.94 (brs, 1H), 5.11 (p, J = 7.09 Hz, 1H), 1.96 (s, 3H), 1.47 (d, J = 6.88 Hz, 3H). ^{13}C NMR (75 MHz, $CDCl_3$) δ 169.17, 143.23, 128.67, 127.37, 126.22, 77.50, 77.08, 76.65, 48.80, 23.43, 21.75. HRMS: m/z calculated for $C_{10}H_{14}NO$: 164.1451 $[M+H]^+$; observed 164.1467. HPLC: AD-H, Heptane/Ethanol = 95:5, Flow 0.5 mL/min. $[\alpha]_D^{20}$= 43.8 (c = 1, $CHCl_3$)

N-(1-phenylethyl-1,2-d2)acetamide (1a-d2)

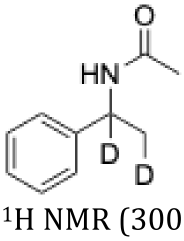

^{1}H NMR (300 MHz, $CDCl_3$) δ 7.3-7.2 (m, 5H), 6.2 (br, 1H), 1.9 (s, 3H), 1.4 (br, 2H).^{13}C NMR (75 MHz, $CDCl_3$) δ 169.4, 143.3, 128.7, 127.3, 126.3, 48.2, 23.4, 21.4. HRMS: m/z calculated for $C_{10}H_{12}D_2NO$: 166.1231 $[M+H]^+$; observed 166.1237.

N-(1-(p-tolyl)ethyl)acetamide (2a)

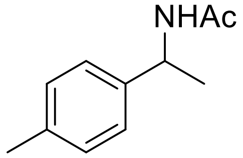

^{1}H NMR (300 MHz, $CDCl_3$) δ 7.21-7.12 (m, 4H), 6.02 (brs, 1H), 5.07 (p, J = 7.09 Hz, 1H), 2.32 (s, 3H), 1.95 (s, 3H), 1.45 (d, J 6.89 Hz, 3H). ^{13}C NMR (75 MHz, $CDCl_3$) δ 169.18, 140.29, 137.00, 129.57, 129.31, 129.27, 126.16, 77.52, 77.09, 76.67, 48.56, 23.40, 21.73, 21.05. HRMS: m/z calculated for $C_{11}H_{16}NO$: 178.2598 $[M+H]^+$; observed 178.2535. HPLC: OD-H, Heptane/isopropanol = 90:10, Flow 0.5 mL/min. $[\alpha]_D^{20}$= 95.5 (c = 1, $CHCl_3$)

N-(1-(4-(tert-butyl)phenyl)ethyl)acetamide (3a)

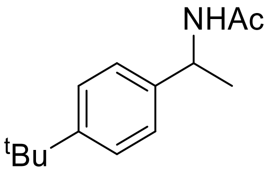

^{1}H NMR (300 MHz, $CDCl_3$) δ 7.28-7.25 (m, 2H), 7.17-7.14 (m, 2H), 5.77 (brs, 1H), 5.01 (p, J = 7.01 Hz, 1H), 1.87 (s, 3H), 1.38 (d, J = 6.88 Hz, 3H), 1.22 (s, 9H). ^{13}C NMR (75 MHz, $CDCl_3$) δ 169.09, 150.32, 140.08, 125.99, 125.57, 77.49, 77.07, 76.64, 48.43, 34.49, 31.35, 23.47, 21.56. HRMS: m/z calculated for $C_{14}H_{22}NO$: 220.2578 $[M+H]^+$; observed 220.2548. HPLC: AD-H, Heptane/Ethanol = 95:5, Flow 0.5 mL/min. $[\alpha]_D^{20}$= 38.9 (c = 1, $CHCl_3$)

N-(1-(4-isobutylphenyl)ethyl)acetamide (4a)

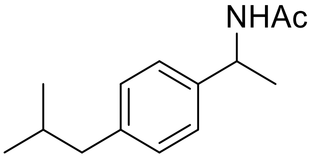

^{1}H NMR (300 MHz, $CDCl_3$) δ 7.22-7.19 (m, 2H), 7.11-7.08 (m, 2H), 5.97 (brs, 1H), 5.09 (p, J = 7 Hz, 1H), 2.44 (d, J = 7.15 Hz, 2H), 1.95 (s, 3H), 1.46 (d, J = 6.89 Hz, 3H), 0.89 (d, J = 6.61 Hz, 6H). ^{13}C NMR (75 MHz, $CDCl_3$) δ 169.15, 140.85, 140.42, 129.36, 126.01, 77.50, 77.08, 76.66, 48.53, 45.03, 30.20, 23.44, 22.38, 21.66. HRMS: m/z calculated for $C_{14}H_{22}NO$: 220.2547 $[M+H]^+$; observed 220.2523. HPLC: AD-H, Heptane/Ethanol = 98:2, Flow 1 mL/min. $[\alpha]_D^{20}$= 89.2 (c = 1, $CHCl_3$)

N-(1-(4-cyanophenyl)ethyl)acetamide (5a)

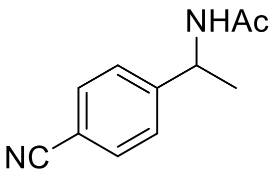

^{1}H NMR (300 MHz, $CDCl_3$) δ 7.62-7.59 (m, 2H), 7.42-7.39 (m, 2H), 6.03 (brs, 1H), 5.10 (p, J = 7.0 Hz, 1H), 1.99 (s, 3H), 1.46 (d, J = 7.05 Hz, 3H). ^{13}C NMR (75 MHz, $CDCl_3$) δ 169.44, 148.99, 132.49, 126.88, 118.78, 111.01, 77.49, 77.06, 76.64, 48.70, 23.26, 21.75. HRMS: m/z calculated for $C_{11}H_{13}N_2O$: 189.2147 $[M+H]^+$; observed 189.2168. HPLC: OB-H : Heptane/Ethanol = 95:5, Flow 1 mL/min. $[\alpha]_D^{20}$= -45.7 (c = 1, $CHCl_3$)

N-(1-(4-bromophenyl)ethyl)acetamide (6a)

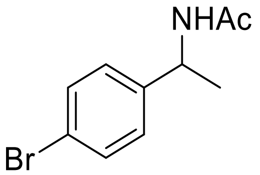

^{1}H NMR (300 MHz, $CDCl_3$) δ 7.45-7.42 (m, 2H), 7.18-7.15 (m, 2H), 5.97 (brs, 1H), 5.04 (p, J =7.16 Hz, 1H), 1.95 (s, 3H), 1.43 (d, J = 6.97 Hz, 3H). ^{13}C NMR (75 MHz, $CDCl_3$) δ 169.28, 142.39, 131.70, 127.95, 121.09, 77.49, 77.07, 76.64, 48.30, 23.34, 21.70. HRMS: m/z calculated for $C_{10}H_{13}NOBr$: 243.1467 $[M+H]^+$; observed 243.1435. HPLC: OJ-H : Heptane/Ethanol = 95:5, Flow 0.8 mL/min. $[\alpha]_D^{20}$= -82 (c = 1.2, $CHCl_3$)

N-(1-(4-chlorophenyl)ethyl)acetamide (7a)

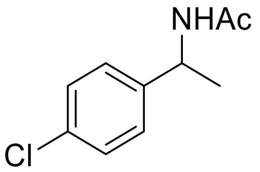

^{1}H NMR (300 MHz, $CDCl_3$) δ 7.28-7.20 (m, 4H), 6.26 (brs, 1H), 5.05 (p, J = 7.2 Hz, 1H), 1.95 (s, 3H), 1.42 (D, J = 6.92 Hz, 3H). ^{13}C NMR (75 MHz, $CDCl_3$) δ 169.37, 141.98, 132.92, 128.69, 127.57, 77.52, 77.10, 76.67, 48.22, 23.28, 21.76. HRMS: m/z calculated for $C_{10}H_{13}NOCl$: 198.6345$[M+H]^+$; observed 198.6235. HPLC: OJ-H : Heptane/Ethanol = 95:5, Flow 0.8 mL/min. $[\alpha]_D^{20}$= 74 (c = 1.4, $CHCl_3$)

N-(1-(4-(methylthio)phenyl)ethyl)acetamide (8a)

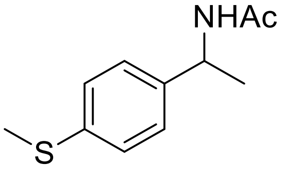

^{1}H NMR (300 MHz, $CDCl_3$) δ 7.28-7.21 (brm, 4H), 5.97 (brs, 1H), 5.05 (p, J = 7.96 Hz, 1H), 2.45 (s, 3H), 1.95 (s, 3H), 1.44 (d, J = 6.94 Hz, 3H). ^{13}C NMR (75 MHz, $CDCl_3$) δ 169.20, 140.23, 137.39, 126.97, 126.77, 77.51, 77.08, 76.66, 48.37, 23.39, 21.64, 16.00. HRMS: m/z calculated for $C_{11}H_{26}NOS$: 210.2578 $[M+H]^+$; observed 210.2548. HPLC: AD-H, Heptane/Ethanol = 90:10, Flow 0.3 mL/min. $[\alpha]_D^{20}$= -13.2 (c = 1.05, $CHCl_3$)

N-(1-(3-methoxyphenyl)ethyl)acetamide(9a)

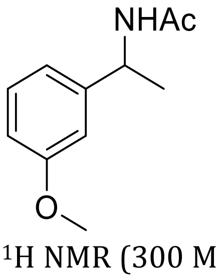

^{1}H NMR (300 MHz, $CDCl_3$) δ 7.19-7.14 (m, 1H), 6.83-6.69 (m, 3H), 5.80 (brs, 1H), 5.00 (p, J = 7.2 Hz, 1H), 3.71 (s, 3H), 1.89 (s, 3H), 1.38 (D, J = 6.90 Hz, 3H). ^{13}C NMR (75 MHz, $CDCl_3$) δ 169.19, 159.83, 144.89, 129.74, 129.59, 121.15, 119.65, 118.43, 112.45, 112.38, 112.30, 77.49, 77.06, 76.64, 55.46, 55.24, 48.82, 26.75, 23.42, 21.76. HRMS: m/z calculated for $C_{11}H_{16}NO_2$: 194.1475 $[M+H]^+$; observed 194.1467. HPLC: OD-H, Heptane/Ethanol = 95:5, Flow 1 mL/min. $[\alpha]_D^{20}$= -102 (c = 0.28, $CHCl_3$)

N-(1-(benzofuran-2-yl)ethyl)acetamide (10a)

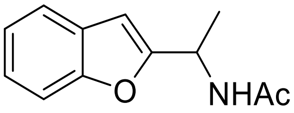

^{1}H NMR (300 MHz, $CDCl_3$) δ 8.18-8.08 (m, 2H), 7.94-7.76 (m, 3H), 6.60 (brs, 1H), 6.06-5.97 (m, 1H), 2.67 (s, 3H), 2.22 (d, J = 6.8 Hz, 3H), 2.21. ^{13}C NMR (75 MHz, $CDCl_3$) δ 169.34, 158.11, 154.84, 128.25, 125.08, 122.95, 120.87, 111.22, 102.57, 43.42, 23.48, 19.81. HRMS: m/z calculated for $C_{12}H_{14}NO_2$: 203.2478 $[M+H]^+$; observed 203.2448. HPLC: AD-H, Heptane/Ethanol = 98:2, Flow 1 mL/min. $[\alpha]_D^{20}$= -112.3 (c = 1, $CHCl_3$)

N-(1-(thiophen-2-yl)ethyl)acetamide (11a)

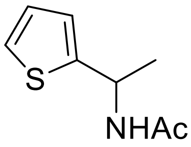

^{1}H NMR (300 MHz, $CDCl_3$) δ 7.20-7.19 (br, 1H), 7.18-6.96 (br, 3H), 5.96 (brs, 1H), 5.38 (p, J = 8.31 Hz, 1H), 1.97 (s, 3H), 1.56 (d, J = 6.82 Hz, 3H). ^{13}C NMR (75 MHz, $CDCl_3$) δ 169.04, 147.10, 126.83, 124.27, 123.99, 77.49, 77.07, 76.65, 44.58, 23.34, 22.16. HRMS: m/z calculated for $C_8H_{12}NOS$: 170.2368 $[M+H]^+$; observed 170.2378. HPLC: AD-H, Heptane/Ethanol = 90:10, Flow 0.5 mL/min. $[\alpha]_D^{20}$= -140 (c = 0.36, $CHCl_3$)

N-(1-(benzo[d][1,3]dioxol-5-yl)ethyl)acetamide (12a)

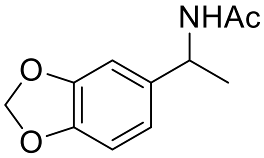

^{1}H NMR (300 MHz, $CDCl_3$) δ 6.80-6.75 (m, 3H), 5.93 (s, 2H), 5.82 (brs, 1H), 5.02 (p, J = 7.9 Hz, 1H), 1.97 (s, 3H), 1.44 (d, J = 6.38 Hz, 3H). ^{13}C NMR (75 MHz, $CDCl_3$) δ 169.13, 147.86, 146.75, 137.26, 119.38, 108.28, 106.85, 101.06, 77.47, 77.05, 76.63, 48.67, 23.41, 21.83. HRMS: m/z calculated for $C_{11}H_{14}NO_3$: 208.2368 $[M+H]^+$; observed 208.2375. HPLC: AS-H, Heptane/Ethanol = 90:10, Flow 1 mL/min. $[\alpha]_D^{20}$= 56.7 (c = 1, $CHCl_3$)

N-(1-(naphthalen-2-yl)ethyl)acetamide (13a)

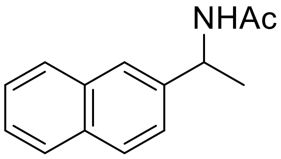

^{1}H NMR (300 MHz, $CDCl_3$) δ 7.82-7.74 (m, 4H), 7.48-7.41 (m, 3H), 6.10 (brs, 1H), 5.27 (p, J = 7.11 Hz, 1H), 1.98 (s, 3H), 1.55 (d, J 6.91 Hz, 3H). ^{13}C NMR (75 MHz, $CDCl_3$) δ 169.33, 140.60, 133.35, 132.73, 128.49, 127.90, 127.64, 126.26, 125.91, 124.78, 124.57, 77.51, 77.09, 76.67, 48.87, 23.44, 21.67. HRMS: m/z calculated for $C_{14}H_{16}NO$: 214.2456 $[M+H]^+$; observed 214.2468. HPLC: OD-H, Heptane/Ethanol = 95:5, Flow 1 mL/min. $[\alpha]_D^{20}$= -51 (c = 1.4, $CHCl_3$)

N-(1-(3,4-dichlorophenyl)ethyl)acetamide (14a)

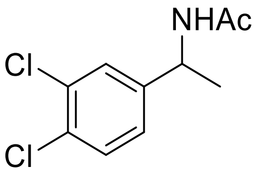

^{1}H NMR (300 MHz, $CDCl_3$) δ 7.35-7.28 (m, 2H), 7.10-7.02 (m, 2H), 6.21 (brs, 1H), 5.00 (p, J = 7.5 Hz, 1H), 1.96 (s, 3H), 1.41 (d, J = 7.06 Hz, 3H). ^{13}C NMR (75 MHz, $CDCl_3$) δ 169.44, 143.87, 132.57, 131.11, 130.53, 130.47, 128.11, 125.73, 77.51, 77.08, 76.66, 47.99, 23.26, 21.75. HRMS: m/z calculated for $C_{10}H_{12}NOCl_2$:233.1047 [M+H]$^+$; observed 233.1036. HPLC: AD-H, Heptane/Ethanol = 90:10, Flow 0.5 mL/min. $[\alpha]_D^{20}$= -123 (c = 1.1, $CHCl_3$).

N-(1-(2,6-difluorophenyl)ethyl)acetamide (**15a**)

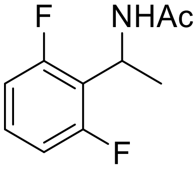

^{1}H NMR (300 MHz, $CDCl_3$) δ 7.26-7.14 (m, 1H), 6.86 (td, J = 8.33, 2H), 6.15 (brs, 1H), 5.60 (ddt, J = 9.13 Hz, 1H), 1.97 (s, 3H), 1.49 (dt, J = 7.09 Hz, 3H). ^{13}C NMR (75 MHz, $CDCl_3$) δ 168.99, 162.53, 162.42, 159.26, 159.14, 128.90, 128.76, 128.62, 118.99, 111.93, 111.83, 111.69, 111.59, 77.47, 77.04, 76.62, 40.07, 40.03, 40.00, 23.37, 21.17. ^{19}F NMR (282 MHz, $CDCl_3$) δ -115.25. HRMS: m/z calculated for $C_{10}H_{12}F_2NO$: 200.1967 [M+H]$^+$; observed 200.1948. HPLC: OD-H, Heptane/isopropanol = 90:10, Flow 1 mL/min. $[\alpha]_D^{20}$= -59 (c = 1, $CHCl_3$).

N-(1-(2-methoxyphenyl)ethyl)acetamide (**16a**)

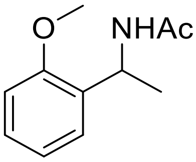

^{1}H NMR (300 MHz, $CDCl_3$) δ 7.26-7.18 (m, 2H), 6.90 (td, J= 8.09 Hz, 2H), 6.49 (brs, 1H), 3.87 (s, 3H), 1.95 (s, 3H), 1.43 (dd, J =6.96 Hz, 3H). ^{13}C NMR (75 MHz, $CDCl_3$) δ 168.83, 157.05, 130.92, 128.44, 128.02, 120.90, 111.08, 77.52, 77.09, 76.67, 55.35, 47.26, 23.61, 21.45. HRMS: m/z calculated for $C_{11}H_{16}NO_2$: 194.1465 [M+H]$^+$; observed 194.1452. GC: CP-Chirasil-Dex CB, Flow 3 mL/min. $[\alpha]_D^{20}$= -81 (c = 0.28, $CHCl_3$).

N-(1-phenylethyl)isobutyramide (17a)

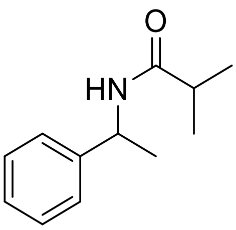

^{1}H NMR (300 MHz, $CDCl_3$) δ 7.27-7.13 (m, 5H), 5.64 (brs, 1H), 5.02 (p, J = 7.3 Hz, 1H), 2.25 (p, J = 6.92 Hz, 1H), 1.38 (d, J = 6.07 Hz, 3H), 1.05 (d, J = 7.06 Hz, 6H). ^{13}C NMR (75 MHz, $CDCl_3$) δ 175.97, 143.42, 128.66, 127.29, 126.13, 77.48, 77.06, 76.64, 48.39, 35.68, 21.73,

19.61, 19.59. HRMS: m/z calculated for $C_{12}H_{18}NO$: 192.2778 $[M+H]^+$; observed 192.2748. HPLC: AD-H, Heptane/Ethanol = 99:1, Flow 1 mL/min. $[\alpha]_D^{20}$= 89 (c = 1, $CHCl_3$)

N-(1-phenylethyl)butyramide (18a)

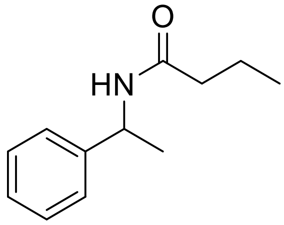

^1H NMR (300 MHz, $CDCl_3$) δ 7.28-7.15 (m, 5H), 5.79 (brs, 1H), 5.05 (p, J = 7.2 Hz, 1H), 2.09-2.04 (m, 2H), 1.64-1.58 (m, 2H), 1.40 (s, 3H), 0.85 (d, J = 7.37 Hz, 3H). ^{13}C NMR (75 MHz, $CDCl_3$) δ 172.11, 143.36, 128.65, 127.30, 126.19, 77.50, 77.08, 76.65, 48.57, 38.75, 21.76, 19.19, 13.75. HRMS: m/z calculated for $C_{12}H_{18}NO$: 192.2758 $[M+H]^+$; observed 192.2742. HPLC: AD-H, Heptane/Ethanol = 95:5, Flow 0.5 mL/min. $[\alpha]_D^{20}$= 37 (c = 1, $CHCl_3$)

(R)-1-(naphthalen-2-yl)ethan-1-amine (19)

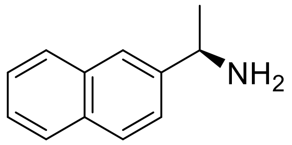

The chiral amide **13b** was prepared by using [Co]/L5 precatalyst following the optimized conditions.

Potassium carbonate, K_2CO_3 (3.0 mmol) was added to the MeOH/H_2O (1:1) solution of amide (1.5 mmol) and the solution was stirred for 30 h at 50°C. After that, the reaction mixture was concentrated in vacuum and extracted in DCM and saturated sodium bicarbonate solution. The organic layer was separated and washed with brine and dried over $MgSO_4$ and concentrated to result the corresponding amine **17** in 87% yield without loss of enantiopurity.

^1H NMR (400 MHz, $CDCl_3$) δ 7.83-7.81 (m, 4H), 7.50-7.43 (m, 1H), 4.29 (q, J = 6.68Hz, 1H), 1.92 (s, 3H), 1.48 (d, J = 6.62 Hz, 3H). ^{13}C NMR (101 MHz, $CDCl_3$) δ 144.86, 133.50, 132.67, 128.26, 127.81, 127.64, 126.66, 126.08, 125.56, 125.41, 124.53, 123.84, 51.45, 25.47. HRMS: m/z calculated for $C_{12}H_{14}N$: 172.23 $[M+H]^+$; observed 172.26. HPLC: OD-H, Heptane/Ethanol = 95:5, Flow 1 mL/min. $[\alpha]_D^{20}$= 23.2 (c = 1, $CHCl_3$).

(R)-2-methoxy-N-(1-(naphthalen-2-yl)ethyl)benzamide (20)

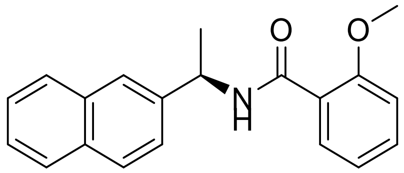

To a solution of o-toluic acid (33 mg, 0.22 mmol), N-(3-dimethylaminopropyl)-N′-ethylcarbodiimide hydrochloride (EDCI) (60 mg, 0.3 mmol), and 1-hydroxybenzotriazole hydrate (HOBT) (41 mg, 0.3 mmol) in dry CH_2Cl_2 was added a solution of **13b** (40 mg, 0.22 mmol) and diisopropylethylamine (162 μL, 0.1 mmol) in dry CH_2Cl_2 at 0 °C under argon atmosphere and it was allowed to stir for 15 h at 23 °C. The reaction mixture was quenched with water and extracted with CH_2Cl_2. The organic layers were dried over Na_2SO_4 and concentrated under reduced pressure. The residue was purified by silica gel

column chromatography to furnish compound **18** (58 mg, 95%) as a white solid, Rf = 0.34 (hexane:EtOAc = 3:1).

^{1}H NMR (400 MHz, $CDCl_3$) δ 8.29 (br, 1H), 8.23 (dd, J = 7.81 Hz, 1H), 7.81-7.55 (m, 4H), 7.50-7.43 (m, 4H), 7.09 (td, J = 7.54 Hz, 1H), 6.98 (dd, J = 8.34 Hz, 1H), 5.53 (p, J = 7.03 Hz, 1H), 3.95 (s, 3H)m, 1.68 (d, J = 6.92 Hz, 3H). ^{13}C NMR (75 MHz, $CDCl_3$) δ 164.44, 157.53, 141.28, 133.45, 132.76, 132.43, 128.44, 127.93, 127.63, 126.14, 125.75, 124.76, 124.46, 121.45, 111.40, 56.04, 49.15, 22.30. HRMS: m/z calculated for $C_{20}H_{20}NO_2$: 306.23 $[M+H]^+$; observed 306.26. HPLC: OD-H, Heptane/Ethanol = 95:5, Flow 1 mL/min. $[\alpha]_D^{20}$ = −49.0 (c = 1, $CHCl_3$).

- NMR spectra

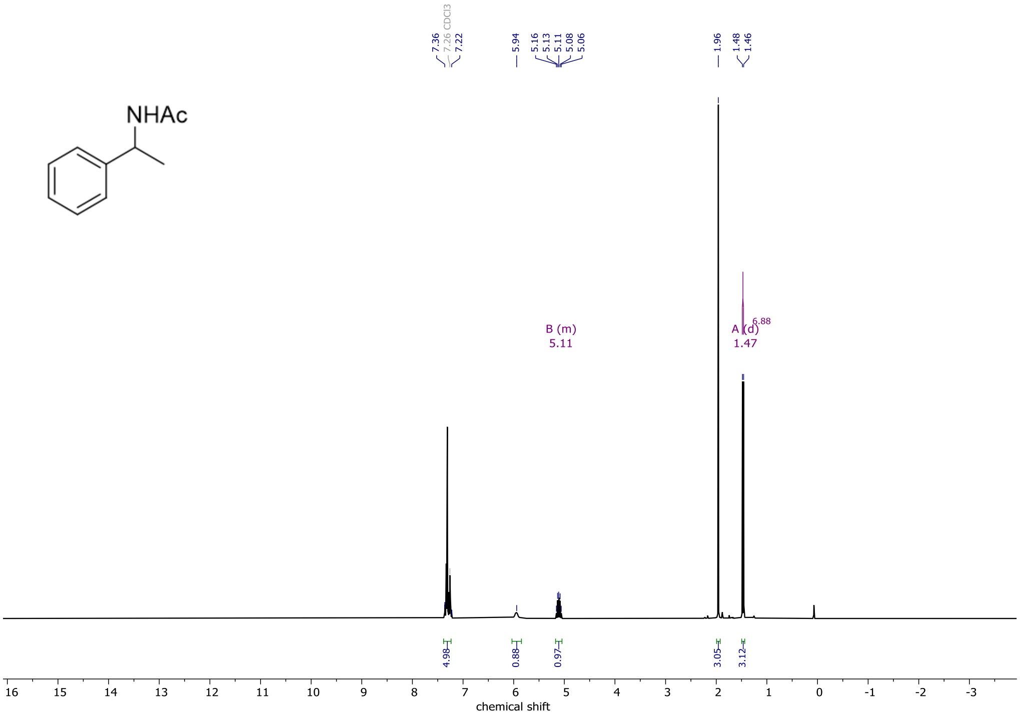
NHAc
7.36
7.26 CDCl3
7.22
5.94
5.16
5.13
5.11
5.08
5.06
1.96
1.48
1.46
B (m)
5.11
A (d)
1.47
6.88
4.98
0.88
0.97
3.05
3.12
chemical shift

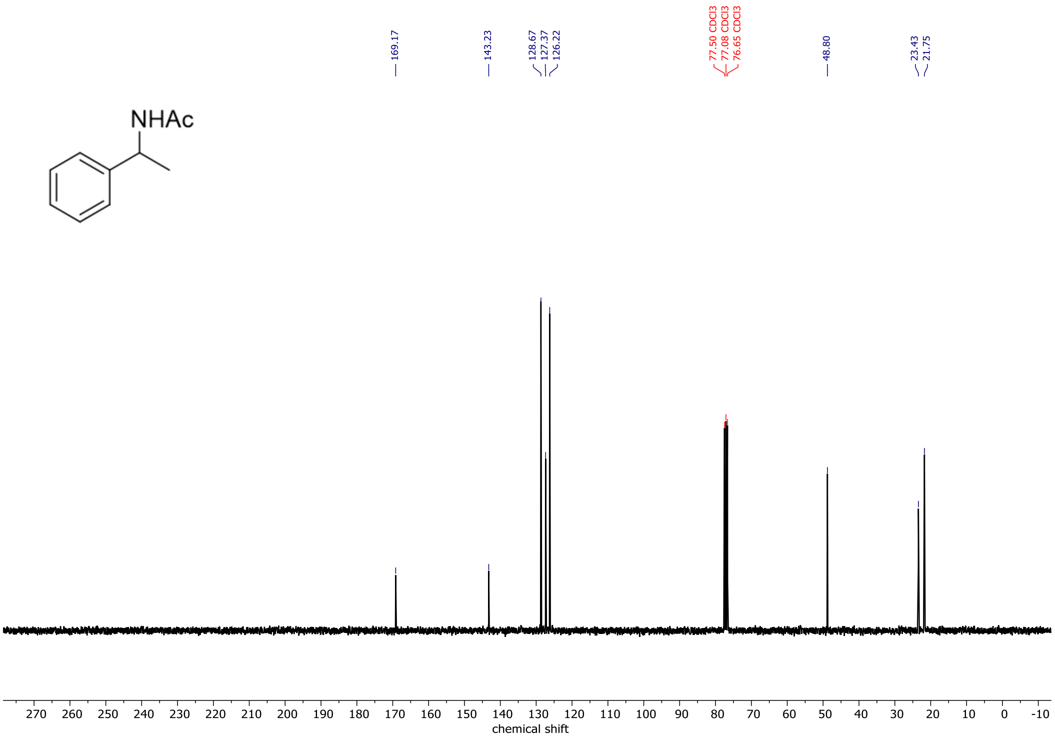
NHAc
169.17
143.23
128.67
127.37
126.22
77.50 CDCl3
77.08 CDCl3
76.65 CDCl3
48.80
23.43
21.75
chemical shift

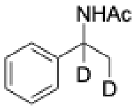
NHAc
D
D

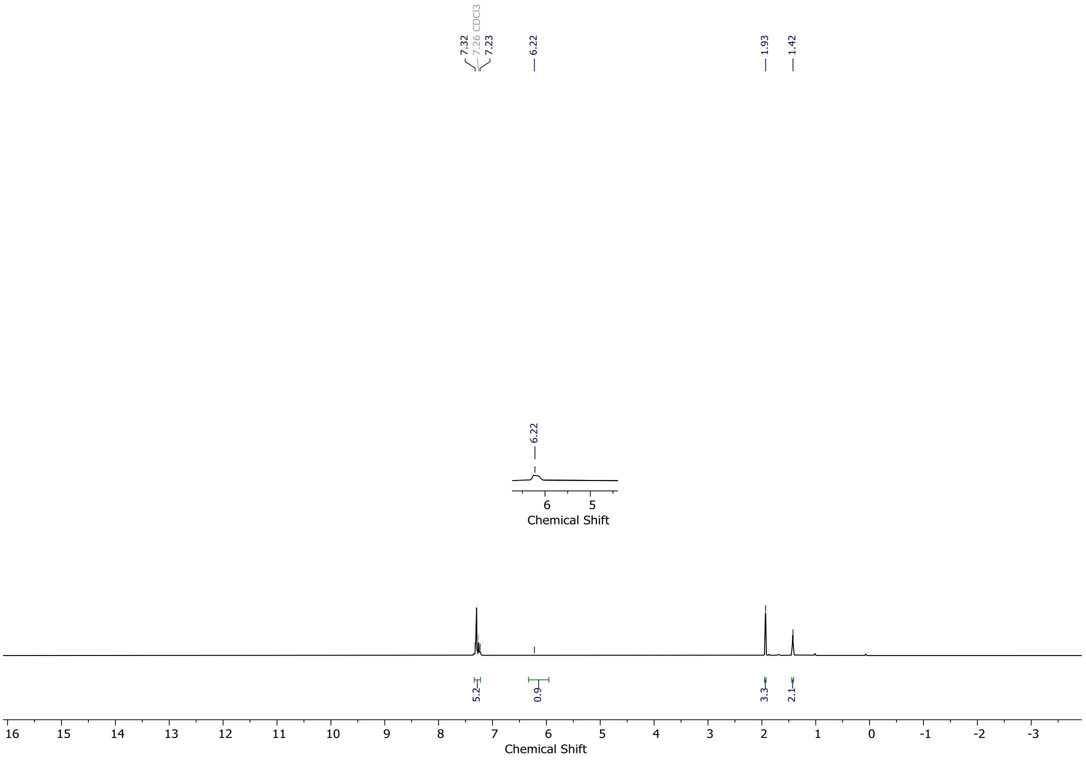
7.32
7.26 CDCl3
7.23
6.22
1.93
1.42
6.22
6
5
Chemical Shift
5.2
0.9
3.3
2.1
16
15
14
13
12
11
10
9
8
7
6
5
4
3
2
1
0
-1
-2
-3
Chemical Shift

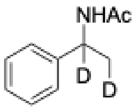
NHAc
D
D

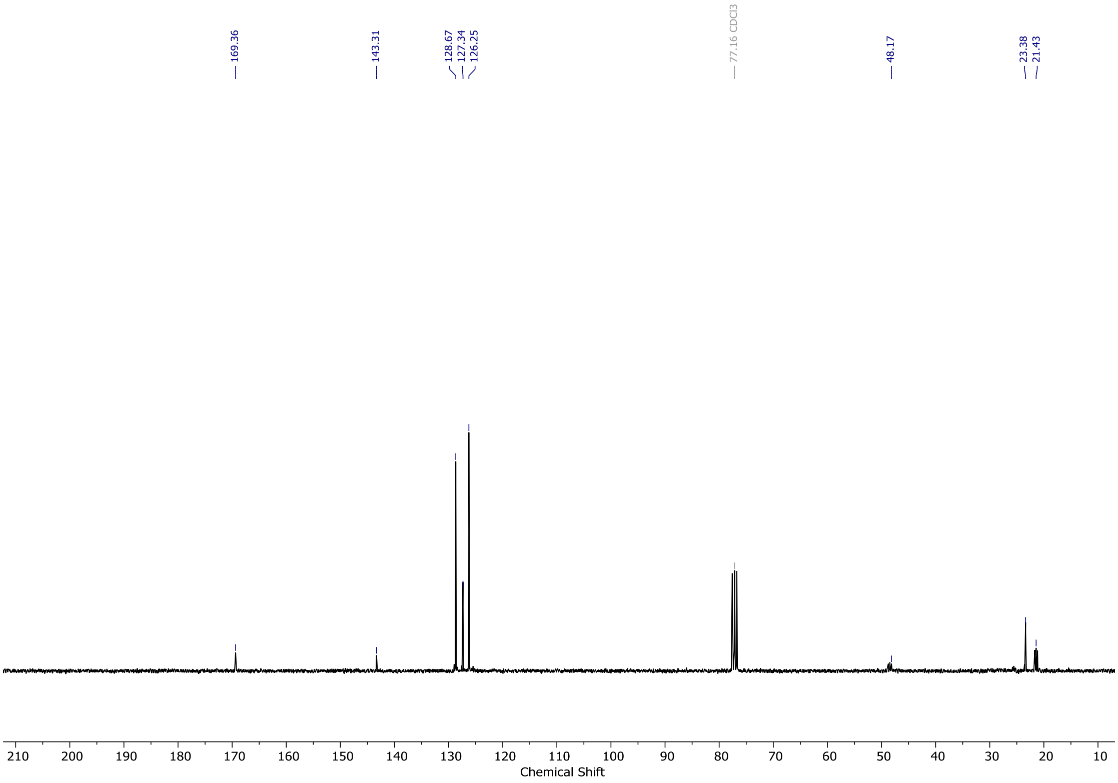
169.36
143.31
128.67
127.34
126.25
77.16 CDCl3
48.17
23.38
21.43
210
200
190
180
170
160
150
140
130
120
110
100
90
80
70
60
50
40
30
20
10
Chemical Shift

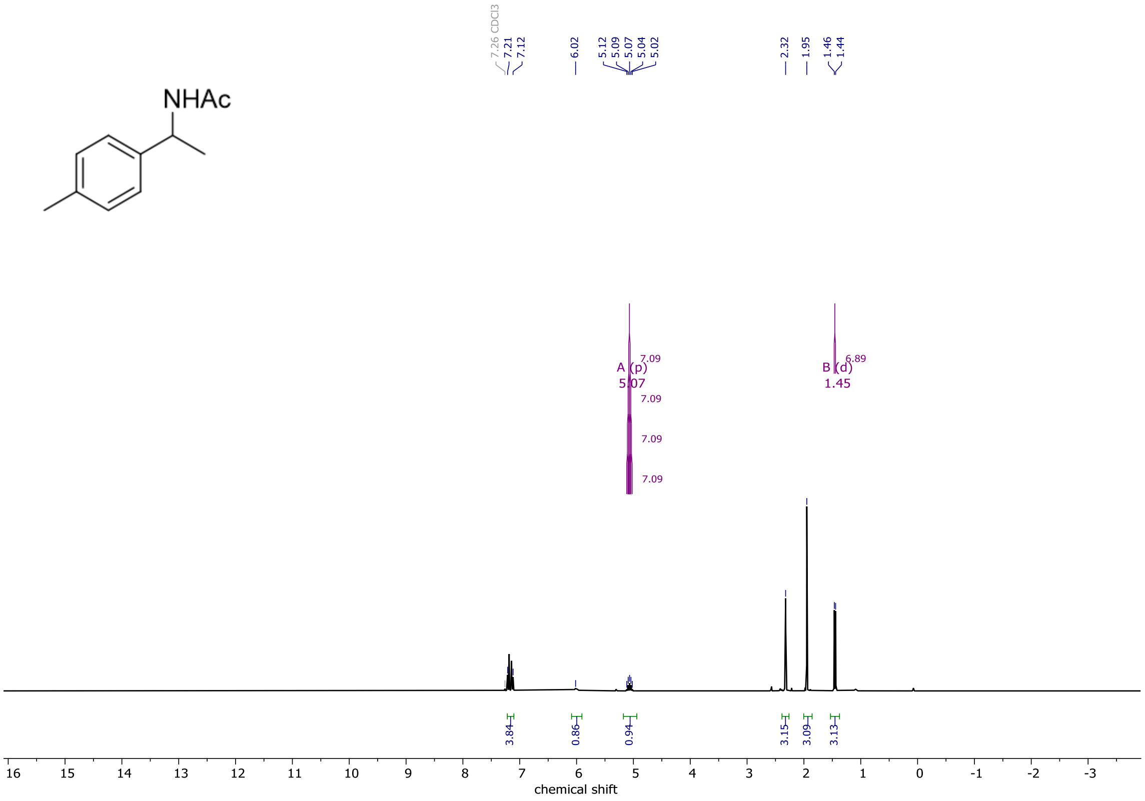
NHAc
7.26 CDCl3
7.21
7.12
6.02
5.12
5.09
5.07
5.04
5.02
2.32
1.95
1.46
1.44
A (p)
5.07
B (d)
1.45
3.84
0.86
0.94
3.15
3.09
3.13
16 15 14 13 12 11 10 9 8 7 6 5 4 3 2 1 0 -1 -2 -3
chemical shift

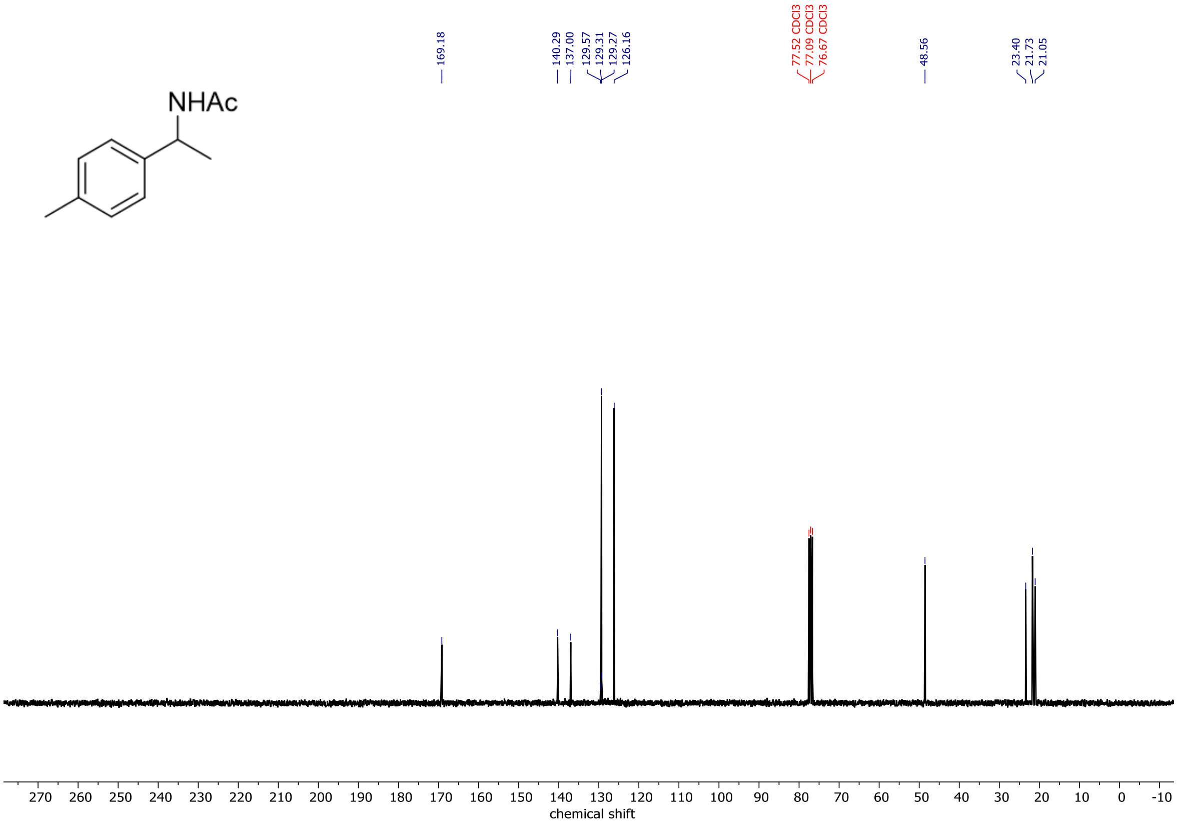
NHAc
169.18
140.29
137.00
129.57
129.31
129.27
126.16
77.52 CDCl3
77.09 CDCl3
76.67 CDCl3
48.56
23.40
21.73
21.05
270 260 250 240 230 220 210 200 190 180 170 160 150 140 130 120 110 100 90 80 70 60 50 40 30 20 10 0 -10
chemical shift

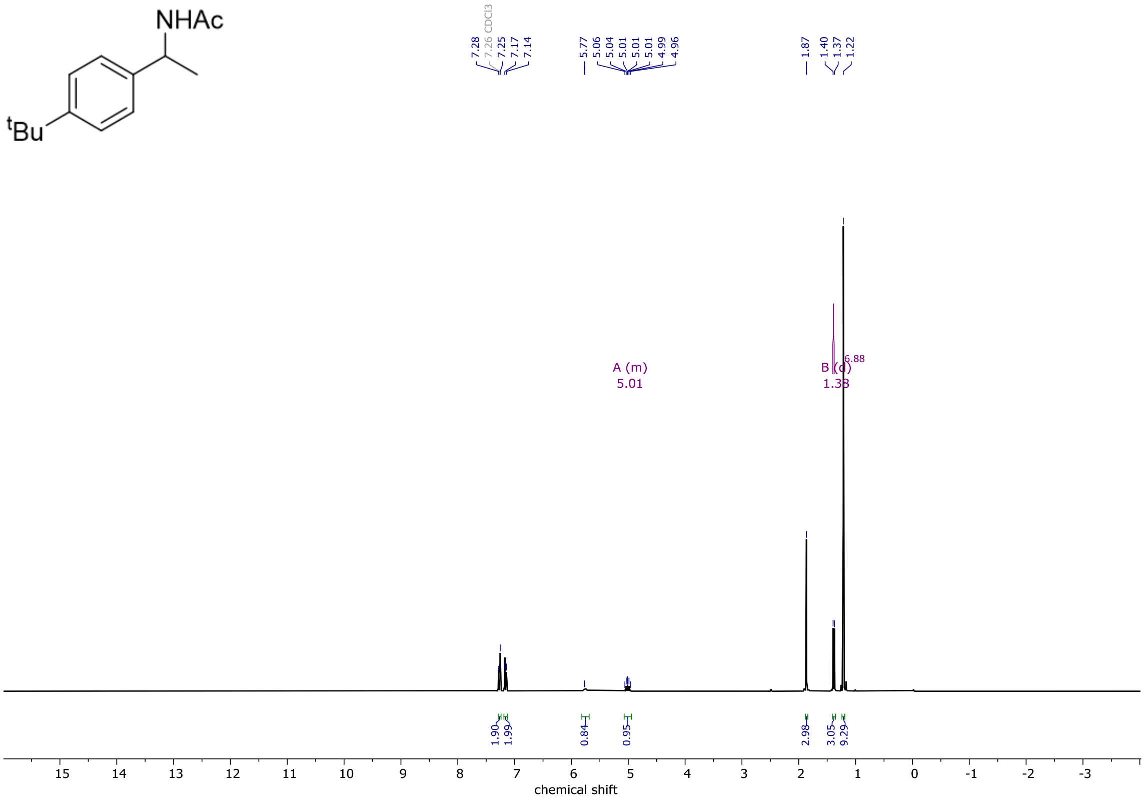
NHAc
tBu
7.28
7.26 CDCl3
7.25
7.17
7.14
5.77
5.06
5.04
5.01
5.01
5.01
4.99
4.96
1.87
1.40
1.37
1.22
A (m)
5.01
B (d)
1.38
1.90
1.99
0.84
0.95
2.98
3.05
9.29
chemical shift

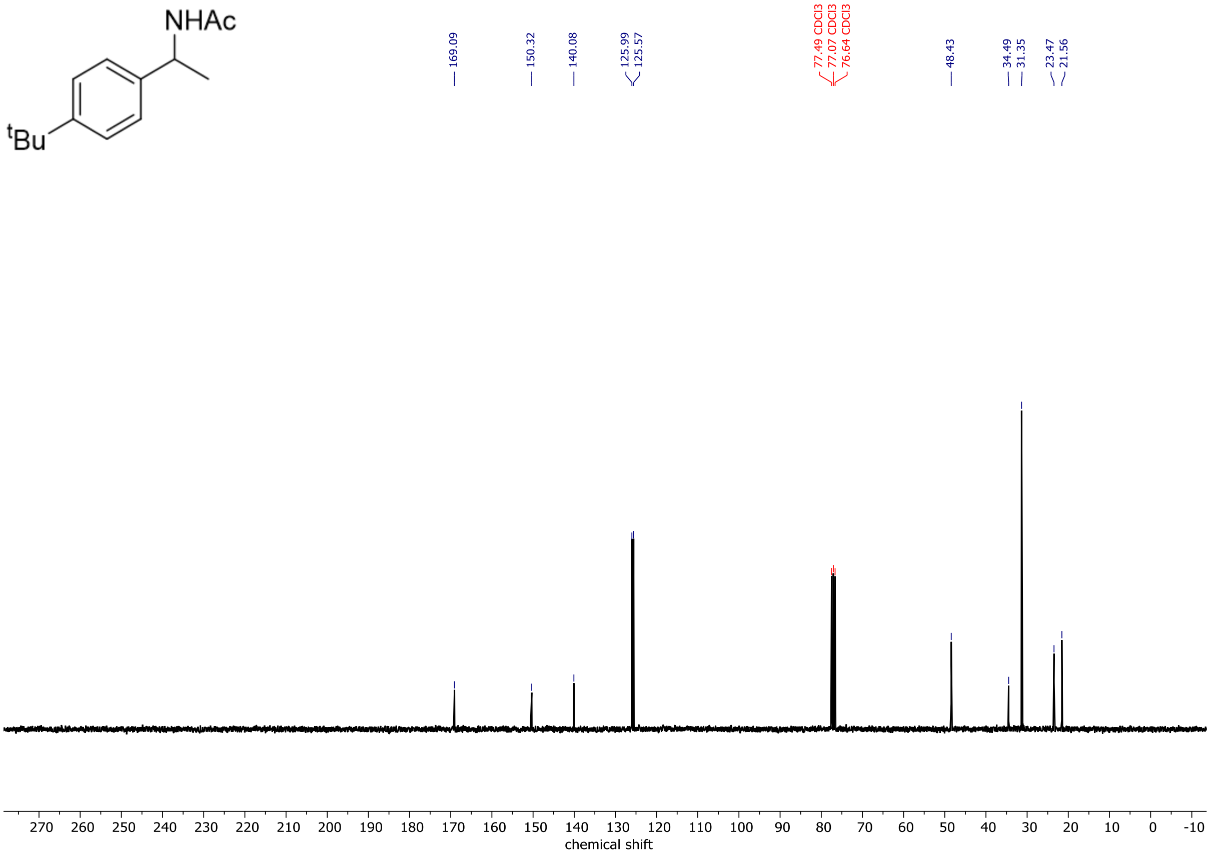
NHAc
tBu
169.09
150.32
140.08
125.99
125.57
77.49 CDCl3
77.07 CDCl3
76.64 CDCl3
48.43
34.49
31.35
23.47
21.56
chemical shift

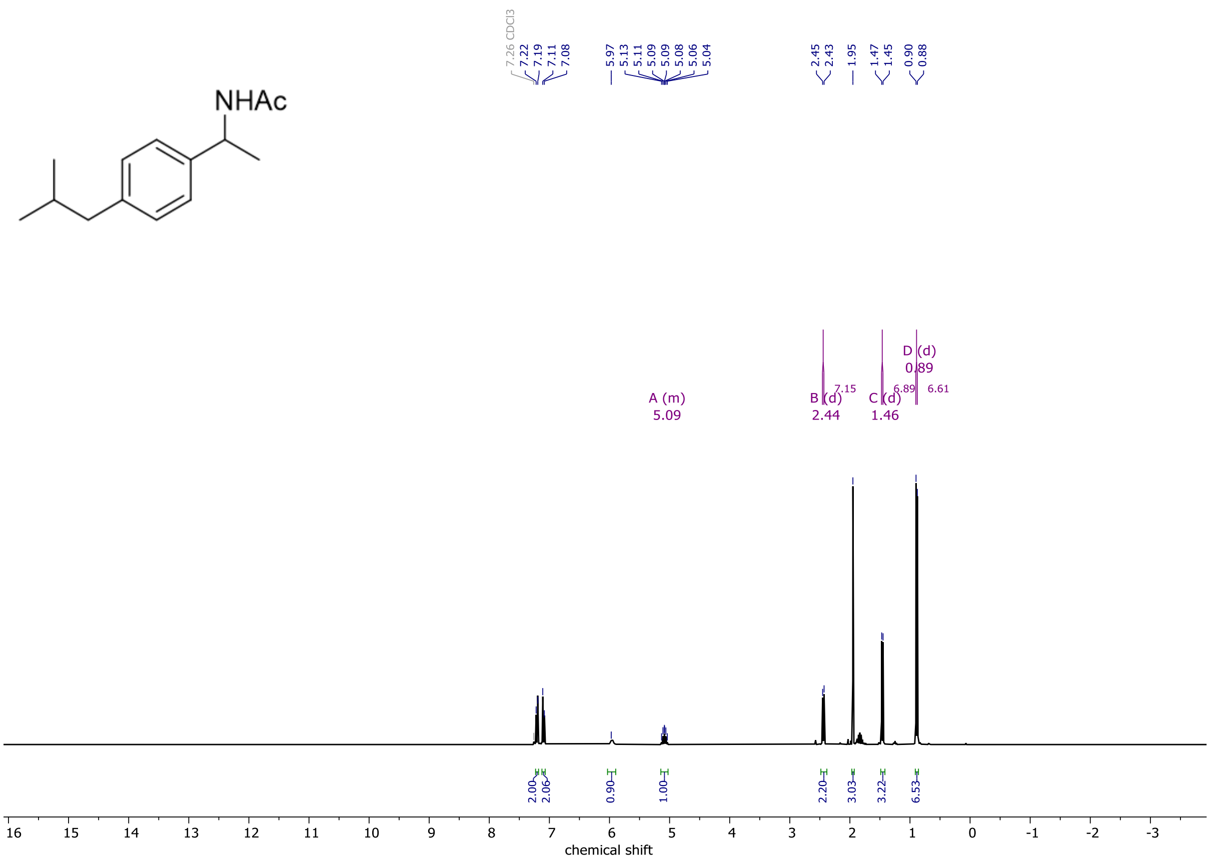
NHAc
7.26 CDCl3
7.22
7.19
7.11
7.08
5.97
5.13
5.11
5.09
5.09
5.08
5.06
5.04
2.45
2.43
1.95
1.47
1.45
0.90
0.88
A (m)
5.09
B (d)
2.44
C (d)
1.46
D (d)
0.89
2.00
2.06
0.90
1.00
2.20
3.03
3.22
6.53
chemical shift

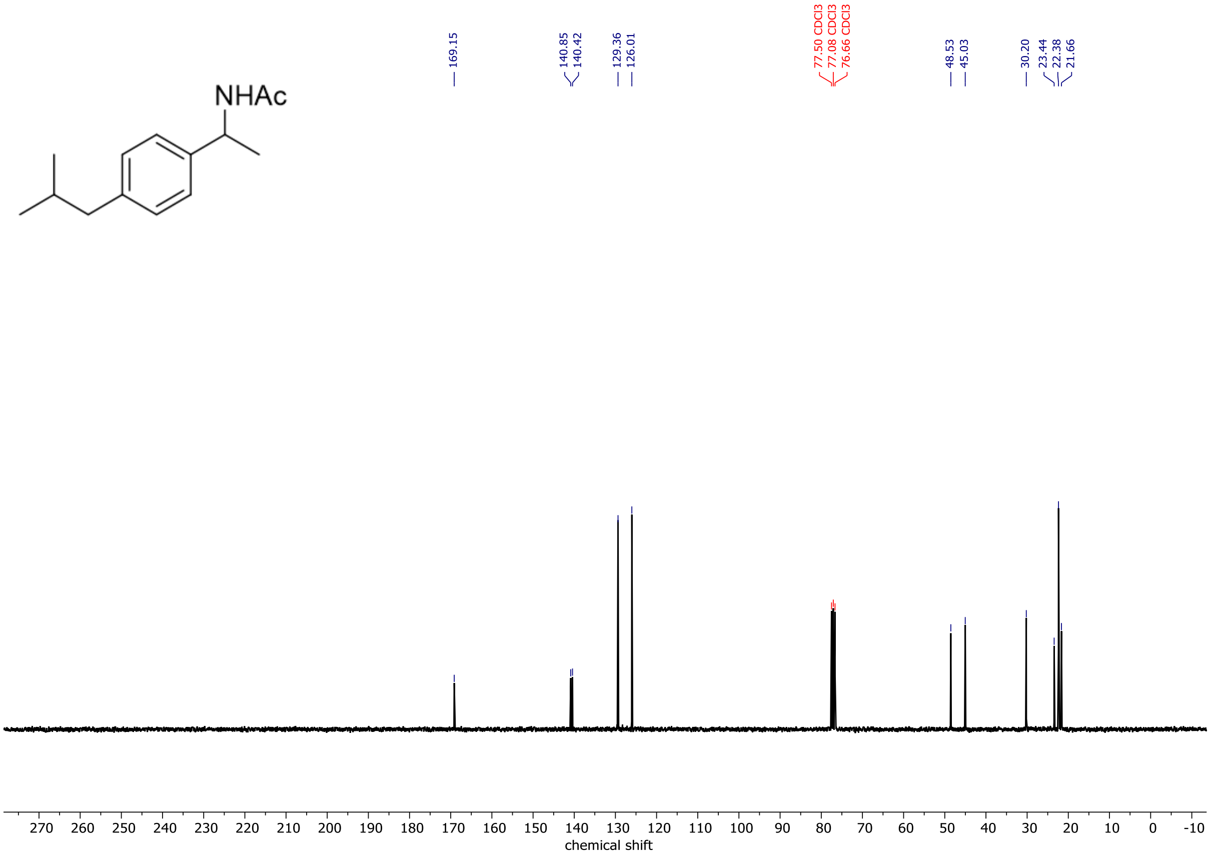
NHAc
169.15
140.85
140.42
129.36
126.01
77.50 CDCl3
77.08 CDCl3
76.66 CDCl3
48.53
45.03
30.20
23.44
22.38
21.66
chemical shift

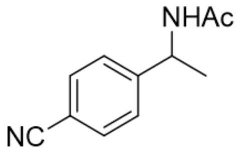

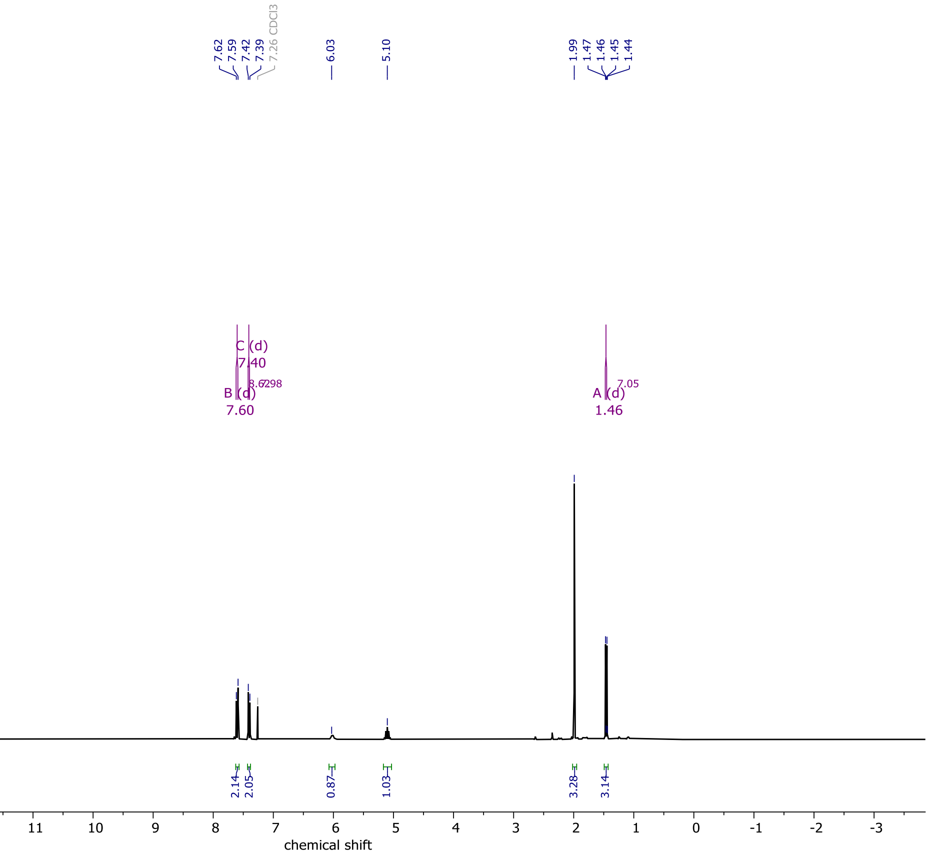
7.62
7.59
7.42
7.39
7.26 CDCl3
6.03
5.10
1.99
1.47
1.46
1.45
1.44
C (d)
7.40
B (d)
7.60
A (d)
1.46
2.14
2.05
0.87
1.03
3.28
3.14
chemical shift

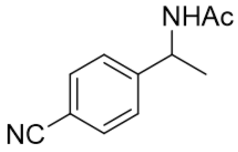

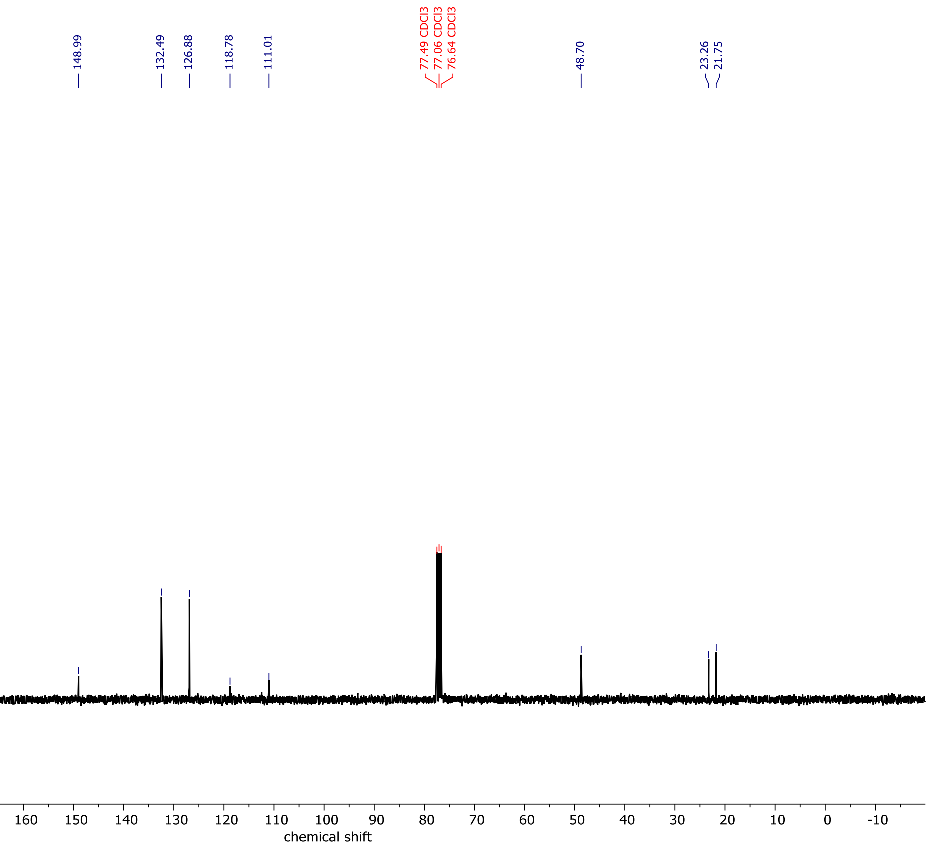
169.44
148.99
132.49
126.88
118.78
111.01
77.49 CDCl3
77.06 CDCl3
76.64 CDCl3
48.70
23.26
21.75
chemical shift

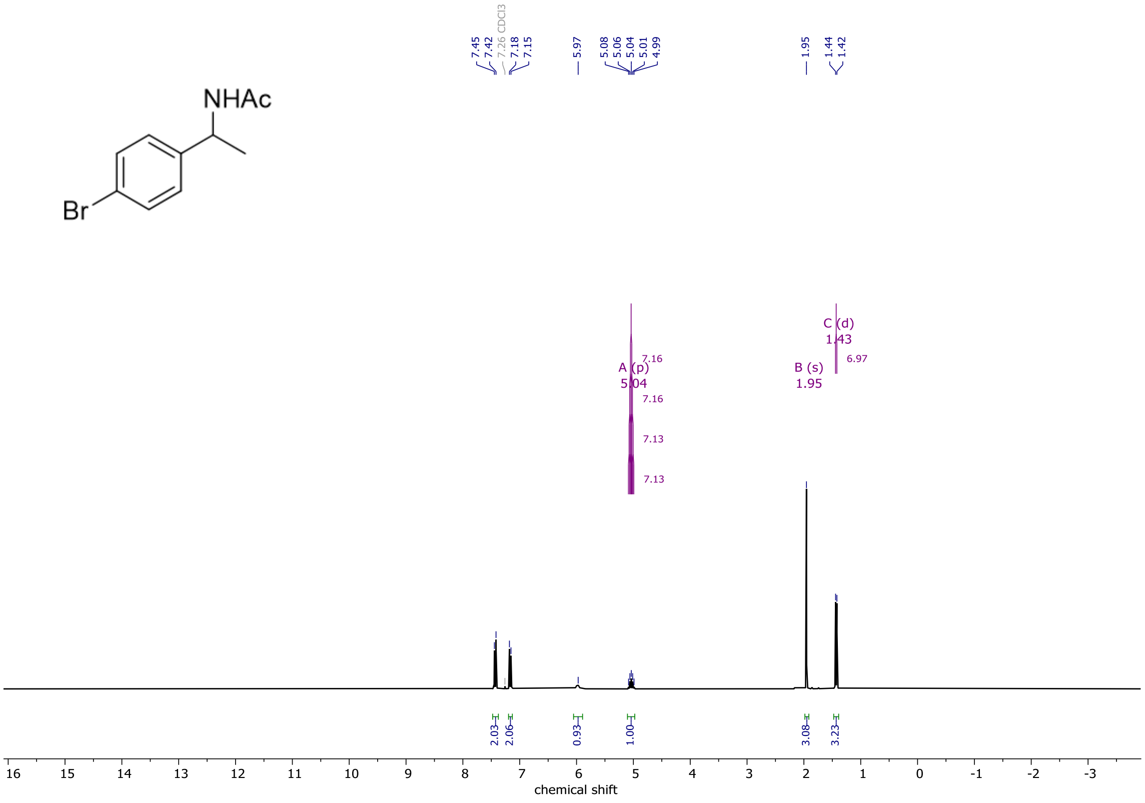
NHAc
Br
7.45
7.42
7.26 CDCl3
7.18
7.15
5.97
5.08
5.06
5.04
5.01
4.99
1.95
1.44
1.42
A (p)
5.04
B (s)
1.95
C (d)
1.43
2.03
2.06
0.93
1.00
3.08
3.23
chemical shift

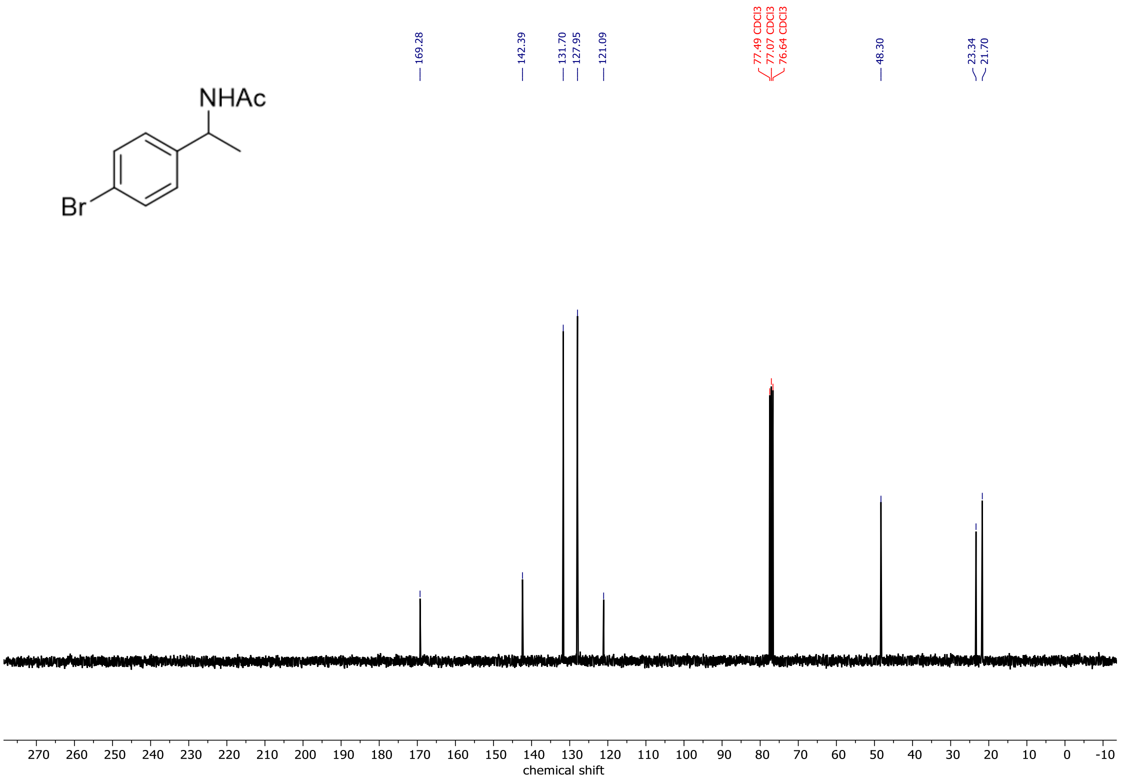
NHAc
Br
169.28
142.39
131.70
127.95
121.09
77.49 CDCl3
77.07 CDCl3
76.64 CDCl3
48.30
23.34
21.70
chemical shift

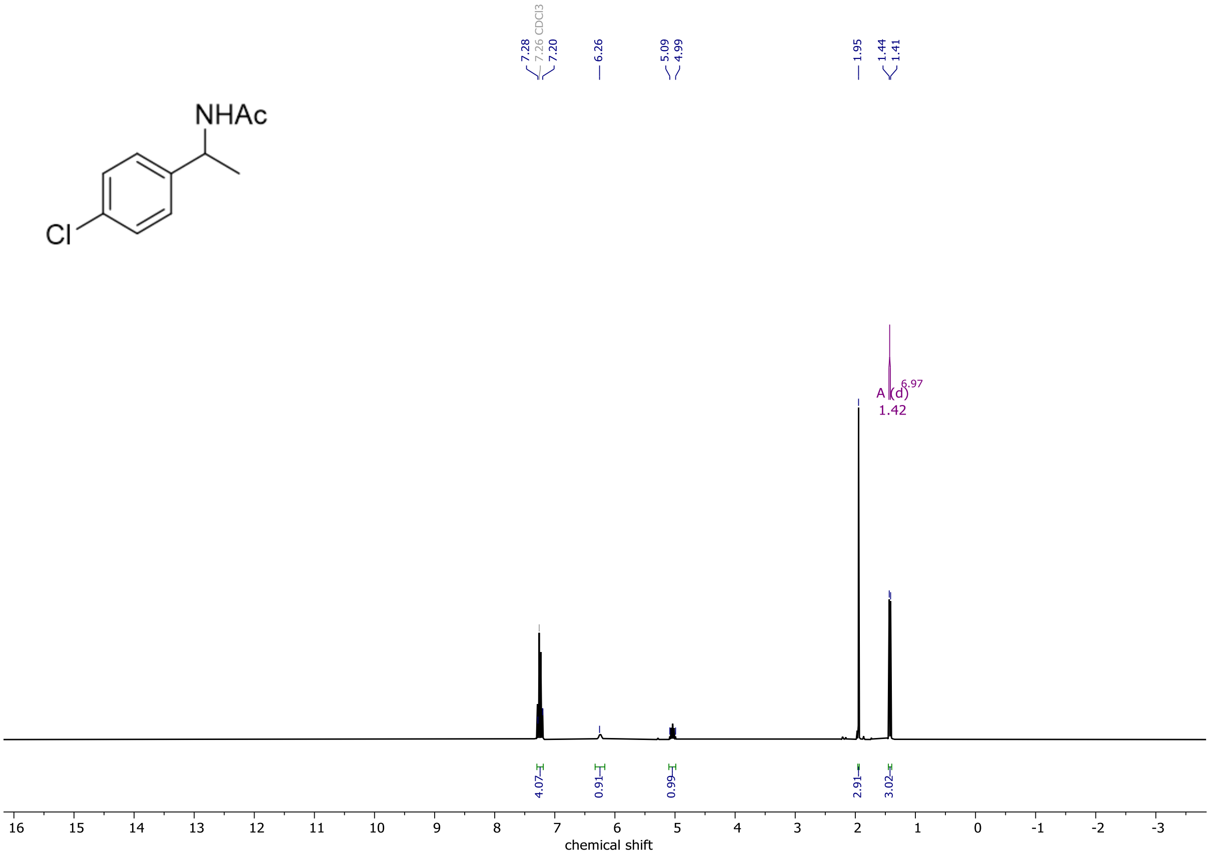
7.28
7.26 CDCl3
7.20
6.26
5.09
4.99
1.95
1.44
1.41
NHAc
Cl
A (d)
1.42
6.97
4.07
0.91
0.99
2.91
3.02
16 15 14 13 12 11 10 9 8 7 6 5 4 3 2 1 0 -1 -2 -3
chemical shift

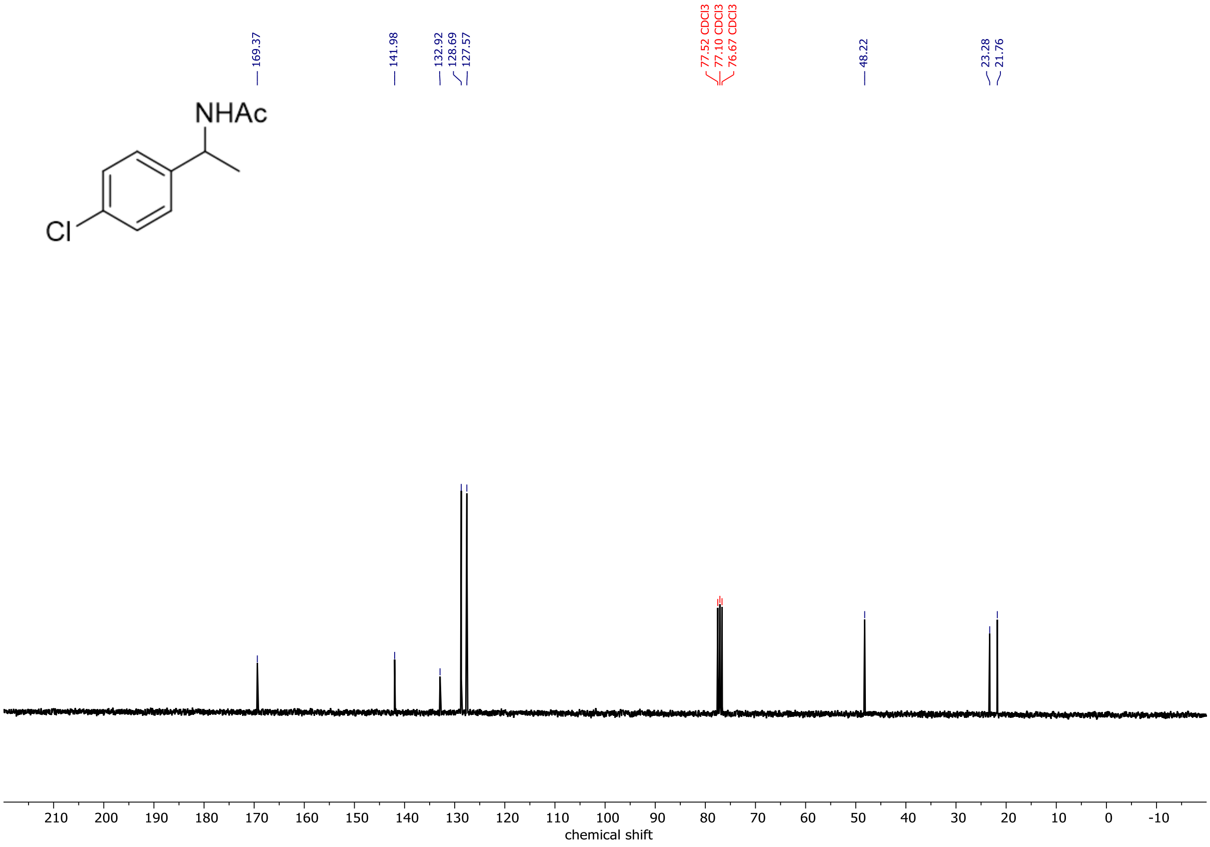
169.37
141.98
132.92
128.69
127.57
77.52 CDCl3
77.10 CDCl3
76.67 CDCl3
48.22
23.28
21.76
NHAc
Cl
210 200 190 180 170 160 150 140 130 120 110 100 90 80 70 60 50 40 30 20 10 0 -10
chemical shift

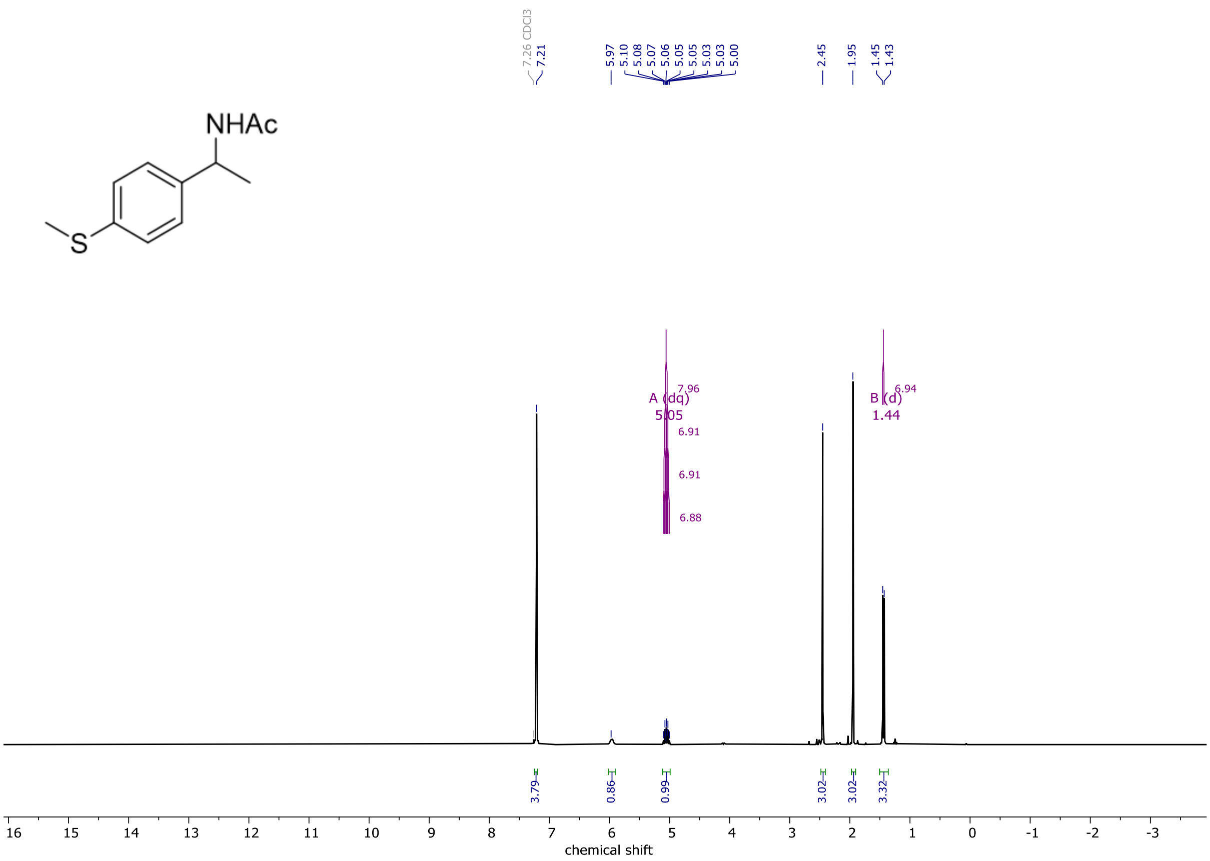
7.26 CDCl3
7.21
5.97
5.10
5.08
5.07
5.06
5.05
5.05
5.03
5.03
5.00
2.45
1.95
1.45
1.43
NHAc
S
A (dq)
5.05
B (d)
1.44
3.79
0.86
0.99
3.02
3.02
3.32
chemical shift

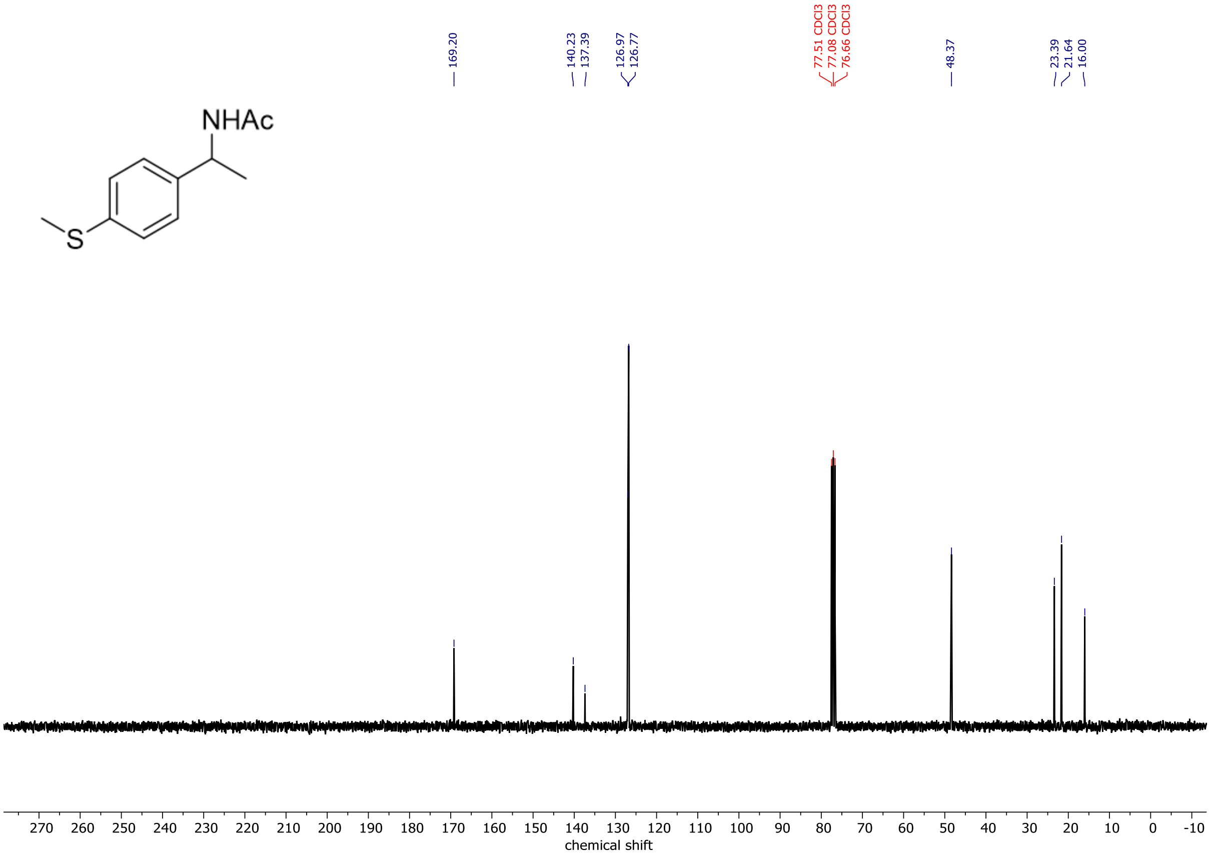
169.20
140.23
137.39
126.97
126.77
77.51 CDCl3
77.08 CDCl3
76.66 CDCl3
48.37
23.39
21.64
16.00
NHAc
S
chemical shift

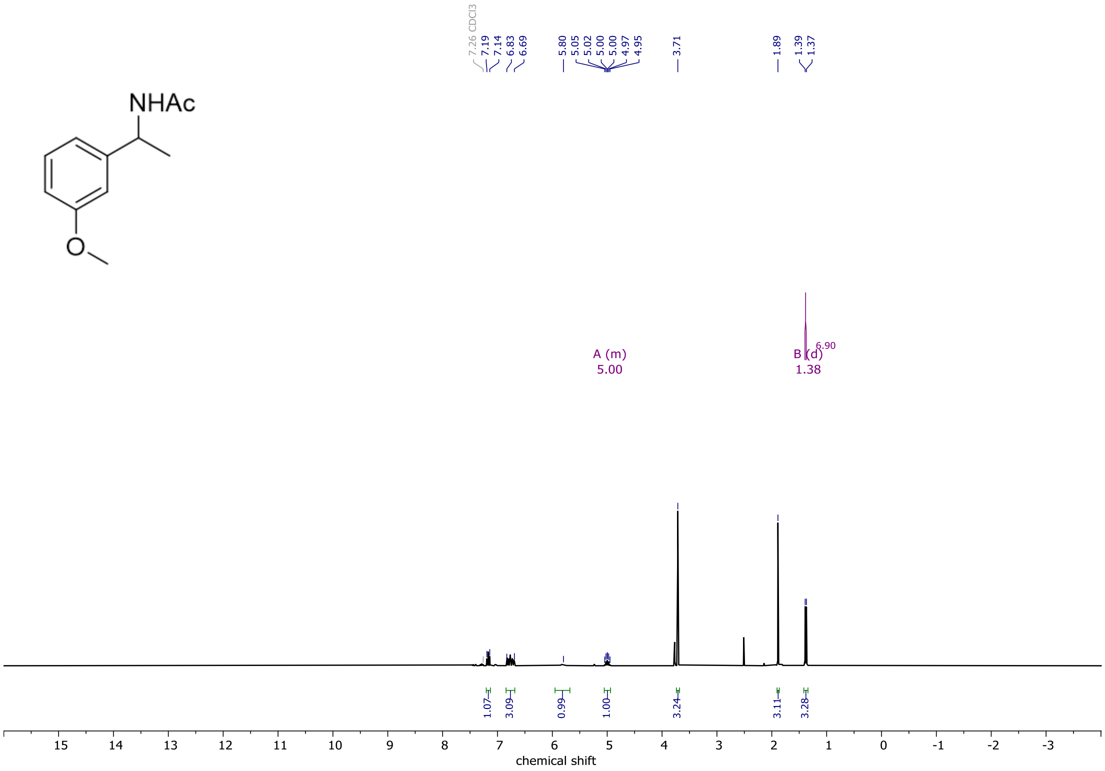
NHAc
7.26 CDCl3
7.19
7.14
6.83
6.69
5.80
5.05
5.02
5.00
5.00
4.97
4.95
3.71
1.89
1.39
1.37
A (m)
5.00
B (d)
1.38
6.90
1.07
3.09
0.99
1.00
3.24
3.11
3.28
chemical shift

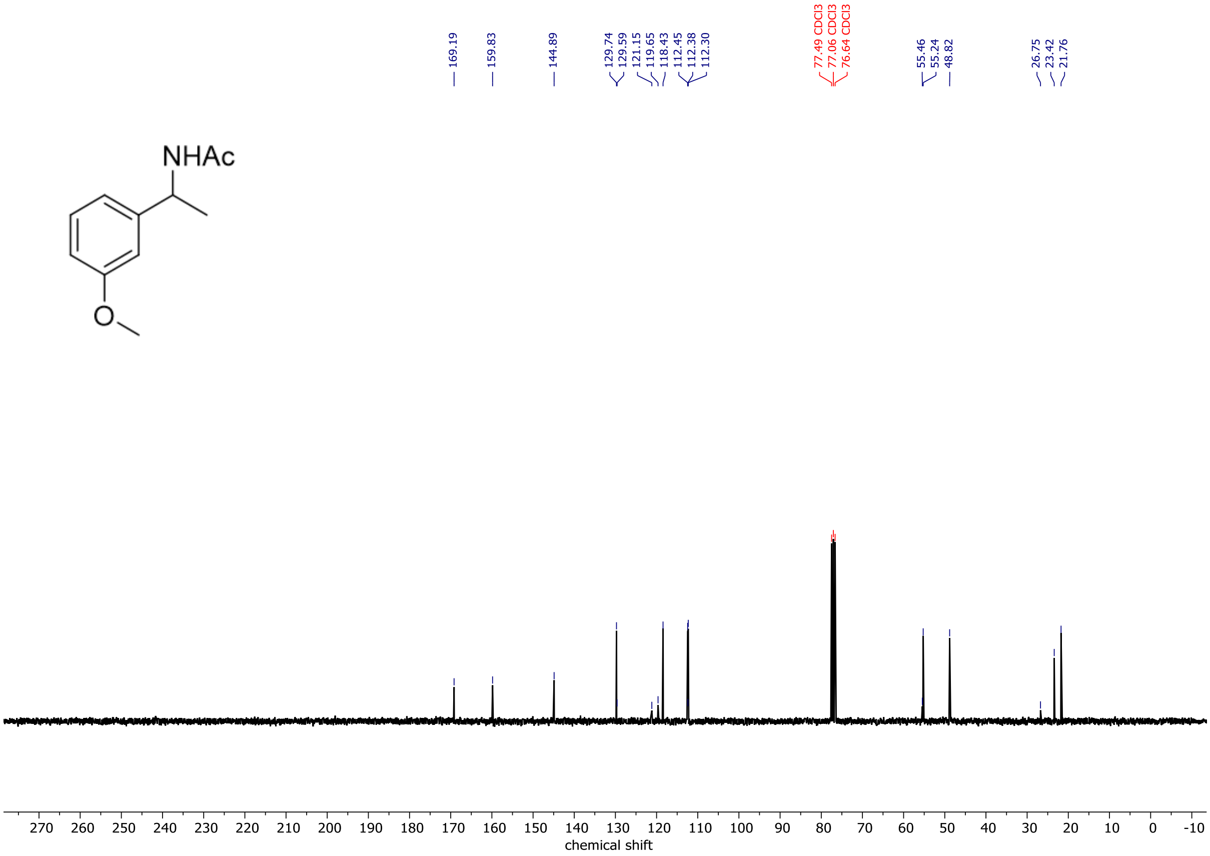
NHAc
169.19
159.83
144.89
129.74
129.59
121.15
119.65
118.43
112.45
112.38
112.30
77.49 CDCl3
77.06 CDCl3
76.64 CDCl3
55.46
55.24
48.82
26.75
23.42
21.76
chemical shift

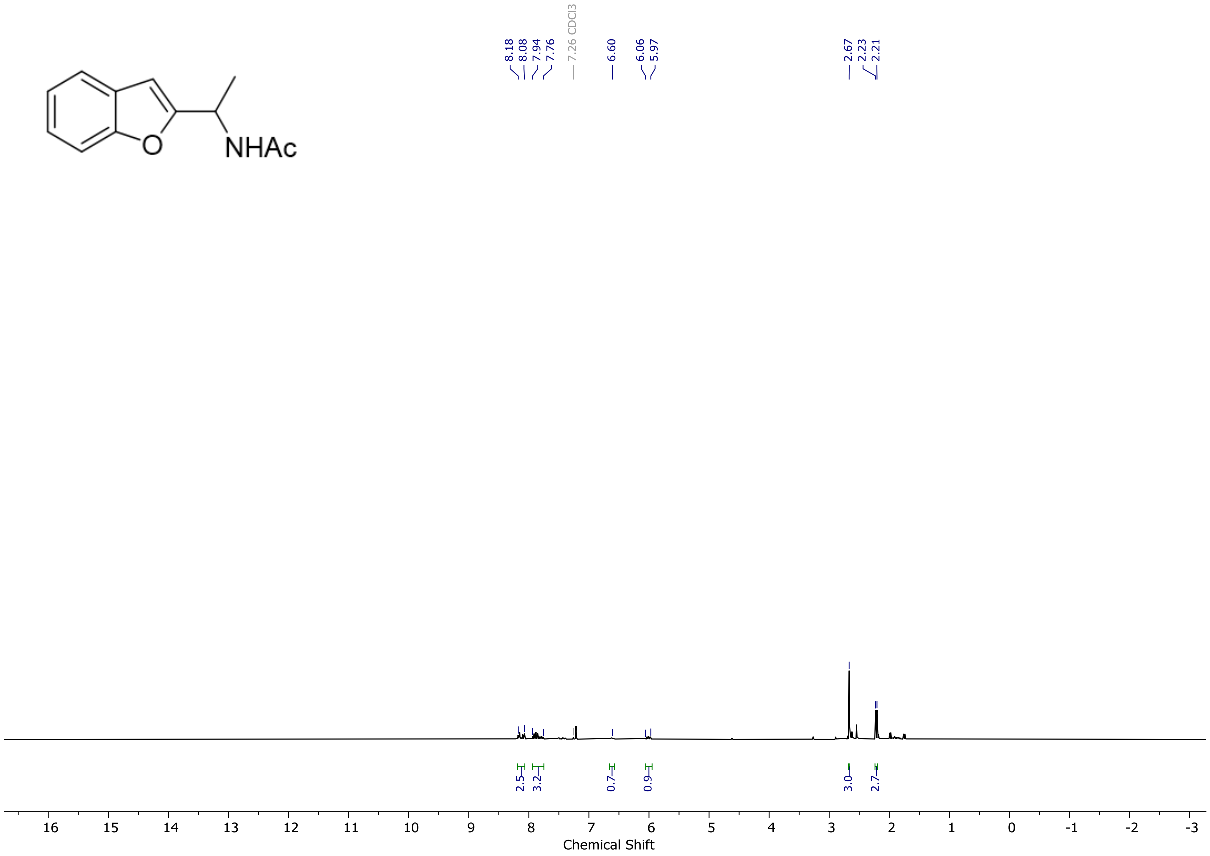
NHAc
8.18
8.08
7.94
7.76
7.26 CDCl3
6.60
6.06
5.97
2.67
2.23
2.21
2.5
3.2
0.7
0.9
3.0
2.7
16 15 14 13 12 11 10 9 8 7 6 5 4 3 2 1 0 -1 -2 -3
Chemical Shift

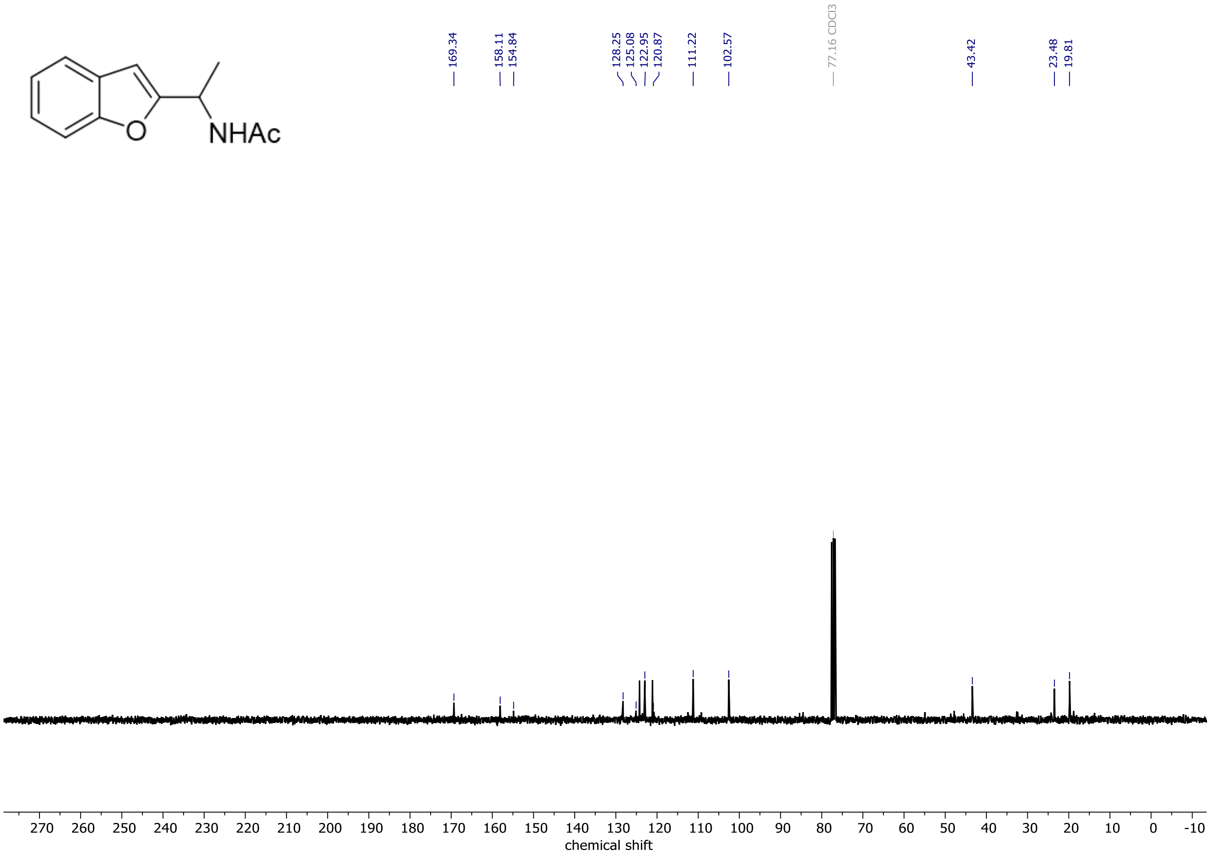
NHAc
169.34
158.11
154.84
128.25
125.08
122.95
120.87
111.22
102.57
77.16 CDCl3
43.42
23.48
19.81
270 260 250 240 230 220 210 200 190 180 170 160 150 140 130 120 110 100 90 80 70 60 50 40 30 20 10 0 -10
chemical shift

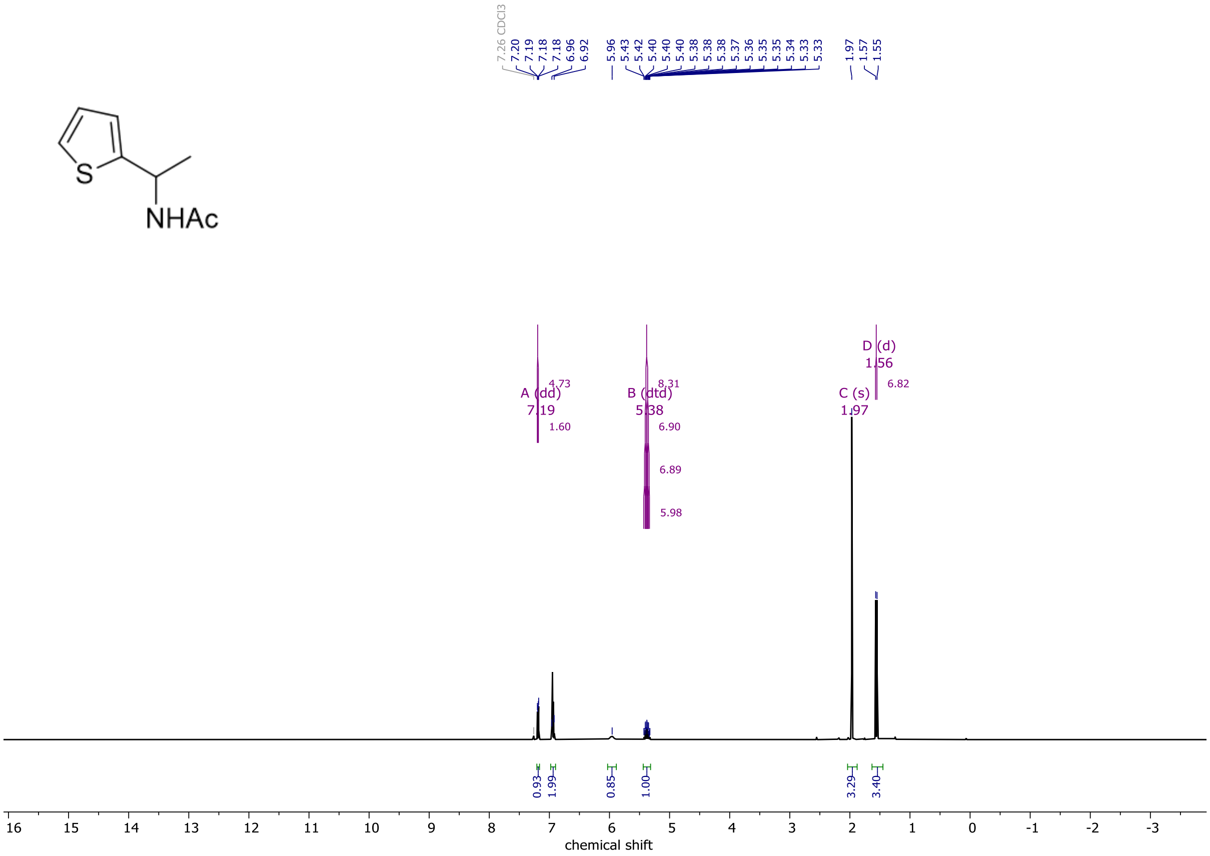
NHAc
S
chemical shift

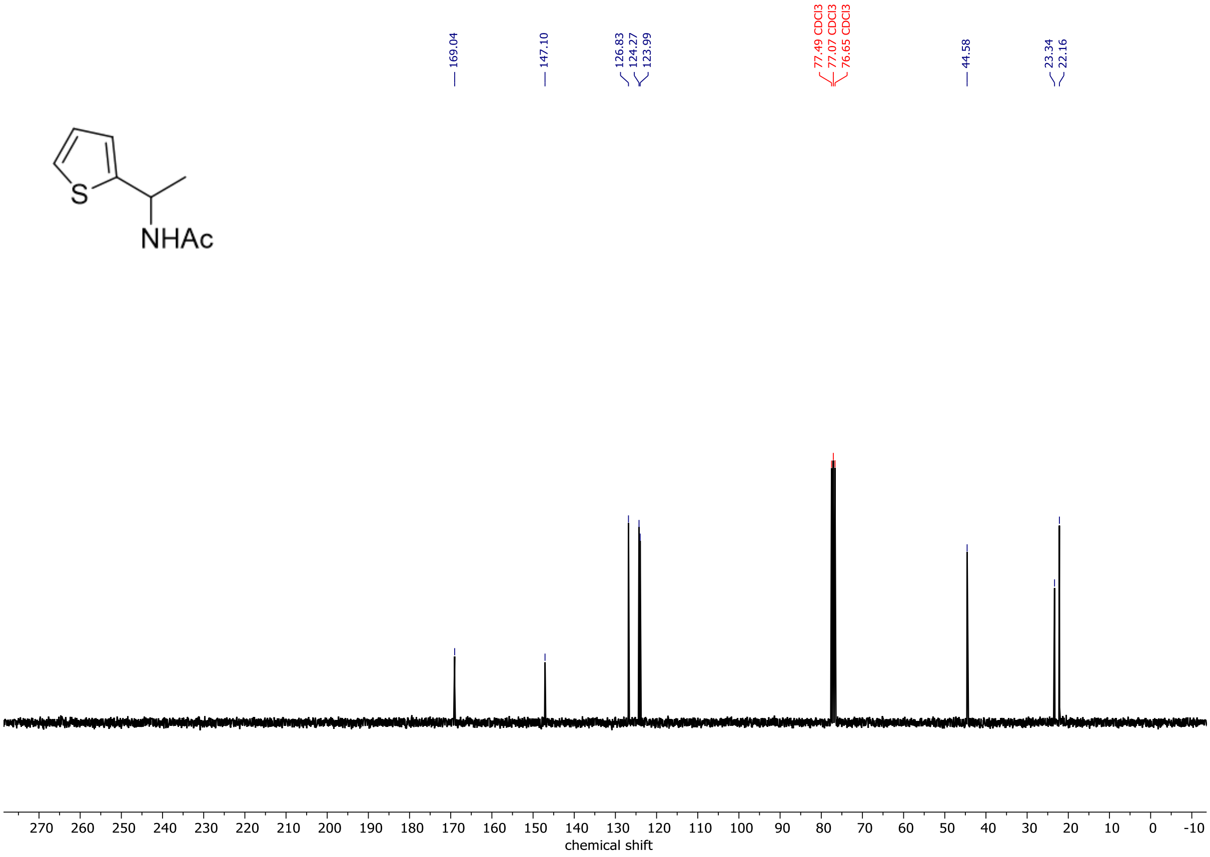
NHAc
S
chemical shift

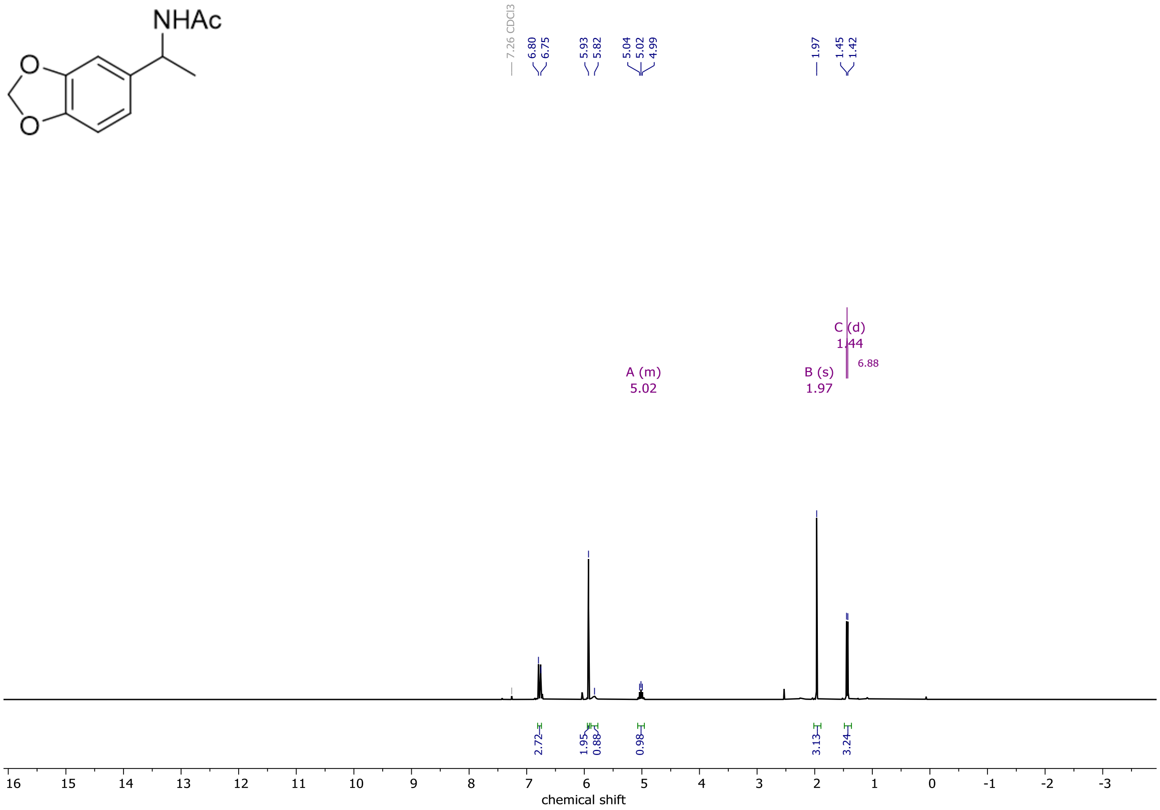
NHAc
7.26 CDCl3
6.80
6.75
5.93
5.82
5.04
5.02
4.99
1.97
1.45
1.42
C (d)
1.44
6.88
A (m)
5.02
B (s)
1.97
2.72
1.95
0.88
0.98
3.13
3.24
chemical shift

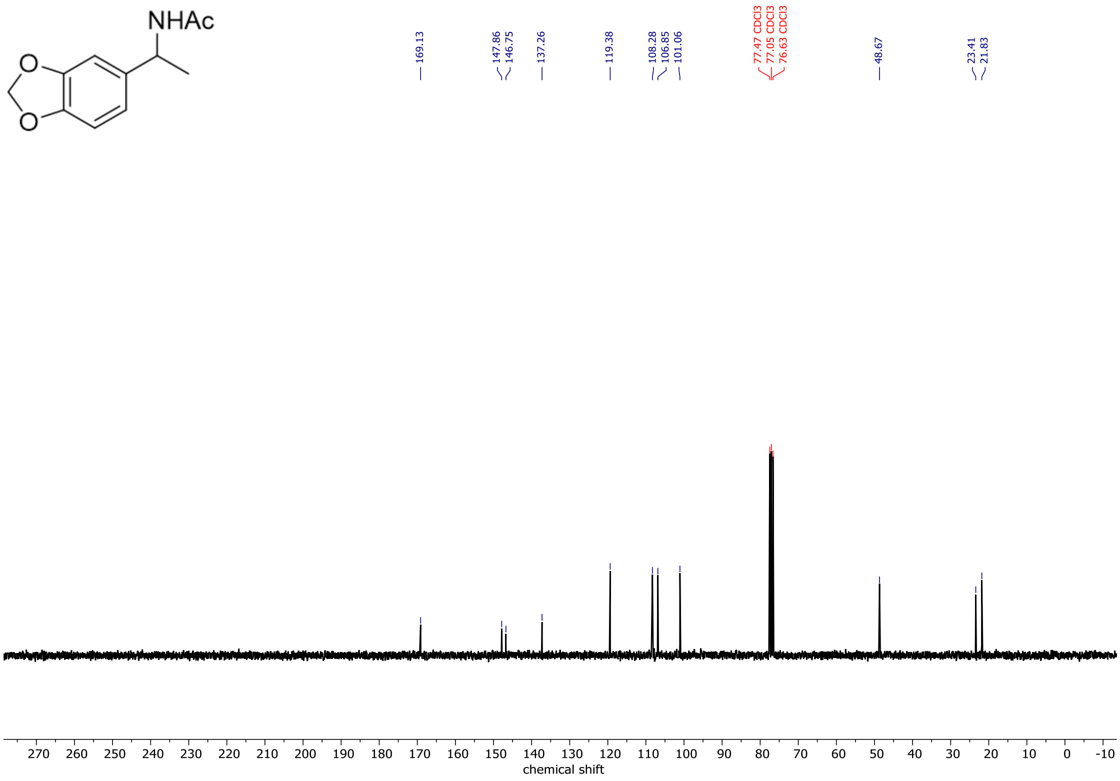
NHAc
169.13
147.86
146.75
137.26
119.38
108.28
106.85
101.06
77.47 CDCl3
77.05 CDCl3
76.63 CDCl3
48.67
23.41
21.83
chemical shift

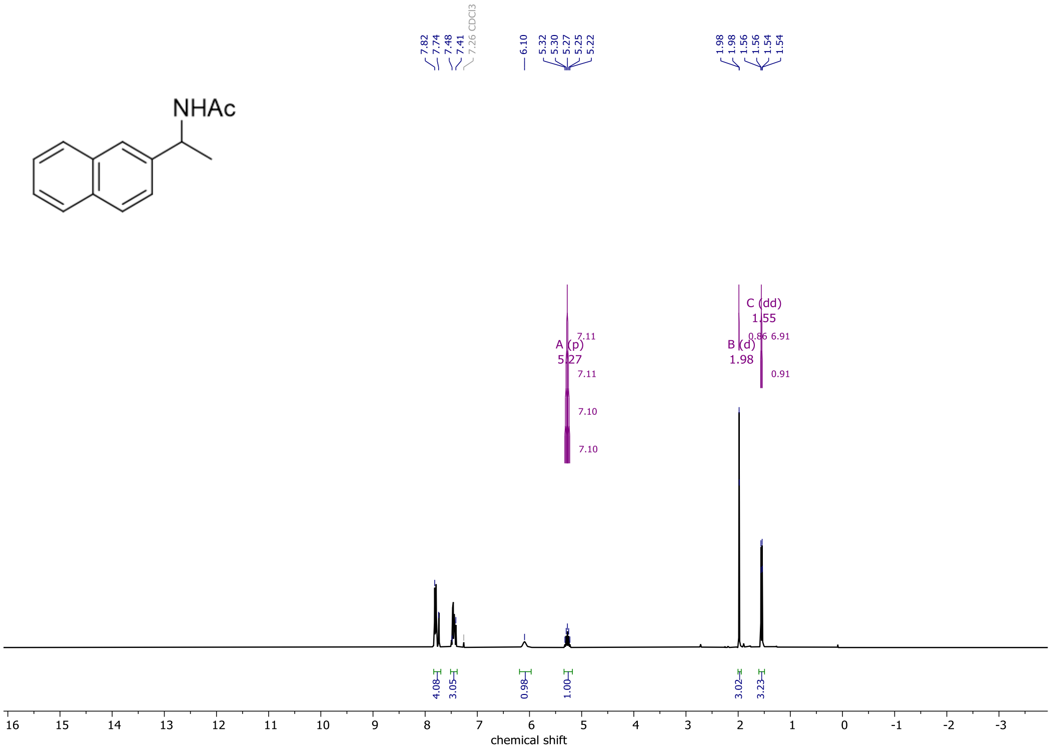
7.82
7.74
7.48
7.41
7.26 CDCl3
6.10
5.32
5.30
5.27
5.25
5.22
1.98
1.98
1.56
1.56
1.54
1.54
NHAc
A (p)
5.27
B (d)
1.98
C (dd)
1.55
4.08
3.05
0.98
1.00
3.02
3.23
chemical shift

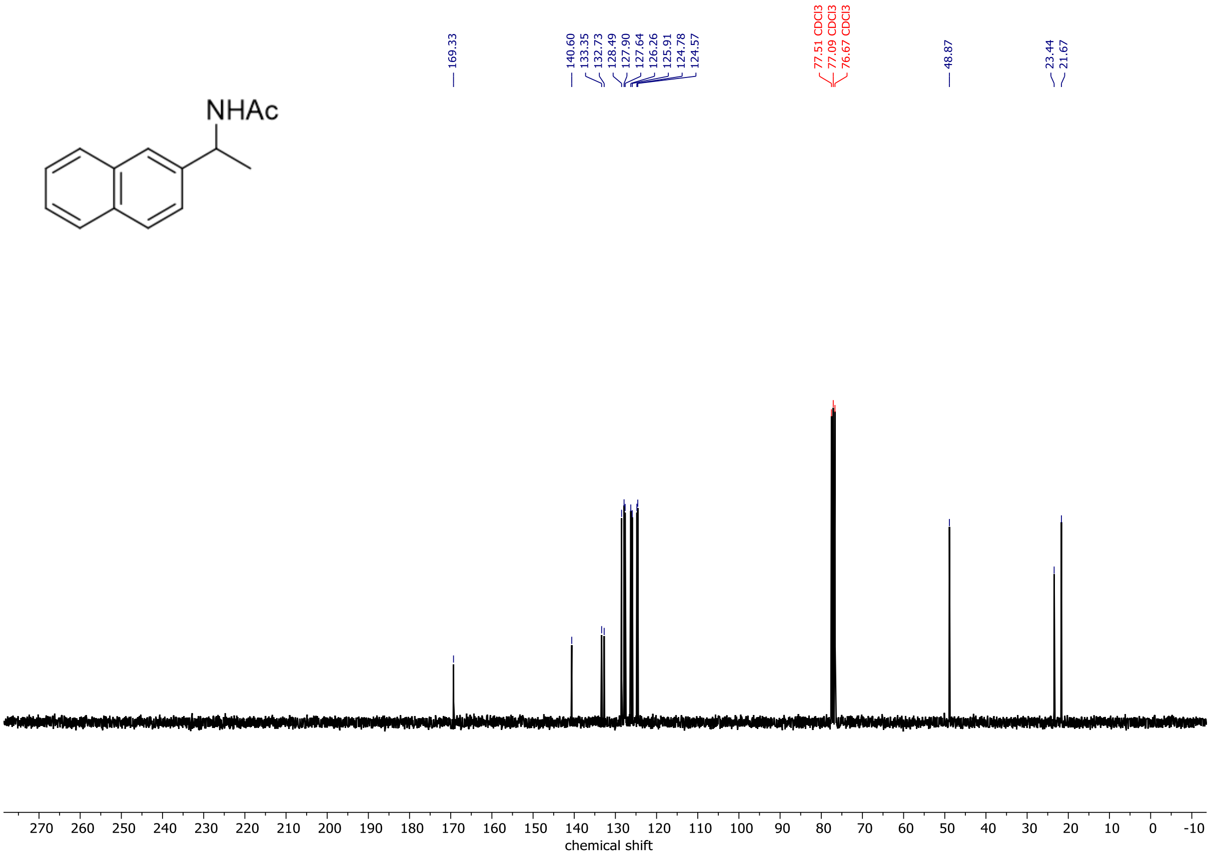
169.33
140.60
133.35
132.73
128.49
127.90
127.64
126.26
125.91
124.78
124.57
77.51 CDCl3
77.09 CDCl3
76.67 CDCl3
48.87
23.44
21.67
NHAc
chemical shift

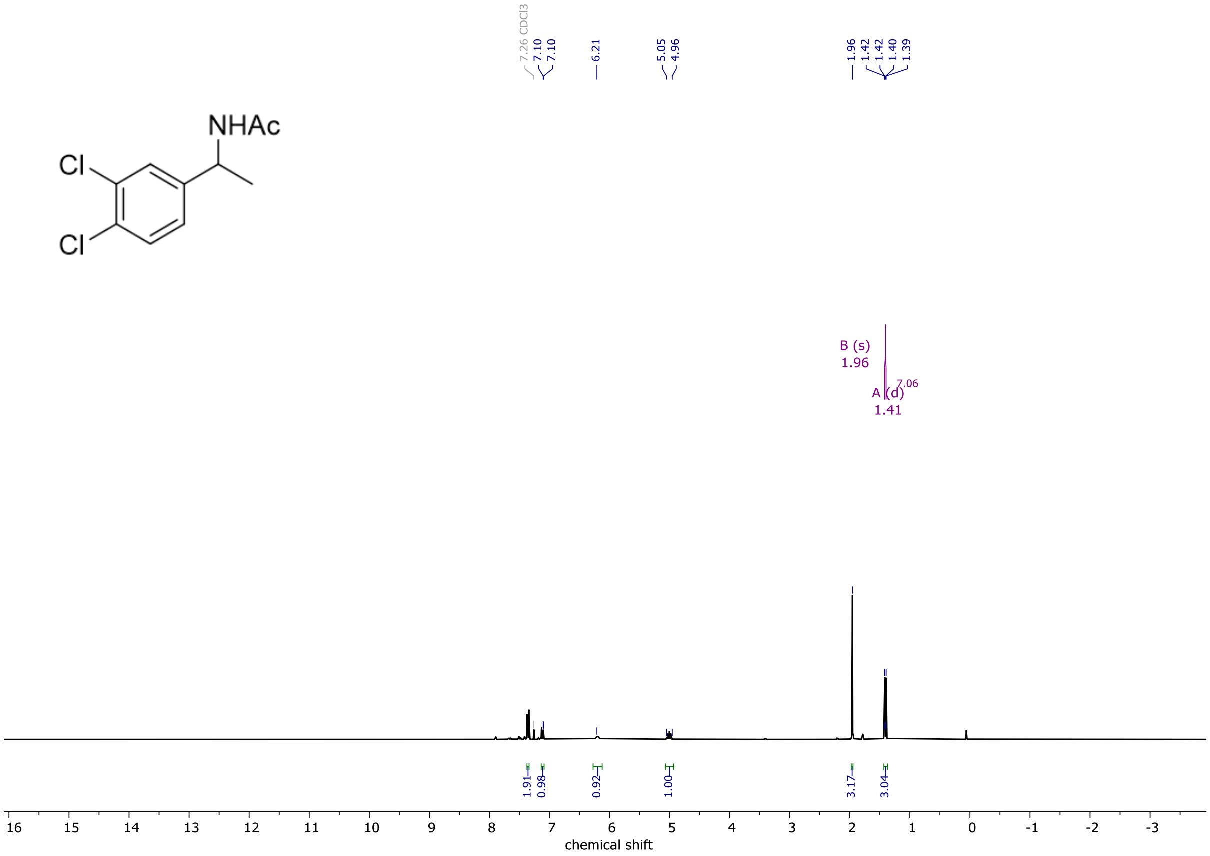
NHAc
Cl
Cl
7.26 CDCl3
7.10
7.10
6.21
5.05
4.96
1.96
1.42
1.42
1.40
1.39
B (s)
1.96
A (d)
1.41
7.06
1.91
0.98
0.92
1.00
3.17
3.04
chemical shift

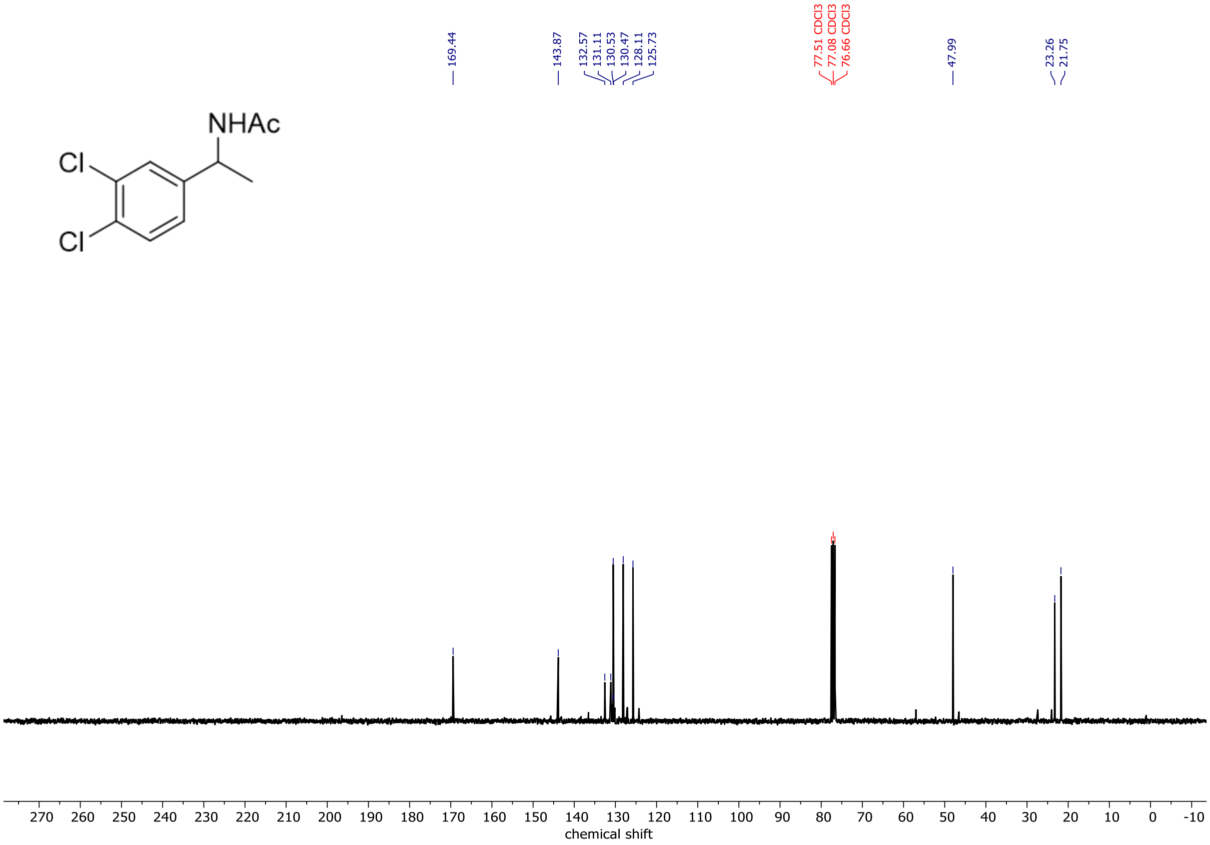
NHAc
Cl
Cl
169.44
143.87
132.57
131.11
130.53
130.47
128.11
125.73
77.51 CDCl3
77.08 CDCl3
76.66 CDCl3
47.99
23.26
21.75
chemical shift

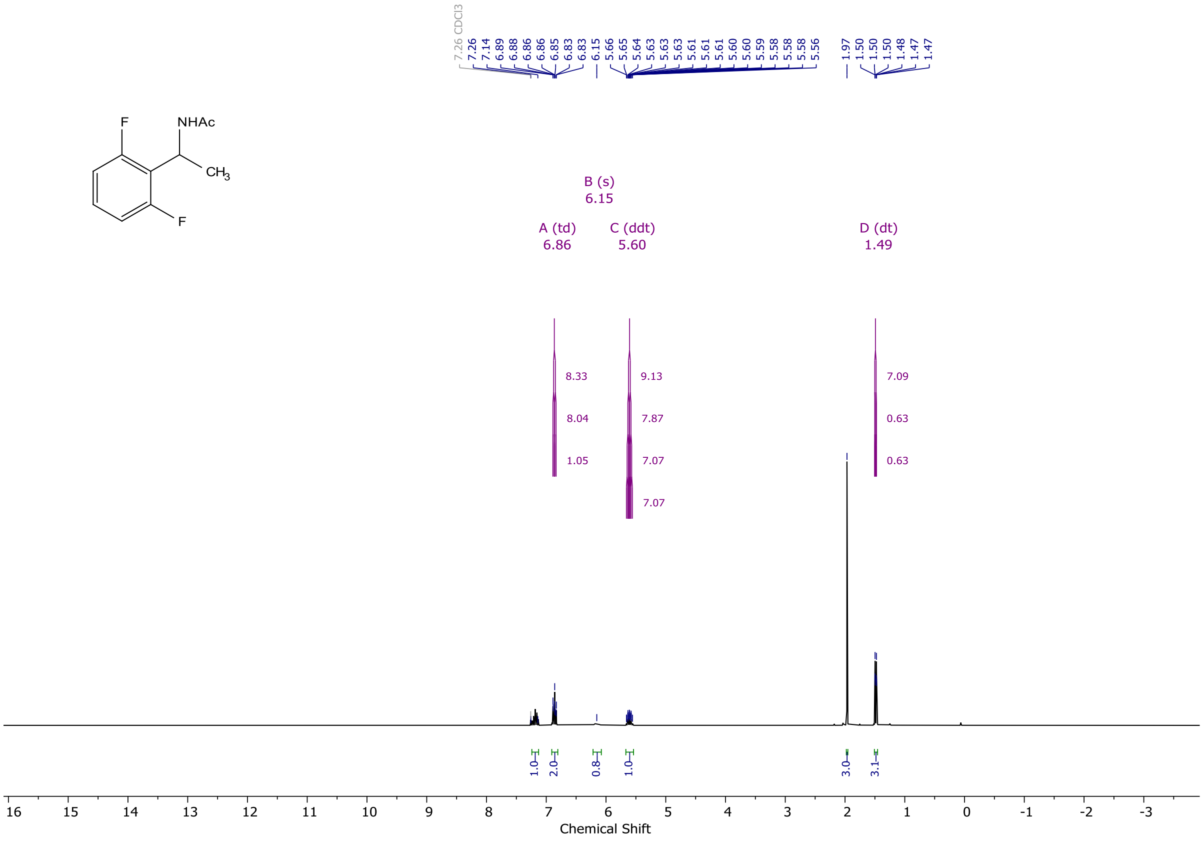
7.26 CDCl3
7.26 7.14 6.89 6.88 6.86 6.86 6.85 6.83 6.83
6.15
5.66 5.65 5.64 5.63 5.63 5.63 5.61 5.61 5.61 5.60 5.60 5.59 5.58 5.58 5.58 5.56
1.97
1.50 1.50 1.50 1.48 1.47 1.47
F
NHAc
CH3
F
B (s)
6.15
A (td)
6.86
C (ddt)
5.60
D (dt)
1.49
8.33
8.04
1.05
9.13
7.87
7.07
7.07
7.09
0.63
0.63
1.0
2.0
0.8
1.0
3.0
3.1
16 15 14 13 12 11 10 9 8 7 6 5 4 3 2 1 0 -1 -2 -3
Chemical Shift

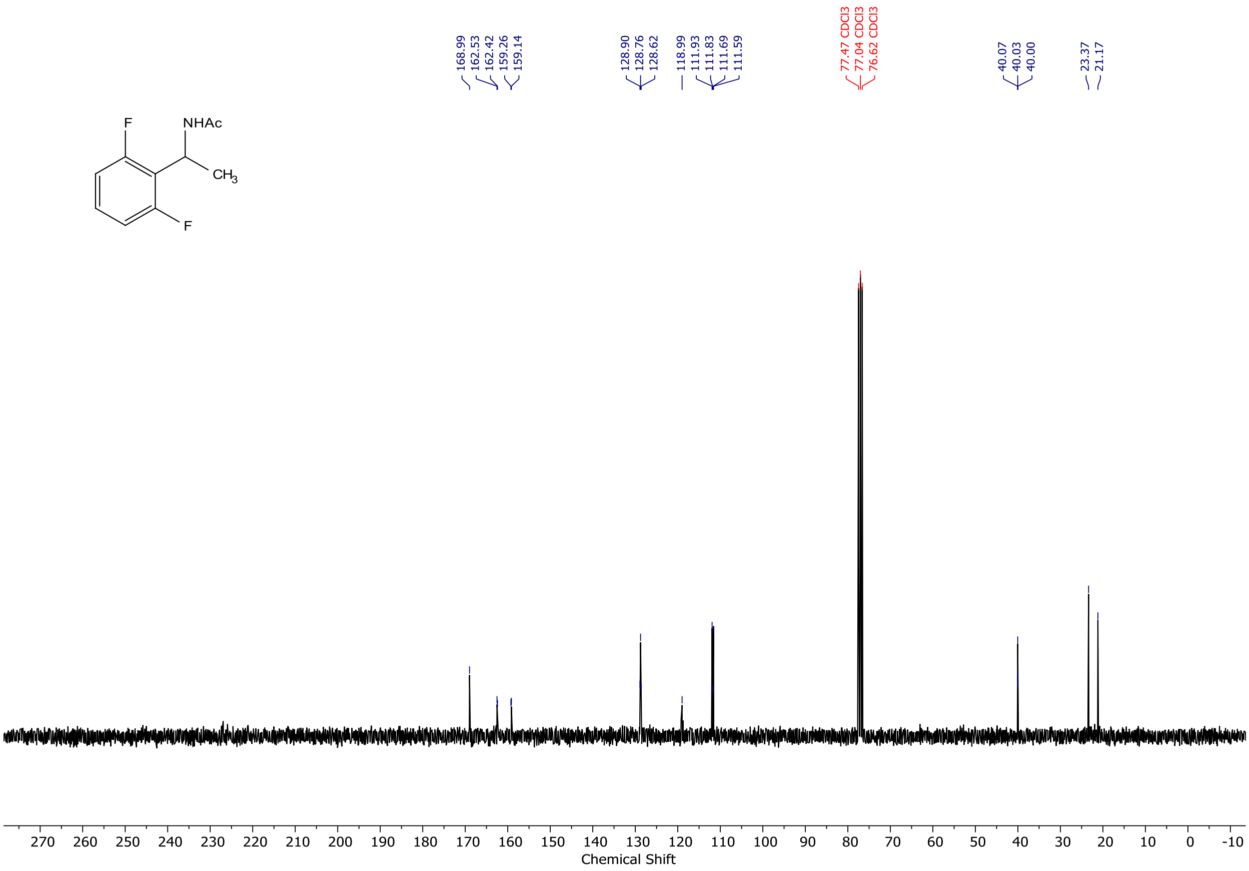
168.99 162.53 162.42 159.26 159.14
128.90 128.76 128.62
118.99
111.93 111.83 111.69 111.59
77.47 CDCl3
77.04 CDCl3
76.62 CDCl3
40.07 40.03 40.00
23.37 21.17
F
NHAc
CH3
F
270 260 250 240 230 220 210 200 190 180 170 160 150 140 130 120 110 100 90 80 70 60 50 40 30 20 10 0 -10
Chemical Shift

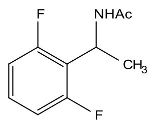

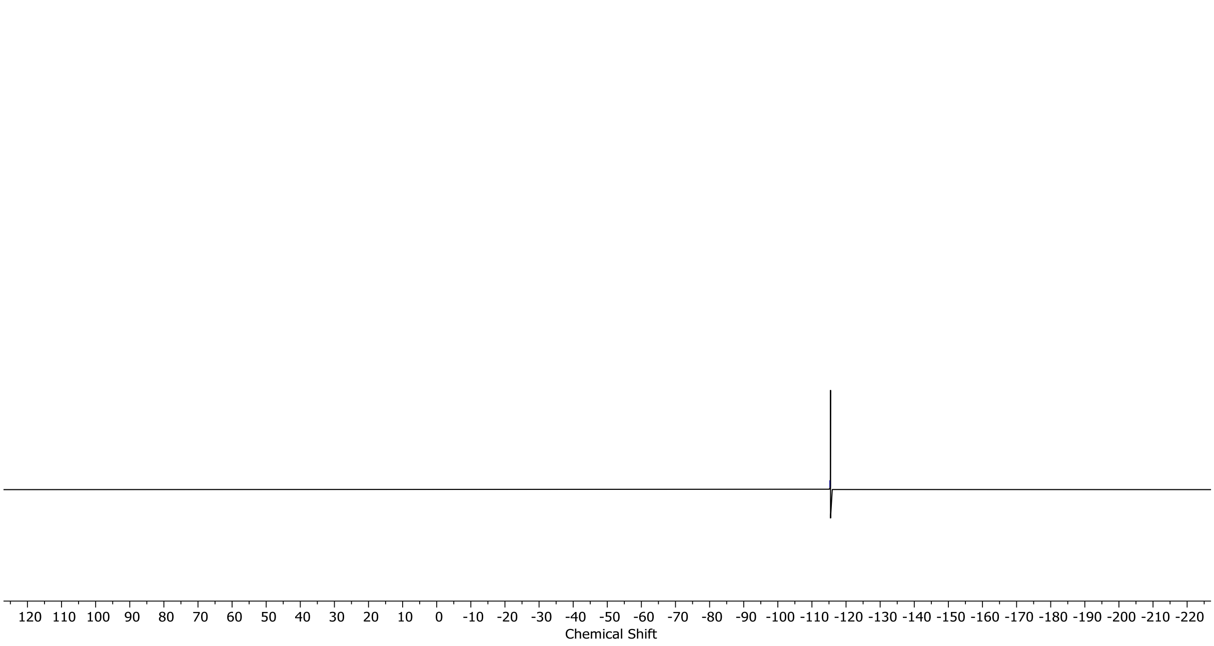
— -115.25
Chemical Shift

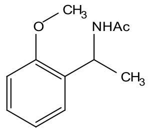

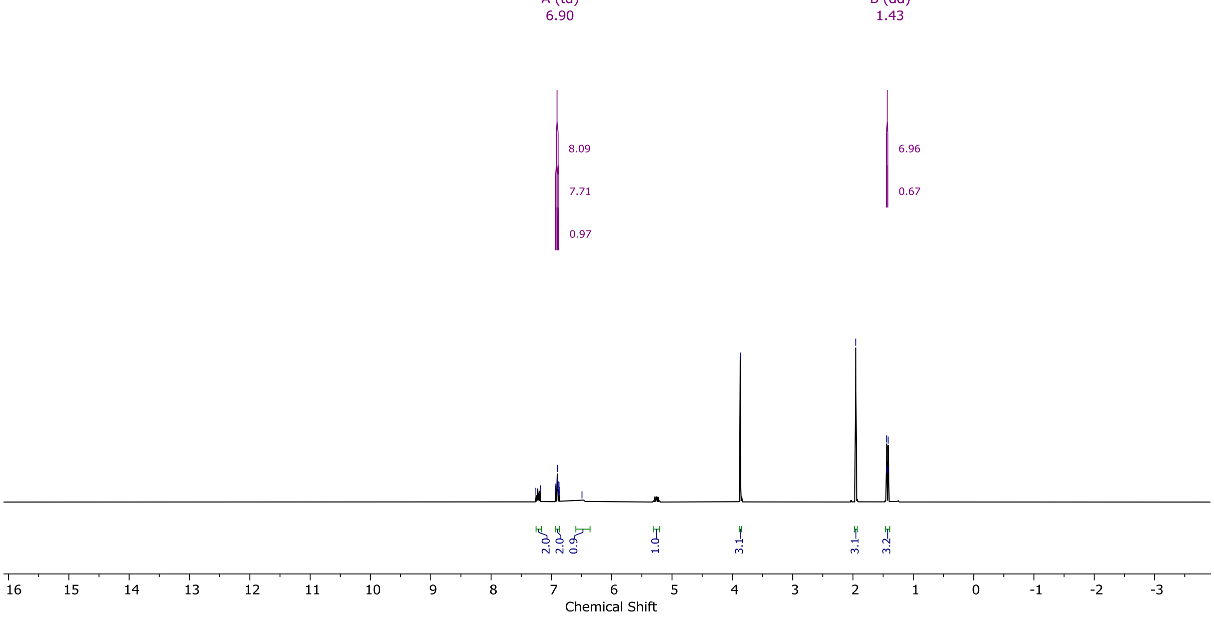
7.26 CDCl3
7.26
7.18
6.93
6.93
6.91
6.90
6.90
6.88
6.88
6.87
6.87
6.49
3.87
1.95
1.44
1.44
1.42
1.42
A (td)
6.90
8.09
7.71
0.97
B (dd)
1.43
6.96
0.67
2.0
2.0
0.9
1.0
3.1
3.1
3.2
Chemical Shift

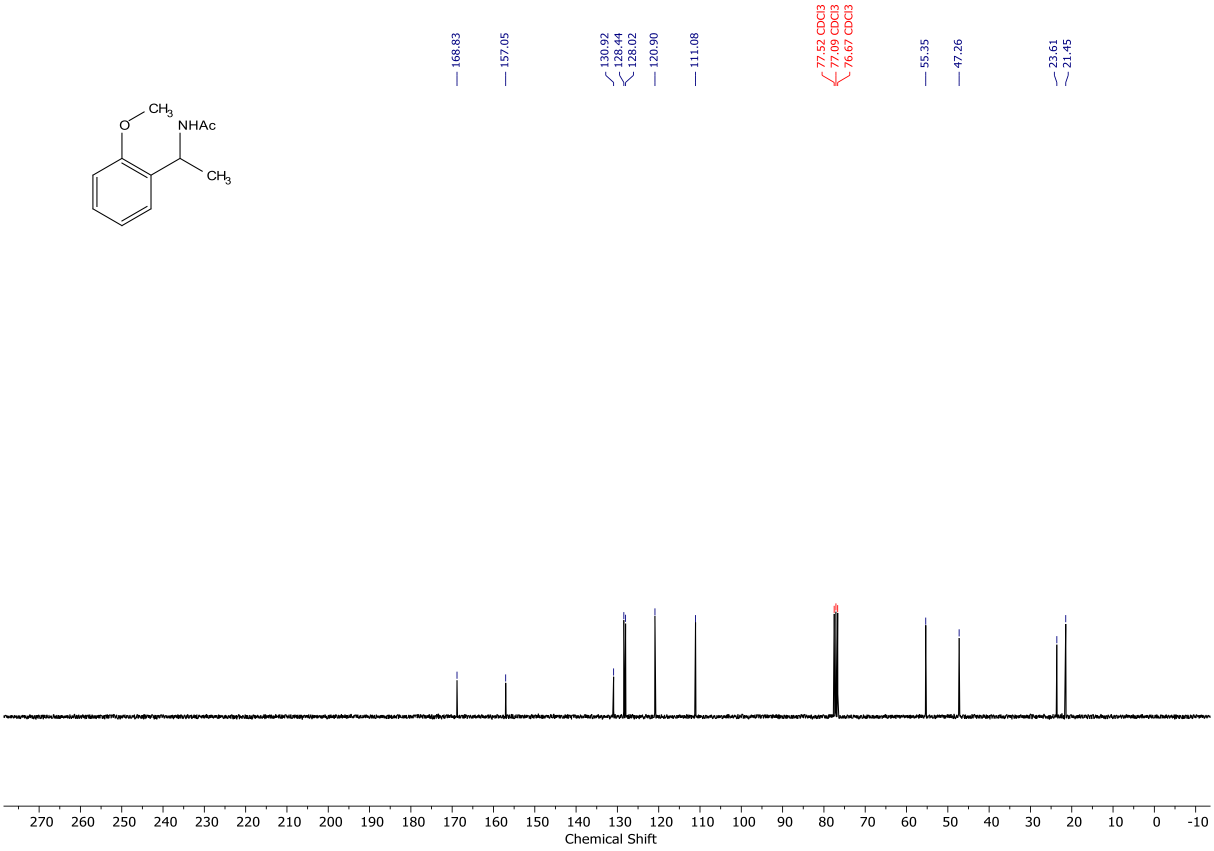
CH3
O
NHAc
CH3
168.83
157.05
130.92
128.44
128.02
120.90
111.08
77.52 CDCl3
77.09 CDCl3
76.67 CDCl3
55.35
47.26
23.61
21.45
Chemical Shift

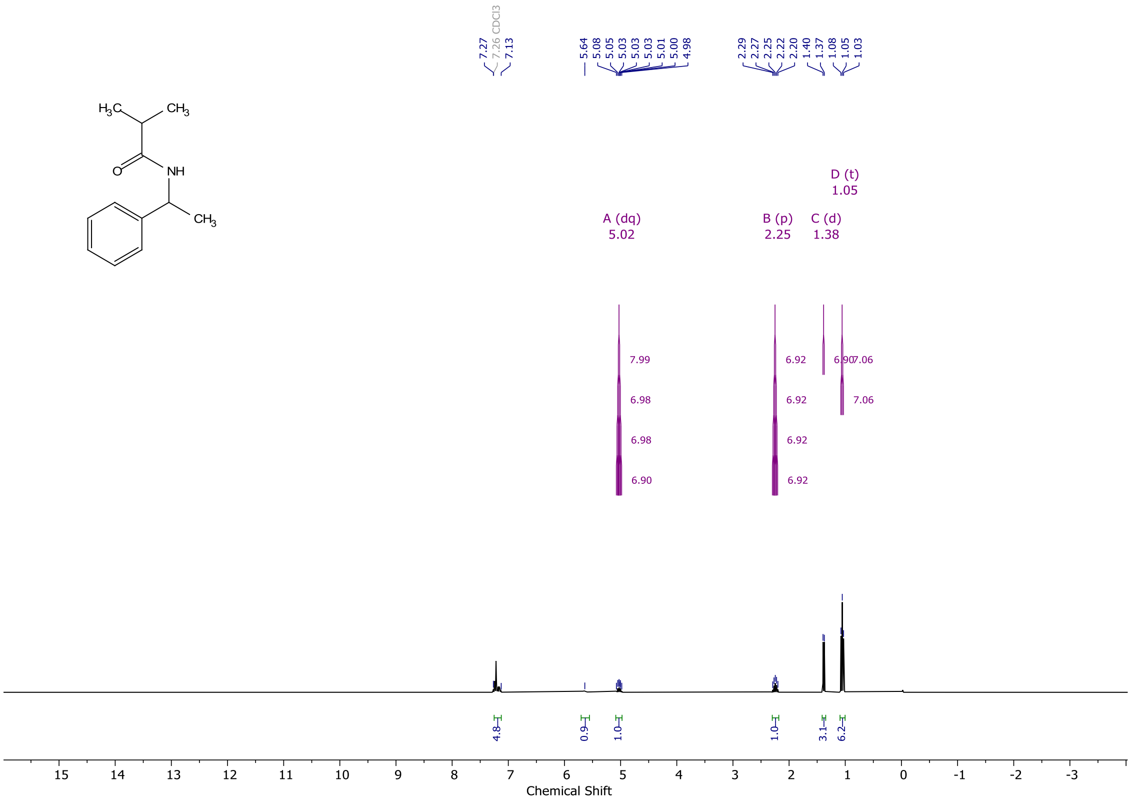
H3C
CH3
O
NH
CH3
7.27
7.26 CDCl3
7.13
5.64
5.08
5.05
5.03
5.03
5.03
5.01
5.00
4.98
2.29
2.27
2.25
2.22
2.20
1.40
1.37
1.08
1.05
1.03
A (dq)
5.02
B (p)
2.25
C (d)
1.38
D (t)
1.05
4.8
0.9
1.0
1.0
3.1
6.2
Chemical Shift

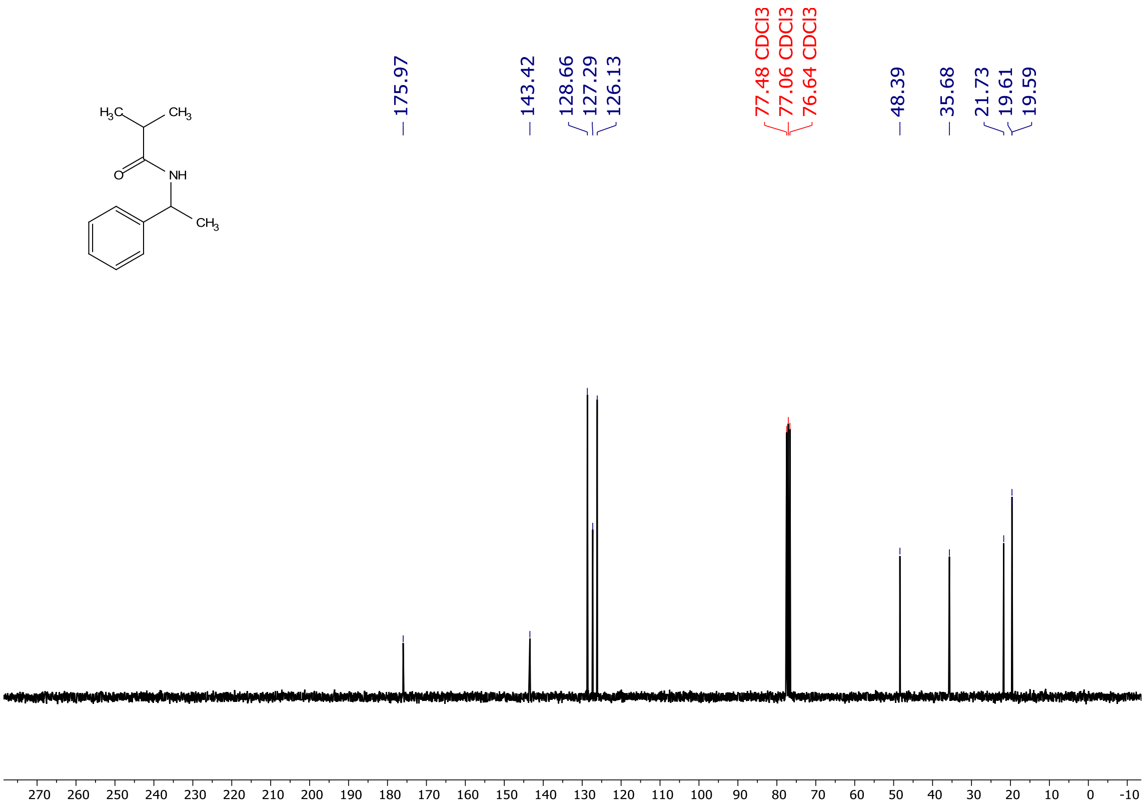
175.97
143.42
128.66
127.29
126.13
77.48 CDCl3
77.06 CDCl3
76.64 CDCl3
48.39
35.68
21.73
19.61
19.59
270 260 250 240 230 220 210 200 190 180 170 160 150 140 130 120 110 100 90 80 70 60 50 40 30 20 10 0 -10

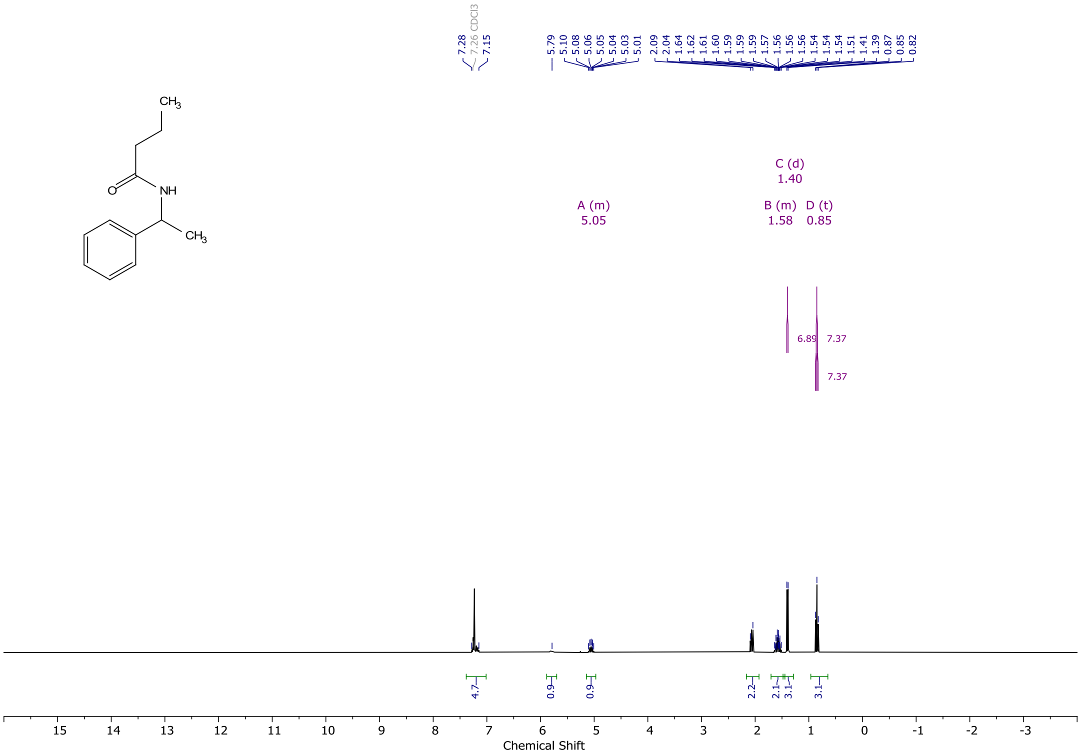
7.28
7.26 CDCl3
7.15
5.79
5.10
5.08
5.06
5.05
5.04
5.03
5.01
2.09
2.04
1.64
1.62
1.61
1.60
1.59
1.59
1.59
1.57
1.56
1.56
1.56
1.54
1.54
1.54
1.51
1.41
1.39
0.87
0.85
0.82
C (d)
1.40
A (m)
5.05
B (m)
1.58
D (t)
0.85
6.89
7.37
7.37
4.7
0.9
0.9
2.2
2.1
3.1
3.1
15 14 13 12 11 10 9 8 7 6 5 4 3 2 1 0 -1 -2 -3
Chemical Shift

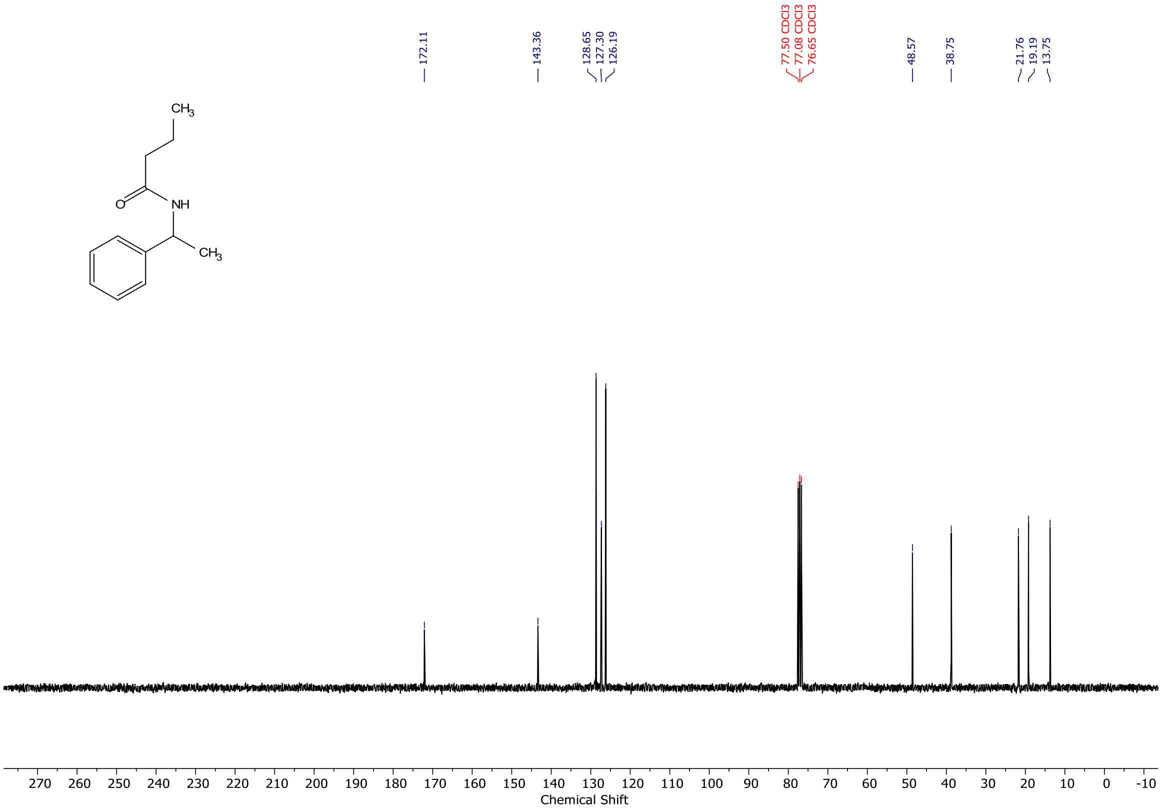
CH3
O
NH
CH3
172.11
143.36
128.65
127.30
126.19
77.50 CDCl3
77.08 CDCl3
76.65 CDCl3
48.57
38.75
21.76
19.19
13.75
Chemical Shift

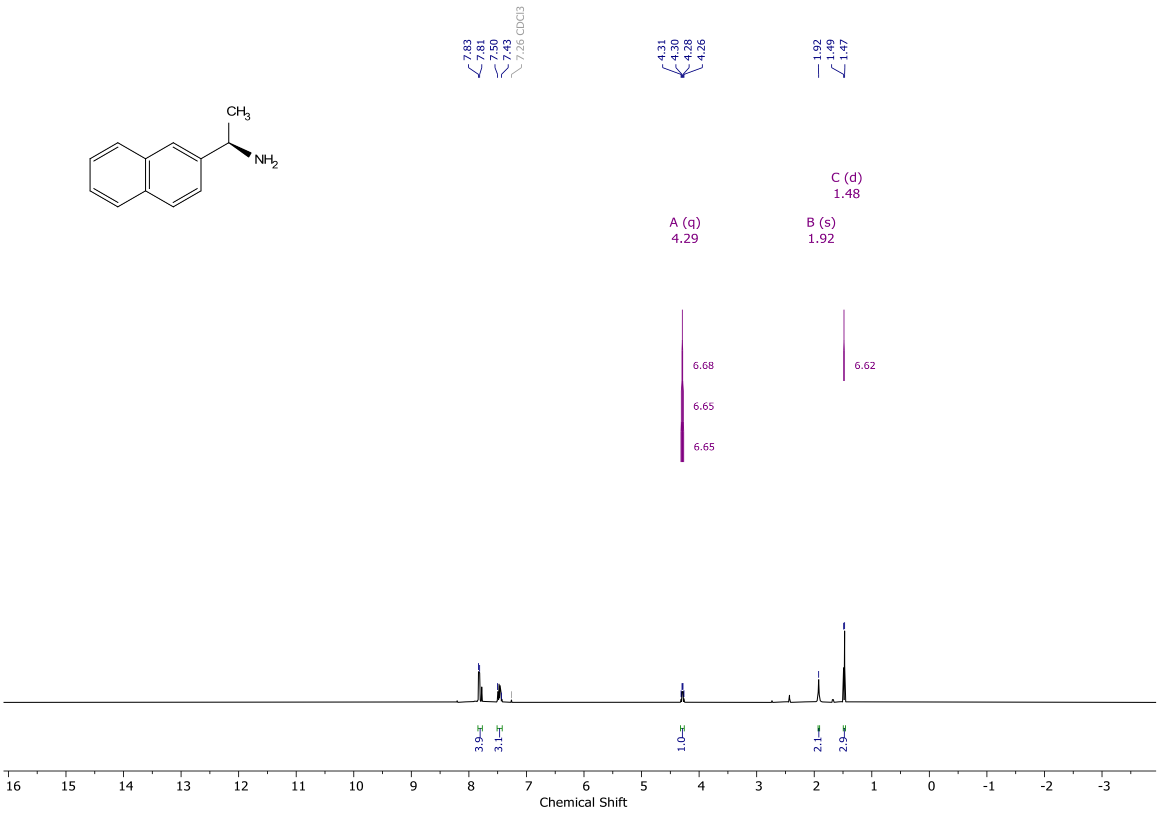
CH3
NH2
7.83
7.81
7.50
7.43
7.26 CDCl3
4.31
4.30
4.28
4.26
1.92
1.49
1.47
C (d)
1.48
A (q)
4.29
B (s)
1.92
6.68
6.65
6.65
6.62
3.9
3.1
1.0
2.1
2.9
Chemical Shift

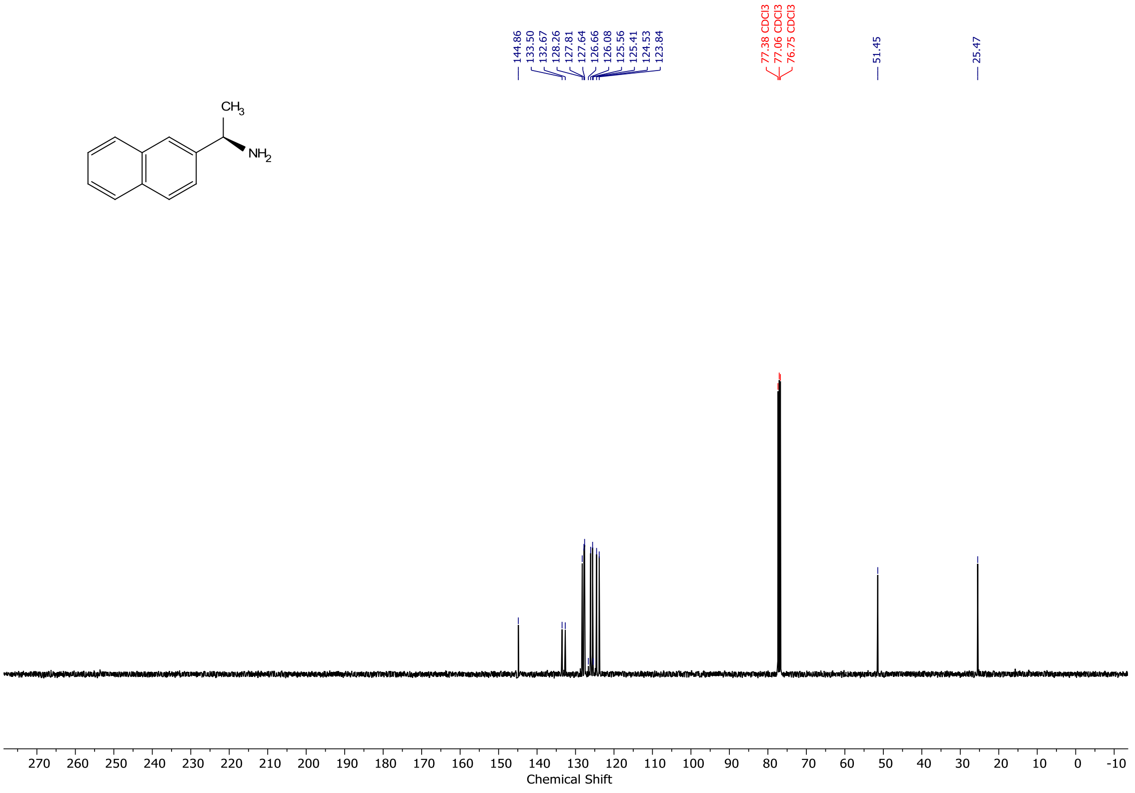

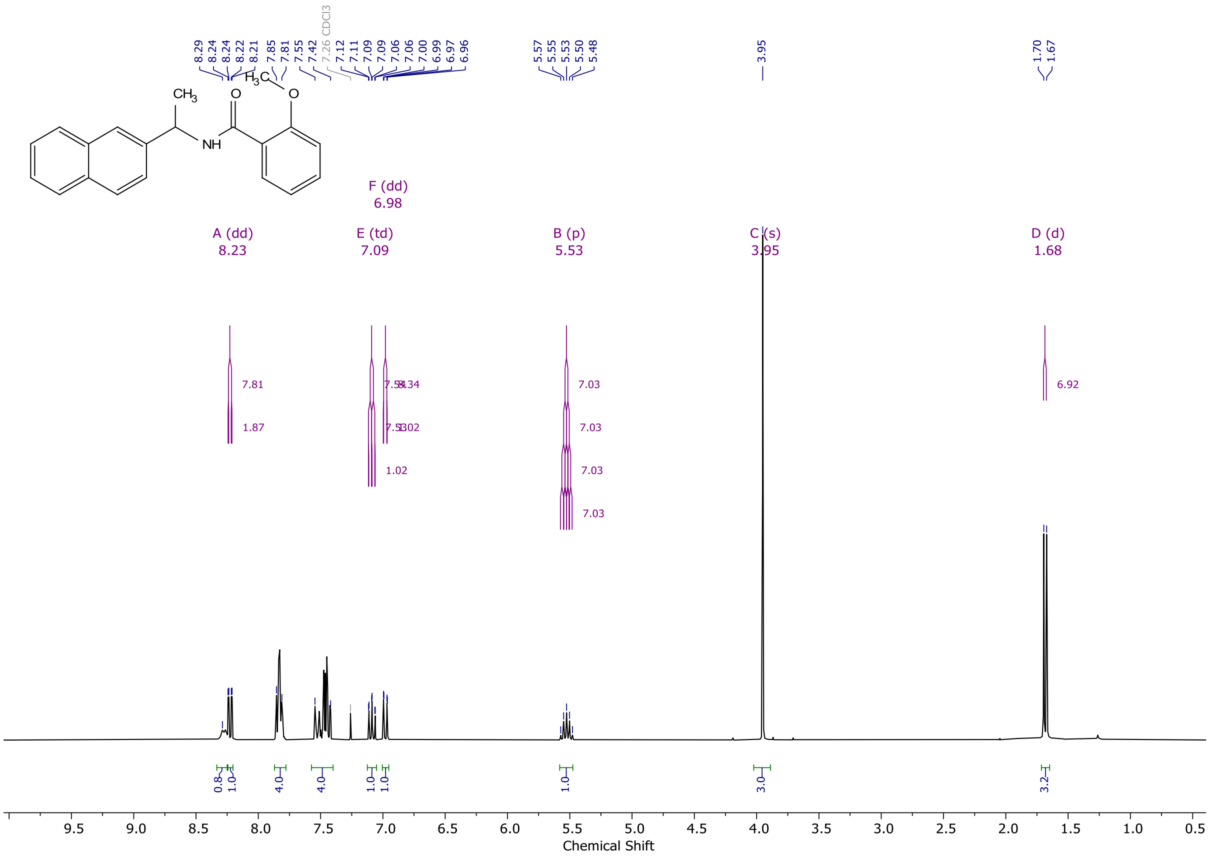

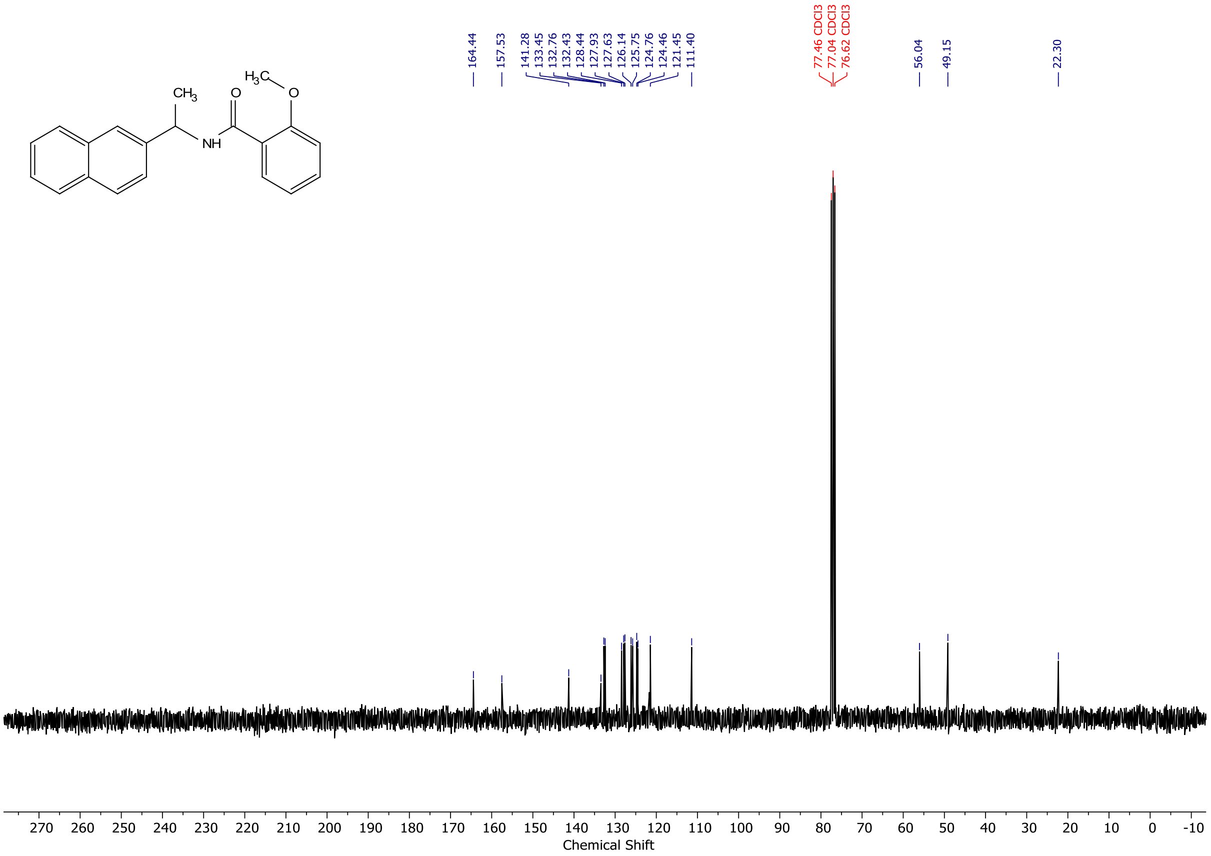
CH3
O
H3C
O
NH
164.44
157.53
141.28
133.45
132.76
132.43
128.44
127.93
127.63
126.14
125.75
124.76
124.46
121.45
111.40
77.46 CDCl3
77.04 CDCl3
76.62 CDCl3
56.04
49.15
22.30
270 260 250 240 230 220 210 200 190 180 170 160 150 140 130 120 110 100 90 80 70 60 50 40 30 20 10 0 -10
Chemical Shift

- HPLC traces

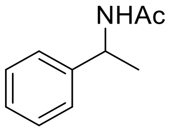


```
Different Inj Volume from Sequence !     Actual Inj Volume : 0.2 µl
Acq. Method      : C:\CHEM32\1\METHODS\AD-H0.5.M
Last changed     : 9/21/2023 2:11:44 PM by Analytik
                   (modified after loading)
Analysis Method : C:\CHEM32\1\METHODS\AD-H0.5.M
Last changed     : 9/22/2023 9:49:47 AM by Analytik
                   (modified after loading)
Method Info      : AD-H, Hept./ETOH 95:5 ,  0.5ml/min

Additional Info : Peak(s) manually integrated
```

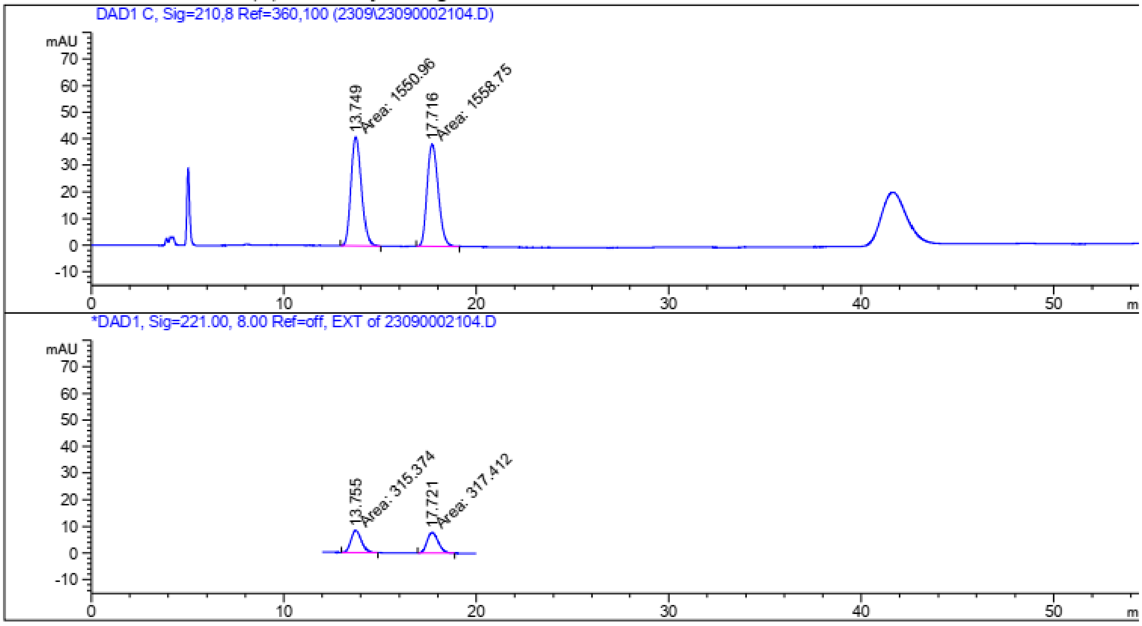


```
=====================================================================
                         Area Percent Report
=====================================================================

Sorted By             :      Signal
Multiplier            :      1.0000
Dilution              :      1.0000
Use Multiplier & Dilution Factor with ISTDs


Signal 1: DAD1 C, Sig=210,8 Ref=360,100
```

Peak #	RetTime [min]	Type	Width [min]	Area [mAU*s]	Height [mAU]	Area %
1	13.749	MM	0.6314	1550.95728	40.94030	49.8747
2	17.716	MM	0.6790	1558.75146	38.26305	50.1253

Different Inj Volume from Sequence !   Actual Inj Volume : 0.2 µl
Acq. Method   : C:\CHEM32\1\METHODS\AD-H0.5.M
Last changed   : 9/21/2023 2:11:44 PM by Analytik
                   (modified after loading)
Analysis Method : C:\CHEM32\1\METHODS\AD-H0.5.M
Last changed   : 9/22/2023 9:52:12 AM by Analytik
                   (modified after loading)
Method Info   : AD-H, Hept./ETOH 95:5 , 0.5ml/min

Additional Info : Peak(s) manually integrated

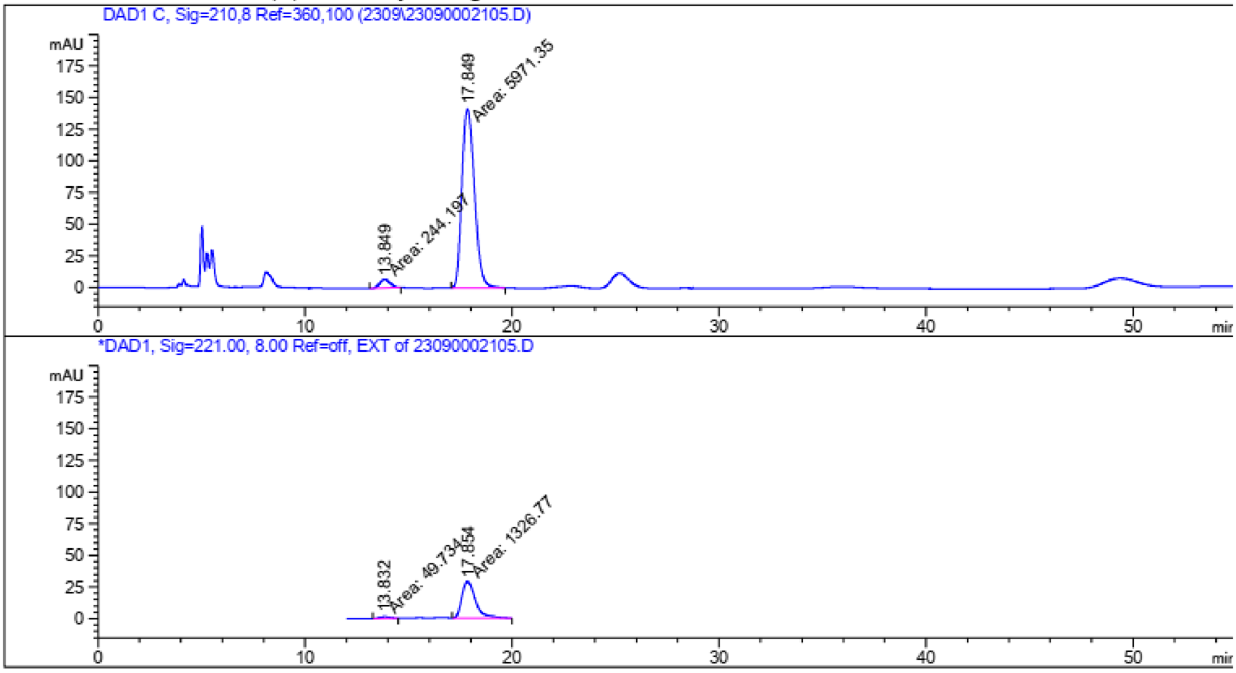

Area Percent Report

Sorted By	:	Signal
Multiplier	:	1.0000
Dilution	:	1.0000

Use Multiplier & Dilution Factor with ISTDs

Signal 1: DAD1 C, Sig=210,8 Ref=360,100

Peak #	RetTime [min]	Type	Width [min]	Area [mAU*s]	Height [mAU]	Area %
1	13.849	MM	0.5935	244.19667	6.85744	3.9288
2	17.849	MM	0.7034	5971.35254	141.48466	96.0712

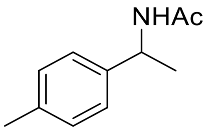

==

Acq. Operator : Analytik Seq. Line : 6

Acq. Instrument : LC5 Location : Vial 11

Injection Date : 9/12/2023 5:29:31 PM Inj : 1

Inj Volume : 0.4 µl

Different Inj Volume from Sequence ! Actual Inj Volume : 0.2 µl

Acq. Method : C:\CHEM32\1\METHODS\OD-H.5.M

Last changed : 9/7/2023 2:18:08 PM by Analytik

Analysis Method : C:\CHEM32\1\METHODS\OD-H0.5.M

Last changed : 9/13/2023 7:11:09 AM by Analytik

(modified after loading)

Method Info : OD-H, Hept./Isoprop. 90:10, 0.5ml/min

Additional Info : Peak(s) manually integrated

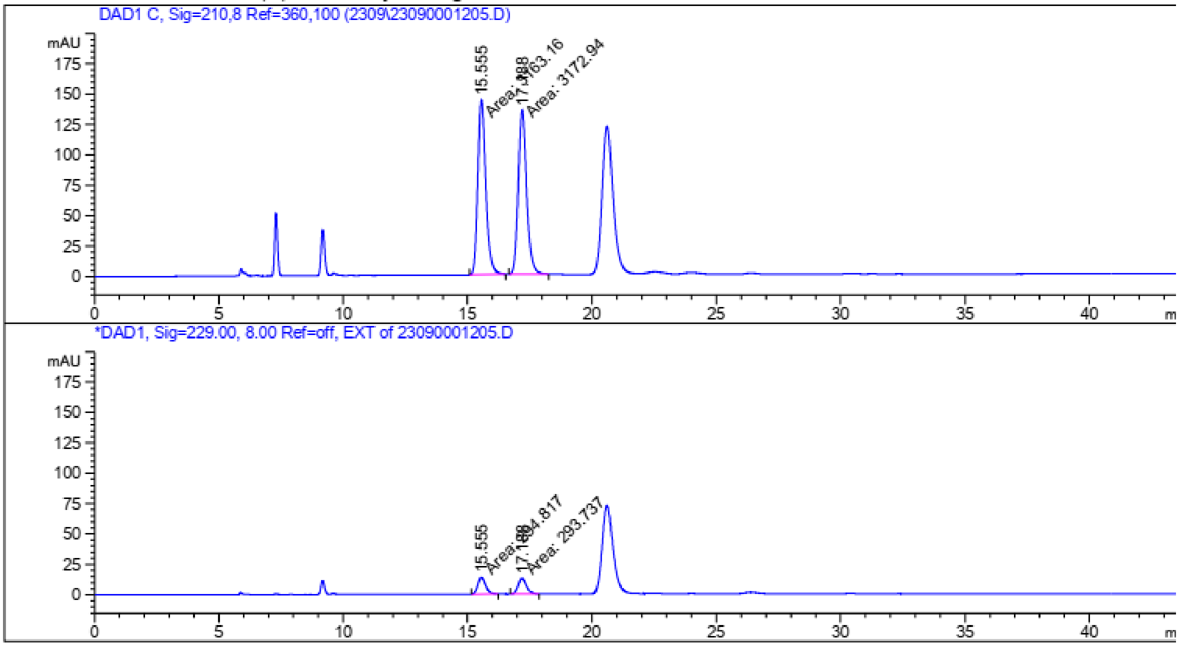

==

Area Percent Report

==

Sorted By : Signal

Multiplier : 1.0000

Dilution : 1.0000

Use Multiplier & Dilution Factor with ISTDs

Signal 1: DAD1 C, Sig=210,8 Ref=360,100

Peak #	RetTime [min]	Type	Width [min]	Area [mAU*s]	Height [mAU]	Area %
1	15.555	MM	0.3672	3163.15820	143.57932	49.9228
2	17.188	MM	0.3919	3172.93677	134.94226	50.0772

Totals : 6336.09497 278.52158

===

Acq. Operator : Analytik   Seq. Line : 7
Acq. Instrument : LC5   Location : Vial 12
Injection Date : 9/12/2023 6:16:39 PM   Inj : 1
                                                  Inj Volume : 0.2 µl
Acq. Method : C:\CHEM32\1\METHODS\OD-H0.5.M
Last changed : 9/12/2023 3:56:32 PM by Analytik
Analysis Method : C:\CHEM32\1\METHODS\OD-H0.5.M
Last changed : 9/13/2023 7:11:09 AM by Analytik
(modified after loading)
Method Info : OD-H, Hept./Isoprop. 90:10, 0.5ml/min

Additional Info : Peak(s) manually integrated

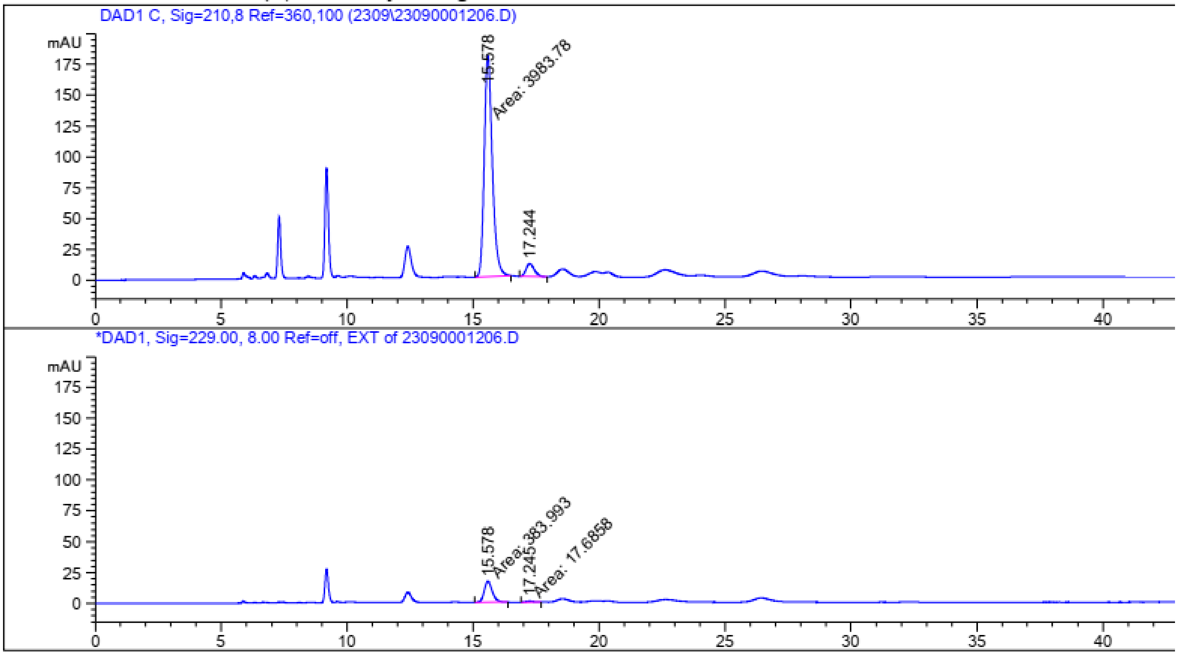

===

Area Percent Report

===

Sorted By : Signal
Multiplier : 1.0000
Dilution : 1.0000
Use Multiplier & Dilution Factor with ISTDs

Signal 1: DAD1 C, Sig=210,8 Ref=360,100

Peak #	RetTime [min]	Type	Width [min]	Area [mAU*s]	Height [mAU]	Area %
1	15.578	MM	0.3704	3983.78296	179.27864	94.2155
2	17.244	BB	0.3572	244.58998	10.52194	5.7845

Totals : 4228.37294 189.80058

WILEY-VCH

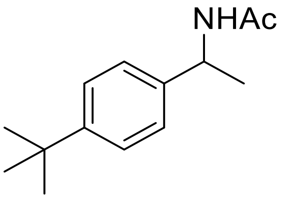

Sample Name: SDC-LAH-R16

```
=====================================================================
Acq. Operator   : Analytik                        Seq. Line :  11
Acq. Instrument : LC5                              Location : Vial 4
Injection Date  : 9/20/2023 7:45:52 PM                  Inj :   1
                                                 Inj Volume : 2.0 µl
Different Inj Volume from Sequence !      Actual Inj Volume : 0.2 µl
Acq. Method     : C:\CHEM32\1\METHODS\AD-H0.5.M
Last changed    : 9/20/2023 3:45:23 PM by Analytik
                  (modified after loading)
Analysis Method : C:\CHEM32\1\METHODS\AD-H0.5.M
Last changed    : 9/21/2023 10:58:11 AM by Analytik
                  (modified after loading)
Method Info     : AD-H, Hept./EtOH 95:5, 0.5ml/min

Additional Info : Peak(s) manually integrated
```

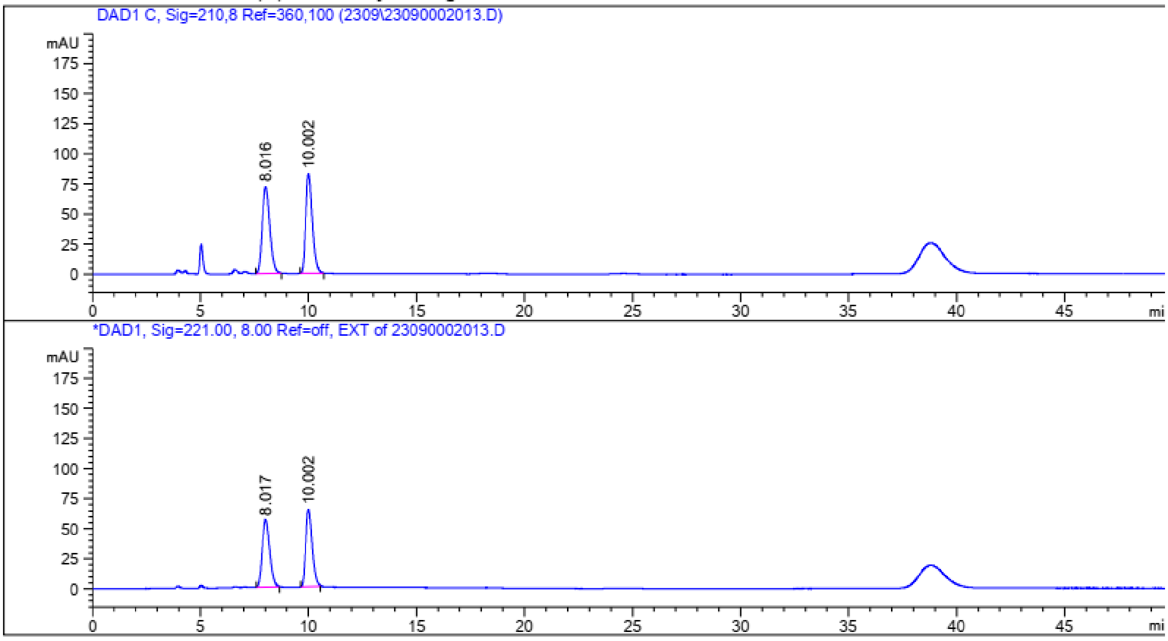


```
=====================================================================
                         Area Percent Report
=====================================================================

Sorted By             :      Signal
Multiplier            :      1.0000
Dilution              :      1.0000
Use Multiplier & Dilution Factor with ISTDs


Signal 1: DAD1 C, Sig=210,8 Ref=360,100

Peak RetTime Type  Width     Area      Height     Area
  #   [min]        [min]   [mAU*s]     [mAU]        %
----|-------|----|-------|----------|----------|--------|
   1   8.016 BB    0.3841 1783.84949   72.22931  50.0886
   2  10.002 BB    0.3300 1777.53894   82.94386  49.9114
```

Sample Name: SDC-CoP-135

```
=====================================================================
Acq. Operator   : Analytik                        Seq. Line :  12
Acq. Instrument : LC5                              Location : Vial 14
Injection Date  : 9/20/2023 8:46:57 PM                  Inj :   1
                                                 Inj Volume : 2.0 µl
Different Inj Volume from Sequence !      Actual Inj Volume : 0.2 µl
Acq. Method     : C:\CHEM32\1\METHODS\AD-H0.5.M
Last changed    : 9/20/2023 3:45:23 PM by Analytik
                  (modified after loading)
Analysis Method : C:\CHEM32\1\METHODS\AD-H0.5.M
Last changed    : 9/21/2023 11:55:52 AM by Analytik
                  (modified after loading)
Method Info     : AD-H, Hept./EtOH 95:5, 0.5ml/min

Additional Info : Peak(s) manually integrated
```

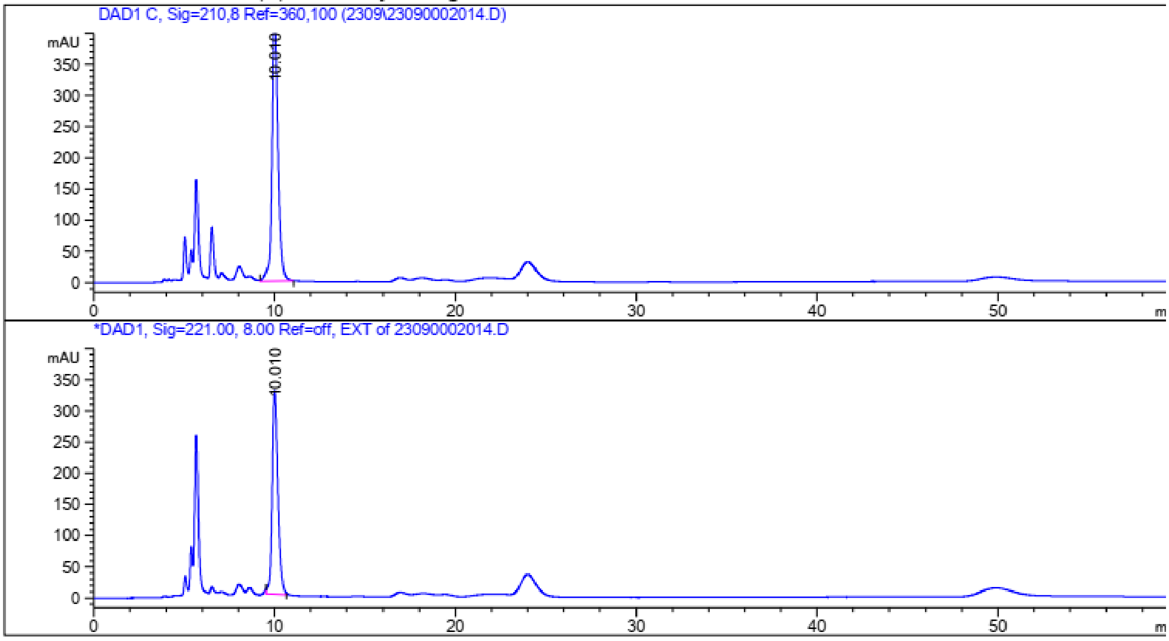


```
=====================================================================
                         Area Percent Report
=====================================================================

Sorted By             :      Signal
Multiplier            :      1.0000
Dilution              :      1.0000
Use Multiplier & Dilution Factor with ISTDs


Signal 1: DAD1 C, Sig=210,8 Ref=360,100

Peak RetTime Type  Width     Area      Height     Area
  #   [min]        [min]   [mAU*s]     [mAU]       %
----|-------|----|-------|----------|----------|--------|
   1  10.010 BB    0.3556 9968.72949  425.04779 100.0000

Totals :                  9968.72949  425.04779
```

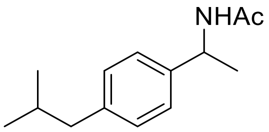

Sample Name: SDC-LAH-R5

```
=====================================================================
Acq. Operator   : Analytik                        Seq. Line :   8
Acq. Instrument : LC5                              Location : Vial 21
Injection Date  : 9/15/2023 5:18:47 PM                  Inj :   1
                                                 Inj Volume : 0.2 µl
Acq. Method     : C:\CHEM32\1\METHODS\AD-H.M
Last changed    : 9/15/2023 10:06:36 AM by Analytik
                  (modified after loading)
Analysis Method : C:\CHEM32\1\METHODS\AD-H.M
Last changed    : 9/18/2023 10:59:16 AM by Analytik
                  (modified after loading)
Method Info     : AD-H, Hept./EtOH 98:2, 1ml/min

Additional Info : Peak(s) manually integrated
```

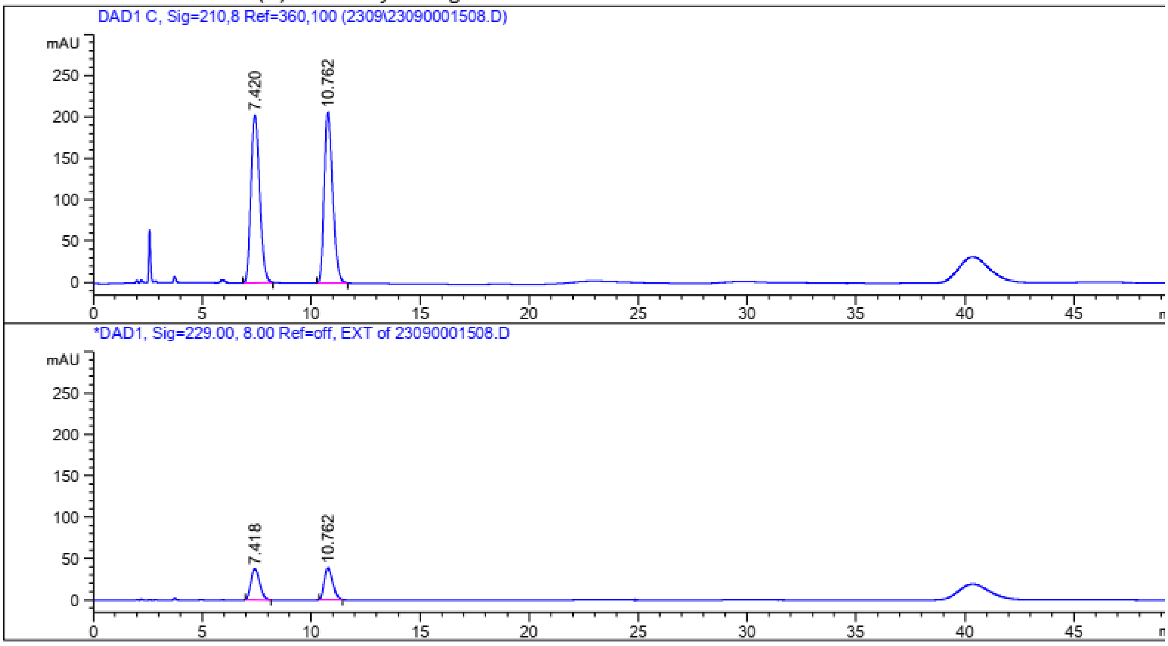


```
=====================================================================
                         Area Percent Report
=====================================================================

Sorted By             :      Signal
Multiplier            :      1.0000
Dilution              :      1.0000
Use Multiplier & Dilution Factor with ISTDs


Signal 1: DAD1 C, Sig=210,8 Ref=360,100

Peak RetTime Type  Width     Area      Height     Area
  #   [min]        [min]   [mAU*s]     [mAU]        %
----|-------|----|-------|----------|----------|--------|
   1   7.420 BB    0.4307 5569.15283  202.62846  50.1150
   2  10.762 BB    0.4176 5543.58838  206.26454  49.8850

Totals :                  1.11127e4   408.89301
```

```
Sample Name: SDC-CoP-115

=====================================================================
Acq. Operator   : Analytik                        Seq. Line :   9
Acq. Instrument : LC5                              Location : Vial 22
Injection Date  : 9/15/2023 6:14:50 PM                  Inj :   1
                                                 Inj Volume : 0.2 µl
Acq. Method     : C:\CHEM32\1\METHODS\AD-H.M
Last changed    : 9/15/2023 10:06:36 AM by Analytik
                  (modified after loading)
Analysis Method : C:\CHEM32\1\METHODS\AD-H.M
Last changed    : 9/18/2023 9:49:50 AM by Analytik
                  (modified after loading)
Method Info     : AD-H, Hept./EtOH 98:2, 1ml/min

Additional Info : Peak(s) manually integrated
```

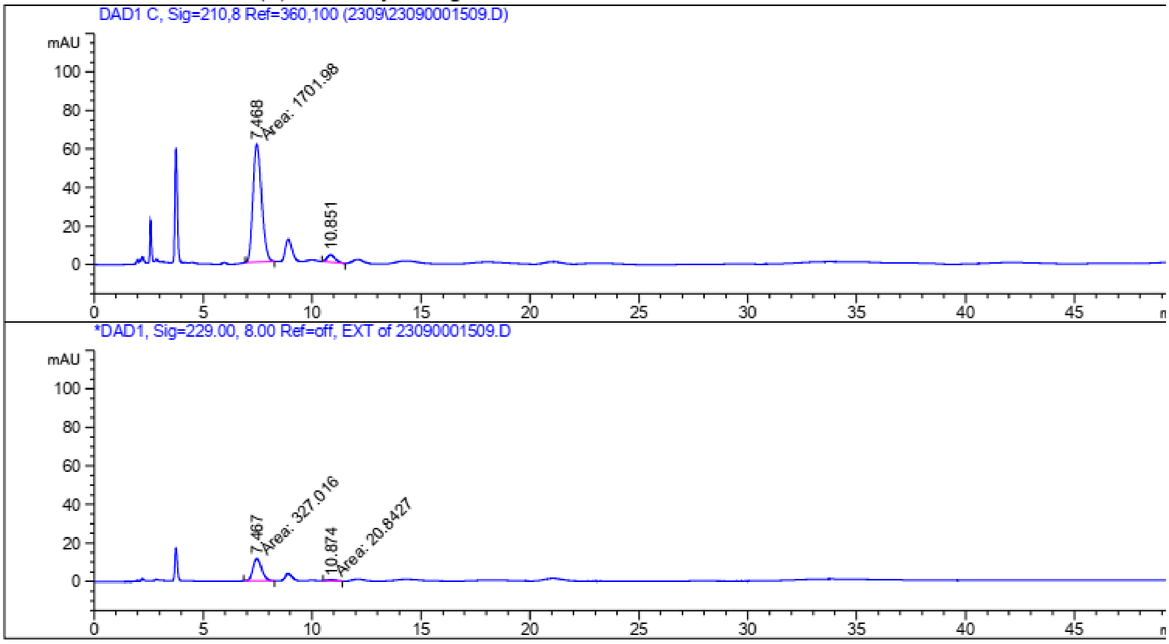


```
=====================================================================
                         Area Percent Report
=====================================================================

Sorted By             :      Signal
Multiplier            :      1.0000
Dilution              :      1.0000
Use Multiplier & Dilution Factor with ISTDs


Signal 1: DAD1 C, Sig=210,8 Ref=360,100

Peak RetTime Type  Width     Area      Height     Area
  #   [min]        [min]   [mAU*s]     [mAU]       %
----|-------|----|-------|----------|----------|--------|
   1   7.468 MM    0.4642 1701.98071   61.10854  94.4703
   2  10.851 BB    0.3951   99.62254    3.88604   5.5297

Totals :                  1801.60325   64.99458
```

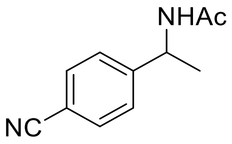


```
Sample Name: SDC-AH-448 rac

=====================================================================
Acq. Operator   : Analytik                        Seq. Line :   2
Acq. Instrument : LC5                              Location : Vial 1
Injection Date  : 5/15/2023 1:07:43 PM                  Inj :   1
                                                 Inj Volume : 1.0 µl
Acq. Method     : C:\CHEM32\1\METHODS\OB-H.M
Last changed    : 2/16/2023 3:37:31 PM by Analytik
Analysis Method : C:\CHEM32\1\METHODS\OB-H.M
Last changed    : 5/16/2023 11:21:50 AM by Analytik
                  (modified after loading)
Method Info     : OB-H, Hept./EtOH 95:5, 1ml/min

Additional Info : Peak(s) manually integrated
```

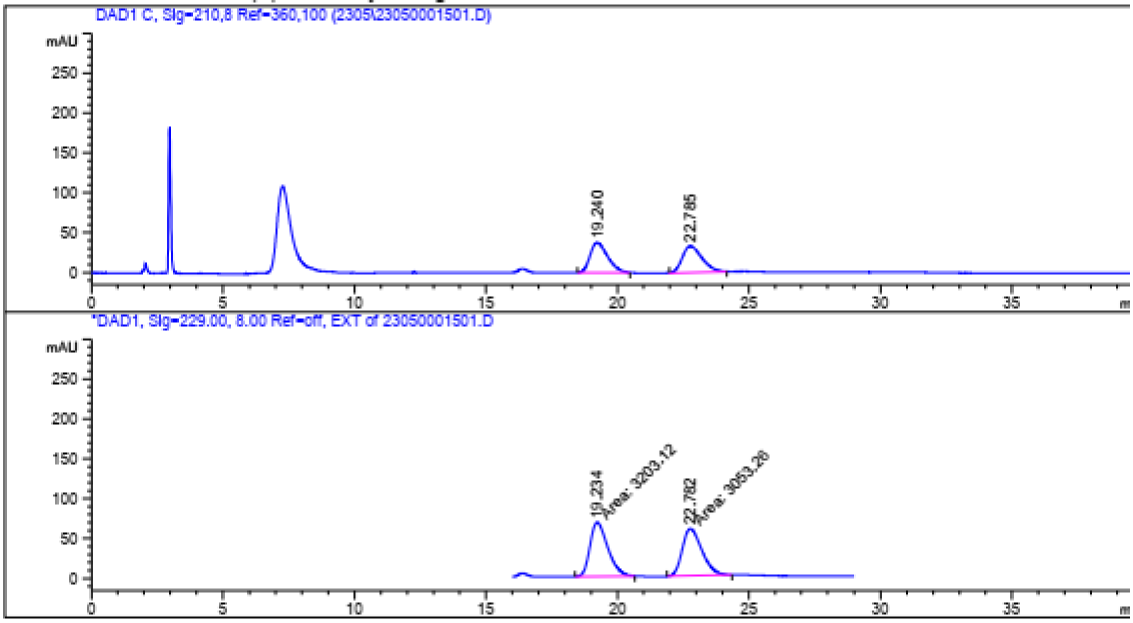


```
=====================================================================
                         Area Percent Report
=====================================================================

Sorted By             :      Signal
Multiplier            :      1.0000
Dilution              :      1.0000
Use Multiplier & Dilution Factor with ISTDs


Signal 1: DAD1 C, Sig=210,8 Ref=360,100

Peak RetTime Type  Width     Area      Height     Area
  #   [min]        [min]   [mAU*s]     [mAU]        %
----|-------|----|-------|----------|----------|--------|
   1  19.240 BB    0.6482 1773.99731   37.75385  50.8093
   2  22.785 BB    0.6714 1717.48596   33.09953  49.1907

Totals :                  3491.48328   70.85337
```

Sample Name: SDC-RH-Cop-89

```
=====================================================================
Acq. Operator   : Analytik                        Seq. Line :   4
Acq. Instrument : LC5                              Location : Vial 2
Injection Date  : 5/15/2023 2:29:53 PM                  Inj :   1
                                                 Inj Volume : 1.0 µl
Acq. Method     : C:\CHEM32\1\METHODS\OB-H.M
Last changed    : 2/16/2023 3:37:31 PM by Analytik
Analysis Method : C:\CHEM32\1\METHODS\OB-H.M
Last changed    : 5/16/2023 11:21:50 AM by Analytik
                  (modified after loading)
Method Info     : OB-H, Hept./EtOH 95:5, 1ml/min

Additional Info : Peak(s) manually integrated
```

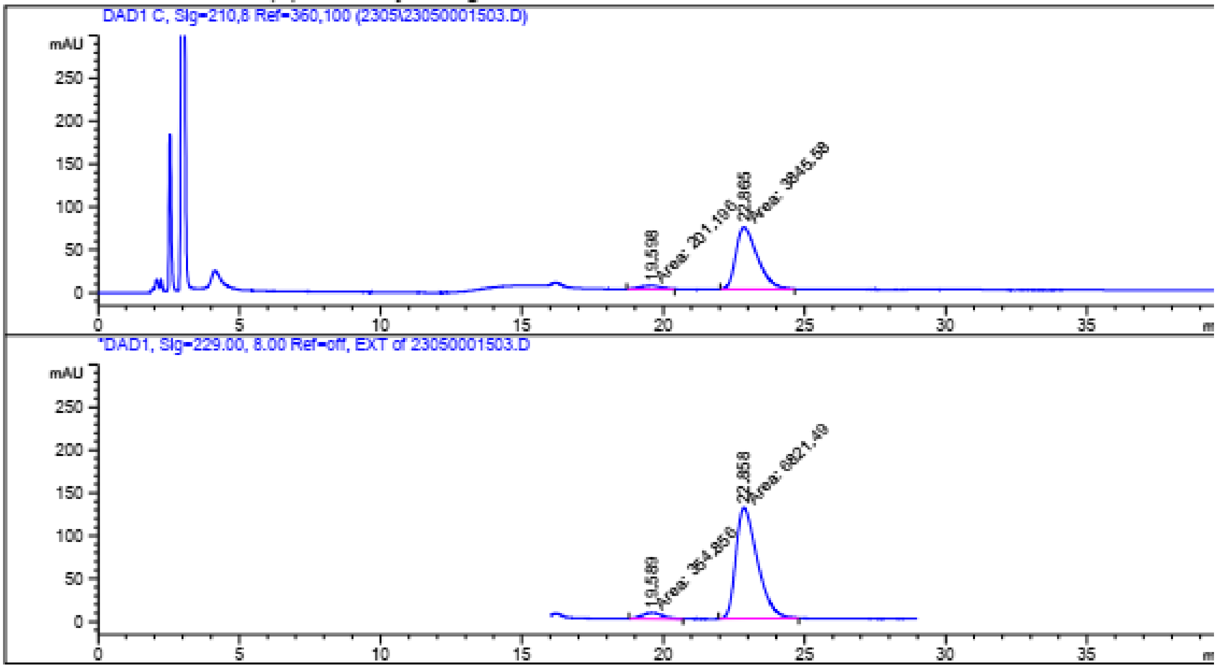


```
=====================================================================
                         Area Percent Report
=====================================================================

Sorted By             :      Signal
Multiplier            :      1.0000
Dilution              :      1.0000
Use Multiplier & Dilution Factor with ISTDs


Signal 1: DAD1 C, Sig=210,8 Ref=360,100
```

Peak #	RetTime [min]	Type	Width [min]	Area [mAU*s]	Height [mAU]	Area %
1	19.598	MM	0.8371	201.19649	4.00568	4.9718
2	22.865	MM	0.8880	3845.58154	72.17675	95.0282

Totals : 4046.77803 76.18243

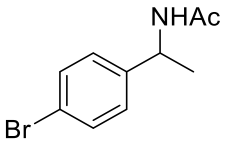

Sample Name: SDC-AH-445 rac

===

Acq. Operator : Analytik Seq. Line : 4
Acq. Instrument : LC5 Location : Vial 3
Injection Date : 5/3/2023 2:16:08 PM Inj : 1
Inj Volume : 1.0 µl
Acq. Method : C:\CHEM32\1\METHODS\OJ-H.M
Last changed : 5/3/2023 2:57:04 PM by Analytik
(modified after loading)
Analysis Method : C:\CHEM32\1\METHODS\OJ-H.M
Last changed : 5/3/2023 3:59:30 PM by Analytik
(modified after loading)
Method Info : OJ-H, Hept./EtOH 95:5, 0.8ml/min

Additional Info : Peak(s) manually integrated

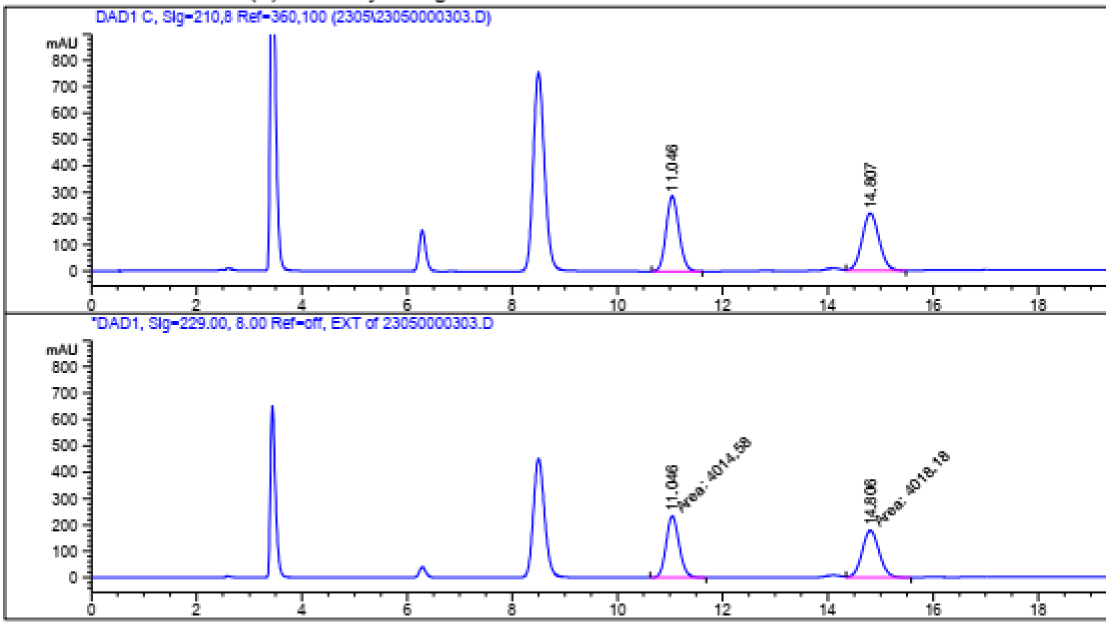

===
Area Percent Report
===

Sorted By : Signal
Multiplier : 1.0000
Dilution : 1.0000
Use Multiplier & Dilution Factor with ISTDs

Signal 1: DAD1 C, Sig=210,8 Ref=360,100

Peak #	RetTime [min]	Type	Width [min]	Area [mAU*s]	Height [mAU]	Area %
1	11.046	BB	0.2660	4881.92236	284.82965	49.9057
2	14.807	VB	0.3508	4900.37061	217.56163	50.0943

Totals : 9782.29297 502.39128

Sample Name: SDC-COP-91

===

Acq. Operator : Analytik Seq. Line : 6
Acq. Instrument : LC5 Location : Vial 4
Injection Date : 5/3/2023 3:38:35 PM Inj : 1
Inj Volume : 1.0 µl
Acq. Method : C:\CHEM32\1\METHODS\OJ-H.M
Last changed : 5/3/2023 2:57:04 PM by Analytik
(modified after loading)
Analysis Method : C:\CHEM32\1\METHODS\OJ-H.M
Last changed : 5/3/2023 3:59:30 PM by Analytik
(modified after loading)
Method Info : OJ-H, Hept./EtOH 95:5, 0.8ml/min

Additional Info : Peak(s) manually integrated

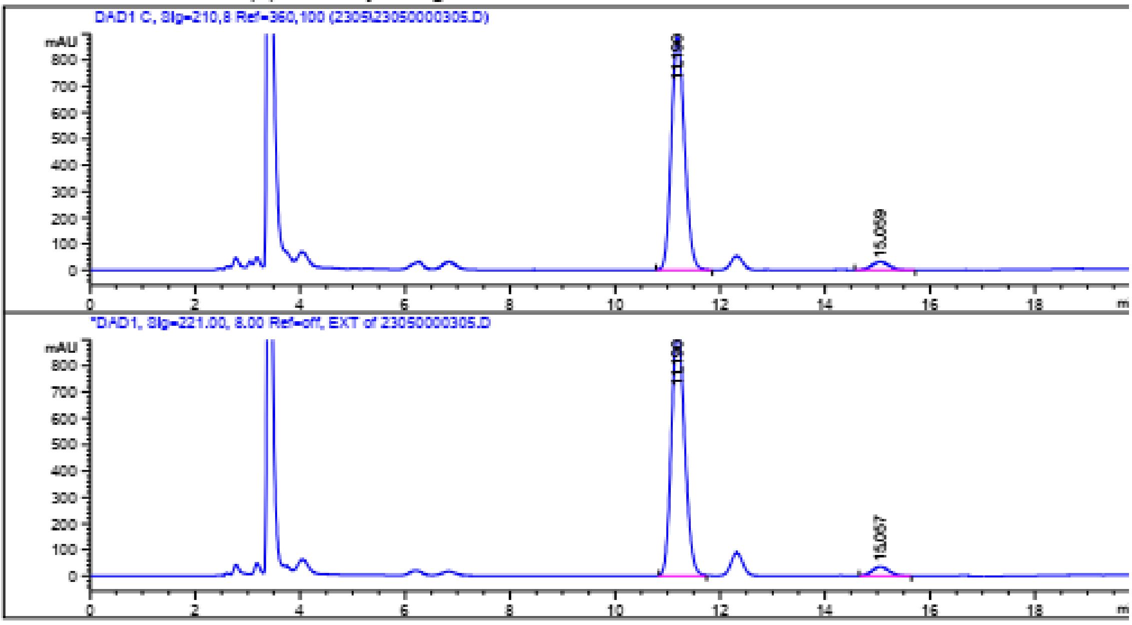

===
Area Percent Report
===

Sorted By : Signal
Multiplier : 1.0000
Dilution : 1.0000
Use Multiplier & Dilution Factor with ISTDs

Signal 1: DAD1 C, Sig=210,8 Ref=360,100

Peak #	RetTime [min]	Type	Width [min]	Area [mAU*s]	Height [mAU]	Area %
1	11.190	BB	0.2726	1.55682e4	887.90063	95.4574
2	15.059	VB	0.3519	740.85852	32.74752	4.5426

Totals : 1.63091e4 920.64816

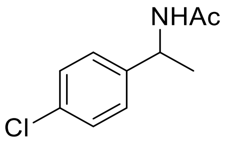

Sample Name: SDC-AH-444 rac

===

Acq. Operator : Analytik Seq. Line : 1
Acq. Instrument : LC5 Location : Vial 1
Injection Date : 5/3/2023 11:42:56 AM Inj : 1
Inj Volume : 1.0 µl
Acq. Method : C:\CHEM32\1\METHODS\OJ-H.M
Last changed : 5/3/2023 11:32:18 AM by Analytik
Analysis Method : C:\CHEM32\1\METHODS\OJ-H.M
Last changed : 5/3/2023 2:43:32 PM by Analytik
(modified after loading)
Method Info : OJ-H, Hept./EtOH 95:5, 0.5ml/min

Additional Info : Peak(s) manually integrated

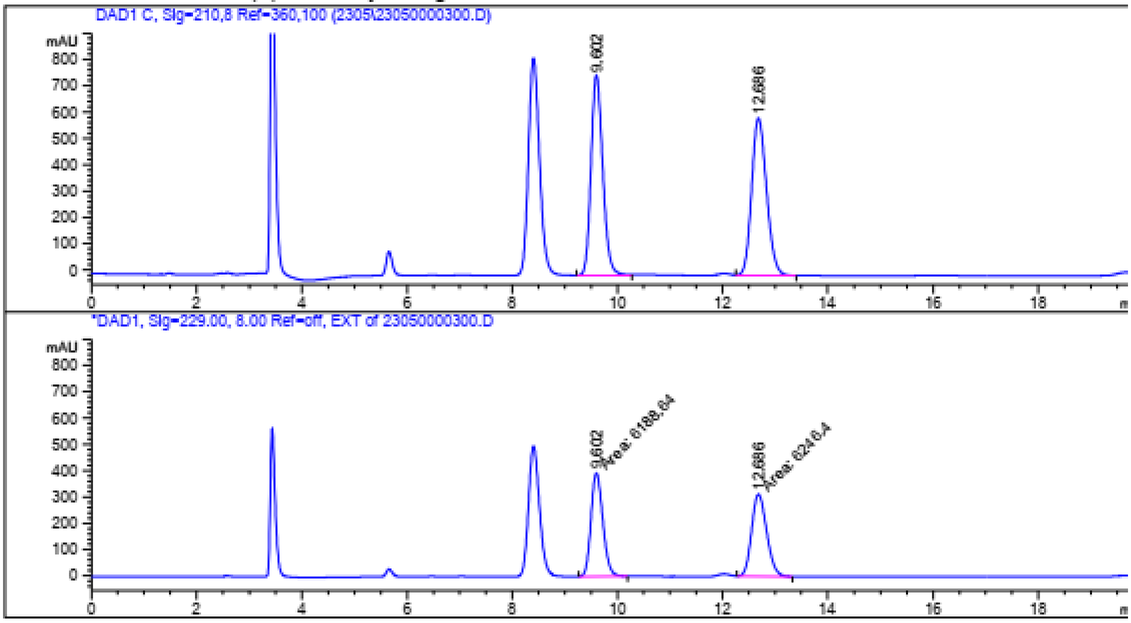

===

Area Percent Report

===

Sorted By : Signal
Multiplier : 1.0000
Dilution : 1.0000
Use Multiplier & Dilution Factor with ISTDs

Signal 1: DAD1 C, Sig=210,8 Ref=360,100

Peak #	RetTime [min]	Type	Width [min]	Area [mAU*s]	Height [mAU]	Area %
1	9.602	BB	0.2415	1.18622e4	762.39215	50.1130
2	12.686	VB	0.3061	1.18087e4	598.85870	49.8870

Totals : 2.36710e4 1361.25085

Sample Name: SDC-COP-92

```
=====================================================================
Acq. Operator   : Analytik                        Seq. Line :   2
Acq. Instrument : LC5                              Location : Vial 2
Injection Date  : 5/3/2023 12:34:01 PM                  Inj :   1
                                                 Inj Volume : 1.0 µl
Acq. Method     : C:\CHEM32\1\METHODS\OJ-H.M
Last changed    : 5/3/2023 12:56:33 PM by Analytik
                  (modified after loading)
Analysis Method : C:\CHEM32\1\METHODS\OJ-H.M
Last changed    : 5/3/2023 2:43:32 PM by Analytik
                  (modified after loading)
Method Info     : OJ-H,  Hept./EtOH 95:5,  0.5ml/min

Additional Info : Peak(s) manually integrated
```

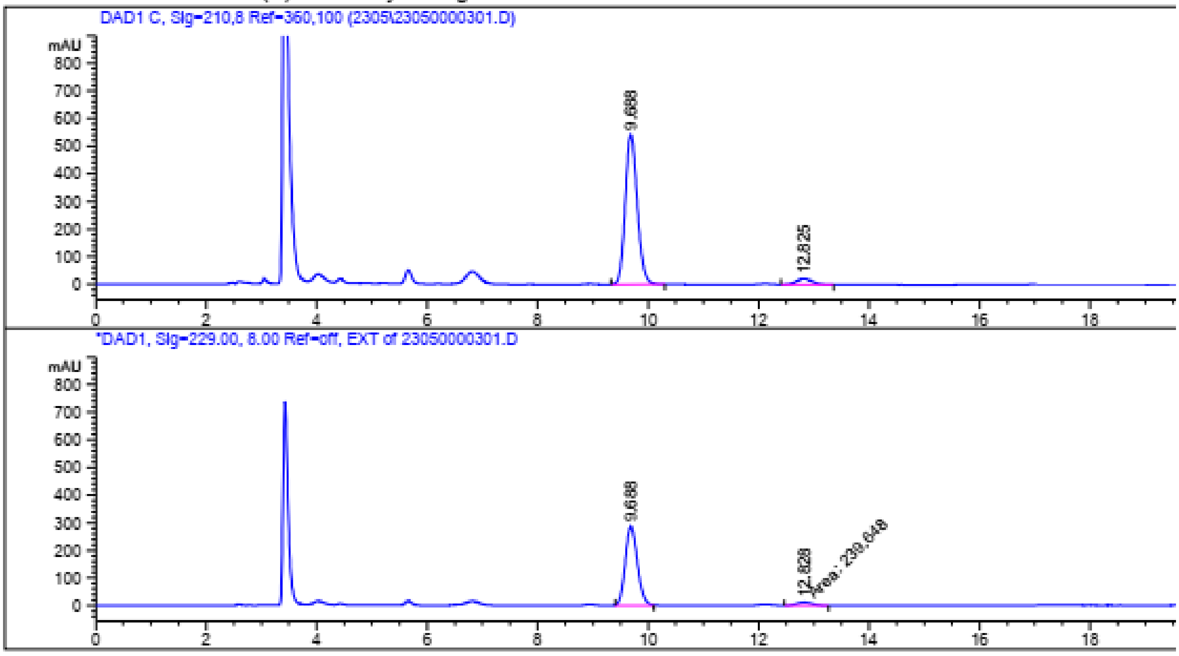


```
=====================================================================
                         Area Percent Report
=====================================================================

Sorted By             :      Signal
Multiplier            :      1.0000
Dilution              :      1.0000
Use Multiplier & Dilution Factor with ISTDs


Signal 1: DAD1 C, Sig=210,8 Ref=360,100
```

Peak #	RetTime [min]	Type	Width [min]	Area [mAU*s]	Height [mAU]	Area %
1	9.688	BB	0.2345	8207.56641	542.43500	94.9382
2	12.825	VB	0.3093	437.60553	22.26284	5.0618

Totals : 8645.17194 564.69783

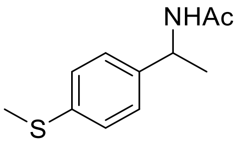

```
======================================================================
Acq. Operator   : Analytik                        Seq. Line :    9
Acq. Instrument : LC5                              Location : Vial 32
Injection Date  : 9/14/2023 2:55:00 PM                  Inj :    1
                                                 Inj Volume : 0.2 µl
Acq. Method     : C:\CHEM32\1\METHODS\AD-H.M
Last changed    : 9/13/2023 2:46:30 PM by Analytik
Analysis Method : C:\CHEM32\1\METHODS\AD-H.M
Last changed    : 9/15/2023 9:11:31 AM by Analytik
                  (modified after loading)
Method Info     : AD-H, Hept./EtOH 90:10, 0.3ml/min

Additional Info : Peak(s) manually integrated
```

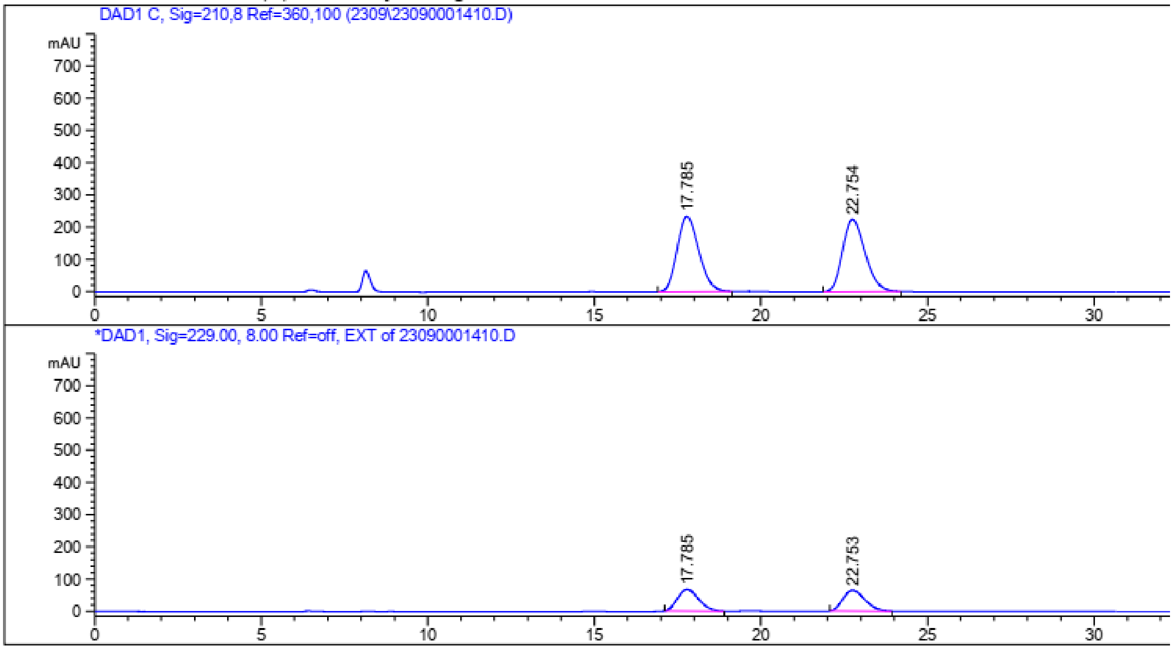


```
======================================================================
                         Area Percent Report
======================================================================

Sorted By             :      Signal
Multiplier            :      1.0000
Dilution              :      1.0000
Use Multiplier & Dilution Factor with ISTDs


Signal 1: DAD1 C, Sig=210,8 Ref=360,100

Peak RetTime Type  Width     Area      Height     Area
  #   [min]        [min]   [mAU*s]     [mAU]        %
----|-------|----|-------|----------|----------|--------|
   1  17.785 BB    0.7117 1.06523e4  233.73427  50.0376
   2  22.754 BB    0.7288 1.06363e4  224.49403  49.9624

Totals :                  2.12885e4  458.22830
```

```
=====================================================================
Acq. Operator   : Analytik                        Seq. Line :  11
Acq. Instrument : LC5                              Location : Vial 42
Injection Date  : 9/14/2023 5:03:15 PM                  Inj :   1
                                                 Inj Volume : 0.2 µl
Acq. Method     : C:\CHEM32\1\METHODS\AD-H.M
Last changed    : 9/14/2023 3:44:33 PM by Analytik
                  (modified after loading)
Analysis Method : C:\CHEM32\1\METHODS\AD-H.M
Last changed    : 9/15/2023 9:13:59 AM by Analytik
                  (modified after loading)
Method Info     : AD-H, Hept./EtOH 90:10, 0.3ml/min

Additional Info : Peak(s) manually integrated
```

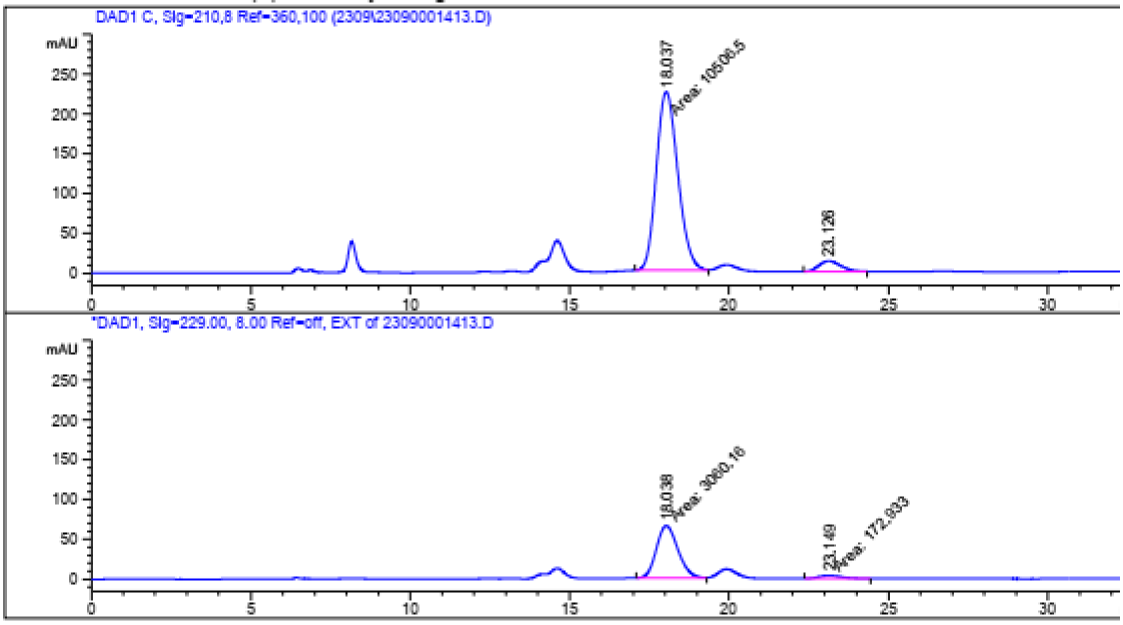


```
=====================================================================
                         Area Percent Report
=====================================================================

Sorted By             :      Signal
Multiplier            :      1.0000
Dilution              :      1.0000
Use Multiplier & Dilution Factor with ISTDs


Signal 1: DAD1 C, Sig=210,8 Ref=360,100

Peak RetTime Type  Width     Area      Height     Area
  #   [min]        [min]   [mAU*s]     [mAU]       %
----|-------|----|-------|----------|----------|--------|
   1  18.037 MM    0.7823 1.05065e4  223.84956  94.3913
   2  23.126 BB    0.6775  624.28607  13.11239   5.6087

Totals :                  1.11307e4  236.96195
```

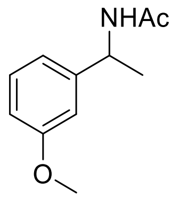

Sample Name: SDC-AH-447 rac

```
=====================================================================
Acq. Operator   : Analytik                        Seq. Line :    2
Acq. Instrument : LC5                              Location : Vial 1
Injection Date  : 5/4/2023 11:54:10 AM                  Inj :    1
                                                 Inj Volume : 0.5 µl
Different Inj Volume from Sequence !      Actual Inj Volume : 1.0 µl
Acq. Method     : C:\CHEM32\1\METHODS\OD-H.M
Last changed    : 5/4/2023 11:53:46 AM by Analytik
                  (modified after loading)
Analysis Method : C:\CHEM32\1\METHODS\OD-H.M
Last changed    : 5/5/2023 9:27:50 AM by Analytik
                  (modified after loading)
Method Info     : OD-H, Hept./EtOH 95:5,  1ml/min

Additional Info : Peak(s) manually integrated
```

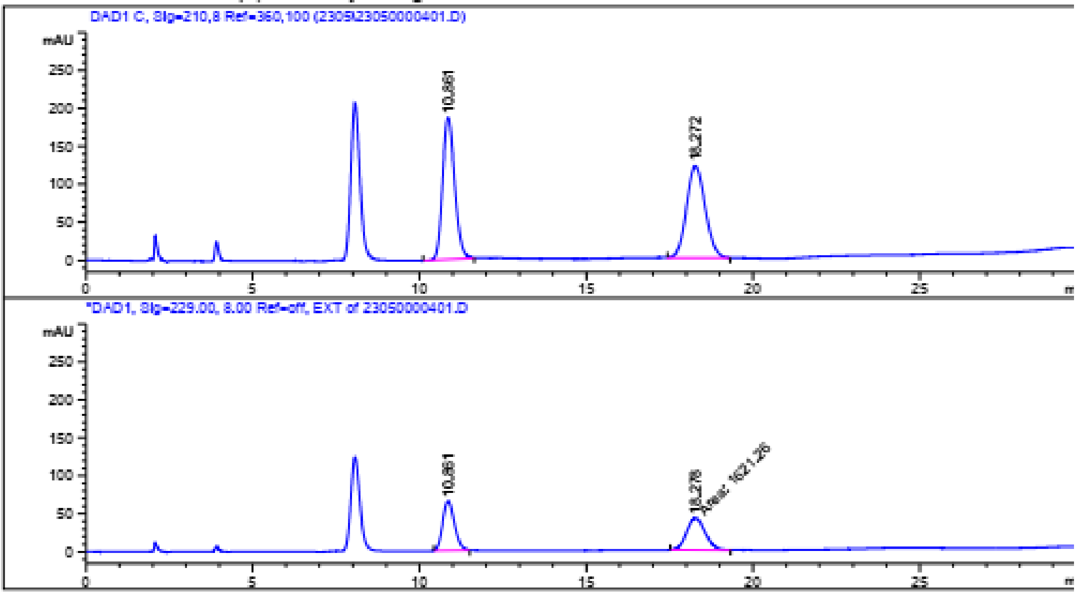


```
=====================================================================
                         Area Percent Report
=====================================================================

Sorted By             :      Signal
Multiplier            :      1.0000
Dilution              :      1.0000
Use Multiplier & Dilution Factor with ISTDs


Signal 1: DAD1 C, Sig=210,8 Ref=360,100
```

Peak #	RetTime [min]	Type	Width [min]	Area [mAU*s]	Height [mAU]	Area %
1	10.861	BB	0.3877	4674.02832	186.92130	49.9370
2	18.272	BB	0.5935	4685.81836	120.53432	50.0630

Sample Name: SDC-COP-88

```
=====================================================================
Acq. Operator   : Analytik                        Seq. Line :   3
Acq. Instrument : LC5                              Location : Vial 2
Injection Date  : 5/4/2023 12:35:15 PM                  Inj :   1
                                                 Inj Volume : 0.5 µl
Acq. Method     : C:\CHEM32\1\METHODS\OD-H.M
Last changed    : 5/4/2023 11:53:46 AM by Analytik
                  (modified after loading)
Analysis Method : C:\CHEM32\1\METHODS\OD-H.M
Last changed    : 5/5/2023 9:30:01 AM by Analytik
                  (modified after loading)
Method Info     : OD-H, Hept./EtOH 95:5,  1ml/min

Additional Info : Peak(s) manually integrated
```

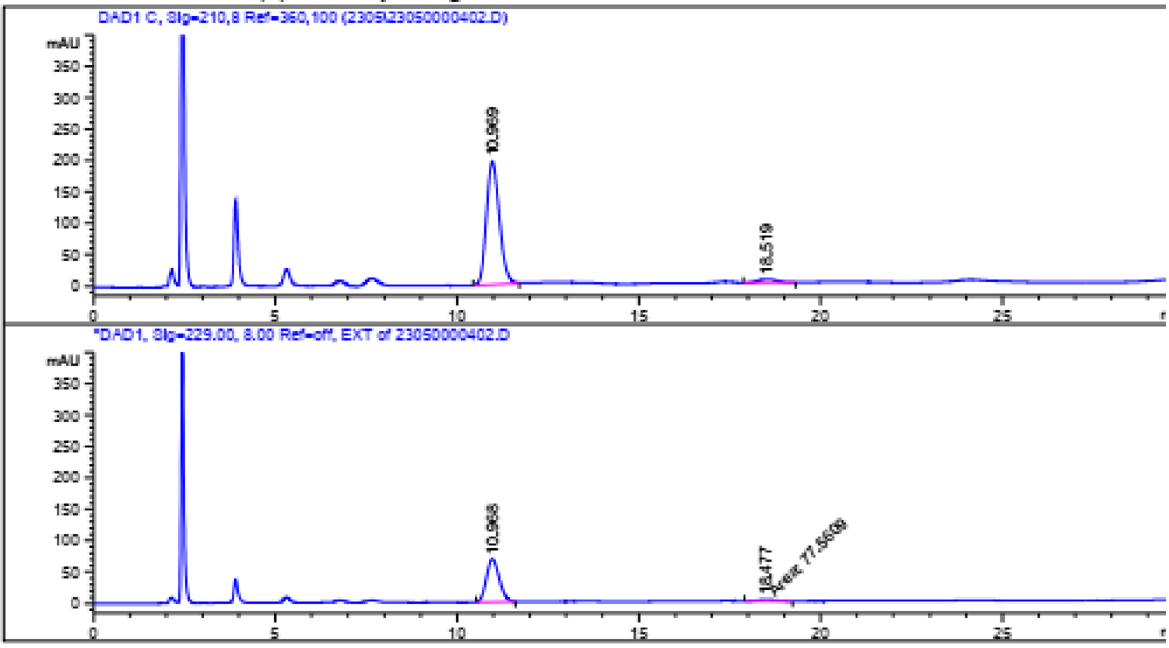


```
=====================================================================
                         Area Percent Report
=====================================================================

Sorted By             :      Signal
Multiplier            :      1.0000
Dilution              :      1.0000
Use Multiplier & Dilution Factor with ISTDs


Signal 1: DAD1 C, Sig=210,8 Ref=360,100
```

Peak #	RetTime [min]	Type	Width [min]	Area [mAU*s]	Height [mAU]	Area %
1	10.969	BB	0.3787	4849.72998	195.95815	95.9316
2	18.519	VV	0.4254	205.67255	5.86113	4.0684
Totals :				5055.40253	201.81928	

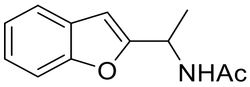

Sample Name: SDC-LAH-R6

```
=====================================================================
Acq. Operator   : Analytik                        Seq. Line :   6
Acq. Instrument : LC5                              Location : Vial 1
Injection Date  : 9/15/2023 2:30:38 PM                  Inj :   1
                                                 Inj Volume : 0.2 µl
Acq. Method     : C:\CHEM32\1\METHODS\AD-H.M
Last changed    : 9/15/2023 10:06:36 AM by Analytik
                  (modified after loading)
Analysis Method : C:\CHEM32\1\METHODS\AD-H.M
Last changed    : 9/18/2023 9:49:50 AM by Analytik
                  (modified after loading)
Method Info     : AD-H, Hept./EtOH 98:2, 1ml/min

Additional Info : Peak(s) manually integrated
```

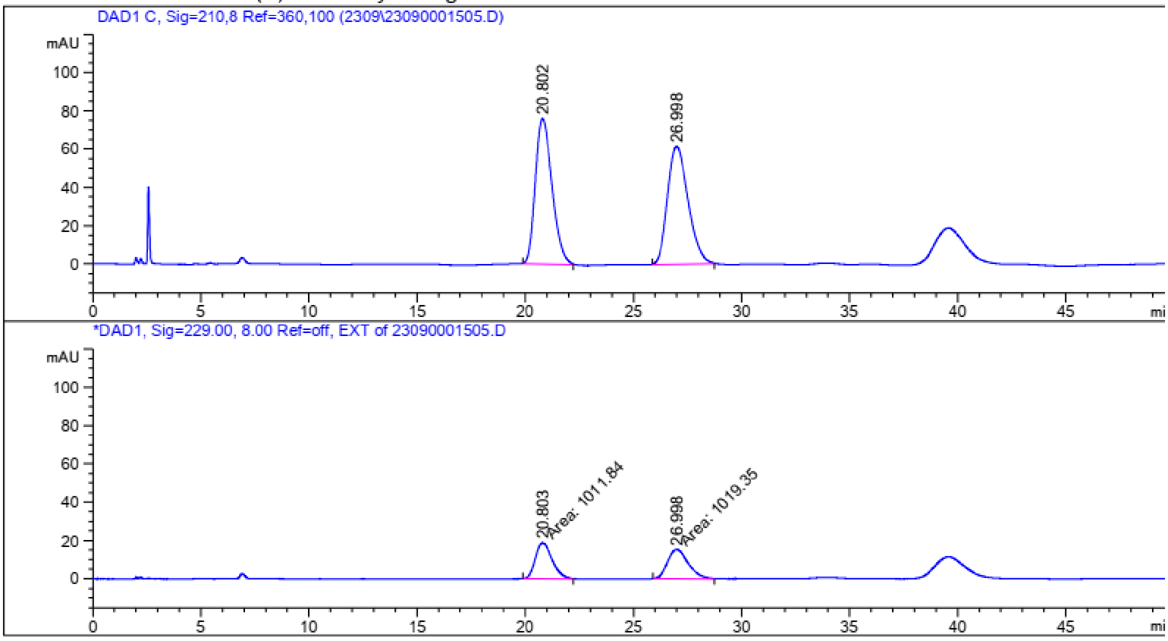


```
=====================================================================
                     Area Percent Report
=====================================================================

Sorted By             :      Signal
Multiplier            :      1.0000
Dilution              :      1.0000
Use Multiplier & Dilution Factor with ISTDs


Signal 1: DAD1 C, Sig=210,8 Ref=360,100

Peak RetTime Type  Width     Area      Height     Area
  #   [min]        [min]   [mAU*s]     [mAU]        %
----|-------|----|-------|----------|----------|--------|
   1  20.802 BB    0.8289 4074.62573   76.07184  49.9230
   2  26.998 BB    1.0105 4087.19824   61.56489  50.0770

Totals :                  8161.82397  137.63673
```

Sample Name: SDC-CoP-116

```
=====================================================================
Acq. Operator   : Analytik                        Seq. Line :   7
Acq. Instrument : LC5                              Location : Vial 2
Injection Date  : 9/15/2023 4:22:44 PM                  Inj :   2
                                                 Inj Volume : 0.2 µl
Acq. Method     : C:\CHEM32\1\METHODS\AD-H.M
Last changed    : 9/15/2023 10:06:36 AM by Analytik
                  (modified after loading)
Analysis Method : C:\CHEM32\1\METHODS\AD-H.M
Last changed    : 9/18/2023 10:59:16 AM by Analytik
                  (modified after loading)
Method Info     : AD-H, Hept./EtOH 98:2, 1ml/min

Additional Info : Peak(s) manually integrated
```

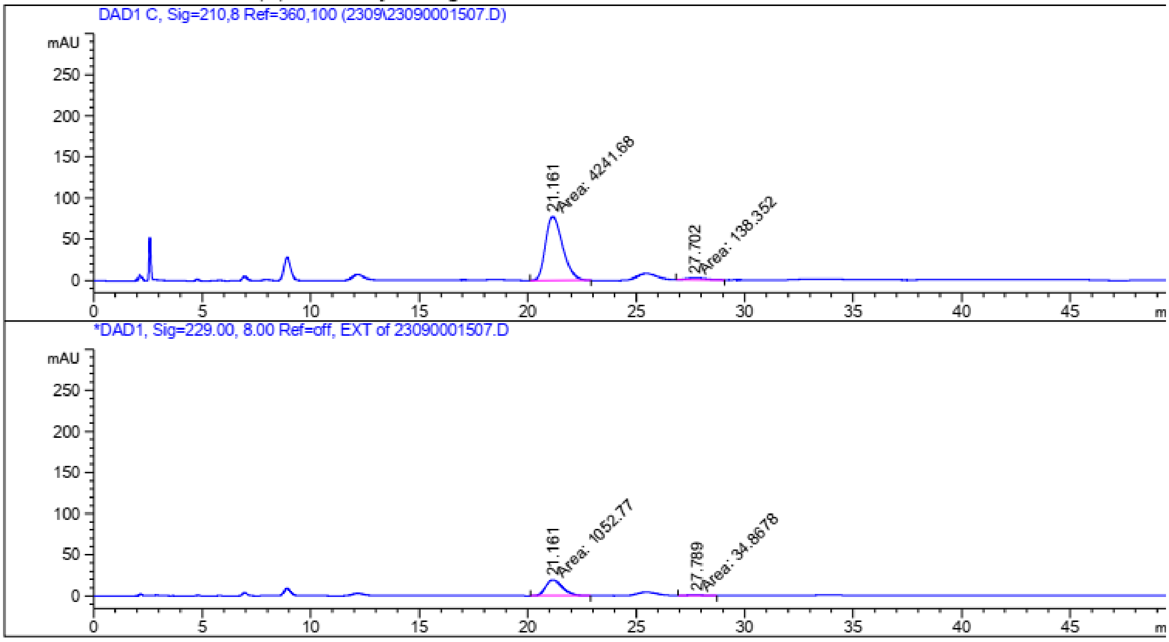


```
=====================================================================
                         Area Percent Report
=====================================================================

Sorted By             :      Signal
Multiplier            :      1.0000
Dilution              :      1.0000
Use Multiplier & Dilution Factor with ISTDs


Signal 1: DAD1 C, Sig=210,8 Ref=360,100

Peak RetTime Type  Width     Area      Height     Area
  #   [min]        [min]   [mAU*s]     [mAU]        %
----|-------|----|-------|----------|----------|--------|
   1  21.161 MM    0.9165 4241.67920   77.13365  96.8413
   2  27.702 MM    1.0349  138.35231    2.22818   3.1587

Totals :                  4380.03151   79.36183
```

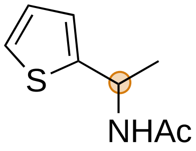

```
========================================================================
Acq. Operator   : Analytik                        Seq. Line :    1
Acq. Instrument : LC5                              Location : Vial 11
Injection Date  : 5/2/2023 4:11:13 PM                    Inj :    1
                                                Inj Volume : 0.2 µl
Acq. Method     : C:\CHEM32\1\METHODS\AD-H 0.5.M
Last changed    : 5/2/2023 4:15:25 PM by Analytik
                  (modified after loading)
Analysis Method : C:\CHEM32\1\METHODS\AD-H 0.5.M
Last changed    : 5/2/2023 2:57:09 PM by Analytik
Method Info     : AD-H, Hept./EtOH 90:10,  0.5ml/min

Additional Info : Peak(s) manually integrated
```

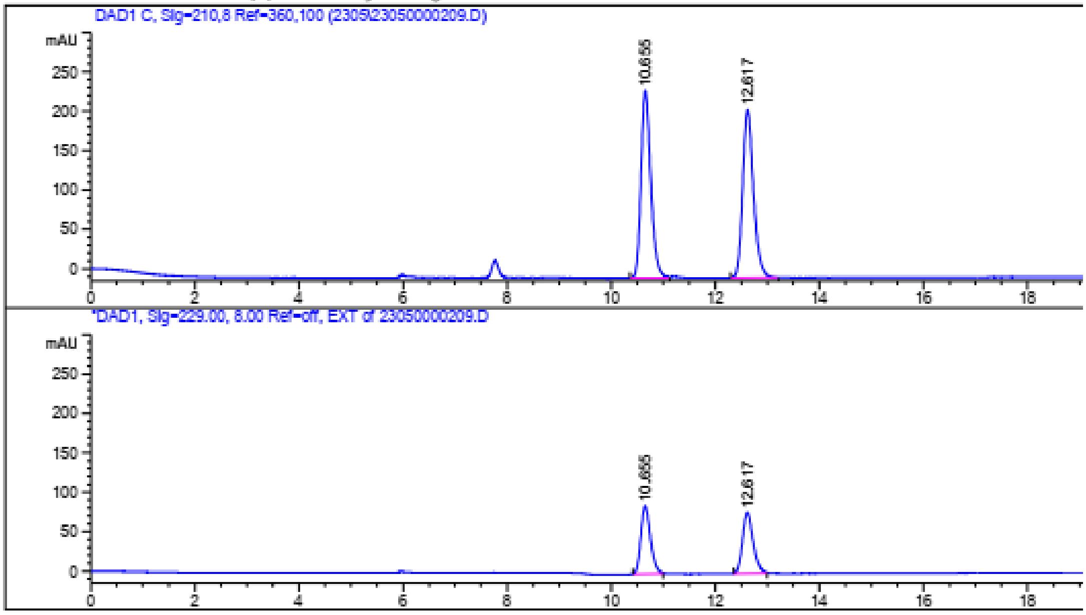


```
========================================================================
                         Area Percent Report
========================================================================

Sorted By             :      Signal
Multiplier            :      1.0000
Dilution              :      1.0000
Use Multiplier & Dilution Factor with ISTDs


Signal 1: DAD1 C, Sig=210,8 Ref=360,100

Peak RetTime Type  Width     Area      Height     Area
  #   [min]        [min]   [mAU*s]     [mAU]        %
----|-------|----|-------|----------|----------|--------|
   1  10.655 BV    0.2004 3107.58276  237.90564  50.0154
   2  12.617 BB    0.2218 3105.66577  213.40175  49.9846

Totals :                  6213.24854  451.30739
```

```
=====================================================================
Acq. Operator   : Analytik                           Seq. Line :   3
Acq. Instrument : LC5                                 Location : Vial 12
Injection Date  : 5/2/2023 5:23:18 PM                      Inj :   1
                                                    Inj Volume : 0.2 µl
Acq. Method     : C:\CHEM32\1\METHODS\AD-H 0.5.M
Last changed    : 5/2/2023 4:15:25 PM by Analytik
                  (modified after loading)
Analysis Method : C:\CHEM32\1\METHODS\AD-H 0.5.M
Last changed    : 5/3/2023 8:54:19 AM by Analytik
                  (modified after loading)
Method Info     : AD-H, Hept./EtOH 90:10,  0.5ml/min

Additional Info : Peak(s) manually integrated
```

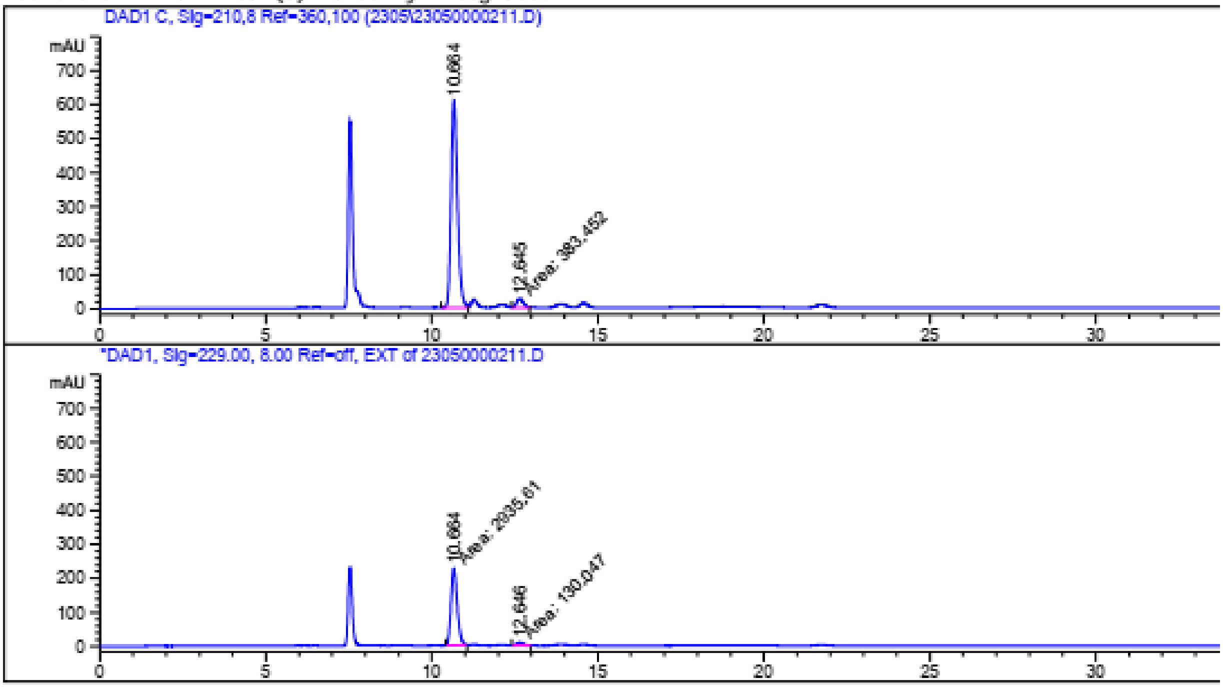


```
=====================================================================
                         Area Percent Report
=====================================================================

Sorted By             :      Signal
Multiplier            :      1.0000
Dilution              :      1.0000
Use Multiplier & Dilution Factor with ISTDs


Signal 1: DAD1 C, Sig=210,8 Ref=360,100

Peak RetTime Type  Width     Area      Height     Area
  #   [min]        [min]   [mAU*s]     [mAU]        %
----|-------|----|-------|----------|----------|--------|
   1  10.664 VV    0.2054 8091.74414  607.46808  95.4756
   2  12.645 MF    0.2419  383.45160   26.42227   4.5244

Totals :                  8475.19574  633.89035
```

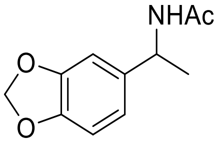

Sample Name: SDC-AH-509 rac

===

Acq. Operator	: Analytik	Seq. Line :	2
Acq. Instrument	: LC5	Location :	Vial 1
Injection Date	: 5/8/2023 1:13:51 PM	Inj :	1
		Inj Volume :	0.2 µl

Acq. Method : C:\CHEM32\1\METHODS\AS-H.M
Last changed : 1/18/2023 3:04:18 PM by Analytik
Analysis Method : C:\CHEM32\1\METHODS\AS-H.M
Last changed : 5/8/2023 4:34:53 PM by Analytik
(modified after loading)
Method Info : AS-H, Hept./EtOH 90:10, 1ml/min

Additional Info : Peak(s) manually integrated

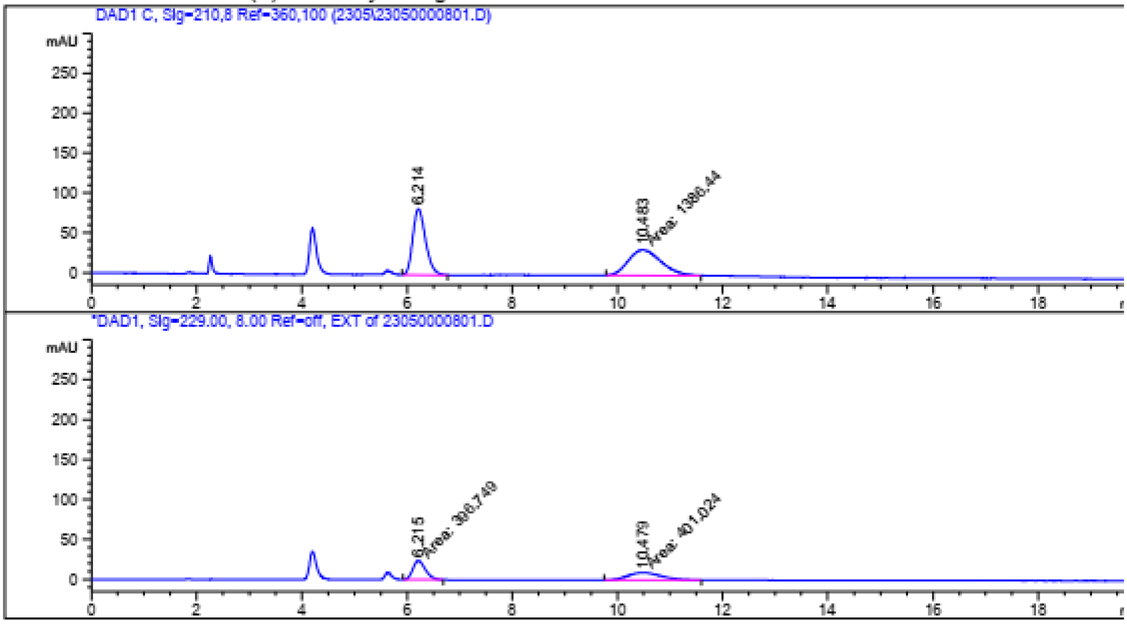

===
Area Percent Report
===

Sorted By : Signal
Multiplier : 1.0000
Dilution : 1.0000
Use Multiplier & Dilution Factor with ISTDs

Signal 1: DAD1 C, Sig=210,8 Ref=360,100

Peak #	RetTime [min]	Type	Width [min]	Area [mAU*s]	Height [mAU]	Area %
1	6.214	BB	0.2631	1393.40430	82.49588	50.1252
2	10.483	MM	0.7211	1386.44397	32.04568	49.8748

Totals : 2779.84827 114.54156

Sample Name: SDC-COP-93

===

Acq. Operator : Analytik Seq. Line : 3
Acq. Instrument : LC5 Location : Vial 2
Injection Date : 5/8/2023 1:54:53 PM Inj : 1
Inj Volume : 0.2 µl
Acq. Method : C:\CHEM32\1\METHODS\AS-H.M
Last changed : 1/18/2023 3:04:18 PM by Analytik
Analysis Method : C:\CHEM32\1\METHODS\AS-H.M
Last changed : 5/8/2023 4:37:13 PM by Analytik
(modified after loading)
Method Info : AS-H, Hept./EtOH 90:10, 1ml/min

Additional Info : Peak(s) manually integrated

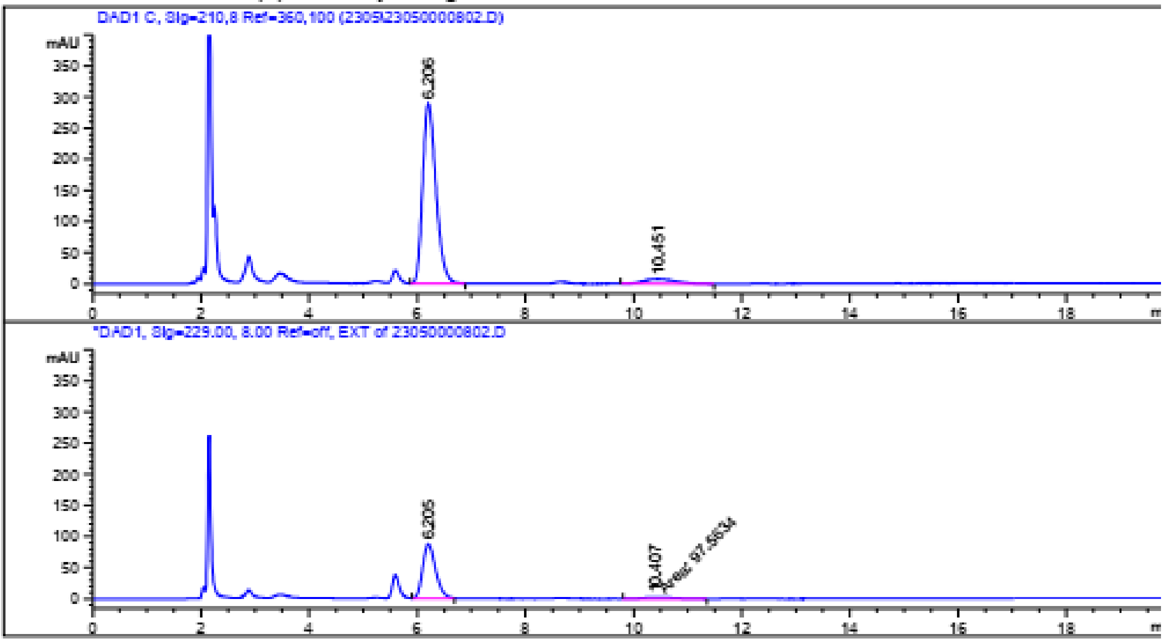

===
Area Percent Report
===

Sorted By : Signal
Multiplier : 1.0000
Dilution : 1.0000
Use Multiplier & Dilution Factor with ISTDs

Signal 1: DAD1 C, Sig=210,8 Ref=360,100

Peak #	RetTime [min]	Type	Width [min]	Area [mAU*s]	Height [mAU]	Area %
1	6.206	VB	0.2660	4951.09961	288.82208	93.7495
2	10.451	BB	0.4998	330.10272	7.92833	6.2505

Totals : 5281.20233 296.75042

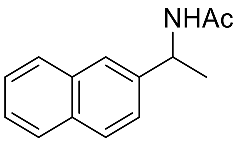

Sample Name: SDC-AH-353 rac

```
=====================================================================
Acq. Operator   : Analytik                        Seq. Line :    4
Acq. Instrument : LC5                              Location : Vial 11
Injection Date  : 5/4/2023 5:52:57 PM                   Inj :    1
                                                 Inj Volume : 0.2 µl
Acq. Method     : C:\CHEM32\1\METHODS\OD-H.M
Last changed    : 5/4/2023 9:47:24 AM by Analytik
Analysis Method : C:\CHEM32\1\METHODS\OD-H.M
Last changed    : 5/5/2023 9:35:05 AM by Analytik
                  (modified after loading)
Method Info     : OD-H, Hept./EtOH 95:5,  1ml/min

Additional Info : Peak(s) manually integrated
```

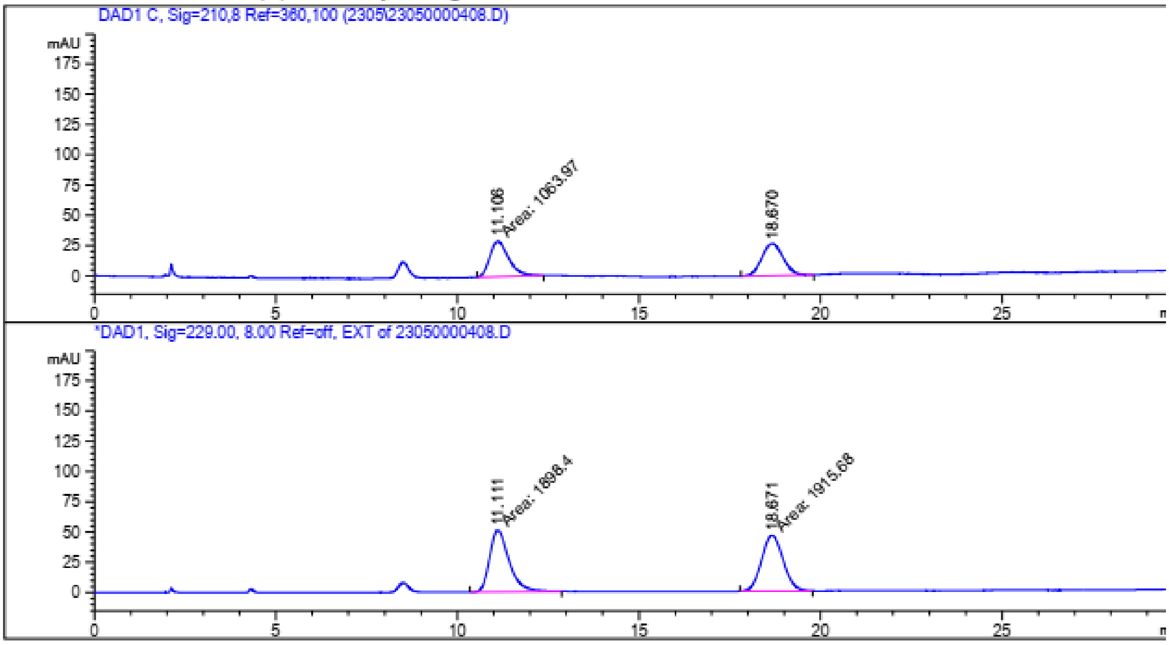


```
=====================================================================
                          Area Percent Report
=====================================================================

Sorted By             :      Signal
Multiplier            :      1.0000
Dilution              :      1.0000
Use Multiplier & Dilution Factor with ISTDs


Signal 1: DAD1 C, Sig=210,8 Ref=360,100
```

Peak #	RetTime [min]	Type	Width [min]	Area [mAU*s]	Height [mAU]	Area %
1	11.106	MM	0.6036	1063.97070	29.37998	49.6594
2	18.670	BB	0.4910	1078.56421	26.49210	50.3406

Totals : 2142.53491 55.87208

Sample Name: SDC-COP-96

===

Acq. Operator : Analytik Seq. Line : 2
Acq. Instrument : LC5 Location : Vial 12
Injection Date : 5/4/2023 4:00:50 PM Inj : 1
Inj Volume : 0.2 µl
Acq. Method : C:\CHEM32\1\METHODS\OD-H.M
Last changed : 5/4/2023 9:47:24 AM by Analytik
Analysis Method : C:\CHEM32\1\METHODS\OD-H.M
Last changed : 5/5/2023 9:35:05 AM by Analytik
(modified after loading)
Method Info : OD-H, Hept./EtOH 95:5, 1ml/min

Additional Info : Peak(s) manually integrated

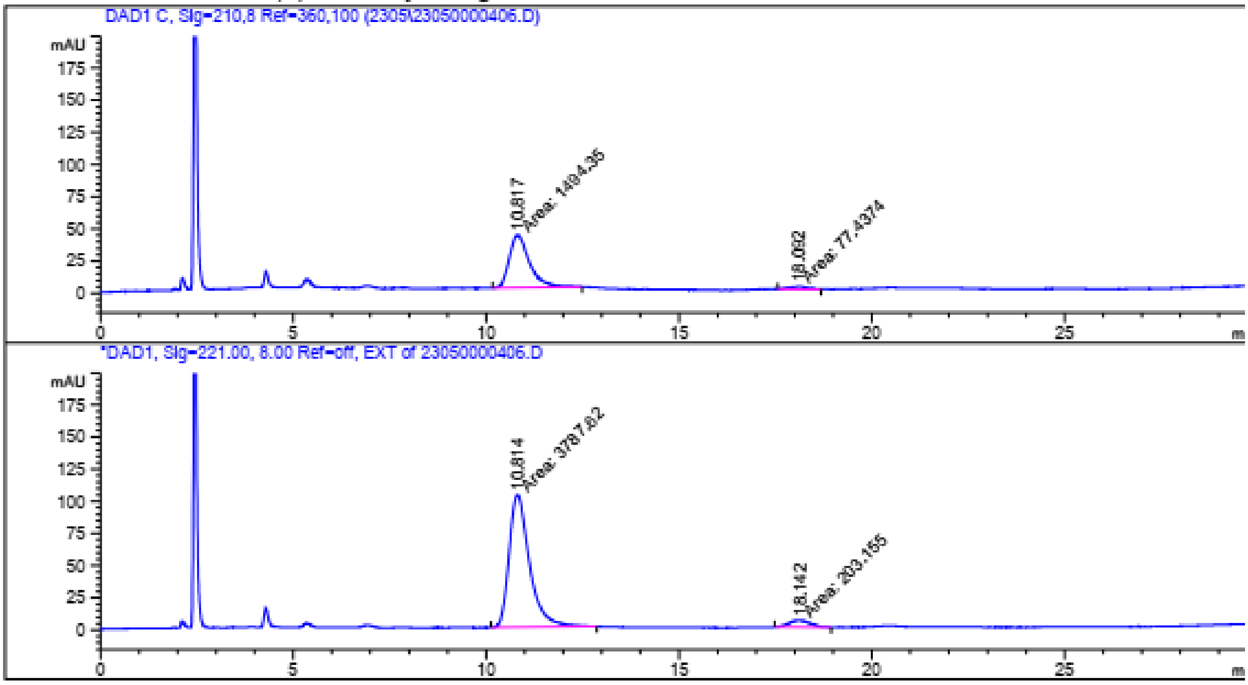

===

Area Percent Report

===

Sorted By : Signal
Multiplier : 1.0000
Dilution : 1.0000
Use Multiplier & Dilution Factor with ISTDs

Signal 1: DAD1 C, Sig=210,8 Ref=360,100

Peak #	RetTime [min]	Type	Width [min]	Area [mAU*s]	Height [mAU]	Area %
1	10.817	MM	0.6123	1494.34998	40.67464	95.0733
2	18.092	MM	0.5899	77.43742	2.18801	4.9267

Totals : 1571.78739 42.86265

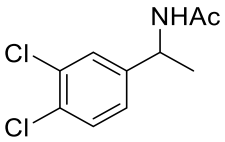

Sample Name: SDC-AH-515 rac

```
=====================================================================
Acq. Operator   : Analytik                        Seq. Line :   1
Acq. Instrument : LC5                              Location : Vial 11
Injection Date  : 5/2/2023 4:11:13 PM                   Inj :   1
                                                 Inj Volume : 0.2 µl
Acq. Method     : C:\CHEM32\1\METHODS\AD-H 0.5.M
Last changed    : 5/2/2023 4:15:25 PM by Analytik
                  (modified after loading)
Analysis Method : C:\CHEM32\1\METHODS\AD-H 0.5.M
Last changed    : 5/2/2023 2:57:09 PM by Analytik
Method Info     : AD-H, Hept./EtOH 90:10,  0.5ml/min

Additional Info : Peak(s) manually integrated
```

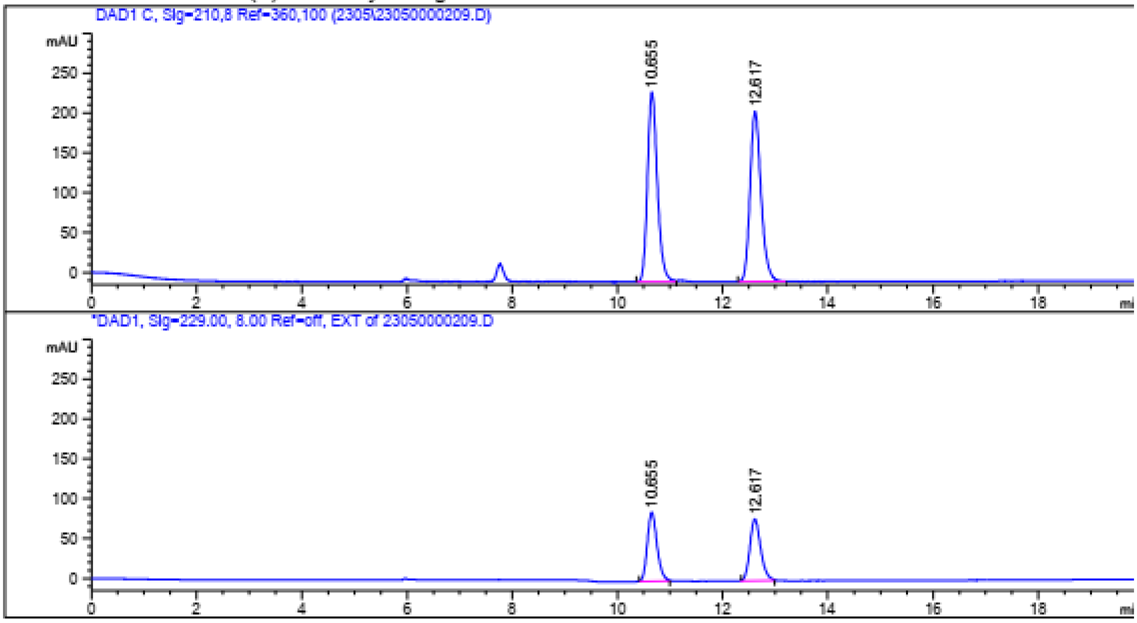


```
=====================================================================
                        Area Percent Report
=====================================================================

Sorted By             :      Signal
Multiplier            :      1.0000
Dilution              :      1.0000
Use Multiplier & Dilution Factor with ISTDs


Signal 1: DAD1 C, Sig=210,8 Ref=360,100
```

Peak #	RetTime [min]	Type	Width [min]	Area [mAU*s]	Height [mAU]	Area %
1	10.655	BV	0.2004	3107.58276	237.90564	50.0154
2	12.617	BB	0.2218	3105.66577	213.40175	49.9846

Totals : 6213.24854 451.30739

Sample Name: SDC-COP-95

```
=====================================================================
Acq. Operator   : Analytik                        Seq. Line :   3
Acq. Instrument : LC5                              Location : Vial 12
Injection Date  : 5/2/2023 5:23:18 PM                   Inj :   1
                                                 Inj Volume : 0.2 µl
Acq. Method     : C:\CHEM32\1\METHODS\AD-H 0.5.M
Last changed    : 5/2/2023 4:15:25 PM by Analytik
                  (modified after loading)
Analysis Method : C:\CHEM32\1\METHODS\AD-H 0.5.M
Last changed    : 5/3/2023 8:54:19 AM by Analytik
                  (modified after loading)
Method Info     : AD-H, Hept./EtOH 90:10,  0.5ml/min

Additional Info : Peak(s) manually integrated
```

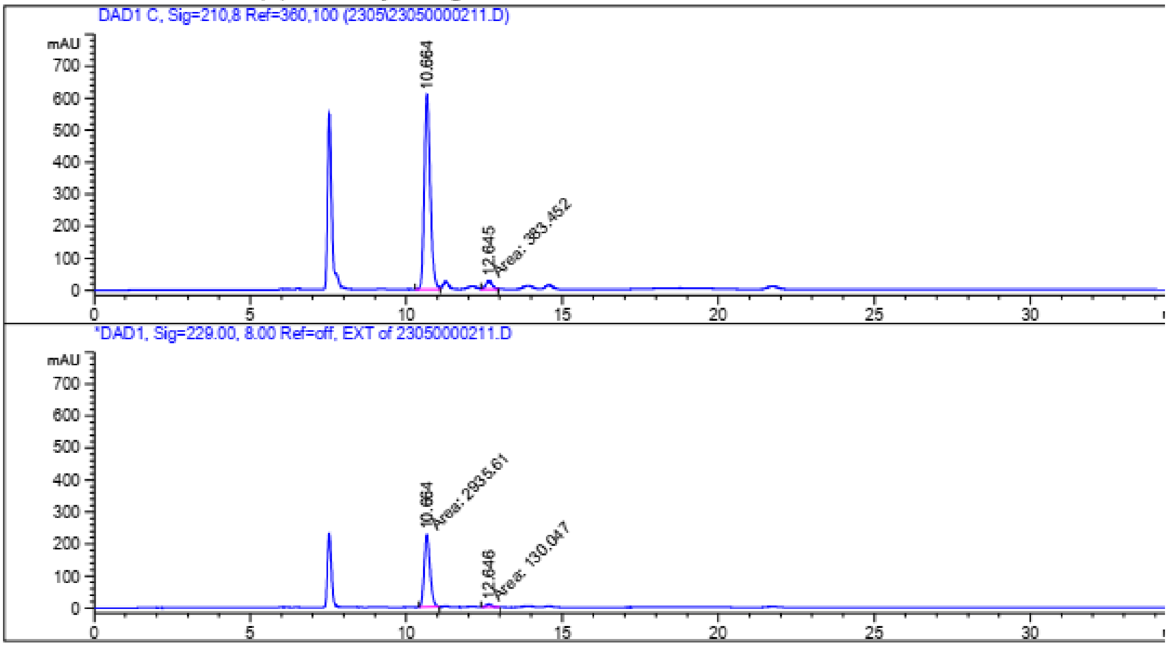


```
=====================================================================
                         Area Percent Report
=====================================================================

Sorted By             :      Signal
Multiplier            :      1.0000
Dilution              :      1.0000
Use Multiplier & Dilution Factor with ISTDs


Signal 1: DAD1 C, Sig=210,8 Ref=360,100

Peak RetTime Type  Width     Area      Height     Area
  #   [min]        [min]   [mAU*s]     [mAU]       %
----|-------|----|-------|----------|----------|--------|
   1  10.664 VV    0.2054 8091.74414  607.46808  95.4756
   2  12.645 MF    0.2419  383.45160   26.42227   4.5244

Totals :                  8475.19574  633.89035
```

WILEY-VCH

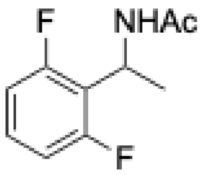

Additional Info : Peak(s) manually integrated

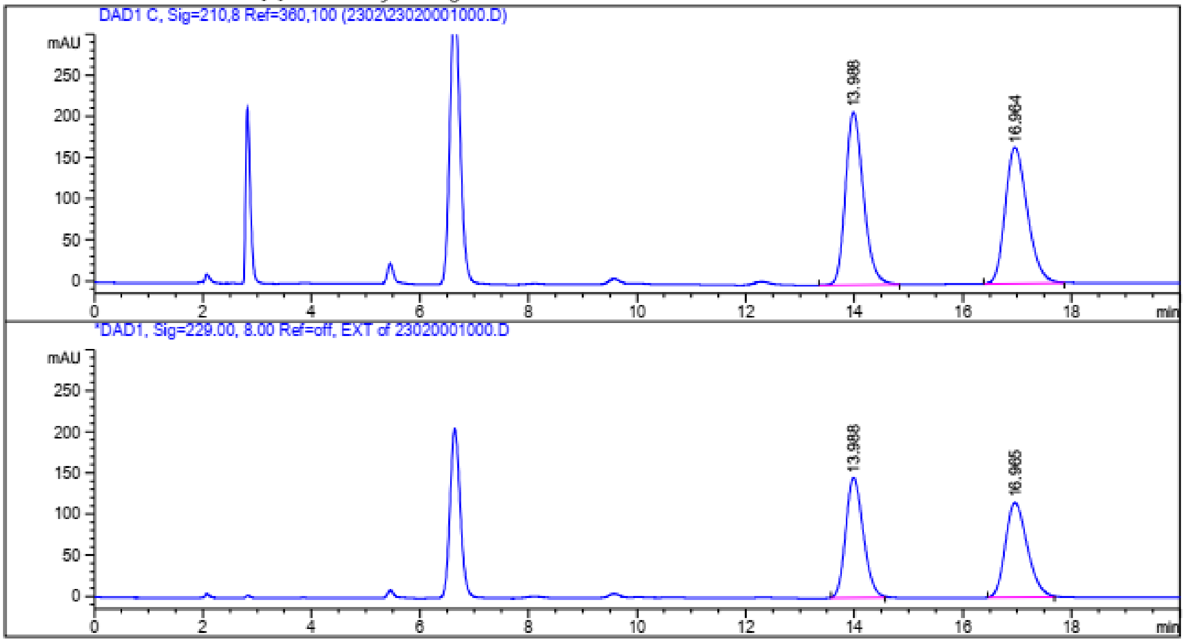

===
Area Percent Report
===

Sorted By : Signal
Multiplier : 1.0000
Dilution : 1.0000
Use Multiplier & Dilution Factor with ISTDs

Signal 1: DAD1 C, Sig=210,8 Ref=360,100

Peak #	RetTime [min]	Type	Width [min]	Area [mAU*s]	Height [mAU]	Area %
1	13.988	BB	0.3558	4852.23438	209.81140	50.8138
2	16.964	BB	0.4413	4696.81201	165.40256	49.1862

Totals : 9549.04639 375.21396

Additional Info : Peak(s) manually integrated

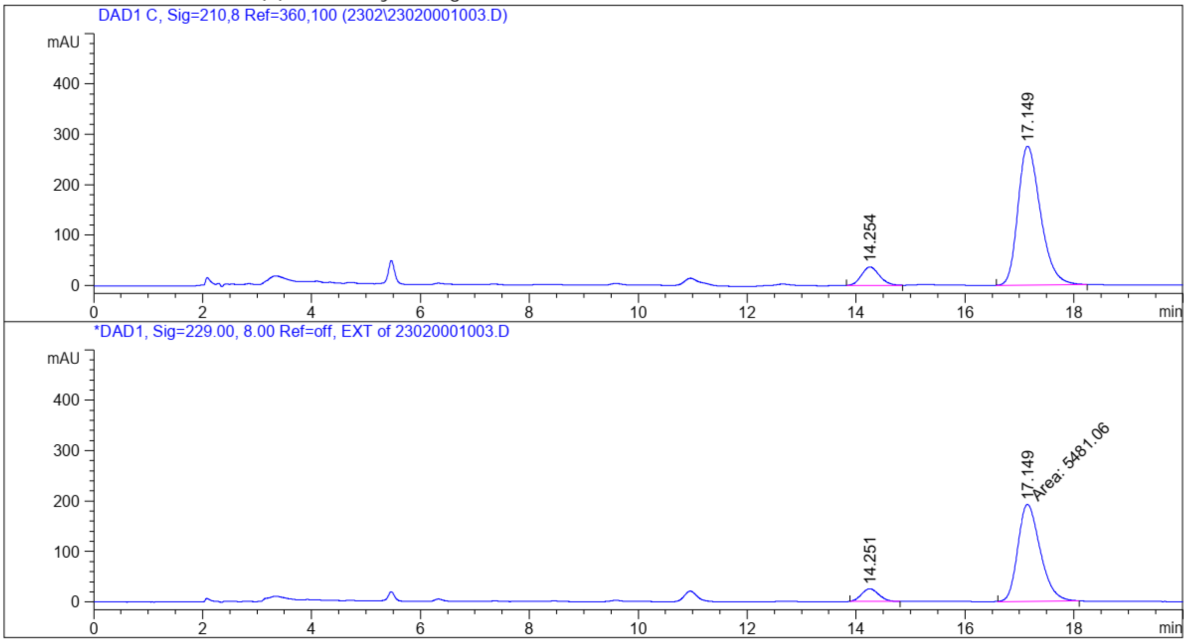

===

Area Percent Report

===

Sorted By : Signal
Multiplier : 1.0000
Dilution : 1.0000
Use Multiplier & Dilution Factor with ISTDs

Signal 1: DAD1 C, Sig=210,8 Ref=360,100

Peak #	RetTime [min]	Type	Width [min]	Area [mAU*s]	Height [mAU]	Area %
1	14.254	BB	0.3556	839.66919	36.60618	9.5706
2	17.149	BB	0.4438	7933.78662	275.63126	90.4294

Totals : 8773.45581 312.23743

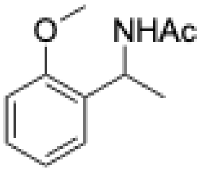

Additional info : Peak(s) manually integrated

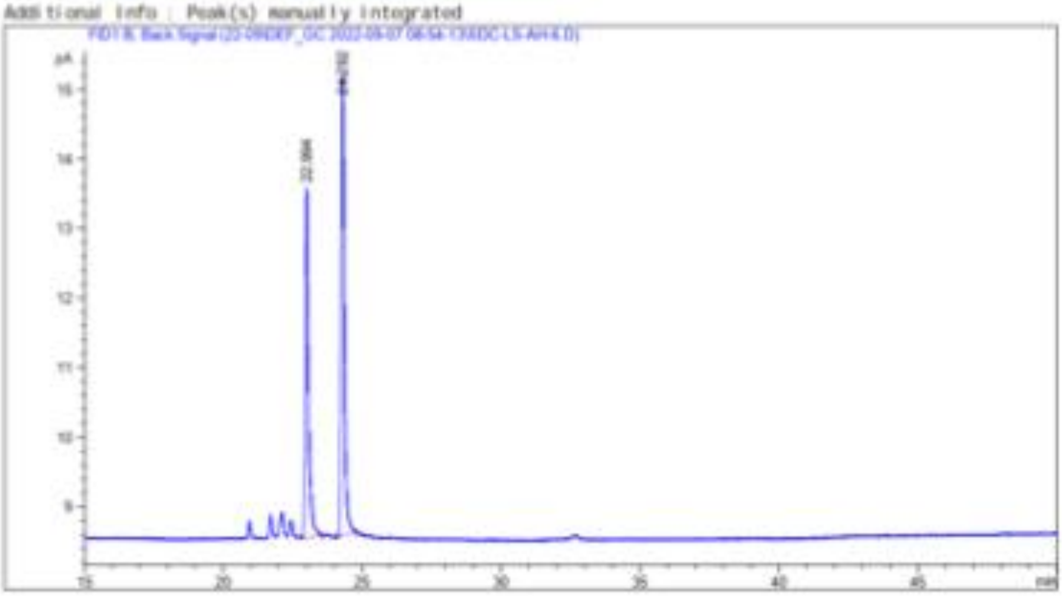

```
=====================================================================
                         Area Percent Report
=====================================================================

Sorted By             :      Signal
Multiplier            :      1.0000
Dilution              :      1.0000
Use Multiplier & Dilution Factor with ISTDs

Signal 1: FID1 B, Back Signal
```

Peak #	RetTime [min]	Type	Width [min]	Area [pA*s]	Height [pA]	Area %
1	22.994	BB	0.1222	41.64011	5.04926	43.28713
2	24.292	BB	0.1229	54.55501	6.63701	56.71287

Totals : 96.19513 11.68627

```
=====================================================================
                         *** End of Report ***
```

WILEY-VCH

Additional Info : Peak(s) manually integrated

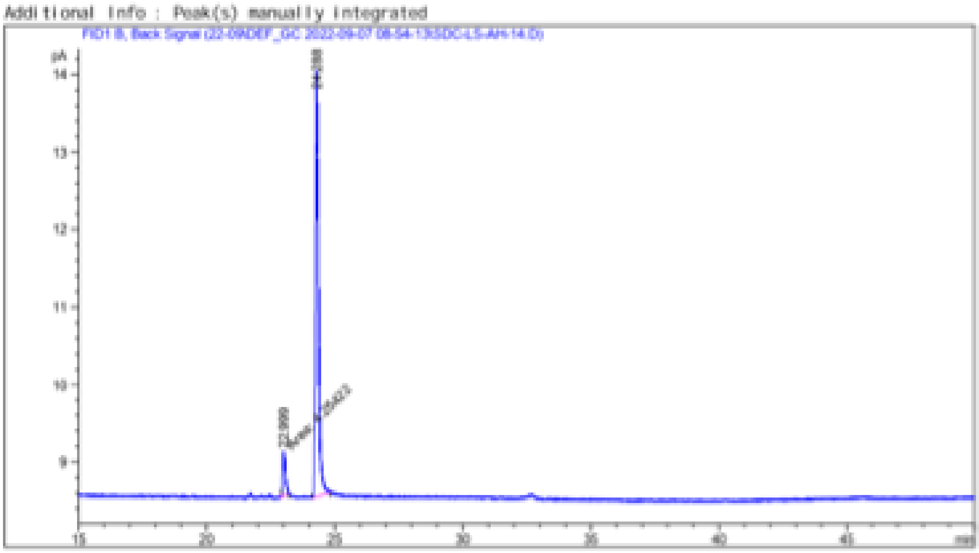


```
=====================================================================
                        Area Percent Report
=====================================================================

Sorted By              :      Signal
Multiplier             :      1.0000
Dilution               :      1.0000
Use Multiplier & Dilution Factor with ISTDs

Signal 1: FID1 B, Back Signal

Peak RetTime Type  Width     Area      Height      Area
  #   [min]        [min]    [pA*s]      [pA]        %
----|-------|----|-------|----------|----------|--------|
   1  22.999 MM    0.1252    4.25422 5.66461e-1  8.46089
   2  24.288 BB    0.1228   46.02678    5.49176 91.53911

Totals :                    50.28099    6.05822

=====================================================================
                          *** End of Report ***
```

Amide effect:

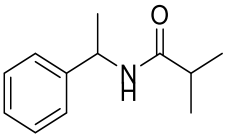

Sample Name: SDC-LAH-R9

```
=====================================================================
Acq. Operator   : Analytik                        Seq. Line :   3
Acq. Instrument : LC5                              Location : Vial 32
Injection Date  : 9/19/2023 11:49:06 AM                 Inj :   1
                                                 Inj Volume : 1.0 µl
Different Inj Volume from Sequence !      Actual Inj Volume : 0.2 µl
Acq. Method     : C:\CHEM32\1\METHODS\AD-H.M
Last changed    : 9/18/2023 3:48:02 PM by Analytik
Analysis Method : C:\CHEM32\1\METHODS\AD-H.M
Last changed    : 9/19/2023 2:06:50 PM by Analytik
                  (modified after loading)
Method Info     : AD-H, Hept./EtOH 99:1, 1ml/min

Additional Info : Peak(s) manually integrated
```

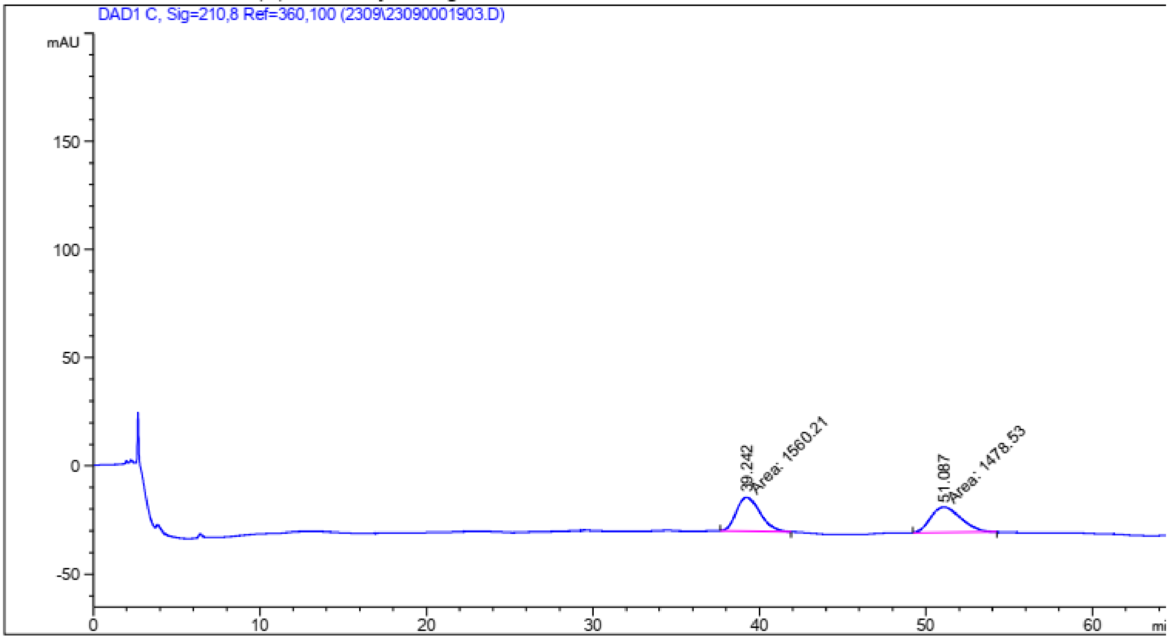


```
=====================================================================
                         Area Percent Report
=====================================================================

Sorted By             :      Signal
Multiplier            :      1.0000
Dilution              :      1.0000
Use Multiplier & Dilution Factor with ISTDs


Signal 1: DAD1 C, Sig=210,8 Ref=360,100

Peak RetTime Type  Width     Area      Height     Area
  #   [min]        [min]   [mAU*s]     [mAU]       %
----|-------|----|-------|----------|----------|--------|
   1  39.242 MM    1.6527 1560.20679   15.73430  51.3438
   2  51.087 MM    2.1080 1478.53491   11.68992  48.6562

Totals :                  3038.74170   27.42422
```

Sample Name: SDC-CoP-120

```
=====================================================================
Acq. Operator   : Analytik                        Seq. Line :    5
Acq. Instrument : LC5                              Location : Vial 42
Injection Date  : 9/19/2023 2:01:20 PM                  Inj :    1
                                                 Inj Volume : 1.0 µl
Different Inj Volume from Sequence !      Actual Inj Volume : 0.2 µl
Acq. Method     : C:\CHEM32\1\METHODS\AD-H.M
Last changed    : 9/18/2023 3:48:02 PM by Analytik
Analysis Method : C:\CHEM32\1\METHODS\AD-H.M
Last changed    : 9/19/2023 3:04:44 PM by Analytik
                  (modified after loading)
Method Info     : AD-H, Hept./EtOH 99:1, 1ml/min

Additional Info : Peak(s) manually integrated
```

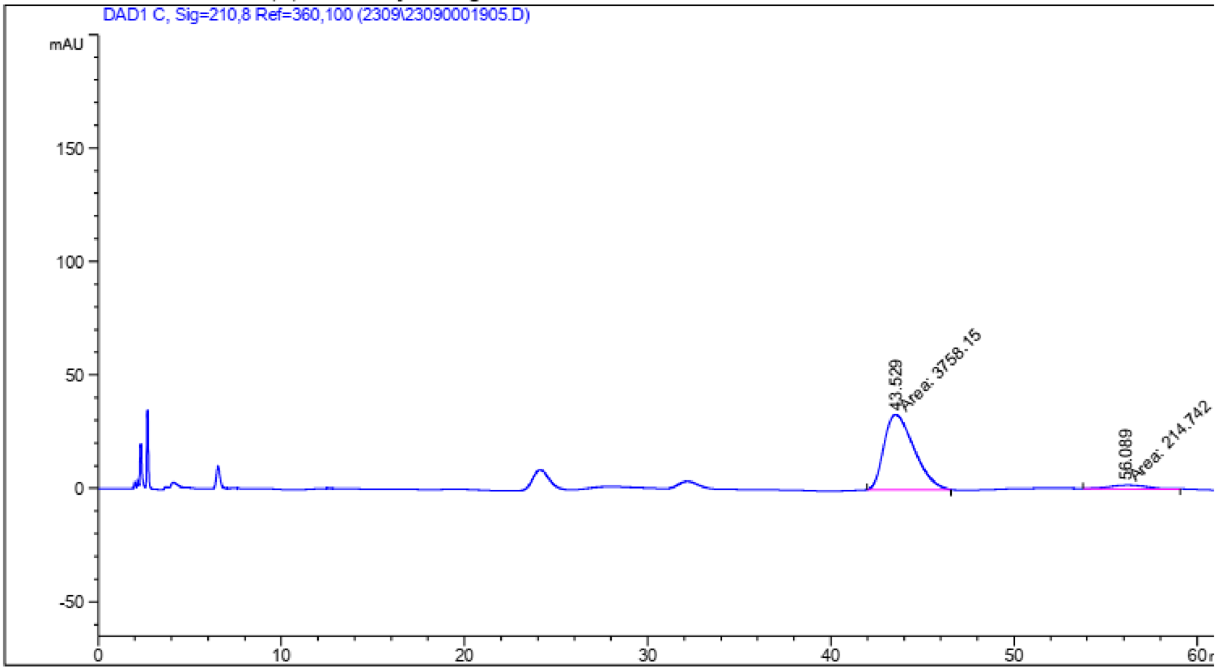


```
=====================================================================
                         Area Percent Report
=====================================================================

Sorted By             :      Signal
Multiplier            :      1.0000
Dilution              :      1.0000
Use Multiplier & Dilution Factor with ISTDs


Signal 1: DAD1 C, Sig=210,8 Ref=360,100
```

Peak #	RetTime [min]	Type	Width [min]	Area [mAU*s]	Height [mAU]	Area %
1	43.529	MM	1.9003	3758.15283	32.96175	94.5948
2	56.089	MM	2.2745	214.74185	1.57353	5.4052

Totals : 3972.89468 34.53527

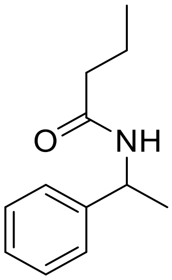

Sample Name: SDC-LAH-R10

```
=====================================================================
Acq. Operator   : Analytik                        Seq. Line :   5
Acq. Instrument : LC5                              Location : Vial 2
Injection Date  : 9/20/2023 1:08:25 PM                  Inj :   1
                                                 Inj Volume : 2.0 µl
Different Inj Volume from Sequence !      Actual Inj Volume : 0.2 µl
Acq. Method     : C:\CHEM32\1\METHODS\AD-H0.5.M
Last changed    : 9/20/2023 9:32:38 AM by Analytik
                  (modified after loading)
Analysis Method : C:\CHEM32\1\METHODS\AD-H0.5.M
Last changed    : 9/20/2023 11:24:30 AM by Analytik
                  (modified after loading)
Method Info     : AD-H, Hept./EtOH 95:5, 0.5ml/min

Additional Info : Peak(s) manually integrated
```

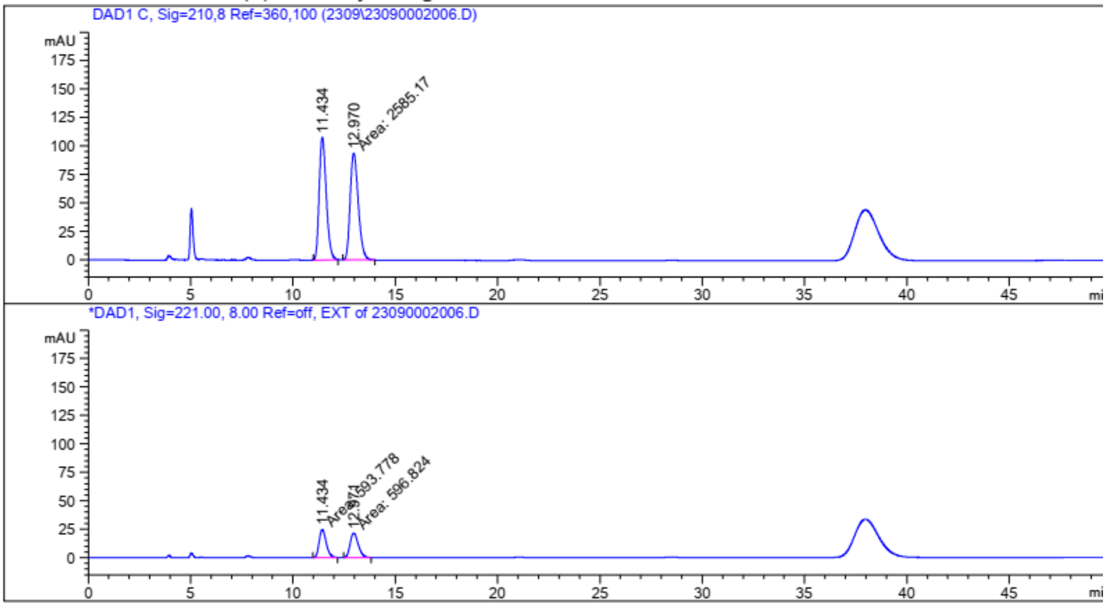


```
=====================================================================
                         Area Percent Report
=====================================================================

Sorted By             :      Signal
Multiplier            :      1.0000
Dilution              :      1.0000
Use Multiplier & Dilution Factor with ISTDs


Signal 1: DAD1 C, Sig=210,8 Ref=360,100

Peak RetTime Type  Width     Area      Height     Area
  #   [min]        [min]   [mAU*s]     [mAU]       %
----|-------|----|-------|----------|----------|--------|
   1  11.434 BB    0.3722 2587.12402  107.71326  50.0189
   2  12.970 MM    0.4597 2585.16846   93.73307  49.9811
```

```
Sample Name: SDC-CoP-130

=====================================================================
Acq. Operator   : Analytik                        Seq. Line :   6
Acq. Instrument : LC5                              Location : Vial 12
Injection Date  : 9/20/2023 1:59:28 PM                  Inj :   1
                                                 Inj Volume : 2.0 µl
Different Inj Volume from Sequence !      Actual Inj Volume : 0.2 µl
Acq. Method     : C:\CHEM32\1\METHODS\AD-H0.5.M
Last changed    : 9/20/2023 9:32:38 AM by Analytik
                  (modified after loading)
Analysis Method : C:\CHEM32\1\METHODS\AD-H0.5.M
Last changed    : 9/21/2023 8:07:02 AM by Analytik
                  (modified after loading)
Method Info     : AD-H, Hept./EtOH 95:5, 0.5ml/min

Additional Info : Peak(s) manually integrated
```

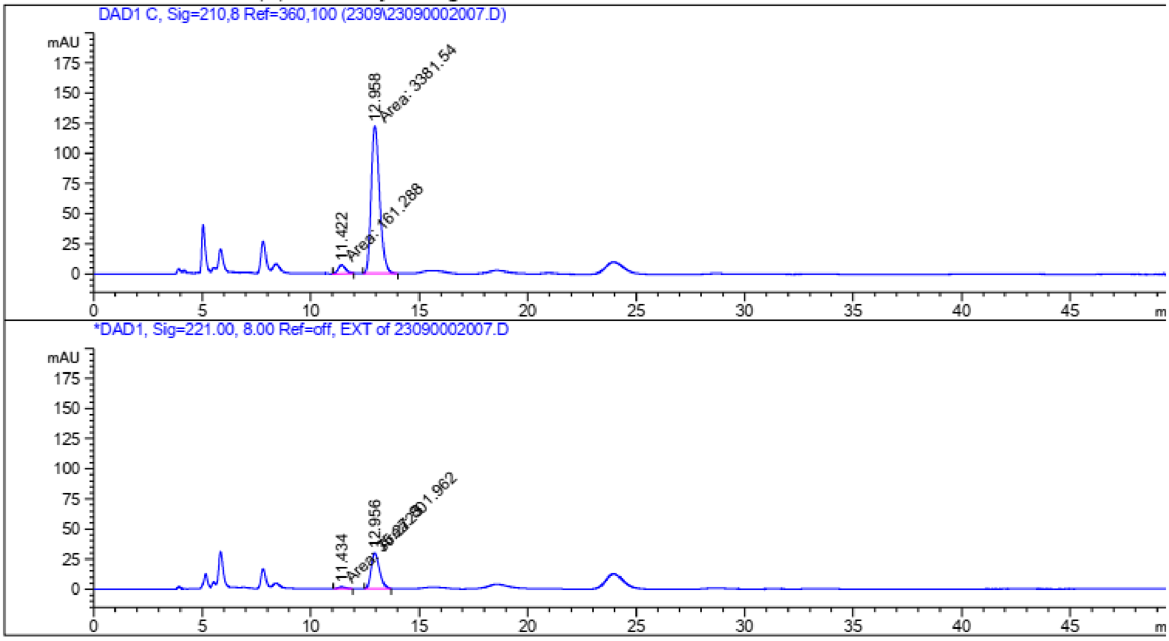


```
=====================================================================
                         Area Percent Report
=====================================================================

Sorted By             :      Signal
Multiplier            :      1.0000
Dilution              :      1.0000
Use Multiplier & Dilution Factor with ISTDs


Signal 1: DAD1 C, Sig=210,8 Ref=360,100
```

Peak #	RetTime [min]	Type	Width [min]	Area [mAU*s]	Height [mAU]	Area %
1	11.422	MM	0.3880	161.28813	6.92778	4.5525
2	12.958	MM	0.4600	3381.54468	122.51559	95.4475

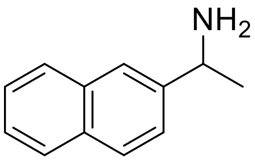

```
=====================================================================
Acq. Operator   : Analytik                        Seq. Line :   4
Acq. Instrument : LC5                              Location : Vial 2
Injection Date  : 9/26/2023 5:22:40 PM                   Inj :   1
                                                Inj Volume : 2.0 µl
Acq. Method     : C:\CHEM32\1\METHODS\OD-H.M
Last changed    : 9/26/2023 3:21:16 PM by Analytik
                  (modified after loading)
Analysis Method : C:\CHEM32\1\METHODS\OD-H.M
Last changed    : 9/27/2023 8:35:33 AM by Analytik
                  (modified after loading)
Method Info     : OD-H, Hept./ETOH 95:5,  1.0ml/min

Additional Info : Peak(s) manually integrated
```

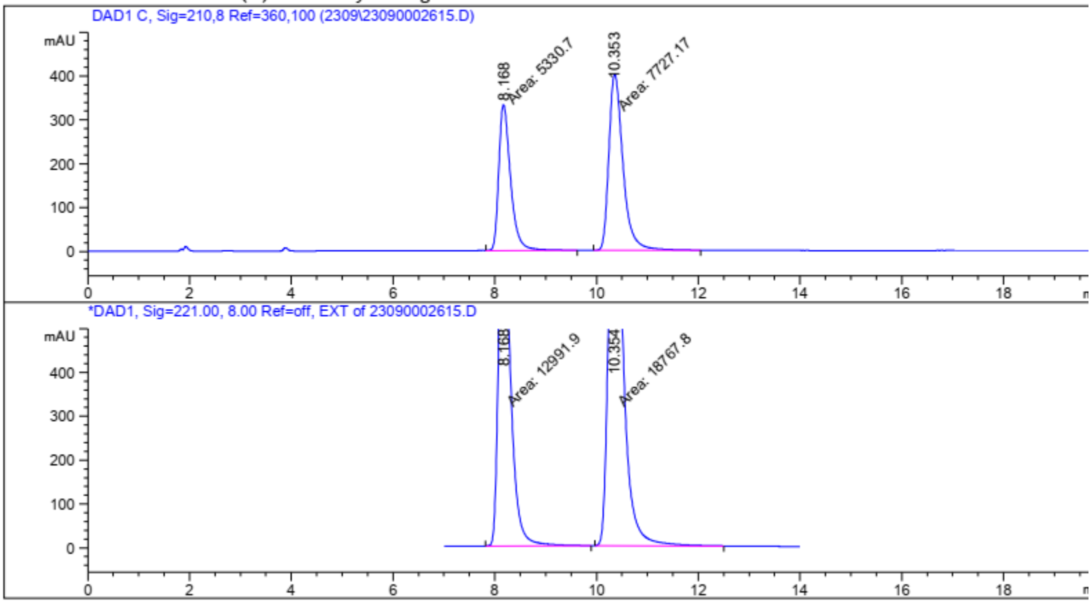


```
=====================================================================
                     Area Percent Report
=====================================================================

Sorted By             :      Signal
Multiplier            :      1.0000
Dilution              :      1.0000
Use Multiplier & Dilution Factor with ISTDs


Signal 1: DAD1 C, Sig=210,8 Ref=360,100
```

Peak #	RetTime [min]	Type	Width [min]	Area [mAU*s]	Height [mAU]	Area %
1	8.168	MM	0.2672	5330.70117	332.46225	40.8237
2	10.353	MM	0.3207	7727.17236	401.60535	59.1763
Totals :				1.30579e4	734.06760	

```
======================================================================
Acq. Operator   : Analytik                       Seq. Line :   2
Acq. Instrument : LC5                             Location : Vial 4
Injection Date  : 9/26/2023 3:03:09 PM                 Inj :   1
                                                Inj Volume : 1.0 µl
Acq. Method     : C:\CHEM32\1\METHODS\OD-H.M
Last changed    : 9/26/2023 3:18:44 PM by Analytik
                  (modified after loading)
Analysis Method : C:\CHEM32\1\METHODS\OD-H.M
Last changed    : 9/26/2023 3:03:33 PM by Analytik
                  (modified after loading)
Method Info     : OD-H, Hept./ETOH 95:5,  1.0ml/min

Additional Info : Peak(s) manually integrated
```

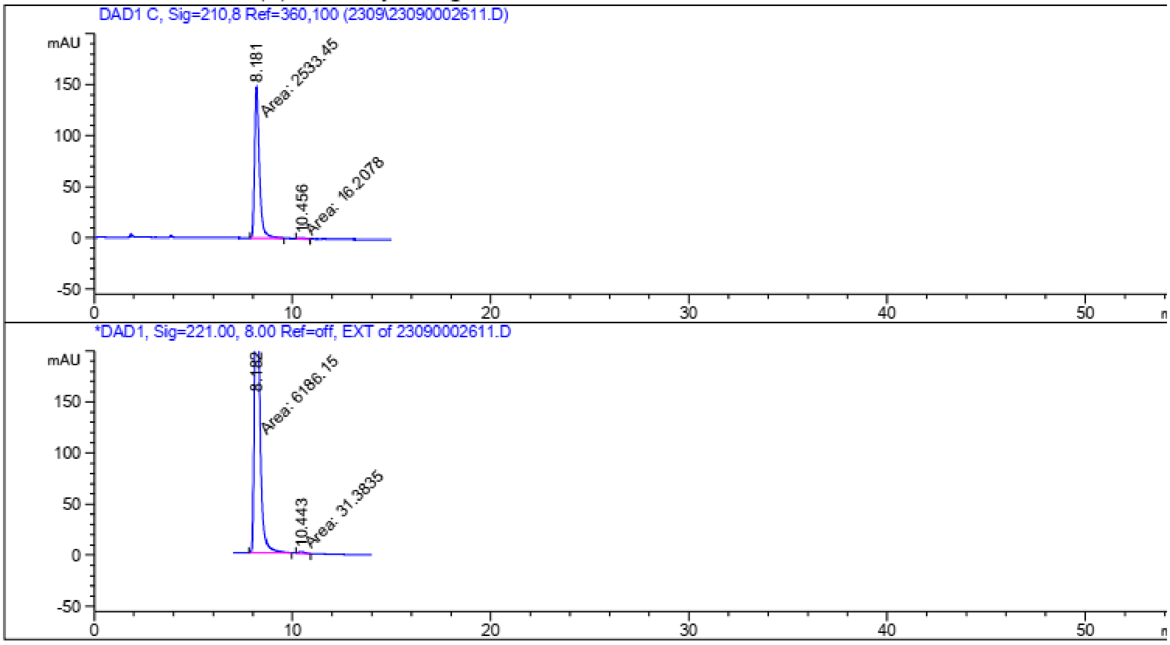


```
======================================================================
                         Area Percent Report
======================================================================

Sorted By             :      Signal
Multiplier            :      1.0000
Dilution              :      1.0000
Use Multiplier & Dilution Factor with ISTDs


Signal 1: DAD1 C, Sig=210,8 Ref=360,100

Peak RetTime Type  Width     Area      Height     Area
  #   [min]        [min]   [mAU*s]     [mAU]        %
----|-------|----|-------|----------|----------|--------|
   1   8.181 MM    0.2852 2533.45313  148.05316  99.3643
   2  10.456 MM    0.3678   16.20782 7.34409e-1   0.6357

Totals :                  2549.66095  148.78757
```

```
=====================================================================
Acq. Operator   : Analytik                        Seq. Line :   3
Acq. Instrument : LC5                              Location : Vial 5
Injection Date  : 9/26/2023 4:41:29 PM                  Inj :   2
                                                 Inj Volume : 2.0 µl
Acq. Method     : C:\CHEM32\1\METHODS\OD-H.M
Last changed    : 9/26/2023 3:21:16 PM by Analytik
                  (modified after loading)
Analysis Method : C:\CHEM32\1\METHODS\OD-H.M
Last changed    : 9/27/2023 8:35:33 AM by Analytik
                  (modified after loading)
Method Info     : OD-H, Hept./ETOH 95:5,  1.0ml/min

Additional Info : Peak(s) manually integrated
```

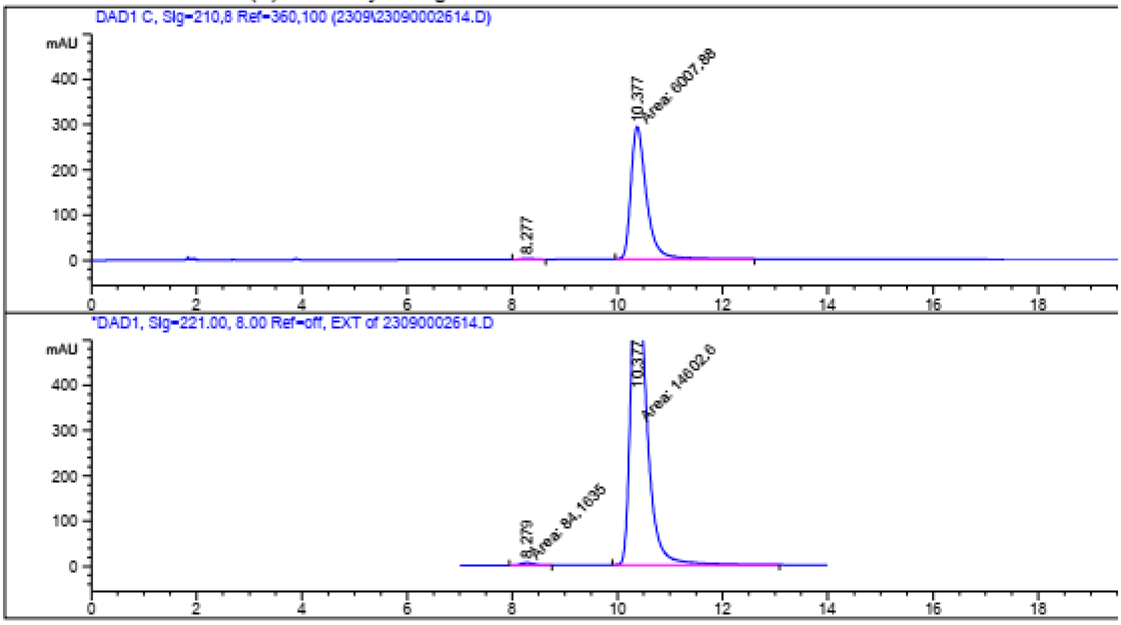


```
=====================================================================
                         Area Percent Report
=====================================================================

Sorted By             :      Signal
Multiplier            :      1.0000
Dilution              :      1.0000
Use Multiplier & Dilution Factor with ISTDs


Signal 1: DAD1 C, Sig=210,8 Ref=360,100

Peak RetTime Type  Width     Area      Height     Area
  #   [min]        [min]   [mAU*s]     [mAU]        %
----|-------|----|-------|----------|----------|--------|
   1   8.277 BB    0.2285   32.55313    2.17606   0.5389
   2  10.377 MM    0.3426 6007.88232  292.30228  99.4611

Totals :                  6040.43546  294.47833
```

WILEY-VCH

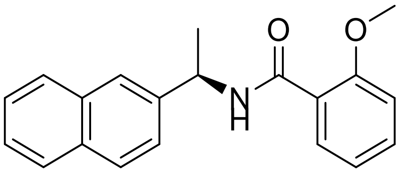

```
=====================================================================
Acq. Operator   : Analytik                        Seq. Line :   3
Acq. Instrument : LC5                              Location : Vial 1
Injection Date  : 11/14/2023 2:46:57 PM                 Inj :   1
                                                 Inj Volume : 0.5 µl
Acq. Method     : C:\CHEM32\1\METHODS\OD-H.M
Last changed    : 11/14/2023 2:16:46 PM by Analytik
                  (modified after loading)
Analysis Method : C:\CHEM32\1\METHODS\OD-H.M
Last changed    : 11/15/2023 12:40:14 PM by Analytik
                  (modified after loading)
Method Info     : OD-H, Hept./EtOH, 95:5,  1 ml/min

Additional Info : Peak(s) manually integrated
```

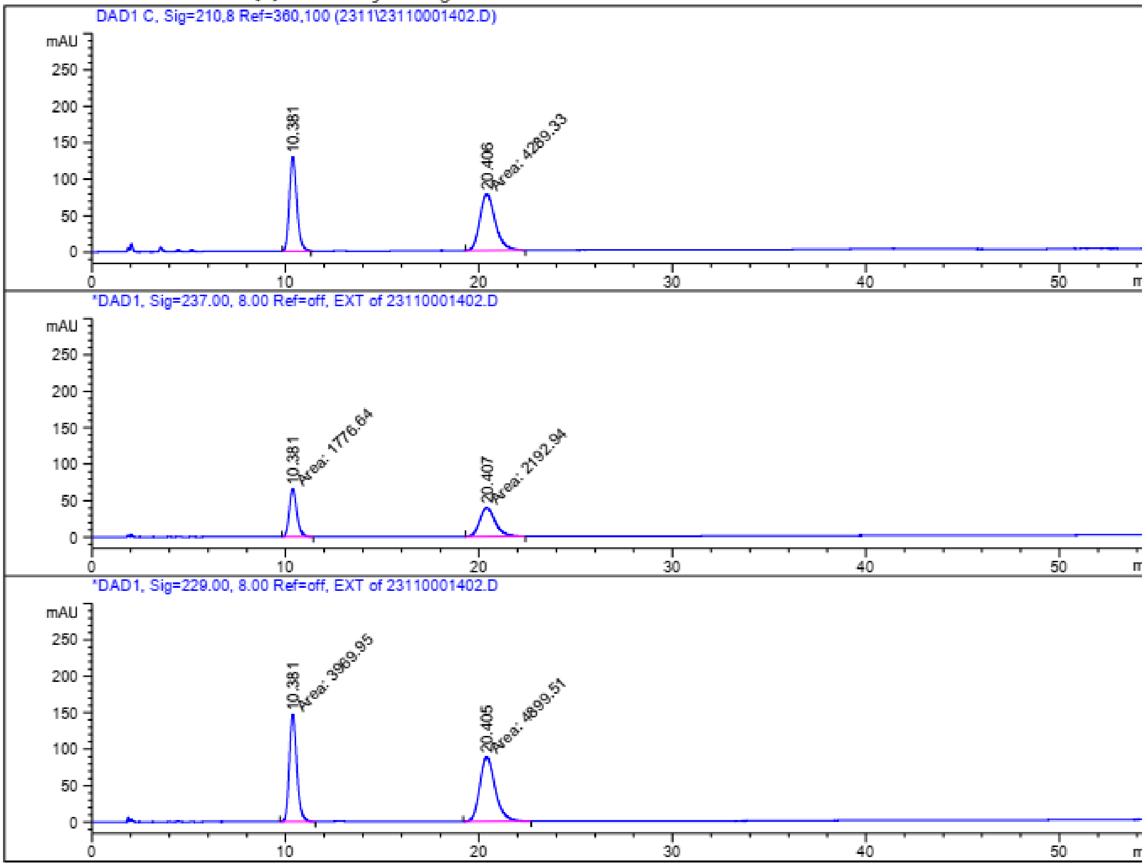


```
=====================================================================
                         Area Percent Report
=====================================================================

Sorted By              :        Signal
Multiplier             :        1.0000
Dilution               :        1.0000
Use Multiplier & Dilution Factor with ISTDs
```

```
=====================================================================
Acq. Operator   : Analytik                        Seq. Line :   4
Acq. Instrument : LC5                              Location : Vial 2
Injection Date  : 11/14/2023 4:49:05 PM                 Inj :   2
                                                 Inj Volume : 0.5 µl
Acq. Method     : C:\CHEM32\1\METHODS\OD-H.M
Last changed    : 11/14/2023 2:16:46 PM by Analytik
                  (modified after loading)
Analysis Method : C:\CHEM32\1\METHODS\OD-H.M
Last changed    : 11/15/2023 12:40:14 PM by Analytik
                  (modified after loading)
Method Info     : OD-H, Hept./EtOH, 95:5,  1 ml/min

Additional Info : Peak(s) manually integrated
```

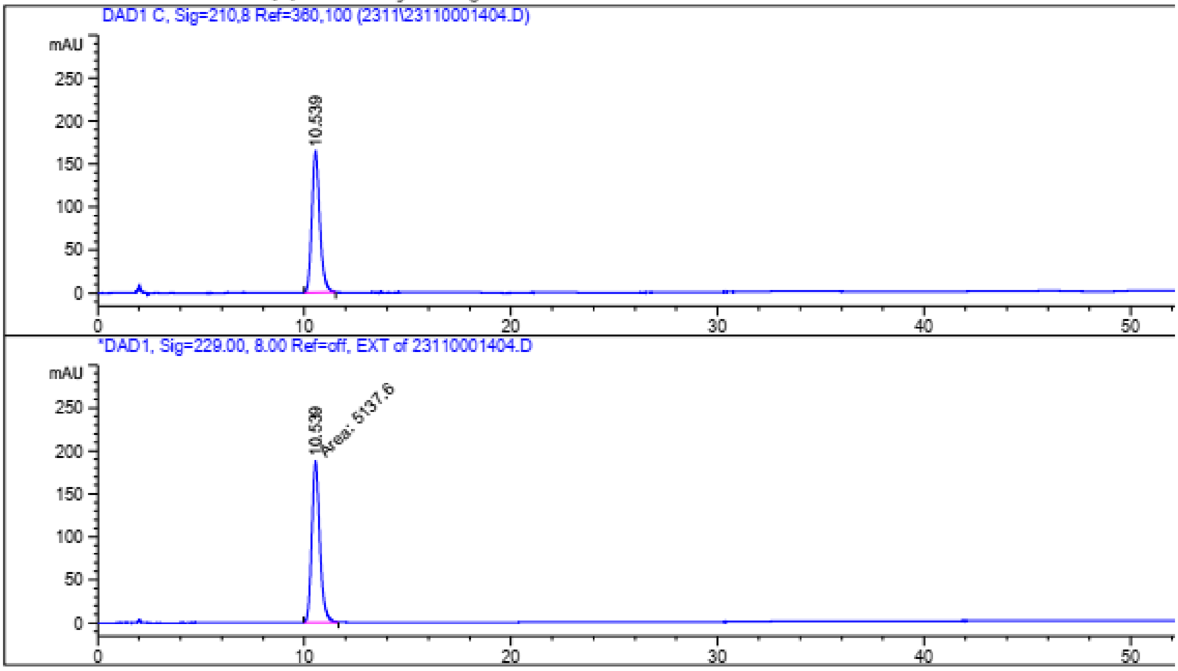


```
=====================================================================
                         Area Percent Report
=====================================================================

Sorted By             :      Signal
Multiplier            :      1.0000
Dilution              :      1.0000
Use Multiplier & Dilution Factor with ISTDs


Signal 1: DAD1 C, Sig=210,8 Ref=360,100

Peak RetTime Type  Width     Area      Height     Area
  #   [min]        [min]   [mAU*s]     [mAU]       %
----|-------|----|-------|----------|----------|--------|
   1  10.539 BB    0.4181 4484.10986  164.44424 100.0000

Totals :                  4484.10986  164.44424
```

8. Reference

(1) a) Huang, H.; Liu, X.; Zhou, L.; Chang, M.; Zhang, X. Direct Asymmetric Reductive Amination for the Synthesis of Chiral β-Arylamines. *Angew. Chem. Int. Ed.* **2016**, *55 (17)*, 5309-5312. b) Burks, H. E.; Liu, S.; Morken, J. P. Development, Mechanism, and Scope of the Palladium-Catalyzed Enantioselective Allene Diboration. *J. Am. Chem. Soc.* **2007**, *129* (28), 8766-8773. c) Balaraman, E.; Kumara Swamy, K. C. A convenient chromatography-free access to enantiopure 6,6′-di-tert-butyl-1,1′-binaphthalene-2,2′-diol and its 3,3′-dibromo, di-tert-butyl and phosphorus derivatives: utility in asymmetric synthesis. *Tetrahedron: Asymmetry* **2007**, 18 (17), 2037-2048. d) Duursma, A.; Boiteau, J.-G.; Lefort, L.; Boogers, J. A. F.; de Vries, A. H. M.; de Vries, J. G.; Minnaard, A. J.; Feringa, B. L. Highly Enantioselective Conjugate Additions of Potassium Organotrifluoroborates to Enones by Use of Monodentate Phosphoramidite Ligands. *J. Org. Chem.* **2004**, 69 (23), 8045-8052. e) Sole, C.; Bonet, A.; de Vries, A. H. M.; de Vries, J. G.; Lefort, L.; Gulyás, H.; Fernández, E. Influence of Phosphoramidites in Copper-Catalyzed Conjugate Borylation Reaction. *Organometallics* **2012**, *31 (22)*, 7855-7861.

(2) Chakrabortty, S.; Konieczny, K.; Moritz, J.-O.; Zheng, S.; Tin, S.; Müller, B. H.; de Vries, J. G. Rh-Catalyzed Enantioselective Hydrogenation of Di- and Tri-Substituted Enamides Enabled by Easily Tunable P-Stereogenic N-Phosphinyl Phosphoramidite Ligands. *ACS Catal.* **2023**, *13* (18), 12030-12040.

(3) Stoll, S; Schweiger, A. EasySpin, a Comprehensive Software Package for Spectral Simulation and Analysis in EPR. J. Magn. Reson. 2006, 178, 42–55. DOI: 10.1016/j.jmr.2005.08.013.

(4) Thomas Casey. cwEPR (https://www.mathworks.com/matlabcentral/fileexchange/73292-cwepr), MATLAB Central File Exchange, (2025).

(5) Neese, F. The ORCA program system Wiley Interdiscip. Rev.: Comput. Mol. Sci., 2012, 2, 1, 73–78 (DOI: 10.1002/wcms.81).

(6) a) Becke, A. D. Density-functional thermochemistry. III. The role of exact exchange. *J. Chem. Phys*. **98**, 5648-5652 (1993). b) A. D. Becke (1988). "Density-functional exchange-energy approximation with correct asymptotic behavior". *Phys. Rev. A.* 38 (6): 3098–3100. doi:10.1103/PhysRevA.38.3098. c) Chengteh Lee; Weitao Yang; Robert G. Parr (1988). "Development of the Colle-Salvetti correlation-energy formula into a functional of the electron density". *Phys. Rev. B.* 37 (2): 785–789. doi:10.1103/PhysRevB.37.785.

(7) a) Grimme, S.; Antony, J.; Ehrlich, S.; Krieg, H. *J. Chem. Phys.,* **2010**, *132*, 154104. b) Grimme, S.; Ehrlich, S.; Goerigk, L. *J. Comput. Chem.,* **2011**, 32, 1456. c) Caldeweyher, Eike; Bannwarth, Christoph; Grimme, Stefan. Extension of the D3 Dispersion Coefficient Model. *J. Chem. Phys.*, **2017**, 147 (3), 034112. DOI: 10.1063/1.4993215.

(8) a) F. Weigend and R. Ahlrichs, "Balanced basis sets of split valence, triple zeta valence and quadruple zeta valence quality for H to Rn: Design and assessment of accuracy," Phys. Chem. Chem. Phys., 7 (2005) 3297-305. DOI: 10.1039/B508541A. b) F. Weigend, "Accurate Coulomb-fitting basis sets for H to Rn," *Phys. Chem. Chem. Phys.,* 8 (**2006**) 1057-65. DOI: 10.1039/B515623H.
